# The characteristics, life circumstances and self-concept of 13 year olds with and without disabilities in Ireland: A secondary analysis of the Growing Up in Ireland (GUI) study

**DOI:** 10.1371/journal.pone.0229599

**Published:** 2020-03-13

**Authors:** Aoife Lily Gallagher, Rose Galvin, Katie Robinson, Carol-Anne Murphy, Paul F. Conway, Alison Perry

**Affiliations:** 1 Health Research Institute, School of Allied Health, University of Limerick, Ireland; 2 Ageing Research Centre, Health Research Institute, School of Allied Health, University of Limerick, Ireland; 3 School of Education, University of Limerick, Ireland; University College Dublin, IRELAND

## Abstract

**Background:**

Population-based studies provide important data to inform policy and service planning for vulnerable children in society. The aim of this study was to characterise social and educational circumstances and self-concept among a nationally representative sample of 13 year olds with developmental disabilities in Ireland.

**Methods:**

A cross-sectional, secondary analysis of data collected from the Growing Up in Ireland (GUI) study was conducted. Descriptive statistics were used to calculate the reported prevalence of disabilities as reported by parents. Differences across the groups (those with and without disabilities) were analysed in relation to gender, socio-economic and school factors. Special education support received in school was described. The association between low self-concept scores (as measured by the Piers Harris Self-Concept Scales 2) and disability type was examined by use of multi-level logistic regression.

**Results:**

Seventeen percent (17.36%) of the sample was reported to have a diagnosis of one or more developmental disabilities. Those with a disability were more likely to live in poorer households, have poorer health status, to experience more episodes of bullying at school, and to have more negative views of school (p<0.05) than their typically-developing peers.

Forty nine percent of children with developmental disabilities were not receiving support in school as reported by parents. Discrepancies in the nature of support received were identified across disability types. Adjusting for individual and school level factors, a disability diagnosis was associated with increased odds of low self-concept scores on three of five self-concept domains. Further associations were identified which differed across disability type.

**Conclusions:**

The findings show that 13 year olds with a disability in Ireland have complex social and educational needs. Findings also suggest significant levels of unmet educational need across this age group. Apparent inequities in access to support in school require further investigation. Reliable measures to provide robust prevalence figures about childhood disabilities in Ireland are needed.

## Introduction

Adolescence is a particularly turbulent time for most children but particularly for those with a disability. This is due to an increased risk of mental health problems, bullying and school refusal amongst this group [1–3]. Population-based studies provide important data for understanding both the individual characteristics of those with disabilities and the circumstances in which they live. Such studies are useful for informing policy and service planning, as well as for identifying gaps in research that may lead to enhanced health and education outcomes for vulnerable groups [4, 5]. In contrast to other countries, Ireland has only recently invested in a population-based study of children [6] and thus there are relatively few published studies about the lives of those with disabilities. The aim of this study was to describe the characteristics, life circumstances and self-concept scores of Irish children who have been diagnosed with a developmental disability.

Findings from population-based studies elsewhere highlight the importance of considering the broader context such as social or family circumstances, in addition to individual factors, in understanding the needs of such vulnerable groups. Children with disabilities are significantly more likely to live in social disadvantage and have chronic health problems as well as the health status of parents of children with disabilities being more likely to be poor [5, 7–10]. Further, if one child in the family has a diagnosis of a disability, there is an increased chance of them having siblings with a disability [8, 11–13].

From the limited number of studies conducted about children in Irish schools, findings suggest that the nature of the school influences the likelihood of being identified as having a disability and in receiving support. Mc Coy et al identified that if a child attends a school which is designated as serving a population of socio-economic disadvantage, that child is more likely to be identified and to receive support for their needs [14]. The gender mix of the school has also been shown to influence educational outcomes [14]. Understanding protective and risk factors in relation to the social, family and school context can inform service needs and, by extension, the quality of life for this population in Ireland.

An individual’s self-concept is concerned with how they view themselves [15]. It is a multi-dimensional construct involving an individual’s perception of their skills across a range of different domains, or areas of competence [16]. In school-aged children these include: academic/ educational status—how well an individual perceives themselves to be performing compared with their peers, in terms of school achievement and social acceptance, or how well an individual feels they are accepted by, or popular among, their peers [17]. According to Craven and Marsh, self-concept should be viewed as having a reciprocal relationship with performance and that a child’s self-concept related to different competencies evolves over time [18].

Self-concept is a useful measure for informing service planning and policy for two reasons. First, a positive self-concept is strongly associated with emotional well-being, academic achievement, maintaining safe and healthy relationships acquiring effective coping skills, and it is central to an individual’s adaptive functioning [19–21]. Therefore, it may be that identifying children at risk of poor self-concept and putting supports in place to promote a more positive self-concept may improve their educational outcomes. Second, self-concept develops in response to an individual’s environment [15]. Given that increasing numbers of children with disabilities are being educated in mainstream classrooms in Ireland [22], self-concept may be a useful indicator of how inclusive these classrooms are. Findings from studies of self-concept amongst children with disabilities are not conclusive. A New Zealand national survey of children with dyspraxia showed significantly lower global self-concept scores (in both academic and social self-concept domains) compared to their typically developing (TD) peers [23], whereas [24] reported an association between motor impairments and self-concept only for children with a low birth weight. For children who have specific learning difficulties (SpLD)—dyslexia and/or dyscalculia—[25] reported significantly lower academic self-concept than in their TD peers, but these findings were not consistent in a subsequent review conducted by [26].

For children with emotional behavioural disorders (EBD) the results of studies of self-concept are also mixed. [27] reported high self-concept scores in a clinical sample of children with EBD whereas [28] reported low self-concept scores in their population-based study. It may be that clinical samples include children with greater levels of need, which is then reflected in lower scores on self-concept.

Comparatively few studies have examined the association between the diagnosis and self-concept of children with Autistic Spectrum Disorder (ASD) and /or those with Speech, Language and Communication Needs (SLCN). Lindsay et al explored self-esteem, a related but different construct, in a clinical sample of children with developmental language disorders (a subset of SLCN) over two time points, and reported they had no difficulties [29]. By contrast, Conti-Ramsden et al reported poor self- concept in a sample of individuals with a history of SLCN [30]. In a population-based sample from the USA, [28] identified children with ASD as having lower self-concept related to social domains but not related to academic achievement, compared with TD peers.

A more consistent picture emerges for children with learning disabilities (LD). Earlier researchers suggested that children with such needs were at increased risk of low self-concept. However, from a more recent systematic review of 28 population-based studies, which included reliable multi-dimensional measures of self-concept, it was concluded that there was evidence of significantly lower self-concept scores for academic status only [25].

Like many other countries, children with a disability in Ireland are considered to have special educational needs (SEN) and are legally entitled to receive additional support in school in order to meet these needs [31]. This support may be delivered by a range of education and health professionals, such as a resource/learning support teacher, an educational psychologist, a special needs assistant (SNA), a speech and language therapist (SLT), an occupational therapist (OT) and/or a visiting teacher with additional skills and knowledge about children with visual/ hearing impairments.

In the Irish education system, a resource/learning support teacher is responsible for delivering interventions for any child with SEN across the school. Unlike learning support assistants in the UK, and para-professionals in the USA, SNAs are not responsible for meeting the *learning* needs of the child with SEN in school, rather, their *care* needs [32].

Visiting teachers in Ireland are responsible for a caseload of children with visual and/or hearing impairment in a particular geographical region, until the end of their compulsory education. Children in second-level education in Ireland who are considered to have emotional and/or behavioural difficulties, such as poor social skills, low self-esteem or difficulties in maintaining friendships and concentration, can access additional support as part of a national behaviour programme. The SEN support received from these professionals by children in second-level education in Ireland has not been documented, resulting in a paucity of data that can inform policy and service planning.

In summary, population-based samples may provide important data to guide policy and service planning for vulnerable groups. Findings from studies elsewhere have highlighted the importance of considering social and school factors, as well as individual characteristics, in understanding the service needs of vulnerable groups. Unlike other countries such as England [33], there are limited published data on the prevalence of developmental disabilities, special educational needs and/or support being received by children in second-level education in Ireland. Self-concept is considered an important predictive of positive educational outcomes but findings from studies of self-concept in children with disabilities are not consistent.

The objectives of this study are; (i) to document the reported prevalence and type of disability found in a population-based sample of 13 year olds in Ireland, (ii) to characterise the socio-demographics and school context of these children with disabilities, (iii) to describe the nature of supports being received by this group in their first year of second-level education and, (iv) to explore the association between self-concept and the presence of a disability.

## Materials and methods

### Study design

This study was a cross-sectional, secondary analysis of quantitative data that were collected as part of the child cohort of the Growing Up in Ireland (GUI) study [34]. The GUI is a nationally-representative sample of children living in Ireland.

The Strengthening the Reporting of Observational Studies in Epidemiology (STROBE) guidelines were followed in the conducting and reporting of this study [35].

### Ethics

Ethics approval in respect of the Growing Up in Ireland Child Cohort was granted by the Health Research Board’s Research Ethics Committee (HRB REC-17/11/06). Written consent was obtained for all participants in the study. Assent was also sought from the children who participated. Re-analysis of the GUI dataset does not require additional ethical approval in accordance with the Central Statistics Office of Ireland who hold the Growing Up in Ireland dataset.

### Participants

For the GUI study, sampling was undertaken in two stages using a clustered sample design.

A stratified random sample of 1,105 schools was identified from a possible 3,000 schools in Ireland. All school types were included in the sampling population including mainstream and special schools. From this, 910 schools (82.3%) agreed to participate. From these schools, a random sample of students was selected. For smaller schools (those with less than 40 students) all students were included. Eight thousand five hundred (8,500) children participated at 9 years of age.

At time 2 (when the children were 13 years) the response rate was 91.2%, resulting in a final sample of 7,525 child participants. The data analysed for this study were limited to the sample who participated in data collection at time 2.

Relevant data for this secondary analysis were collected from questionnaires completed by the children who participated in the study (completed with an interviewer present), their primary caregiver (parent) and by the Principal of the school they attended [36]. Questionnaires are included as supplementary information for ease of reference (S1 File).

### Data collection

#### Disability status

Information about each child’s disability status was obtained from the primary caregiver questionnaire. Questions included: the type of developmental disability diagnosis received, whether or not the child had been diagnosed with the disability by a professional, and the number of diagnoses the child had received. The primary caregiver was asked if their child had any of the following diagnoses/ disabilities: a physical disability (including a visual and/or a hearing impairment); a specific learning disability (SpLD); a learning disability (LD); autism spectrum disorder (ASD); an emotional/behavioural disorder (EBD) and a speech/language difficulty. Further categories not reported in this study included: a disability associated with a particular syndrome, slow progress (reasons unclear), and ‘other,’ where the parent could add their own comments.

In this paper, we refer to some of the categories in language which is slightly different from that used in the questionnaire. We use speech, language and communication needs (SLCN) rather than “speech or language difficulty” when referring to children with these needs for consistency with the broader literature. This classification includes all possible profiles of need in this area [37]. We refer to the category of “physical disability” as physical and sensory disability (PSD), as we consider this to describe the category of need more accurately.

#### Socio-economic status

Two variables, *employment status* and *income*, were used as measures of SES. The Economic and Social Research Institute of Ireland (ESRI) who manage the GUI dataset derived these. Employment status is determined based on the higher level of the primary and the secondary caregiver’s occupations. Of note, it was not possible to categorise the employment of all families’ who participated in the study. Income data from the dataset were derived as a categorical variable by the ESRI, and are presented as quintiles. Whether the child lived in a single parent family or not was also used for analyses.

#### Health status

The primary caregiver questionnaire included two questions about whether or not the parent themselves had a chronic illness or any confirmed medical diagnosis, and whether their child had any chronic health condition.

#### School context

Four variables in relation to the school context of the children in the study were collected. First, a variable on whether or not the school was part of the “Delivering Equality of Opportunity in Schools” (DEIS) programme was included. DEIS is a programme in Ireland that allocates extra funding to schools that serve populations of high social disadvantage. The DEIS designation was determined from the school Principals’ questionnaires. The second variable was the gender mix of the school that the child attended.

A third and fourth variable, based on self-report data from the children were included. The third was the number of episodes of bullying reportedly experienced by the study child in school in the previous three months. The fourth related to each child’s views of school.

#### Additional support in school

As part of the primary caregiver questionnaire, respondents were asked to identify the different health and/or education professionals involved in delivering this additional support to their child in school. Options included: a resource/learning support teacher, a school psychologist, an educational psychologist (EP), a special needs assistant (SNA), a speech and language therapist (SLT), support as part of a behaviour management programme, and/or a visiting teacher. Further options included whether the child received technical assistance and/or transport services. These latter options were not included in our analyses.

In the primary caregiver questionnaire, parents were asked their view of the adequacy of the support being received by their child. A Likert answer included *excellent*, *adequate*, *barely adequate*, *don’t know*. Respondents were also able to tick a box if their child was not receiving any support in school.

#### Self-concept

In the GUI data, self-concept was measured using the Piers-Harris Children’s Self Concept Scale-2^nd^ Edition (PHCSCS-2) [38]. The PHCSCS was standardised on a population of 1,387 children aged from 7 to 18 years and has been shown to have good internal consistency and test-retest reliability (Cronbachs α .91) [38]. Internationally, this is one of the most widely used measures of self-concept [39]. The PHSCS-2 has six domains that relate to self-concept, measured as six subscales that include behavioural adjustment, intellectual/school status, physical appearance, freedom from anxiety, popularity and happiness/satisfaction. The child’s view of their self-concept is scored as either 1 or 0, depending on their yes or no responses to a series of statements across each domain. A total score for each subscale can be calculated, as well as a composite score. Each subscales has a different score range–for example, intellectual/ school status is scored from 0 to 16; behavioural adjustment and freedom from anxiety are scored from 0 to 14; physical appearance and attributes from 0 to 11; and happiness and satisfaction range from 0 to 10. For all subscales, the higher the score, the higher (better) the self-concept. Raw scores for each subscale and a total raw score for the complete test in the GUI dataset were available; as was a derived variable to categorise the total score and the scores from each subscale into five levels, ranging from high to low self-concept. The derived categories were based on clinical cut-offs, as specified by the PHSCS-2. These were recoded into a binary outcome of low self-concept (y/n) for this secondary analysis.

#### Analysis

Analyses were conducted using STATA software [40]. Weighting based on a minimum information loss algorithm has been calculated to adjust for differences between the GUI sample and the population at aged 13 years. These adjustments were used during all analyses to allow for inferences to be made about the population from which the participants were sampled. Further information regarding sampling and weighting in GUI is included as supplementary information (S2 File).

We took a cautious approach when managing missing data. Ten cases in the dataset had incomplete reported data about disability status so these were omitted from the analyses. Descriptive statistics were used to characterise the population. The reported prevalence of children of this age with a diagnosis of one or more developmental disabilities was then calculated as a percentage of the total sample. Variables in relation to SES & income, school context, health status of child and parent, the child’s views of school, and whether the parent reported the child as having experienced bullying in the last three months, were compared to children with and without disabilities, using chi-square tests.

For SEN support, the proportion of children receiving support from a range of health and education professionals was calculated. A series of multilevel logistic regression analyses were used to identify significant associations between disability status and disability type and self-concept scores, adjusting for individual and school level factors. Individual factors included in the analysis were SES, income, gender and self-reported episodes of bullying. School factors included DEIS status and gender mix of school. Adjusted odds ratios (adj ORs) and 95% confidence intervals (CIs) were reported in relation to each factor. All analyses conducted were weighted (aka statistically adjusted) to minimise sampling bias. Statistical significance was set at p<0.05.

## Results

### Reported prevalence of disability

Of the total children for whom there were data on disability in the GUI sample (n = 7,515), 1,304 (17.36%) had reportedly received one or more diagnosis of a developmental disability. A total of 730 males (14.48%) received ≥ 1 disability diagnosis compared to 574 females (11.91%). The proportion of children by number of diagnoses is shown in Table 1.

**Table 1:** Proportions of children with and without disabilities as reported by primary caregivers in the child cohort of the GUI dataset^a^.

N diagnoses	N children		Total N children	% of total sample (n = 7515)
	Male	Female		
0	2949	3,261	6,211	82.64%
1	533	457	990	13.17%
2	124	73	197	2.62%
3	40	29	69	0.92%
4	33	15	48	0.65%

^a^ Adjusted figures using weighting based on a minimum information algorithm calculated by the Economic and Social Research Institute.

Table 2 displays the types of disability diagnoses reported by primary caregivers. The most common diagnosis allocated was SpLD (6.91%). PSD, which included children with a diagnosed hearing impairment and/or visual impairment as well as sensory needs, was the second most common diagnosis (6.5%), and a diagnosis of LD was the third (2.93%). The reported prevalence of SLCN was 2.12%, EBD was 1.54%, and ASD was the least common diagnosis (1.29%). Of note, some children had more than one diagnosis.

**Table 2:** Type and number of disability diagnoses as reported by primary caregivers in the child cohort of the GUI dataset^a^.

Type of Diagnosis	N Children		Total	% of total sample (n = 7515)
	Male	Female		
SpLD	295	225	520	6.91%
PSD	249	240	489	6.50%
LD	130	90	220	2.93%
SLCN	105	55	160	2.12%
EBD	83	35	118	1.57%
ASD	77	20	97	1.29%

^a^ Adjusted figures using weighting based on a minimum information algorithm calculated by the Economic and Social Research Institute; SpLD = specific learning difficulties; PSD = physical and sensory disability; LD = learning disability; SLCN = speech, language and communication needs; EBD = emotional behavioural disorder; ASD = autistic spectrum disorder.

### Group differences for socio-demographics, health status and school context

The socio-demographic data of children with and without a diagnosis of developmental disability are summarised in Table 3.

**Table 3:** Characteristics of children with and without a disability in the GUI dataset^a^.

Variables	Disability group (n = 1304)		TD peers (n = 6211)		P value^b^
	N	%	N	%	
**Gender**					**P<.001**
Male	730	21.50%	2949	78.50%	
Female	575	16.69%	2361	83.32%	
**SES**^c^					**P<.001**
Professional	169	7.83%	1009	11.79%	
Managerial	477	27.86%	2535	35.55%	
Non-manual	217	16.28%	1169	19.35%	
Skilled manual	200	18.91%	688	13.63%	
Semi-skilled manual	98	11.51%	449	9.73%	
Unskilled	22	2.59%	60	1.62%	
**Income**					**P<.001**
Lowest	225	25.29%	852	19.84%	
2^nd^	236	21.36%	947	20.21%	
3^rd^	223	20.05%	1088	19.44%	
4^th^	227	19.7%	1340	20.33%	
Highest	241	13.50%	1509	20.14%	
**Lone parent family**	56	8.73%	329	8.46%	P = 0.72
**Chronic health condition**					
Child	340	28.44%	424	7%	**P<.001**
Parent	307	25.05%	1023	17.31%	**P<.001**
**DEIS**^d^ **school**	223	25.06%	770	16.60%	**P<.001**
**Views of school**					**P<.001**
I like it very much	355	24.57%	1956	29.57%	
I like it quite a bit	388	28.72%	2074	33.1%	
I like it a bit	365	31.63%	1513	25.55%	
I don’t like it very much	105	9.55%	429	7.94%	
I hate it	47	4.87%	132	2.87%	
I don’t know	11	.62%	51	.94%	
**Number of episodes of bullying**	224	18.39%	495	8.40%	**p<.001**

^a^ Chi–squared tests were used to compare differences in proportions across the groups;

^b^ P values have been rounded to 3 decimal places as per journal convention; TD = typically-developing;

^c^ a further category whereby “no class” could be assigned based on occupation has been omitted so percentages do not add up to 100%;

^d^ DEIS is a school recognised as part of national government programme as serving an area of social disadvantage.

The presence of ≥ 1 disability diagnosis was significantly higher in boys than in girls (p<.001). A significantly greater proportion of children with a disability had a parent of lower employment status than did the children who had no diagnosis (p<.001) and they lived in a family with lower income levels than their TD peers. More children with a diagnosed disability had a chronic health condition, as did their parents, compared with their TD peers and the parents of the TD peers. A significantly greater proportion of children with a disability attended a DEIS school than did children without a diagnosis. There were significant differences between the groups in terms of their self-reported view of school, with more children who had a disability reporting negative views of school compared with their TD peers. A significantly higher proportion of children with a diagnosed disability were reported to have been bullied in the last three months in school than were children without a diagnosis (p<.001).

### Support in school

The views of parents about support in school are presented in Table 4.

**Table 4:** Primary caregiver views of the adequacy of supports received by their child with a disability in school^a^.

View of adequacy of support ^b^	Of total sample with a disability diagnosis (n = 1304)	SpLD (n = 520)	PSD (n = 489)	LD (n = 220)	SLCN (n = 160)	ASD (n = 97)	EBD (n = 118)
Excellent	14.70%	18.84%	10.43%	19.54%	20.00%	19.58%	22.03%
Adequate	19.81%	25.00%	10.02%	27.27%	25.00%	38.14%	27.96%
Barely adequate	15.50%	24.42%	07.36%	26.36%	24.37%	27.83%	28.81%
Don’t know	01.30%	00.57%	01.00%	00.00%	01.00%	00.00%	00.84%
No support received	49.00%	31.15%	71.16%	26.81%	30.00%	14.43%	20.33%

^a^ Adjusted figures using weighting based on a minimum information algorithm calculated by the Economic and Social Research Institute;

^b^ Categories as per wording in survey question; SpLD = specific learning disability; ASD = autistic spectrum disorder; SLCN = speech, language and communication needs; PSD = physical and sensory disability; LD = learning disability; EBD = emotional behavioural disorder.

Primary caregivers were asked to give their view of the adequacy of the support being received by their child. Those not receiving any report could select that as an answer.

In terms of adequacy of support, 14.70% of parents reported that the support received by their child in school was excellent, 19.81% reported that the support was adequate, 15.50% reported the support was barely adequate and 1.30% reported they did not know. Forty nine percent of parents reported that their child was not receiving any support in school. Thirty one percent of children with a diagnosis of SpLD, 71.16% of those with a diagnosis of PSD, 26.81% of those with a diagnosis of LD, 30% of children with SLCN, 14.43% of those with a diagnosis of ASD and 20.33% of those with EBD were reported by the primary caregiver as not receiving any support in school.

Parents were also asked about the nature of support received by their child in school. The results by disability type are presented in Table 5.

**Table 5:** Proportion of children receiving supports in school by disability type^a^.

Professionals involved	SpLD %	PSD %	LD %	SLCN %	ASD %	EBD %
Learning/resource teacher	62.99	13.36	29.07	16.11	12.77	15.32
SNA	51.37	26.36	50.90	37.27	35.45	28.18
Visiting teacher	46.15	65.38	46.15	30.76	19.23	26.92
SLT	31.81	29.54	63.63	17.11	29.59	20.45
Behaviour management programme	38.88	38.88	38.88	38.89	33.33	38.88
School Psychology	47.17	24.53	49.06	39.62	24.53	18.87
NEPs	62.22	13.33	40	35.55	28.89	26.67

^a^ Adjusted figures using weighting based on a minimum information algorithm calculated by the Economic and Social Research Institute; SpLD = specific learning disorder; PSD = physical and sensory disability; LD = learning disability; SLCN = speech, language and communication needs; ASD = autistic spectrum disorder; EBD = emotional behavioural disorder; SNA = special needs assistant; SLT = speech and language therapist; NEPS, = national educational psychology service.

Sixty nine percent of children with SpLD, 13.36% of those with PSD, 29.07% with LD, 16.11% with SLCN, 12.77% with ASD and 15.32% of those with EBD reportedly received support from a resource/learning support teacher.

Fifty one percent of children with SpLD, 26.36% of those with PSD, 50.90% of those with LD, 37.27% of those with SLCN, 35.45% of those with ASD and 28.18% of those with EBD reportedly received support from an SNA.

Forty six percent of children with SpLD, 26.36% of those with PSD, 50.90% of those with LD, 37.27% of those with SLCN, 35.45% of those with ASD and 28.18% of those with EBD reportedly received support from a visiting teacher.

Thirty one percent of children with SpLD, 29.54% of those with PSD, 63.63% of those with LD, 17.11% of those with SLCN, 29.59% of those with ASD and 20.45% of those with EBD reportedly received support from an SLT.

Thirty eight percent of children with SpLD, 38.88% of those with PSD, 38.88% of those with LD, 38.89% of those with SLCN, 33.33% of those with ASD and 38.88% of those with EBD reportedly received support from a national behaviour support programme.

Forty seven percent of children with SpLD, 24.53% of those with PSD, 49.06% of those with LD, 39.62% of those with SLCN, 24.53% of those with ASD and 18.87% of those with EBD reportedly received support from a school psychologist. Sixty two percent of children with SpLD, 13.33% with PSD, 40% with LD, 35.55% with SLCN, 28.89% with ASD and 26.67% of children with EBD reportedly received assistance from the national educational psychology service.

### Association between a diagnosis of disability, disability type and scores of low self-concept scores

A series of multilevel logistic regression analyses were used to identify significant associations between disability status and disability type and self-concept scores, adjusting for individual and school level factors. Individual factors included in the analysis were SES, income, gender and self-reported episodes of bullying. School factors included DEIS status and gender mix of school. The adjusted Odds Ratios (adj ORs) for low self-concept scores are presented in Table 6.

**Table 6:** Adjusted odds ratios of likelihood of low self-concept scores by disability type^a^.

	Total self-concept score^b^	BEH	INT	PHY	FRE	POP	HAP
		adj ORs^c^	adj ORs	adj ORs	adj ORs	adj ORs	adj ORs
	(95% CI)	(95% CI)	(95% CI)	(95% CI)	(95% CI)	(95% CI)	(95% CI)
**Disability diagnosis**	1.37* **(1.06–1.76)**	1.23* **(1.05–1.44)**	1.56* **(1.30–1.88)**	0.85 (0.69–1.03)	0.95 (0.78–1.16)	1.38* **(1.17–1.63)**	0.74* **(0.61–0.89)**
**SpLD**	1.49* **(1.04–2.14)**	1.08 (0.85–1.37)	2.23* **(1.70–2.90)**	0.72* **(0.54–0.96)**	0.89 (0.67–1.18)	0.99 (0.76–1.30)	0.86 (0.66–1.13)
**PSD**	1.43 (0.98–2.08)	0.91 (0.71–1.16)	0.94 (0.71–1.25)	1.30 (0.97–1.74)	1.00 (0.75–1.34)	1.44* **(1.12–1.85)**	0.64* **(0.48–0.85)**
**LD**	1.38 (0.80–2.40)	1.66* **(1.15–2.40)**	1.95* **(1.28–2.97)**	0.83 (0.53–1.30)	0.77 (0.50–1.20)	1.30 (0.88–1.91)	0.72 (0.47–1.11)
**SLCN**	1.24 (0.65–2.37)	1.28 (0.84–1.95)	1.17 (0.72–1.92)	0.68 (0.40–1.15)	0.59 (0.35–1.00)	1.70* **(1.10–2.63)**	1.31 (0.82–2.10)
**EBD**	2.97* **(1.49–5.97)**	2.04* **(1.25–3.30)**	1.06 (0.62–1.80)	1.17 (0.42–1.31)	0.96 (0.55–1.70)	1.38 (0.83–2.28)	0.56* **(0.32–0.98)**
**ASD**	1.47 (0.66–3.28)	0.99 (0.59–1.68)	1.09 (0.59–1.99)	0.88 (0.47–1.65)	0.98 (0.53–1.81)	3.40* **(1.96–5.91)**	0.82 (0.45–1.52)

^**a**^ Multi-level logistic regression analyses were used to identify associations between disability diagnoses and self-concept scores, adjusting for individual (SES, Income, gender and self- reported episodes of bullying) and school level factors (DEIS status and gender mix of school). Weighting based on a minimum information algorithm calculated by the Economic and Social Research Institute was used in all of the analyses;

^b^ as measured by the Piers-Harris Children’s Self-Concept Scale- 2^nd^ Edition;

^c^ adj OR = adjusted odds ratio; BEH = behavioural adjustment; INT = intellectual/School Status; PHY = physical appearance; FRE = freedom from anxiety; POP = popularity; HAP = happiness and satisfaction; PSD = physical and sensory disability; SpLD = specific learning difficulties; LD = learning disability; EBD = emotional behavioural disorder; SLCN = speech, language and communication needs; ASD = autistic spectrum disorder.

* = p<0.05.

Overall, there was a significant association between having a diagnosis of a developmental disability and low self-concept scores (adj OR = 1.37, 95% CI 1.06–1.76). In relation to specific self-concept domains, there was a significant association between having a disability and low behavioural adjustment scores (adj OR = 1.23, 95%; CI 1.04–1.44), low intellectual/ school status scores (adj OR = 1.56, 95%; CI 1.30–1.88 and low popularity scores (adj OR = 1.38, 95%; CI 1.17–1.63) and having a diagnosis reduced the odds of low happiness scores (adj OR = .74, 95%; CI 0.61-.89).

Associations differed across disability type. Overall, children with a diagnosis of SpLD showed increased odds of low self-concept scores (adj OR = 1.49, 95%; CI 1.04–2.14). There were increased odds of low self-concept scores with intellectual/school status (adj OR = 2.23, 95%; CI 1.70–2.90). Conversely, a diagnosis of SpLD reduced the odds of low self-concept scores for physical appearance (adj OR = 0.72*, 95% CI 0.54–0.96). A diagnosis of PSD did not increase the overall likelihood of low self-concept scores. However, there were significantly increased odds of low scores in popularity (adj OR = 1.44, 95% CI 1.12–1.85).

Those with a diagnosis of PSD were less likely to have low self-concept scores related to happiness (adj OR = 0.64, 95% CI 0.48–0.85). Those with a diagnosis of LD showed an increased odds of low concept related to behavioural adjustment (adj OR = 1.66, 95% CI 1.15–2.40) and intellectual / school status (adj OR = 1.95, 95% CI 1.28–2.97). A diagnosis of SLCN increased the odds of having low scores in popularity (adj OR = 1.7, 95% CI 1.10–2.63), as did having a diagnosis of ASD (adj OR = 3.4, 95%; CI 1.96–5.91).

Those with a diagnosis of EBD showed increased odds of having low self-concept scores overall (adj OR = 2.97, 95%; CI 1.49–5.97) as well as behavioural adjustment scores (adj OR = 2.04, 95%; CI 1.25–3.30). A diagnosis of EBD was associated with a reduced odds of low self-concept in the domain of happiness (adj OR = 0.56, 95% CI 0.32–0.98).

## Discussion

This is one of the first studies to characterise the life circumstances and self-concept of Irish children with developmental disabilities using data from a population-based sample. From examining this sample (N = 7,515) of 13 year olds in Ireland, 17.07% of children were reported to have a diagnosis of one or more developmental disabilities by their primary caregiver. We found a significant gender difference among those identified as having a disability, with more boys than girls in the sample having a diagnosed disability. Significant differences were also identified between groups in relation to socio-economic status with more children with a disability in the lower socioeconomic groups. Our findings were that both children with a disability and their parents had poorer health status when compared to their typically-developing peers. We also found that more children with disabilities reported that they disliked school and had experienced more episodes of bullying than did their TD peers.

Based on reports from primary caregivers, 49% of 13 year olds with a diagnosed developmental disability were not in receipt of SEN support from either health or education professionals in the first year of second-level education. Overall, adjusting for individual and school level factors, there was an increased likelihood of low self-concept scores amongst those with a disability across three of five self-concept domains (behaviour, intellectual status and popularity) but a reduced odds of having low self-concept scores related to happiness. The odds of low self-concept scores differed across disability type.

Overall the reported prevalence of disability in this study was broadly consistent with the findings of recent estimates from a meta-analysis of international prevalence of population-based studies [41]. Parent reports of a diagnosis of SpLD and ASD were also in line with international prevalence estimates from the USA and the UK [42–45]. However, the proportion of children reported by parents as having a diagnosis of SLCN is low (1.63%) compared with other prevalence studies. In Australia for example, in a study of children of a similar age, based on teacher reports, a prevalence of 12.3% was identified [46]. It may be that children with SLCN in Ireland are disproportionately under-diagnosed relative to other disability groups. Comparing the reported prevalence of children with a diagnosis of SLCN by primary caregivers in this study with school census data related to special educational needs (SEN) in the UK [33, 47] shows even greater disparity. Most recent SEN data in the UK has identified SLCN as the most common category of reported need (23%) in children of school-age with SEN [33] and yet in this population-based sample, a diagnosis of SLCN was one of the least prevalent of the disability groups. It may be that robust, needs—based data, rather than data based on diagnostic categories may be more useful in planning supports of school-aged children in Ireland.

It is not possible to draw inferences about the discrepancy between parental reports of EBD in this sample and prevalence figures elsewhere, due to differences in how such needs are categorised. In the USA for example, a much higher prevalence of EBD is reported [48]. However, in those studies mental health diagnoses are included as one disability category whereas, in the GUI dataset, EBD and mental health diagnoses are categorised separately. It is likely that the lower reported prevalence of 1.27% in the Irish sample is accounted for by differences in categorisation, rather than an under-identification of needs per se.

In terms of socio-demographics, more boys than girls in this sample presented with a disability; a finding which has been well-documented in other population-based studies [49]. Irish children with disabilities are more likely to experience higher levels of poverty and social disadvantage, to attend a school with increased levels of social deprivation, and to have poorer health status, consistent with previous population-based studies from the UK [7] and the USA [13]. Our data also show poorer health status for parents of children with disabilities, in line with previous study findings [50, 51]. For lone parent families, Ireland and the UK have similar proportions of families with one caregiver (one in four) [52]. In contrast to Blackburn et al. [7], we found no difference in the proportion of lone parent families with a child with a disability, compared to those without.

In this sample, more children with a diagnosis of a developmental disability reported a negative view of school and more episodes of bullying than did their TD peers. Negative views of school were found among the same cohort 4 years earlier, at 9 years, when they attended primary education—which McCoy and Banks [53] found to be mediated by children’s levels of academic engagement and peer relations. These reports of bulling are consistent with a comprehensive review of the literature from the UK on bullying and disability [54].

The large proportion of children (49%) not receiving support in school identified in the study was surprising, as Ireland is a relatively large investor in education, in terms of percentage of public expenditure, compared to other OECD countries [55]. However this finding is consistent with views of school principals, reported as part of a national review of Irish schools conducted by Rose et al. [22]. This is also consistent with parents’ view of support reported elsewhere [56].

It is necessary to examine these findings against data held by the Irish Health Services’ Executive and/or the National Council for Special Education; the two public bodies that are responsible for providing SEN support to schools in Ireland in order to contextualise them further. Input to these datasets is, unfortunately, currently voluntary, and therefore may not include all relevant data.

Discrepancies in the types of SEN support received by children were evident, not readily accounted for by the nature of a child’s diagnosis. For example, the lowest proportions of children receiving speech and language therapy support from an SLT in school were those with SLCN (17%) and ASD (29.59%), compared with 63.93% of those with a diagnosis of LD. This may reflect differences in access to SLT services for children in school, based on their disability type. Currently, if a child has more than one disability diagnosis they receive support in school from a multidisciplinary team including an SLT. It is likely that children with a learning disability will have associated SLCN and so might have access to SLT supports through these services. If, on the other hand, a child has just one diagnosis, in this instance related to SLCN, they usually receive SLT support via their local primary care service, which tends to be less well-funded. Such inequality in accessing support furthers the argument for needs- based funding allocations rather than those based on medical diagnosis.

Conversely, many of those in receipt of support from an SNA would not be expected to have significant care needs. For example, 51.37% of those with a diagnosis of specific learning difficulties (dyslexia, dyscalculia and/or dyspraxia) were reportedly in receipt of this support. This may be because many children with developmental disabilities have additional needs or it may be, as has been reported elsewhere [57], that SNAs are being deployed for wider duties in Irish schools than is documented. Since this wave of GUI data collection, the Irish government has announced a further increase in the number of SNAs to be employed in Irish schools [58]. We suggest, consistent with Giangreco et al. [59–61] that the impact of such a model of delivery on the inclusion of the child with SEN in school requires further research.

A small proportion of children with PSD were reported to be receiving support in school, yet a large proportion was reported to receive support from a visiting teacher. It may be that parents consider visiting or consultative models of support as qualitatively different to support provided by those who consistently work in school. Occupational therapists (OTs) were not included as an option in the GUI questionnaire in the list of supports received within school, from which parents could choose. Since the collection of the GUI data however, a pilot therapy service to schools that includes OT services has been proposed by the government [62], suggesting an increased awareness of the importance of the role of these professionals in providing SEN support in school.

The same proportion of 13 year olds (roughly a third), regardless of their disability diagnosis, was in receipt of support from the national behaviour programme for emotional/behavioural needs. This is despite the fact that only 1.27% of the sample had reportedly received a formal diagnosis of EBD. This finding suggests that the needs of children with disabilities in second-level education are interpreted as behavioural in nature and managed as such.

On the measure of self-concept used in the study, a disability diagnosis was associated with increased odds of low scores in relation to behaviour, intellectual status and popularity. Consistent with Zeleke [25], a diagnosis of SpLD in this study significantly increased the likelihood of low self-concept, but only in intellectual status. A diagnosis of PSD was associated with low self-concept scores in the social domain of popularity. Consistent with a previous review of the literature, LD was associated with significant odds of low self-concept in intellectual/academic achievement [25]. A diagnosis of SLCN or ASD was associated with increased odds of low self-concept scores in popularity as would be expected given the reported difficulties such children have in maintaining friendships [63]. ASD was more associated than SLCN with greater likelihood of low self-concept scores in this domain. A diagnosis of EBD was associated with significantly increased odds of low self-concept scores related to behaviour, consistent with the findings of Wei and Marder [64]. Given the proportions of children in the sample receiving emotional/behavioural support, other diagnoses were not also associated with low self-concept. This may suggest that children with disabilities perceive their needs differently than do their teachers.

In contrast to much of the literature, we found reduced odds of low self-concept scores in some domains across disability types. A diagnosis of EBD, PSD, and LA significantly reduced the odds of low self-concept scores in the happiness domain. A further positive finding was that there was no association identified between having a diagnosis and self-concept anxiety scores.

Several initiatives in recent years within Irish education may partly account for these findings, such as additional funding allocations for schools serving under-privileged communities, as well as a national programme providing emotional/behavioural interventions.

## Limitations

The GUI dataset was designed for researchers to study the lives of children in Ireland. The strength of such an initiative is the sampling strategy which allows inferences to be made at a population level and the broad range of variables that are included in the dataset. However, such datasets have several well-documented limitations in measurement of prevalence and disability.

The dataset does not include reliable and valid clinical measures related to different disability types, making it impossible to robustly calculate prevalence data. It also contains pre-defined categories of disabilities, suggesting that these diagnoses reflect needs, which are distinct from each other. In reality, such needs are often on a continuum [65, 66], such that analysing the needs of children as categorically different can be problematic. Further, discrepancy criteria that are still used in diagnosing “specific” learning disabilities are not supported by empirical studies, raising questions about the validity of this category [67]. It is also important to note that whilst most children with a disability would fall under the category of having special educational needs, a child can have SEN without a disability diagnosis. In this dataset such children are unaccounted for.

The impact of their disability on the academic and social functioning of the child has not been included in the analysis. If there is a reciprocal relationship between self-concept and performance, the severity of the impact of the child’s disability would be an important factor to include in the analysis. Likewise, the interaction between different combinations of diagnoses, which was not taken into account in this analysis, could have an effect on self-concept.

Specific models of SEN support in school are not detailed in the dataset. Parents may have only considered one model, such as a professional working directly with their child, when responding to questions about support. In reality, many models of support in school are indirect—involving the implementation of strategies in the classroom under the advice of another professional.

A further limitation relates to the validity of the measure of self-concept used in the analysis. Although different domains of self–concept can be measured within the PHCSCS-2, the theoretical underpinnings of the test are based on an assumption that self-concept is a uni-dimensional construct, and that a child’s self-concept is a relatively stable over time [18, 68].

Finally, the authors acknowledge that other factors shown to influence self-concept, such as parental expectation, were not included in the analysis and this may have influenced the results [69].

## Conclusions and recommendations

We have explored a large, nationally-representative dataset to characterise the life circumstances and self-concept of Irish 13 year olds. We used grossing and weighting factors in our analyses so that inferences about 13 year olds with disabilities in Ireland could be made at a population level.

We identified that Irish children with disabilities in this sample had a complex combination of social and educational needs. We also identified a large proportion of children who were not receiving any additional support in school to address their needs. Discrepancies were evident in the nature and amount of support received by the children, not readily accounted for by the nature of their diagnosis.

From parental reports, it appears that the needs of children with speech, language and communication difficulties are disproportionately under-identified compared with other disability groups. Further, this group reportedly received the least amount of SLT support in school than children with other disability types. Given the essential role that language plays in accessing the curriculum [70, 71], and the lifelong implications of having reduced language skills [72, 73], this finding is concerning.

Odds of low self-concept scores varied across disability type as well as self-concept domains, highlighting the importance of conducting more detailed analyses.

Reliable measures of prevalence and regular, detailed, service audits are required to corroborate these findings and to ensure policy and service planning is responsive to the needs of the Irish school-aged population who have disabilities in school.

## Supporting information

S1 File Respondent questionnaires from the Growing Up in Ireland Child Cohort. (PDF)

S2 File Sampling and weighting information related to the Growing Up in Ireland Child Cohort. (PDF)
