# Supplementary material for: The characteristics, life circumstances and self-concept of 13 year olds with and without disabilities in Ireland: A secondary analysis of the Growing Up in Ireland (GUI) study
Source: PLoS One. 2020 Mar 13;15(3):e0229599. doi: 10.1371/journal.pone.0229599 (PMC7069612; doi:10.1371/journal.pone.0229599)
Supplement: S1 File — (PDF) [file pone.0229599.s001.pdf]

QUESTIONNAIRES AND OTHER DOCUMENTS RELATING TO FIELDWORK FOR WAVE 2 OF THE CHILD COHORT (AT 13 YEARS) OF *GROWING UP IN IRELAND*

February 2014

Table of Contents

Section A: Questionnaires Used in the Child Cohort (at 13 years) 1
Primary Caregiver Main Questionnaire 2
Primary Caregiver Sensitive Questionnaire 27
Secondary Caregiver Main Questionnaire 34
Secondary Caregiver Sensitive Questionnaire 42
Primary Caregiver Twin Questionnaire 48
Secondary Caregiver Twin Questionnaire 59
Young Person Main Questionnaire 62
Young Person Sensitive Questionnaire (Male and Female Versions) 74
Young Person Parenting Inventory - Mum, Dad, Mum’s Partner and Dad’s Partner 81

Section B: Introductory letters, infomation leaflet and consent form used in the Child Cohort (at 13 years) 86
Introductory letter to Parents / Guardians 87
Information Sheet for Parents / Guardians 89
Consent Form for Parents / Guardians 94
Consent Form for Young Person Sensitive Questionnaire 96
Information Sheet for Young Person 98
Assent Form for Young Person 101

SECTION A: QUESTIONNAIRES USED IN THE CHILD COHORT (AT 13 YEARS)

PRIMARY CAREGIVER MAIN QUESTIONNAIRE

The Economic and Social Research Institute
Whitaker Square
Sir John Rogerson's Quay
Dublin 2
Ph: 01-863 2000 Fax 01-863 2100

An Roinn Leanaí agus Gnóthaí Óige
Department of Children and Youth Affairs

University of Dublin
Trinity College
College Green
Dublin 2

GROWING UP IN IRELAND – the national longitudinal study of children

STRICTLY CONFIDENTIAL

PRIMARY CAREGIVER QUESTIONNAIRE – 13-year

AREA ☐☐☐☐ **HOUSEHOLD** ☐☐☐

Interviewer Name__________________________ **Interviewer Number** ☐☐☐☐☐

Date ____ ______ ______
Day month year

Almost four years have passed since you and your family were interviewed as part of *Growing Up in Ireland.* At that time we explained that we would like to make a return visit to your home for a follow-up interview to see how your child has changed and grown since our last visit. We are now seeking to interview the parents/guardians of <child>. The whole interview with the parents/guardians and child will take about 1½ - 2 hours to complete [INTERVIEWER: Adjust as appropriate for you in the field]. All the information you and your family provide will be treated in the strictest confidence and will not be released in any way which would allow the information you provide to be identified with you or your family. If however, we are told something which might suggest that a child or other vulnerable person is at risk we may have to act on it.

The Department of Health and Children is funding the study through the Department of Children and Youth Affairs, in association with the Department of Social Protection and the Central Statistics Office. The Department of Education and Science is represented on the Steering Group which oversees the Study. A group of researchers led by the Economic and Social Research Institute (ESRI) and The Children's Research Centre at Trinity College Dublin is carrying out the study

Section A – Household Composition

A1a. [INTERVIEWER: I'd like to begin by speaking to <primary caregiver at time 1>. Is <primary caregiver at time 1> still resident in the household?

Yes .... ☐1 No........................ ☐2 → Go to A7a

A1b. Do you have a spouse/partner who lives here with you in the household?

Yes .... ☐1 No........................ ☐2

A1c. At the time of the last interview in [MM/YYYY] you told us that [number of people resident at time 1] people lived here in the household. I'd like to begin by asking you to check the information we collected the last time we visited.

A2. ***The name, sex, date of birth, and relationship of each person to the <primary respondent at time 1> and <child> will be checked and edited where necessary and their residency in the household at time 2 confirmed.***

No.	First name	Sex	Date of Birth	Age If DOB not available	Still resident?	Relationship of each member to PCG and child.		(E) Show Card A2F						
		M F			Y N	R'SHIP TO: CARD A2E1 Mother	R'SHIP TO: CARD A2E2 Child	Not yet at school	School/Education	At work/Training	Unemployed	Retired	Home Duties	Other
1		☐1 ☐2	__ __ __		☐1 ☐2	////								
2		☐1 ☐2	__ __ __		☐1 ☐2		////							
3		☐1 ☐2	__ __ __		☐1 ☐2			☐1	☐2	☐3	☐4	☐5	☐6	☐7
4		☐1 ☐2	__ __ __		☐1 ☐2			☐1	☐2	☐3	☐4	☐5	☐6	☐7
5		☐1 ☐2	__ __ __		☐1 ☐2			☐1	☐2	☐3	☐4	☐5	☐6	☐7
6		☐1 ☐2	__ __ __		☐1 ☐2			☐1	☐2	☐3	☐4	☐5	☐6	☐7
7		☐1 ☐2	__ __ __		☐1 ☐2			☐1	☐2	☐3	☐4	☐5	☐6	☐7
8		☐1 ☐2	__ __ __		☐1 ☐2			☐1	☐2	☐3	☐4	☐5	☐6	☐7

Interviewer: Primary Caregiver should be on line 1. Child should be on line 2. Secondary Caregiver on line 3 (if relevant).

[BLAISE CONDITION: IF ANY PERSON RESIDENT AT TIME 1 IS NO LONGER RESIDENT IN THE HOUSEHOLD AT TIME 2: ASK QUESTIONS AS1 – AS3 ON THE SENSITIVE QUESTIONNAIRE]

[INTERVIEWER: IF THE RESPONDENT INDICATES THAT A RESIDENT MEMBER OF THE HOUSEHOLD WAS ACCIDENTALLY OMITTED FROM THE HOUSEHOLD GRID AT TIME 1 - ADD THEM TO THE NEW GRID BELOW]

A3. Has anyone else joined the household since we last spoke and is currently living with you?

Yes .... ☐1

No........................ ☐2 Go to A4

No	First Name	Sex	Date of Birth	Age If DOB not available	Relationship of each member to PCG and child		Since when have they been living with you		Resident	Show Card A2F						
		M F			Mother (Card A2E1)	Child (Card A2E2)	Month	Year	Y/N	Not yet at school	School/Education	At work/Training	Unemployed	Retired	Home Duties	Other
21		☐1 ☐2	__ __ __							☐1	☐2	☐3	☐4	☐5	☐6	☐7
22		☐1 ☐2	__ __ __							☐1	☐2	☐3	☐4	☐5	☐6	☐7
23		☐1 ☐2	__ __ __							☐1	☐2	☐3	☐4	☐5	☐6	☐7
24		☐1 ☐2	__ __ __							☐1	☐2	☐3	☐4	☐5	☐6	☐7
25		☐1 ☐2	__ __ __							☐1	☐2	☐3	☐4	☐5	☐6	☐7
		☐1 ☐2	__ __ __							☐1	☐2	☐3	☐4	☐5	☐6	☐7
		☐1 ☐2	__ __ __							☐1	☐2	☐3	☐4	☐5	☐6	☐7
		☐1 ☐2														

[INT: RECORD DETAILS OF NEW PERSONS ON HOUSEHOLD GRID AT A3 ABOVE INCLUDING WHEN THEY STARTED LIVING WITH RESPONDENT]

A4. So that's a total of _____ people who live here in the household at present. Is that correct?

Yes .... ☐1 No....................... ☐2 → [INT: Check Household Grid]

[ASK ONLY IF <TIME 1 PRIMARY CARER> IS STILL RESIDENT IN THE HOUSEHOLD AT TIME 2.

A5. When we last spoke in [MM/YY], we interviewed you as the primary caregiver of <child>. We would like you to complete the primary caregiver questionnaire with us on this occasion as well. Can I just check, are you still the primary caregiver of <child>?

Yes .... ☐1 **Go to A9a** No....................... ☐2

A6a. Why is that? --

IF PRIMARY CAREGIVER FROM TIME 1 HAS A RESIDENT SPOUSE PARTNER [IDENTIFIED AT A2 ABOVE] THEN:

A6b. You mentioned that <spouse/partner> [identified at A2 above] lives here with you as part of the household. This means that we should interview him/her as the primary caregiver of <child> on this occasion. Is that correct?

Yes .... ☐1 No....................... ☐2 **[[BLAISE INSTRUCTION - END OF THE INTERVIEW]**

Go to A9a

IF PRIMARY CAREGIVER AT TIME 1 IS NO LONGER RESIDENT IN THE HOUSEHOLD AT TIME 2 ASK A7a – A9.

A7a. Are you the parent / legal guardian of <child> who usually provides the most care to him/her?

Yes .... ☐1 No .... ☐2 → [INT: Ask to speak to PCG]

A7b. [Card A7b] Can you please tell me which of the following best describes your relationship to <child>?
[Interviewer use codes only]

Biological mother/ father .... ☐1
Adoptive mother/ father .... ☐2
Step-mother / Step-father / Partner of child's parent .... ☐3
Foster mother / father .... ☐4
Grand parent .... ☐5
Aunt/uncle .... ☐6
Other relative/ in law .... ☐7
Unrelated guardian...................................... ☐8

A7c. Do you have a spouse/partner who lives here with you in the household?

Yes .... ☐1 No....................... ☐2

A8a. How many people in total (including yourself and <child>) live here regularly as members of the household? _________ persons

No.	First name/ Initial	Sex (M F)	Date of Birth	Age If DOB not available	Was this Person Resident at time 1? (Y N)	Relationship of each member to mother and child. R'SHIP TO: CARD A2E1 Mother	R'SHIP TO: CARD A2E2 Child	(E) Show Card A2F: Not yet at school	School/Education	At work/Training	Unemployed	Retired	Home Duties	Other
51		☐1 ☐2	__ __ __		☐1 ☐2	////		☐1	☐2	☐3	☐4	☐5	☐6	☐7
52		☐1 ☐2	__ __ __		☐1 ☐2		////	☐1	☐2	☐3	☐4	☐5	☐6	☐7
53		☐1 ☐2	__ __ __		☐1 ☐2			☐1	☐2	☐3	☐4	☐5	☐6	☐7
54		☐1 ☐2	__ __ __		☐1 ☐2			☐1	☐2	☐3	☐4	☐5	☐6	☐7

55		☐1 ☐2	—— —— ——		☐1 ☐2				☐1	☐2	☐3	☐4	☐5	☐6	☐7

A8b. Was that person born into the household or did they join for another reason?

Born into the household ....☐1
Joined for another reason (specify) _____☐2

A8c. Since when has this person being living here in the household? ____ month ____ year

Go to A9a

A9a. Does <child> have any full / half / step / adoptive brother(s) or sister(s) who live outside the household?

Yes ....☐1 No ....☐2

A9b. How many full / half / step / adoptive brother(s) or sister(s) does <child> have who live outside the household? _____ n

A9c. For each full/half/step brother/sister who lives outside the household, can you tell me:

1) their gender
2) their Date of Birth (DOB)
3) their relationship to <child>

	Male	Female	Date of Birth	Relationship to <child>
1.	☐1	☐2	__ __ / __ __ / __ __ __ __	*SHOW CARD A9c*
2.	☐1	☐2	__ __ / __ __ / __ __ __ __	*SHOW CARD A9c*
3.	☐1	☐2	__ __ / __ __ / __ __ __ __	*SHOW CARD A9c*

Now I would like to ask you a few questions regarding the Child's health.

B. CHILD'S HEALTH

B1. [Card B1] In general, how would you describe <child's> health in the past year?

Very healthy, no problems ....☐1
Healthy, but a few minor problems ....☐2
Sometimes quite ill...☐3
Almost always unwell...☐4

B2. Does <child> have any on-going chronic physical or mental health problem, illness or disability?

Yes ....☐1 No ....☐2

B3. What is the nature of this problem, illness or disability? Please describe as fully as possible.
[Int: Please record diagnosis, not symptoms of the problem]

____

____

B4. Has this problem, illness or disability been diagnosed by a medical professional?

Yes..............☐1 No ....☐2

B5. Since when has <child> had this problem, illness or disability? ______(mth) _____(year)

B6. Is <child> hampered in his/her daily activities by this problem, illness or disability?

Yes, severely ....☐1 Yes, to some extent ....☐2 No...........☐3

B7. In the past year has <child> had any periods when there was wheezing with whistling on his/her chest when he/she breathed?

Yes .... ☐1 No........................ ☐2

B8. How many separate episodes/bouts of wheezing with whistling on his/her chest has <child> had in the past 12 months? _______ N

B9. Has <child> been prescribed medication for this condition (including inhaler, antibiotics, nebuliser) over the last 12 months?

Yes .... ☐1 No........................ ☐2

B10a. Has <child> received a course of antibiotics in the past 12 months?

Yes .... ☐1 No........................ ☐2

B10b. In total, how many courses of antibiotics has <child> received in the past 12 months?

______ **N**

B11. Most children have accidents at some time. In the last 12 months has <child> had an accident or injury that required hospital treatment or admission?

Yes .... ☐1 No .... ☐2

B12. How many separate accidents has <child> ever had that required hospital treatment or admission?
_______________ accidents

B13. How many of these accidents involved bone fractures or breaks? __________________

B14. About how many nights has <child> spent in hospital over his/her lifetime? (Exclude at time of birth)
[INTERVIEWER: IF NONE, ENTER '0' – DO NOT LEAVE BLANK] ________nights

B15. In the last 12months how many visits has <child> made to the A&E (Accident and Emergency) department of a hospital? [INTERVIEWER: IF 'NONE' ENTER '0' DO NOT LEAVE BLANK] ________ visits

B16. [Card B16] In the last 12 months, how many times have you seen, or talked on the telephone with any of the following about the <child's> physical, emotional or mental health? [Int. if 'none' write '0' do not leave blank]

	N times	Don't know	Refused
A. A general practitioner (GP)	_______	☐3	☐4
B. A practice nurse	_______	☐3	☐4
C. Another medical doctor e.g. in a hospital	_______	☐3	☐4
D. Other professional, psychologist, psychiatrist, counsellor etc	_______	☐3	☐4
E. A social worker	_______	☐3	☐4

B17. Was there any time during the past 12 months when <child> really needed to consult a GP or specialist but did not?

Yes, there was at least one occasion ...... ☐1 No, there was no such occasion......... ☐2

B18. [Card B18] What was the main reason for not consulting a GP or specialist?

a) You couldn't afford to pay .... ☐1
b) The necessary medical care wasn't available or accessible to you .... ☐2
c) You could not take time off work to visit the doctor with <child> .... ☐3
d) You wanted to wait and see if the problem got better .... ☐4
e) Child refused / fear of doctor .... ☐5
f) Child is still on the waiting list .... ☐6
g) Too far to travel/no means of transport .... ☐7
h) Other (specify) .... ☐8

B19. [Card B19] Which of the following best describes how regularly <child> visits the dentist?

At least once a year .... ☐1
Once every two years .... ☐2

Once every three years .... ☐3
Only when there is a problem .... ☐4
Never/almost never................................. ☐5

B20. Has <child> ever had:

	Yes	No
(a) Any permanent / secondary teeth filled?	☐1	☐2
(b) Any permanent / secondary teeth pulled?	☐1	☐2

B21. Was there any time during the past 12 months when <child> really needed to consult a dentist but did not?

Yes, there was at least one occasion ...... ☐1 No, there was no such occasion......... ☐2

B22. [Card B22] What was the main reason for not consulting the dentist?

a) You couldn't afford to pay .... ☐1
b) The necessary medical care wasn't available or accessible to you .... ☐2
c) You could not take time off work to visit the dentist with <child>........ ☐3
d) You wanted to wait and see if the problem got better .... ☐4
e) Child refused / fear of dentist.. ☐5
f) Child is still on the waiting list .... ☐6
g) Too far to travel/no means of transport .... ☐7
h) Other (specify) .... ☐8

B23. Does <child> usually have breakfast at home before going to school?

Yes ....☐1 No ....☐2

B24. [Card B24] Which of these best describes <child's> weight?
[INT: ASK THE RESPONDENT TO USE THE CODES AS ON THE CARD IF CHILD IS PRESENT AT TIME OF INTERVIEW]

Very underweight .... ☐1
Moderately underweight.................................. ☐2
Slightly underweight....................................... ☐3
About the right weight .... ☐4
Slightly overweight.. ☐5
Moderately overweight.................................... ☐6
Very overweight. .... ☐7
Don't know .... ☐8

B25. [Card B25] How far away is <child's> school from your home (one-way distance)?

Less than ½mile (less than 1km) .... ☐1
½ to less than 1 mile (1 - less than 2km).............. ☐2
1-5 miles (2 - less than 8km)................................ ☐3
More than 5 miles away (8km or more) .... ☐4
Attends boarding school .... ☐5
Not applicable .... ☐7

B26. [Card B26] How does <child> usually go to school?

1. He/she walks... ☐1
2. By public transport .... ☐2
3. School bus/coach....................................... ☐3
4. By car .... ☐4
5. Rides a bicycle .... ☐5
6. Other (please describe) .... ☐6
7. Not applicable .... ☐7

C. RESPONDENT'S HEALTH

Now I'd like to ask you some questions about your own health.

C1. [Card C1] In general, how would you say your current health is?

Excellent.. ☐1
Very Good .... ☐2
Good .... ☐3
Fair .... ☐4
Poor... ☐5

C2. Do you have any on-going chronic physical or mental health problem, illness or disability?

Yes .... ☐1 No .... ☐2

C3. What is the nature of this problem, illness or disability? Please describe as fully as possible.
[Int. please record diagnosis – not symptoms of the problem.]

____

____

C4. Since when have you had this problem, illness or disability? ___________(mth) _____(year)

C5. Are you hampered in your daily activities by this problem, illness or disability?

Yes, severely .... ☐1 Yes, to some extent .... ☐2 No .... ☐3

C6. Do you currently or have you in the past suffered from any chronic illness or disability <u>which made it difficult for you to look after <child>?</u>

Yes, in the past .... ☐1 Yes, currently........ ☐2 No............ ☐3

C7. Thinking about your free-time, in general would you say you are…[INT:READ OUT]

Very physically active..................... ☐1
Fairly physically active .... ☐2
Not very physically active............... ☐3
Not at all physically active.............. ☐4

C8. [Card C8] Do you think that you are:
[INT: ASK THE RESPONDENT TO USE CODES 1-8 AS ON THE CARD IF CHILD IS PRESENT AT TIME OF INTERVIEW]

Very underweight .... ☐1
Moderately underweight.. ☐2
Slightly underweight.. ☐3
About the right weight .... ☐4
Slightly overweight .... ☐5
Moderately overweight.. ☐6
Very overweight. .... ☐7
Don't know .... ☐8

C9. [Card C9] How often do you try to lose weight through dieting? Would you say…[INT:READ OUT]

Very often .... ☐1 Often .... ☐2 Sometimes ..... ☐3 Rarely .... ☐4 Never .... ☐5

C10. Is <child> covered by a medical card?

Yes, full card .... ☐1 Yes, doctor only card........ ☐2 Not covered .... ☐3

C11. Is <child> covered by private medical insurance?

Yes .... ☐1 No.............................. ☐2

C12. Does that insurance include the cost of GP visits?

Yes, in full .... ☐1 Yes, partially.............. ☐2 No .... ☐3

D. CHILD'S EMOTIONAL HEALTH AND WELL-BEING

Now I'd like to ask some questions on the Child's emotional health and well-being.

D1. [Card D1] Looking at Card D1, has <child> experienced any of the following since we last interviewed you when he/ she was nine:

[INT: ASK THE RESPONDENT TO USE CODES A-P AS ON THE CARD IF CHILD IS PRESENT AT TIME OF INTERVIEW]

A. Death of a parent ☐1
B. Death of a close family member (other than a parent) please specify ☐2 ________________
C. Death of close friend ☐3
D. Divorce/separation of parents ☐4
E. Moving house within Ireland ☐5
F. Moving country ☐6
G. Stay in foster home/ residential care ☐7
H. Serious illness/injury ☐8
I. Serious illness/injury of a family member ☐9
J. Drug taking/alcoholism in the immediate family ☐10
K. Mental disorder in immediate family ☐11
L. Your house being broken into ☐12
M. Conflict between parents ☐13
N. Parent in prison ☐14
O. Other disturbing event (please specify) ☐15 ________________
P. None of the above ☐16

D2. [Card D2] Listed on Card D2, is a set of statements which could be used to describe <child's> behaviour. For each item, please indicate whether it is Not True, Somewhat True or Certainly True. It would help us if you answered all items as best you can even if you are not absolutely certain. Please give answers on the basis of <child's> behaviour over the last six months. Use answers 1, 2 or 3 as on the card if you like.

	Not True	Somewhat True	Certainly True
A. Considerate of other people's feelings	☐1	☐2	☐3
B. Restless, overactive, cannot stay still for long	☐1	☐2	☐3
C. Often complains of headaches, stomach aches or sickness	☐1	☐2	☐3
D. Shares readily with other children (treats, toys, pencils etc.)	☐1	☐2	☐3
E. Often has temper tantrums or hot tempers	☐1	☐2	☐3
F. Rather solitary, tends to play alone	☐1	☐2	☐3
G. Generally obedient, usually does what adults request	☐1	☐2	☐3
H. Many worries, often seems worried	☐1	☐2	☐3
I. Helpful if someone is hurt, upset or feeling ill	☐1	☐2	☐3
J. Constantly fidgeting or squirming	☐1	☐2	☐3
K. Has at least one good friend	☐1	☐2	☐3
L. Often fights with other children or bullies them	☐1	☐2	☐3
M. Often unhappy, down-hearted or tearful	☐1	☐2	☐3
N. Generally liked by other children	☐1	☐2	☐3
O. Easily distracted, concentration wanders	☐1	☐2	☐3
P. Nervous or clingy in new situations, easily loses confidence	☐1	☐2	☐3
Q. Kind to younger children	☐1	☐2	☐3
R. Often lies or cheats	☐1	☐2	☐3
S. Picked on or bullied by other children	☐1	☐2	☐3
T. Often volunteers to help others (parents, teachers, other children)	☐1	☐2	☐3
U. Thinks things out before acting	☐1	☐2	☐3
V. Steals from home, school or elsewhere	☐1	☐2	☐3
W. Gets on better with adults than with other children	☐1	☐2	☐3
X. Many fears, easily scared	☐1	☐2	☐3
Y. Sees tasks through to the end, good attention span	☐1	☐2	☐3

D3. [Card D3] Listed on card D3 are a number of personality traits that may or may not apply to your child. Please indicate the extent to which you agree or disagree with that statement. You should rate the extent to which the pair of traits applies to him/her, even if one characteristic applies more strongly than the other.

I see my child as:

	Disagree strongly	Disagree moderately	Disagree a little	Neither agree nor disagree	Agree a little	Agree moderately	Agree strongly
Extroverted, enthusiastic	☐1	☐2	☐3	☐4	☐5	☐6	☐7
Critical, quarrelsome	☐1	☐2	☐3	☐4	☐5	☐6	☐7
Dependable, self-disciplined	☐1	☐2	☐3	☐4	☐5	☐6	☐7
Anxious, easily upset	☐1	☐2	☐3	☐4	☐5	☐6	☐7
Open to new experiences, complex	☐1	☐2	☐3	☐4	☐5	☐6	☐7
Reserved, quiet	☐1	☐2	☐3	☐4	☐5	☐6	☐7
Sympathetic, warm	☐1	☐2	☐3	☐4	☐5	☐6	☐7
Disorganized, careless	☐1	☐2	☐3	☐4	☐5	☐6	☐7
Calm, emotionally stable	☐1	☐2	☐3	☐4	☐5	☐6	☐7
Conventional, uncreative	☐1	☐2	☐3	☐4	☐5	☐6	☐7

Now I'd like to ask you some questions about the Child's education

E. CHILD'S EDUCATION – PAST AND CURRENT

E1a. What class did / will <child> start in September 2011?

- 5th Class ☐1 Go to E1b
- 6th Class ☐2 Go to E1b
- First Year ☐3 Go to E1b
- Second Year ☐4 Go to E1b
- Child is being home schooled. ☐5 Go to E7
- Child attends a special school ☐6 Go to E1b
- Child no longer attends school ☐7 Go to E10

E1b. What school does <child> attend / will attend from September 2011?

Name of school: ________________________________

Full address of school: ____

____

E1c. In what year did <child> start primary school? September 20_ _

E1d. [Card E1d] How would you describe <child's> current base class – the one they will be in from September 2011? (Tick one box)

- Special class ☐1
- Class which is mixed ability / randomly allocated ☐2
- Higher stream class in streamed school ☐3
- Middle stream class in streamed school ☐4
- Lower stream class in streamed school ☐5
- Not sure / don't know ☐6

[ONLY ASK IF CHILD IS IN 2nd YEAR AT E1a, THEN GO TO E5]

E2. [Card E2] Here are some views about how your child settled into their new school. There are no right or wrong answers. For each statement please tick ONE BOX ONLY to show whether you agree or disagree with these views.

	**Strongly agree**	**Agree**	**Neither agree nor disagree**	**Disagree**	**Strongly disagree**
My child settled well into secondary school.	☐1	☐2	☐3	☐4	☐5
My child missed old friends from primary school.	☐1	☐2	☐3	☐4	☐5
My child was anxious about making new friends.	☐1	☐2	☐3	☐4	☐5
My child coped well with the school work.	☐1	☐2	☐3	☐4	☐5
My child made new friends	☐1	☐2	☐3	☐4	☐5
My child is involved in extra-curricular activities.	☐1	☐2	☐3	☐4	☐5
My child gets too much homework at this school.	☐1	☐2	☐3	☐4	☐5

[ONLY ASK IF CHILD IS IN 1st YEAR AT E1a, THEN GO TO E4b]

E3. [Card E3] Here are some views about how your child is settling into their new school. There are no right or wrong answers. For each statement please tick ONE BOX ONLY to show whether you agree or disagree with these views.

	Strongly agree	Agree	Neither agree nor disagree	Disagree	Strongly disagree
My child is settling in well into secondary school	☐1	☐2	☐3	☐4	☐5
My child misses old friends from primary school.	☐1	☐2	☐3	☐4	☐5
My child is anxious about making new friends	☐1	☐2	☐3	☐4	☐5
My child is coping well with the school work.	☐1	☐2	☐3	☐4	☐5
My child has made new friends	☐1	☐2	☐3	☐4	☐5
My child is involved in extra-curricular activities.	☐1	☐2	☐3	☐4	☐5
My child gets too much homework at this school.	☐1	☐2	☐3	☐4	☐5

[ONLY ASK IF CHILD IS IN 5th / 6th CLASS AT E1a, THEN GO TO E5]

E4a. [Card E4a] If your child is still in fifth / sixth class for each statement please tick ONE BOX ONLY to show whether you agree or disagree with these views.

	Strongly agree	Agree	Neither agree nor disagree	Disagree	Strongly disagree
My child is excited about starting secondary school	☐1	☐2	☐3	☐4	☐5
My child is looking forward to making new friends	☐1	☐2	☐3	☐4	☐5
My child is nervous about moving to a new school	☐1	☐2	☐3	☐4	☐5

E4b. Has <child> attended an Open Day at his/her new school Yes.......... ☐1 No ..... ☐2

E5. [Card E5] Over the last 12 months, have you had any contact with the school? (Please include contact you have had with the child's current school or any other school the child attended in the last 12 months) [Please tick 'Yes' or 'No' to each.]

	Yes	No
A. You have attended a parent-teacher meeting	☐1	☐2
B. You have attended a school concert, play or other event (such as sports day)	☐1	☐2
C. You have <u>been to see</u> the principal or another teacher about child's behaviour or school performance	☐1	☐2
D. You have spoken to the principal or another teacher <u>on the phone</u> about child's behaviour or school performance	☐1	☐2

E6a. [Card E6a] Looking at Card E6a, during the last 12 months, about how many days was <child> absent from school for any reason? (Only include days the child was absent when the school was open e.g. do not include days missed because of the school being closed due to bad weather).

0 days	☐1	11 to 20 days	☐5
1 - 3 days	☐2	More than 20 days	☐6
4 to 6 days	☐3	Not in school last year	☐7
7 to 10 days	☐4		

E6b. [Card E6b] Looking at Card E6b, what was the main reason for <child> being absent from school?

Health reasons (illness or injuries)	☐1	A problem with a teacher	☐8
Problems with transportation	☐2	A problem with children at school	☐9
Problems with the weather	☐3	Difficulties with childcare arrangements	☐10
A family vacation	☐4	Family crisis	☐11
Refused to go to school	☐5	Child has left school	☐12
A fear of school (school phobia)	☐6	Other (specify) ____________________	☐13
Suspended from school	☐7		

E7. [Card E7] Looking at Card E7, how much time does <child> usually spend doing homework on a weekday during term time?

0 to 30 minutes	☐1	2 to less than 3 hours	☐5
31 minutes to less than one hour	☐2	3 to less than 4 hours	☐6
1 to less than 1.5 hours	☐3	4 hours or more	☐7
1.5 to less than 2 hours	☐4	Doesn't get homework	☐8 Go to E9

E8a. How often do you or your spouse/partner provide help with <child>'s homework? Would you say…[INT: READ OUT]

Always/ Nearly Always	Regularly	Now and Again	Rarely	Never	Never gets homework
☐1	☐2	☐3	☐4	☐5	☐6

E8b. Why is that?

Child doesn't need help	I / We don't have time	I / We are not able to help	Child doesn't want help	Someone else helps
☐1	☐2	☐3	☐4	☐5

E9. [Card E9] Looking at Card E9, taking everything into account, how far do you expect <child> will go in his/her education or training?

- Junior Certificate or equivalent ☐1
- Leaving Certificate or equivalent ☐2
- An apprenticeship or trade ☐3
- Diploma/Certificate ☐4
- Degree ☐5
- Postgraduate/higher degree ☐6
- Don't know ☐7

E10. About how many close friends does <child> have?

None....... ☐1 1 .... ☐2 2 or 3.......... ☐3 4 or 5 .... ☐4 6 or more ...... ☐5

E11. To your knowledge, has <child> been a victim of bullying in the last 3 months?

Yes.............. ☐1 No .... ☐2

E12. [Card E12] Looking at Card E12, what form did the bullying take? [Int. tick all that apply]

- A. Physical bullying ☐1
- B. Verbal bullying (name calling, hurtful slagging). ☐2
- C. Electronic (phone messaging, emails, Facebook, etc) ☐3
- D. Graffiti/pinning up notes/passing notes in class ☐4
- E. Taking /damaging personal possessions ☐5
- F. Sexual comments ☐6
- G. Exclusion (being left out). ☐7
- H. Gossip, spreading rumours ☐8
- I. Threatened or forced to do things s/he didn't want to ☐9
- J. Other (specify)________________ ☐10

E13. [Card E13] How often did the bullying take place?

- A. Once or twice ☐1
- B. 2 or 3 times a month ☐2
- C. About once a week ☐3
- D. Several times a week ☐4

E14. Did this upset your child?

- A. A lot ☐1
- B. A little ☐2
- C. Not at all ☐3

E15. [Card E15] Does <child> have any of the following conditions or disabilities? [Tick all that apply]

- a. Physical disability or visual or hearing impairment ☐1
- b. Specific learning disability (e.g. Dyslexia, Dyscalculia, Dyspraxia) ☐2
- c. General learning disabilities (Mild, Moderate, Severe/Profound) ☐3
- d. Autism Spectrum Disorders (e.g. Austism, Aspergers syndrome) ☐4
- e. Emotional or behavioural disorders (e.g. ADHD (Attention Deficit Hyperactivity Disorder)/ ADD) ☐5
- f. Mental health difficulty ☐6
- g. Speech or language difficulty (including speech impediment) ☐7
- h. Assessed Syndrome (e.g. Down Syndrome, Tourettes Syndrome) ☐8
- i. Slow progress (reasons unclear) ☐9
- j. Other (please specify) ☐10
- k. None of the above ☐11 → **Go to E24**

E16. Has this condition or disability been diagnosed by a medical professional?

Yes .... ☐1 No .... ☐2 Awaiting Consultation .... ☐3

E17. What age was <child> when this condition or disability was first diagnosed? ______________ years

[INT: If condition or disability was diagnosed at time of birth, code as '0']

Ask E18 only of respondents who ticked yes at E15e

E18. Has <child> been prescribed any medication for this condition (e.g. Ritalin, Abilify etc…)?

Yes .... ☐1 No .... ☐2

Ask E19 only of respondents who ticked yes at E15f

E19. Has <child> been prescribed any medication for this condition?

Yes .... ☐1 No .... ☐2

Ask E20 only of respondents who ticked yes at E15G

E20. [Card E20] In which areas does <child> have difficulties? What speech problems does <child> have?
[TICK ALL THAT APPLY]

A. Reluctant to speak .... ☐1
B. Speech not clear to the family .... ☐2
C. Speech not clear to others .... ☐3
D. Speech is developing slowly .... ☐4
E. Difficulty finding words .... ☐5
F. Difficulty putting words together .... ☐6
G. Voice sounds unusual .... ☐7
H. Stutters, stammers .... ☐8
I. Lisp or difficulty pronouncing certain letter combinations .... ☐9
J. Other (please specify) .... ☐10
K. Don't know .... ☐99

E21. [Card E21] Please indicate if <child> receives support from any of the following IN SCHOOL
[Tick all that apply]
In School

Resource Teaching/ Learning Support .... ☐1
Special Needs Assistant .... ☐2
Technical Assistance .... ☐3
Visiting Teacher .... ☐4
Transport Service .... ☐5
Speech and Language Therapist .... ☐6
Behavioural Management Programme .... ☐7
School psychologist .... ☐8
National Educational Psychological Service .... ☐9
Other (please specify)……………… .... ☐10
Doesn't receive any supports .... ☐11

E22. [Card E22] Please indicate if <child> receives support from any of the following OUTSIDE SCHOOL
[Tick all that apply]
Outside School

Speech and Language Therapist .... ☐1
Occupational Therapist .... ☐2
Physiotherapist .... ☐3
Psychologist .... ☐4
Psychiatrist .... ☐5
Extra tuition/private tuition .... ☐6
Other (please specify) __________________ ..... ☐7
Doesn't receive any supports .... ☐8

E23. In general, how adequate are the supports <child> receives for this/these condition(s) or disability(ies)

Barely adequate .... ☐1
Adequate .... ☐2
Excellent .... ☐3
Doesn't receive any supports .... ☐4

E24. How many books does <child> have access to in the home? Would you say…[INT: READ OUT]

None .... ☐1
1 to 10 .... ☐2
11 to 30 .... ☐3
31 to 50 .... ☐4
51 to 100 .... ☐5
More than 100 .... ☐6

E25a. Do you have a computer at home? Yes.......... ☐1 No .... ☐2

E25b. Does <child> have access to the internet? Yes ☐1 No .... ☐2

E25c. Do you have an internet filter system (e.g. Net Nanny) which controls <child's> access to the internet?

Yes .... ☐1 No .... ☐2

E26. [Card E26] On a normal weekday, during term-time, about how much time does <child> spend using the computer. Please include time before school as well as time after school. DO NOT include time spent using computers in school.

None... ☐1
Less than an hour .... ☐2
1 hour to less than 3 hours .... ☐3
3 hours to less than 5 hours.................. ☐4
5 hours to less than 7 hours.................. ☐5
7 hours or more.................................... ☐6

E27. [Card E27] On a typical weekday, who, if anyone, minds <child> between the time they finish school and 6pm in the evening? (Tick one only; if more than one indicate the type of care where <child> spends MOST time or is the most frequently used)

They come home and take care of themselves .... ☐1
Minded at home by an older sibling.. ☐2
Minded at home by you or your spouse/partner...................... ☐3
Minded at home by a relative .... ☐4
Minded at home by another adult (not a relative).................... ☐5
Attend an after-school program/club .... ☐6
Hang out with friends... ☐7
Other (please specify) .... ☐8

F: FAMILY CONTEXT

Now some questions about your relationship with <Child>.

F1. [Show Card F1] Looking at Card F1, I am going to read out some statements about the relationship between you and your child. Please listen to each statement and describe the degree to which each of the following statements currently applies.

	Definitely does not apply	Not really	Neutral, not sure	Applies somewhat	Definitely applies
A. I share an affectionate, warm relationship with my child.	☐1	☐2	☐3	☐4	☐5
B. My child and I always seem to be struggling with each other.	☐1	☐2	☐3	☐4	☐5
C. If upset, my child will seek comfort from me.	☐1	☐2	☐3	☐4	☐5
D. My child is uncomfortable with physical affection or touch from me.	☐1	☐2	☐3	☐4	☐5
E. My child values his/her relationship with me.	☐1	☐2	☐3	☐4	☐5
F. When I praise my child, he/she beams with pride.	☐1	☐2	☐3	☐4	☐5
G. My child spontaneously shares information about himself/herself	☐1	☐2	☐3	☐4	☐5
H. My child easily becomes angry at me.	☐1	☐2	☐3	☐4	☐5
I. It is easy to be in tune with what my child is feeling.	☐1	☐2	☐3	☐4	☐5
J. My child remains angry or is resistant after being disciplined	☐1	☐2	☐3	☐4	☐5
K. Dealing with my child drains my energy.	☐1	☐2	☐3	☐4	☐5
L. When my child is in a bad mood, I know we're in for a long and difficult day.	☐1	☐2	☐3	☐4	☐5
M. My child's feelings toward me can be unpredictable or can change suddenly.	☐1	☐2	☐3	☐4	☐5
N. My child is sneaky or manipulative with me.	☐1	☐2	☐3	☐4	☐5
O. My child openly shares his/her feelings and experiences with me.	☐1	☐2	☐3	☐4	☐5

F2. [Card F2] The following are some questions on your knowledge of what <child> does in his/her free time, where he/she goes, and who he/she has as friends.

	Almost never or never	Not very often	Sometimes	Often	Almost always or always	N/A
A. Do you know what <child> does with his/her free time.	☐1	☐2	☐3	☐4	☐5	☐6
B. Do you know who he/she has as friends during his/her free time.	☐1	☐2	☐3	☐4	☐5	☐6
C. Do you usually know what type of homework he/she has.	☐1	☐2	☐3	☐4	☐5	☐6
D. Do you know what he/she spends his/her money on	☐1	☐2	☐3	☐4	☐5	☐6
E. Do you know when he/she has a test or homework due at school.	☐1	☐2	☐3	☐4	☐5	☐6
F. Do you know how he/she does in different subjects at school.	☐1	☐2	☐3	☐4	☐5	☐6
G. Do you know where he/she goes when out at night with friends	☐1	☐2	☐3	☐4	☐5	☐6
H. Do you know where he/she goes and what he/she does after school.	☐1	☐2	☐3	☐4	☐5	☐6
I. How often in the last month have you had no idea where he/she was.	☐1	☐2	☐3	☐4	☐5	☐6

F3. [CARD F3] The following are some questions about how much <child> actually tells you about what he/she is doing, without being asked.

	Almost never or never	Not very often	Sometimes	Often	Almost always or always	N/A
A. Does he/she spontaneously tell you about his/her friends.	☐1	☐2	☐3	☐4	☐5	☐6
B. Does he/she want to tell you about school (how subjects are going; relationships with teachers etc).	☐1	☐2	☐3	☐4	☐5	☐6
C. Does he/she keep a lot of secrets from you about what he/she is doing in his/her spare time	☐1	☐2	☐3	☐4	☐5	☐6
D. Does he/she hide a lot from you about what he/she is doing during nights and weekends	☐1	☐2	☐3	☐4	☐5	☐6
E. Does he/she like to tell you what he/she has been doing and where he/she went when out for the evening..	☐1	☐2	☐3	☐4	☐5	☐6

F4. [Show Card F4] Looking at Card F4, now I'd like to ask you about the time <child> spends with you including times when others are present.   How many days per week do you:

	Every day / 7 days per week	3 to 6 days per week	1 to 2 days per week	1 to 2 times per month	Rarely or never
A. Sit down to eat together	☐1	☐2	☐3	☐4	☐5
B. Play sports, cards or games together	☐1	☐2	☐3	☐4	☐5
C. Talk about things together	☐1	☐2	☐3	☐4	☐5
D. Do household activities together (e.g. gardening, cooking, cleaning, etc)	☐1	☐2	☐3	☐4	☐5
E. Go on an outing together (e.g. going to the cinema, theatre, walking, shopping)	☐1	☐2	☐3	☐4	☐5

F5. [Show Card F5] Looking at Card F5, how often does <child> get together with, see or spend time with the following people (excluding those living in your home)

	Quite a lot	Now and again	Rarely	Live Abroad	Doesn't have
A. Grandparents	☐1	☐2	☐3	☐4	☐5
B. Uncles/Aunts	☐1	☐2	☐3	☐4	☐5
C. Cousins	☐1	☐2	☐3	☐4	☐5
D. Other family members/ close family friends	☐1	☐2	☐3	☐4	☐5

F6. [Show Card F6] Please tell me how strongly you agree or disagree with the following statements.

	Strongly Disagree	Disagree	Neither Agree nor disagree	Agree	Strongly Agree	NA
**Because of your work responsibilities:**						
A. You have missed out on home or family activities that you would have liked to have taken part in	☐1	☐2	☐3	☐4	☐5	☐6
B. Your family time is less enjoyable and more pressured	☐1	☐2	☐3	☐4	☐5	☐6
**Because of your family responsibilities:**						
C. You have to turn down work activities or opportunities you would prefer to take on	☐1	☐2	☐3	☐4	☐5	☐6
D. The time you spend working is less enjoyable and more pressured	☐1	☐2	☐3	☐4	☐5	☐6

F7. How fairly or unfairly would you say the household tasks are distributed between you and your partner? Would you say…[INT: READ OUT]

Very unfairly .... ☐1 Quite unfairly .... ☐2 Fairly .... ☐3 Don't have partner. .... ☐4

F8. [Show Card F8] I would now like to ask some questions about <child's> behaviour over the last 12 months. Please tell me the extent to which the following statements apply:

	Not at all	Once	2-5 times	6 or more times
A. Often started fights or bullies, threatens or intimidates others	☐1	☐2	☐3	☐4
B. Has used a weapon that could cause serious physical harm to others (eg, a bat, brick, broken bottle, knife)	☐1	☐2	☐3	☐4
C. Has been physically cruel to other people	☐1	☐2	☐3	☐4
D. Has been physically cruel to animals	☐1	☐2	☐3	☐4
E. Deliberately destroyed or damaged property	☐1	☐2	☐3	☐4
F. Has broken into someone else's house, building or car	☐1	☐2	☐3	☐4
G. Has lied to obtain goods or favours (i.e., 'cons' others)	☐1	☐2	☐3	☐4
H. Has stolen items of value without confronting a victim (e.g., shoplifting, but without breaking and entering)	☐1	☐2	☐3	☐4
I. Has stayed out at night despite parental prohibitions	☐1	☐2	☐3	☐4
J. Has run away from home overnight at least twice while living in parental home (or once for a lengthy period)	☐1	☐2	☐3	☐4
K. Has truanted from school	☐1	☐2	☐3	☐4

F9. [Card F9] For the following items could you indicate whether or not the child / children in the family has the item and, if not, if it is because you couldn't afford it or for another reason?

	Yes	No, Cannot Afford	No, other reason
a. Does the child / children have some new (not second hand) clothes?	☐1	☐2	☐3
b. Does the child / children have two pairs of properly fitting shoes, including a pair of all-weather shoes?	☐1	☐2	☐3
c. Does the child / children eat fresh fruit and/or vegetables at least once a day?	☐1	☐2	☐3
d. Does the child / children eat three meals a day?	☐1	☐2	☐3
e. Does the child / children eat a meal with meat, chicken or fish (or vegetarian equivalent) at least once a day?	☐1	☐2	☐3
f. Does the child / children have books at home suitable for his/her age	☐1	☐2	☐3
g. Does the child / children have outdoor leisure equipment (bicycle, roller skates, etc.)?	☐1	☐2	☐3
h. Does the child / children have indoor games (board games, computer games etc)?	☐1	☐2	☐3
i. Does the child / children participate in a regular leisure activity (swimming, playing an instrument, youth organisations, etc.)?	☐1	☐2	☐3
j. Does the child / children have celebrations on special occasions (birthdays, religious events)?	☐1	☐2	☐3
k. Does the child / children invite/have friends to your house to play and/or eat from time to time?	☐1	☐2	☐3
l. Does the child / children participate in school trips and school events that cost money?	☐1	☐2	☐3
m. Does the child / children have a suitable place to study or do homework?	☐1	☐2	☐3
n. Does the child / children have outdoor space in the neighbourhood to play safely (including gardens)	☐1	☐2	☐3

G: SOCIO-DEMOGRAPHICS

Now some questions about the circumstances of your household.

G1. Does your accommodation have access to a garden or common space (either private or shared)?

Yes .... ☐1   No .... ☐2

G2. [Card G2] From this card, please tell me which best describes your (and your partner's) occupancy of the accommodation?

1. Owner occupied (with or without a mortgage) .... ☐1
2. Being purchased from a Local Authority under a Tenant Purchase Scheme .... ☐2
3. Rented from a Local Authority .... ☐3
4. Rented from a Voluntary Body.. ☐4
5. Rented from a Private Landlord .... ☐5
6. Living with and paying rent to your (or your partner's) parent(s) .... ☐6
7. Occupied free of rent with your (or your partner's) parent(s) .... ☐7
8. Occupied free of rent from your (or your partner's) job .... ☐8

G3. [Card G3] Which of these descriptions BEST describes your usual situation in regard to work?
[Int: If respondent is on maternity leave and she has a job which she intends to return to, she should be coded as 'O']

0. Currently on maternity leave, but with a job to return to .... ☐0
1. Employee (incl. apprenticeship or Community Employment) .... ☐1
2. Self employed outside farming......................... ☐2
3. Farmer.. ☐3
4. Student full-time.. ☐4
5. On State training scheme (FAS, Failte Ireland etc)..... ☐5
6. Unemployed, actively looking for a job .... ☐6
7. Long-term sickness or disability......................... ☐7
8. Home duties / looking after home or family .... ☐8
9. Retired .... ☐9
10. Other (please specify) ________________________ ☐10

[BLAISE CONDITION: IF RESPONDENT NOT WORKING AT TIME 1 BUT IS WORKING AT TIME 2 ASK G4]

G4. When did you return to work? ________ **year**

G5. How many hours do you normally work per week, including any regular overtime work? If you work at more than one job, please include the hours in all jobs. _____________ hours

G6. On a typical work day, how much time in minutes do you spend commuting to and from work (outward and return journey combined)?

_________ **minutes**   [Int. if respondent works at home enter '0' for minutes]

G7. [Card G7] What is your occupation in your main job?

In all cases please describe the occupation fully and precisely giving the full job title.

Use precise terms such as:	Do not use general terms such as:
RETAIL STORE MANAGER	MANAGER
SECONDARY TEACHER	TEACHER
ELECTRICAL ENGINEER	ENGINEER

Civil servants and local government employees should state their grade e.g. SENIOR ADMINISTRATIVE OFFICER.
Members of the Gardai or Army should state their rank. Teachers should state the branch of teaching e.g. PRIMARY TEACHER.
Clergy and religious orders should give full description e.g. NUN, REGISTERED GENERAL NURSE.

Write in your main OCCUPATION

____

G8. Do you supervise or manage any personnel in your job?

Yes .... ☐1   No .... ☐2

G9. How many? ___________________

G10. How many employees (if any) do you have?_________ **employees**   N A …. ☐99

G11. [Ask only if Farmer at G3.] How many acres do you farm? ______________ acres

Go to G23

G12. Apart from holiday or casual work, have you ever had a full-time job? Yes .. ☐1 No .. ☐2 **Go to G19**

G13. In what year did you last work in that full-time job? _______ **year**

G14. When you last worked in that full-time job were you?

Employee (incl. apprenticeship or Community Employment) .... ☐1 Self-employed outside farming ..... ☐2 Farmer .... ☐3

G15. [Card G15] What (was) your occupation in your main job?

In all cases describe the occupation fully and precisely giving the full job title.

Use precise terms such as:	Do not use general terms such as:
RETAIL STORE MANAGER	MANAGER
SECONDARY TEACHER	TEACHER
ELECTRICAL ENGINEER	ENGINEER

Civil servants and local government employees should state their grade e.g. SENIOR ADMINISTRATIVE OFFICER.
Members of the Gardai or Army should state their rank. Teachers should state the branch of teaching e.g. PRIMARY TEACHER.
Clergy and religious orders should give full description e.g. NUN, REGISTERED GENERAL NURSE.

Write in your main OCCUPATION

____

G16a. Did you supervise or manage any personnel in your job?

Yes .... ☐1 No .... ☐2

G16b. How many? __________________

G17. How many employees (if any) did you have?_________ **employees** N A .... ☐99

G18. [Ask only if Farmer at G14] How many acres do you farm? ______________ acres

G19. Do you currently have a part time job outside the home? Yes .... ☐1 No....... ☐2 **Go to G22**

G20. On average, how many hours per week do you work in that part-time job? ___________ **hours**

G21. [Card G21] What is your occupation in that job?

In all cases describe the occupation fully and precisely giving the full job title.

Use precise terms such as:	Do not use general terms such as:
RETAIL STORE MANAGER	MANAGER
SECONDARY TEACHER	TEACHER
ELECTRICAL ENGINEER	ENGINEER

Civil servants and local government employees should state their grade e.g. SENIOR ADMINISTRATIVE OFFICER.
Members of the Gardai or Army should state their rank. Teachers should state the branch of teaching e.g. PRIMARY TEACHER.
Clergy and religious orders should give full description e.g. NUN, REGISTERED GENERAL NURSE.

Write in your main OCCUPATION

____

If a farmer or a farm worker, write in the SIZE of the farm ________**acres**

Go to G23

G22. [**Card G22] From the reasons listed on this card could you tell me the most important reasons for you not working in a paid job outside the home? If more than one reason, please rank them in order of importance, where 1 is the most important reason, up to a maximum of 3.**

A. I can't find a job .... ___
B. I chose not to work .... ___
C. I am caring for an elderly or ill relative or friend .. ___
D. I prefer be at home to look after my children myself
E. I cannot earn enough to pay for childcare .... ___
F. I cannot find suitable childcare...................... ___
G. There are no suitable jobs available for me .. ___
H. My family would lose Social Welfare or medical benefits if I was earning .... ___
I. Other reason (specify)___________________ ___

Go to G23

G23. [Card G23] What is the occupation of your spouse / partner? [If not currently employed, please record last occupation]

In all cases describe the occupation fully and precisely giving the full job title.

Use precise terms such as:	Do not use general terms such as:
RETAIL STORE MANAGER	MANAGER
SECONDARY TEACHER	TEACHER
ELECTRICAL ENGINEER	ENGINEER

Civil servants and local government employees should state their grade e.g. SENIOR ADMINISTRATIVE OFFICER.
Members of the Gardai or Army should state their rank. Teachers should state the branch of teaching e.g. PRIMARY TEACHER.
Clergy and religious orders should give full description e.g. NUN, REGISTERED GENERAL NURSE.

Write in main OCCUPATION [If a farmer or a farm worker, how many acres do you farm? ________ acres]

____

HOUSEHOLD INCOME

Now I would like you ask you a few questions about household income. Once again I would like to assure you that all information will be treated in the strictest confidence.

G24. [Card G24] Looking at Card G24, which of the following sources of income does the HOUSEHOLD receive? Please consider the income of *ALL* household members, not just your own, your spouse/partner's income. *[INT. Tick 'Yes' or 'No' for each in Col. A]*

G25. [Card G24] And of these sources of income which is the largest source of income at present?*[Int Tick one box only in Col. B]*

	A Receive? Yes	A Receive? No	B Largest Source
A. Wages or Salaries	☐1	☐2	☐3
B. Income from Self-Employment	☐1	☐2	☐3
C. Income from Farming	☐1	☐2	☐3
D. Children's Allowance/ Child Benefit	☐1	☐2	☐3
E. Other Social Welfare Payments	☐1	☐2	☐3
F. Other Income (incl. income from maintenance payments, investments, savings, dividends, private pensions, property)	☐1	☐2	☐3

HOUSEHOLD INCOME FROM ALL HOUSEHOLD MEMBERS

G26. [Card G26] If you added up all the income sources from ALL household members what would be the total HOUSEHOLD NET income, i.e. after deductions for tax and PRSI as well as the income levy and public sector pension levy [if applicable]? Include income from all sources and from all household members. *[INT: IF RESPONDENT CANNOT GIVE EXACT FIGURE GO TO G27.IF EXACT FIGURE GIVEN GO TO G29]*

Don't know........☐99 €__________________ per Week.........☐1 Month.........☐2 Year ☐3

G27. [Card G27] I know that it is difficult to give an exact figure for household income but on Card G27 we have a scale of incomes, and we would like to know into which group your total HOUSEHOLD NET income falls, i.e. after deductions for tax and PRSI as well as the income levy and public sector pension levy [if applicable]? Include income from all sources and from all members of the household. Looking at the card could you tell me the letter of the group your household falls into, after deductions for tax and PRSI. [Int: Tick the letter of the group your household falls into, after deductions for tax and PRSI only]

***HOUSEHOLD NET INCOME* AFTER DEDUCTIONS OF TAX AND PRSI**

Per Week	*Per Month*	*Per Year*	*Category*
Under €230	Under €1,000	Under €12,000	A☐1 → Section A, Card G28
€231 to under €350	€1,001 to under €1,500	€12,001 to under €18,000	B☐2 → Section B, Card G28
€351 to under €460	€1,501 to under €2,000	€18,001 to under €24,000	C☐3 → Section C, Card G28
€461 to under €575	€2,001 to under €2,500	€24,001 to under €30,000	D☐4 → Section D, Card G28
€576 to under €800	€2,501 to under €3,500	€30,001 to under €42,000	E☐5 → Section E, Card G28
€801 to under €925	€3,501 to under €4,000	€42,001 to under €48,000	F☐6 → Section F, Card G28
€926 to under €1,150	€4,001 to under €5,000	€48,001 to under €60,000	G☐7 → Section G, Card G28
€1,151 to under €1,500	€5,001 to under €6,500	€60,001 to under €78,000	H☐8 → Section H, Card G28
€1,501 to under €1,850	€6,501 to under €8,000	€78,001 to under €96,000	I☐9 → Section I, Card G28
€1,851 or more	€8,001 or more	€96,001 or more	J☐10 → Section J, Card G28

Refused.............................☐77 **GO TO G29** Don't Know...☐88 **GO TO G29**

G28. [Card G28] Would that be [Int: Show Card G28 and tick 1, 2 or 3 in appropriate section under per wk; per mth or per yr]

**A**	**Per week**	under €75 ☐1	€75 to €150 ☐2	€151 to €230 ☐3
	**Per Month**	€0 to €300 ☐1	€301 to €650 ☐2	€651 to €1,000 ☐3
	**Per Year**	€0 to €4,000 ☐1	€4,001 to €8,000 ☐2	€8,001 to €12,000 ☐3
**B**	**Per week**	€231 to €270 ☐1	€271 to €310 ☐2	€311 to €350 ☐3
	**Per Month**	€1,001 to €1,150 ☐1	€1,151 to €1,350 ☐2	€1,351 to €1,500 ☐3
	**Per Year**	€12,001 to €14,000 ☐1	€14,001 to €16,000 ☐2	€16,001 to €18,000 ☐3
**C**	**Per week**	€351 to €390 ☐1	€391 to €420 ☐2	€421 to €460 ☐3
	**Per Month**	€1,501 to €1,700 ☐1	€1,701 to €1,800 ☐2	€1,801 to €2,000 ☐3
	**Per Year**	€18,001 to €20,000 ☐1	€20,001 to €22,000 ☐2	€22,001 to €24,000 ☐3
**D**	**Per week**	€461 to €500 ☐1	€501 to €535 ☐2	€536 to €575 ☐3
	**Per Month**	€2,001 to €2,150 ☐1	€2,151 to €2,300 ☐2	€2,301 to €2,500 ☐3
	**Per Year**	€24,001 to €26,000 ☐1	€26,001 to €28,000 ☐2	€28,001 to €30,000 ☐3
**E**	**Per week**	€576 to €650 ☐1	€651 to €750 ☐2	€751 to €800 ☐3
	**Per Month**	€2,501 to €2,800 ☐1	€2,801 to €3,250 ☐2	€3,251 to €3,500 ☐3
	**Per Year**	€30,001 to €34,000 ☐1	€34,001 to €38,000 ☐2	€38,001 to €42,000 ☐3
**F**	**Per week**	€801 to €850 ☐1	€851 to €880 ☐2	€881 to €925 ☐3
	**Per Month**	€3,501 to €3,650 ☐1	€3,651 to €3,800 ☐2	€3,801 to €4,000 ☐3
	**Per Year**	€42,001 to €44,000 ☐1	€44,001 to €46,000 ☐2	€46,001 to €48,000 ☐3
**G**	**Per week**	€926 to €1,000 ☐1	€1,001 to €1,050 ☐2	€1,051 to €1,150 ☐3
	**Per Month**	€4,001 to €4,300 ☐1	€4,301 to €4,600 ☐2	€4,601 to €5,000 ☐3
	**Per Year**	€48,001 to €52,000 ☐1	€52,001 to €56,000 ☐2	€56,001 to €60,000 ☐3
**H**	**Per week**	€1,151 to €1,250 ☐1	€1,251 to €1,375 ☐2	€1,376 to €1,500 ☐3
	**Per Month**	€5,001 to €5,500 ☐1	€5,501 to €6,000 ☐2	€6,001 to €6,500 ☐3
	**Per Year**	€60,001 to €66,000 ☐1	€66,001 to €72,000 ☐2	€72,001 to €78,000 ☐3
**I**	**Per week**	€1,501 to €1,600 ☐1	€1,601 to €1,750 ☐2	€1,751 to €1,850 ☐3
	**Per Month**	€6,501 to €7,000 ☐1	€7,001 to €7,500 ☐2	€7,501 to €8,000 ☐3
	**Per Year**	€78,001 to €84,000 ☐1	€84,001 to €90,000 ☐2	€90,001 to €96,000 ☐3
**J**	**Per week**	€1,851 to €2,100 ☐1	€2,101 to €2,400 ☐2	€2,401 or more ☐3
	**Per Month**	€8,001 to €9,250 ☐1	€9,251 to €10,500 ☐2	€10,501 or more ☐3
	**Per Year**	€96,000 to €110,000 ☐1	€110,001 to €125,000 ☐2	€125,001 or more ☐3

G29. Does anyone in your household currently receive any other Social Welfare payments?

Yes ....☐1 No .... ☐2

G30. [Card G30] Now I'd like to record information on any Social Welfare payments which are received by ANYONE in the household. Looking at Card G30, could you tell me whether or not ANYONE in the household currently receives any of these Social Welfare payments? *[Int Tick payments received by any household member]*

**Social Welfare Payment**		**Social Welfare Payment**	
UNEMPLOYMENT PAYMENTS			
Jobseeker's Benefit	☐1	Jobseeker's Allowance or Unemployment Assistance	☐2
EMPLOYMENT SUPPORTS			
Family Income Supplement	☐3	Back to Work Enterprise Allowance	☐6
Farm Assist	☐4	Part-time Job Incentive Scheme	☐7
Back to Work Allowance (Employees)	☐5	Back to Education Allowance	☐8
Supplementary Welfare Allowance (SWA)	☐9	Rural Social Scheme	☐10
ONE-PARENT FAMILY / WIDOW(ER) PAYMENTS			
Widow's or Widower's (Contributory) Pension	☐11	Deserted Wife's Allowance	☐15
Deserted Wife's Benefit	☐12	Prisoner's Wife's Allowance	☐16
Widowed Parent Grant	☐13	One-Parent Family Payment	☐17
Widow's or Widower's (Non-Contrib) Pension	☐14		
CHILD RELATED PAYMENTS			
Maternity Benefit	☐18	Guardian's Payment (Contributory)	☐21
Adoptive Benefit	☐19	Guardian's Payment (Non-Contributory)	☐22
Health & Safety Benefit	☐20	Guardian/Orphan's pension	☐23
DISABILITY AND CARING PAYMENTS			
Illness Benefit	☐24	Prescribed Relative's Allowance	☐32

Invalidity Pension	☐25	Injury Benefit	☐33
Disability Allowance	☐26	Incapacity Supplement	☐34
Blind Pension	☐27	Disablement Benefit	☐35
Carer's Benefit	☐28	Medical Care Scheme	☐36
Domiciliary Care Allowance	☐29	Constant Attendance Allowance	☐37
Carer's Allowance	☐30	Death Benefits (Survivor's Benefits)	☐38
Half-rate Carer's Allowance	☐31		
RETIREMENT PAYMENTS			
State Pension (Transition)	☐39	State Pension Non-Contributory	☐41
State Pension (Contributory)	☐40	Pre-Retirement Allowance	☐42
OTHER PAYMENTS			
Fuel/Smokeless Fuel Allowance	☐43	Diet/heating supplements	☐45
Household Benefits Package (electricity/gas/phone)	☐44		

G31. Does anyone in your household currently receive rent or mortgage supplement? Yes…☐1 No…☐2

G32.How much does the household receive PER WEEK in rent or mortgage supplement? €----------------------

G33. Do you receive or have you received in the last 12 months, any of the following payments? [Tick all that apply]

(a) Back to school clothing and footwear allowance.. ☐1
(b) Exceptional and urgent needs payments (from Community Welfare Officer)........... ☐2
(c) Foster Care Allowance .... ☐3

G34. [Card G34] Looking at Card G34 and thinking of your household's total income from all sources and all household members, approximately what proportion of your total household income would you say comes from social welfare payments of any kind – including Children's Allowance /Child Benefit?

None	Less than 5 %	5% to less than 20%	20% to less than 50%	50% to less than 75%	75% to less than 100%	100%
☐1	☐2	☐3	☐4	☐5	☐6	☐7

G35. [Card G35] For the following items could you indicate whether or not your household has the item and, if not, if it is because you couldn't afford it or for another reason?

	**Yes**	**No, Cannot Afford**	**No, other reason**
a. Does your household eat meals with meat, chicken, fish (or vegetarian equivalent) at least every second day?	☐1	☐2	☐3
b. Does your household have a roast joint (or its equivalent) at least once a week?	☐1	☐2	☐3
c. Do household members buy new rather than second-hand clothes?	☐1	☐2	☐3
d. Does each household member possess a warm waterproof coat?	☐1	☐2	☐3
e. Does each household member possess two pairs of strong shoes?	☐1	☐2	☐3
f. Does the household replace any worn out furniture?	☐1	☐2	☐3
g. Does the household keep the home adequately warm?	☐1	☐2	☐3
h. Does the household have family or friends for a drink or meal once a month?	☐1	☐2	☐3
i. Does the household buy presents for family or friends at least once a year?	☐1	☐2	☐3

G36. [Card G36] A household may have different sources of income and more than one household member may contribute to it. Concerning your household's total monthly or weekly income, with which degree of ease or difficulty is the household able to make ends meet?

With great difficulty	With difficulty	With some difficulty	Fairly easily	Easily	Very easily
☐1	☐2	☐3	☐4	☐5	☐6

G37. Have you ever had to go without heating during the last 12 months through lack of money? (I mean have you had to go without a fire on a cold day, or go to bed to keep warm or light the fire late because of lack of coal/fuel?)

Yes ....☐1 No ....☐2

G38a. Did you have a morning, afternoon or evening out in the last fortnight, for your entertainment (something that cost money)?

Yes ....☐1 No ....☐2

G38b. [Card G38b] Why was that?

Didn't want to .... ☐1
Have a full social life in other ways .... ☐2
Couldn't afford to .... ☐3
Couldn't leave the children .... ☐4
Illness.. ☐5
Other (specify) ____________________ ☐6

G39a. Does your family have a car?

Yes ....☐1 No ....☐2

G39b. Would your family like to have a car but you cannot afford it?

Yes ....☐1 No ....☐2

G40. Since our last interview when <child> was 9 years old we have had major changes in the economy with the recession, cutbacks and unemployment. Would you say that the recession has had:

A very significant effect on your family	A significant effect on your family	A small effect on your family	No effect at all on your family
☐1	☐2	☐3	☐4

G41. [Card G41] How has it affected your family? [Int: tick all that apply]

a. You were made redundant / lost your job .... ☐1
b. Your spouse/partner was made redundant / lost their job .... ☐2
c. Your or your spouse/partner's working hours were reduced......................... ☐3
d. Your or your spouse/partner's wages were reduced .... ☐4
e. Your or your spouse/partner's social welfare benefits were reduced .... ☐5
f. Your family can't afford luxuries (holidays, meals out, etc)............................ ☐6
g. Your family can't afford / had to cut back on basics (food, clothes)............... ☐7
h. You are behind with rent / mortgage payments .... ☐8
i. You are behind with utility bills (e.g. electricity, gas bills, etc)........................ ☐9
j. Other (please specify) .... ☐10

Section H – About You

Now some more questions about yourself

H1. [Card H1] What is the highest level of education (full-time or part-time) which you have completed to date?

1. No formal education ....☐1
2. Primary education...☐2

Second Level

3. Lower Secondary ....☐3

(Junior/Intermediate/Group Certificate. 'O' Levels/GCSEs, NCVA Foundation Certificate, Basic Skills Training Certificate or equivalent).

4. Upper Secondary...☐4

(Leaving Certificate (including Applied and Vocational Programmes). 'A' Levels, NCVA Level 1 Certificate or equivalent

5. Technical or Vocational qualification ....☐5

(Completed Apprenticeship, NCVA Level 2/3 Certificate, Post-Leaving Certificate Course/FETAC Level 5, Teagasc Certificate/Diploma or equivalent).

6. Both Upper Secondary and Technical or Vocational qualification ....☐6

Third Level

7. Non Degree ....☐7

(National Certificate, Diploma NCEA/Institute of Technology or equivalent, Nursing Diploma.)

8. Primary Degree ....☐8

(Third Level Bachelor Degree)

9. Professional qualification (of Degree status at least) ....☐9
10. Both a Degree and a Professional qualification..☐10
11. Postgraduate Certificate or Diploma..☐11
12. Postgraduate Degree (Masters) ....☐12
13. Doctorate (Ph.D) ....☐13

H2. At what age did you leave full-time education for the first time? ______ years
[INTERVIEWER: Code as '0' if respondent never undertook full-time education. Code 999 if still in full time education]

H3. What is <child's> first language?
English .... ☐1 Irish………….☐2 Other (please specify) .... ☐3

H4a. What language do you speak most often at home?
English .... ☐1 Irish………….☐2 Other .... ☐3

H4b. Can I just check, can you read aloud to a child from a children's story book written in your native language?
Yes .... ☐1 No........................ ☐2

H5. Can I just check, can you read aloud to a child from a children's story book written in English?
Yes .... ☐1 No........................ ☐2

H6. Can you usually read and fill out forms you might have to deal with in English?
Yes .... ☐1 No........................ ☐2

H7. When you buy things in shops with a five or ten euro note, can you usually tell if you have the right change?
Yes .... ☐1 No........................ ☐2

H8. Do you belong to any religion?
Yes .... ☐1 No........................ ☐2

H9. [Card H9] Which religion?
1. Christian – no denomination .... ☐1
2. Roman Catholic .... ☐2
3. Anglican/Church of Ireland/Episcopalian .... ☐3
4. Other Protestant... ☐4
5. Jewish .... ☐5
6. Muslim.. ☐6
7. Other (please specify).. ☐7

H10.In general, would you describe yourself as a spiritual person?
Not at all........☐1 A little......... ☐2 Quite.......... ☐3 Very much so .... ☐4 Extremely ..... ☐5

H11. Are you a citizen of Ireland? Yes......... ☐1 No .... ☐2

H12. What citizenship do you hold? ____

H13. Were you born in Ireland? Yes......... ☐1 No .... ☐2

H14. In which country were you born? ____

H15. How long ago did you first come to live in Ireland?

Within the last year	1-5 years ago	6-10 years ago	11-20 years ago	More than 20 years ago	Don't Know
☐1	☐2	☐3	☐4	☐5	☐88

H16. [Card H16] Looking at card H16, can you tell me, what is your ethnic or cultural background? Please choose ONE section from 1 to 4 then tick the appropriate box.

1. White
   - Irish .... ☐1
   - Irish Traveller .... ☐2
   - Any other White background .... ☐3
2. Black or Black Irish
   - African .... ☐4
   - Any other Black background .... ☐5
3. Asian or Asian Irish
   - Chinese .... ☐6
   - Any other Asian background .... ☐7
4. Other, including mixed background .... ☐8

J. Neighbourhood / Community

Finally, we would like to ask you some questions about your local area.

J1. How long have you lived in your local area? _________ years OR ________ months

J2. Do you intend to continue living in Ireland?

Yes .... ☐1   No .... ☐2

J3. [Card J3] How common would you say that each of the things listed below is in your area? For each item listed please say whether or not you think it is very common, fairly common, not very common, or not at all common.

	Very Common	Fairly common	Not very common	Not at all common
Rubbish and litter lying about	☐1	☐2	☐3	☐4
Homes and gardens in bad condition	☐1	☐2	☐3	☐4
Vandalism and deliberate damage to property	☐1	☐2	☐3	☐4
People being drunk or taking drugs in public	☐1	☐2	☐3	☐4

J4. [Card J4] To what extent do you agree or disagree with these statements?

	Strongly Agree	Agree	Disagree	Strongly Disagree
A. This is a safe area for my 13 year old	☐1	☐2	☐3	☐4
B. It is safe for me to walk alone in this area after dark	☐1	☐2	☐3	☐4
C. As a family we are happy living in this area	☐1	☐2	☐3	☐4
D. We as a family intend to continue living in this area	☐1	☐2	☐3	☐4
E. There are places in this area where teenagers can safely hang out	☐1	☐2	☐3	☐4
F. There are facilities such as youth clubs, swimming clubs, sports clubs, for teenagers in this area.	☐1	☐2	☐3	☐4

PRIMARY CAREGIVER SENSITIVE QUESTIONNAIRE

The Economic and Social Research Institute
Whitaker Square
Sir John Rogerson's Quay
Dublin 2
Ph: 01-863 2000 Fax 01-863 2100

An Roinn Leanaí agus Gnóthaí Óige
Department of Children and Youth Affairs

University of Dublin
Trinity College
College Green
Dublin 2

GROWING UP IN IRELAND – the national longitudinal study of children
STRICTLY CONFIDENTIAL

Primary Caregiver – SUPPLEMENTARY SECTION, 13-Year Main

AREA ☐☐☐☐ **HHOLD** ☐☐☐

Interviewer Name______________________________ **Interviewer Number** ☐☐☐☐☐

Time Section Started ☐☐☐☐ **(24 hour clock)** **Date** ____ ____ ____
day mth year

We have a few final questions which we would like to discuss with you. As some of these may be considered slightly sensitive we have included them in a section for you to complete by yourself. We would ask you to complete this section and return it to the interviewer. Once again, we would like to assure you that ALL THE INFORMATION PROVIDED IS TREATED IN THE STRICTEST CONFIDENCE.

X1. Are you male or female?

Male............. ☐1 Female ....☐2

X2. What is your date of birth? __ __ /__ __/ __ __ __ __
DD / MM / YYYY

IF ANY PERSON ON HOUSEHOLD GRID AT TIME 1 IS NO LONGER RESIDENT IN THE HOUSEHOLD AT TIME 2 ASK AS1 – AS3]:

AS1. Can you please tell me why <Person at Wave 1> is no longer resident in the household.

He/she is deceased .... ☐1
We separated/divorced .... ☐2
He/she moved out to set up own household.. ☐3
Long-term absence (e.g. hospital, prison, military service abroad)................................. ☐4
Other (please specify).................................. ☐5

AS2. When did <Person from Wave 1> stop living with you: Since what year? [YYYY]

AS3. When did <Person from Wave 1> stop living with you: Since what month? _______ mth

S1. Are you the biological parent of <child>?

Yes................☐1 → **Go to S12** No................ ☐2 → **Go to S2**

S2. Are you the adoptive parent of <child>?

Yes................☐1 No................ ☐2 → **Go to S7**

S3. Was that a domestic or an inter-country adoption?

Domestic..........☐1 Inter-country ....☐2

S4. Was this a within family adoption?

Yes ……… ☐1 No …….. ☐2

S5. From which country?

____

S6. What age was <child> when you adopted him/ her? ____________years

NOW PLEASE GO TO S12

S7. Are you the foster parent of <child>?

Yes................☐1 No................ ☐2 → **Go to S12**

S8. How long has <child> been with your family? ________ years ________ months

S9. Do you anticipate that this will be a long-term foster placement? Yes ……….☐1 No …………..☐2

S10. How many previous foster placements has <child> been in? ______previous placements Don't Know…☐99

S11a. Immediately before coming to live with you was <child> living with another foster family, his/her family or in institutional care?

Another foster family........☐1 Own family ....☐2 Institutional care ....☐3

S11b. Are you related to <child> Yes ……….☐1 No …………..☐2 → **Go to S12**

S11c. How are you related to <child> ____

NOW PLEASE GO TO S12

Because the issue of family life is so important we would now like to ask some questions about your family and marital history.

S12. Can you tell me which of these best describes your current marital status?

Married and living with husband / wife .... ☐1 **Go to S13a**
Married and separated from husband / wife .... ☐2 **Go to S13b**
Divorced .... ☐3 **Go to S13b**
Widowed .... ☐4 **Go to S13b**
Never married .... ☐5 **Go to S15**

S13a. In what year did you marry your husband / wife? ________(year) **Go to S16**

S13b. In what year did you marry your (former) spouse?________(year) **Go to S14**

S14. Since when have you been living apart / spouse deceased? ___________(year) **Go to S15**

S15. May I just check whether you are currently living with someone in the household as a couple?

Yes.....................☐1 No.....................☐2 **Go to S21**

S16. Since when have you and your spouse or partner been living together?_________ (mth) ________(year)

S17. Many couples argue from time to time. Roughly how often would you and your spouse / partner argue?

Most days..☐1 → **Go to S18**
At least once a week............................☐2 → **Go to S18**
Less than once a week ....☐3 → **Go to S18**
Hardly ever..☐4 → **Go to S18**
Never..☐5 → **Go to S19**

S18. When you and your partner argue, how often do you ….

	Almost never/ Never	Not very often	Sometimes	Often	Almost always/ always
Shout or yell at each other	☐1	☐2	☐3	☐4	☐5
Throw something at each other	☐1	☐2	☐3	☐4	☐5
Push, hit or slap each other	☐1	☐2	☐3	☐4	☐5

S19. How often would you say the following happen in your relationship?

	All the time	Most of the time	More often than not	Occasionally	Rarely	Never
You discuss or have considered divorce, separation, or terminating your relationship	☐1	☐2	☐3	☐4	☐5	☐6
You think that things between you and your partner are going well	☐1	☐2	☐3	☐4	☐5	☐6
You confide in your mate / partner	☐1	☐2	☐3	☐4	☐5	☐6

S20. The numbers below represent different degrees of happiness in your relationship. The middle point, "happy," represents the degree of happiness of most relationships. Please circle the number which best describes the degree of happiness, all things considered, of your relationship.

0	1	2	3	4	5	6
Extremely Unhappy	Fairly Unhappy	A little unhappy	Happy	Very Happy	Extremely Happy	Perfect

S21. Please rate how much you agree or disagree with each of the following statements in relation to how things are for you and the Study Child right now. Remember, there no right or wrong answers, just try to be as honest as possible

	Strongly Disagree	Disagree	Not Sure	Agree	Strongly Agree
A. Caring for my child sometimes takes more time and energy than I have to give.	☐1	☐1	☐3	☐4	☐5
B. I sometimes worry whether I am doing enough for my child.	☐1	☐1	☐3	☐4	☐5
C. The major source of stress in my life is my child.	☐1	☐1	☐3	☐4	☐5
D. Having my child leaves little time and flexibility in my life	☐1	☐1	☐3	☐4	☐5
E. Having my child has been a financial burden	☐1	☐1	☐3	☐4	☐5
F. It is difficult to balance different responsibilities because of my child.	☐1	☐1	☐3	☐4	☐5

[ASK S22 ONLY OF FEMALE RESPONDENTS]

S22. Are you currently pregnant? Yes.......... ☐1 No............ ☐2

S23. Which of the following best describes how often you usually drink alcohol?

1. Never............ ☐1 **Go to S26**
2. Less than once a month............ ☐2
3. 1-2 times a month............ ☐3
4. 1-2 times a week............ ☐4
5. 3-4 times a week............ ☐5
6. 5-6 times a week............ ☐6
7. Every day............ ☐7

If currently drink alcohol between everyday and 1-2 times a week ask:

S24. And in an average week, how many pints of beer/cider, glasses of wine, measures of spirit, and bottles of alcopops would you drink?

(a) Pints of Beer/Cider ___ **(b) Glasses of Wine** ___
(c) Measures of Spirits ___ **(d)Bottles of alcopops** ___

For the following questions please consider that 1 drink = ½ pint of beer or 1 glass of wine or 1 single spirits

[ASK S25a ONLY OF FEMALE RESPONDENTS]

S25a. How often do you have 6 or more alcoholic drinks on one occasion?

Never	Less than monthly	Monthly	Weekly	Daily or almost daily
☐1	☐2	☐3	☐4	☐5

[ASK S25b ONLY OF MALE RESPONDENTS]

S25b. How often do you have 8 or more alcoholic drinks on one occasion?

Never	Less than monthly	Monthly	Weekly	Daily or almost daily
☐1	☐2	☐3	☐4	☐5

S25c. How often during the last year have you been unable to remember what happened the night before because you had been drinking?

Never	Less than monthly	Monthly	Weekly	Daily or almost daily
☐1	☐2	☐3	☐4	☐5

S25d. How often during the last year have you failed to do what was expected of you because of drinking?

Never	Less than monthly	Monthly	Weekly	Daily or almost daily
☐1	☐2	☐3	☐4	☐5

S25e. In the last year has a relative or friend, or a doctor or other health worker been concerned about your drinking or suggested you cut down?

No .... ☐1 Yes, on one occasion.......... ☐2 Yes on more than one occasion .... ☐3

S26. Do you currently smoke daily, occasionally or not at all?

Daily .... ☐1 Occasionally .... ☐2 Not at all .... ☐3

27. About how many cigarettes or cigars do you smoke on average each day?

____________ [Int. enter '0' if less than 1 on average]

S28. Including yourself, how many members of the household smoke? ____N

S29. Do you take any drugs such as cannabis, marijuana, ecstasy, speed, heroin, methadone, crack or cocaine?

Regularly................ ☐1 Occasionally........... ☐2 Not at all .... ☐3

S30a. Since the time of the last interview when <child> was 9 years of age, have you been treated by a medical professional for clinical depression, anxiety, 'nerves' or phobias?

Yes…... ☐1 No……. ☐2

S30b. Are you currently taking medication for clinical depression, anxiety, 'nerves' or phobias?

Yes…... ☐1 No……. ☐2

S31. Listed below are 8 statements about some of the ways you may have felt or behaved. Please indicate how often you have felt this way *during the past week*.

	Rarely or none of the time (less than 1 day)	Some or a little of the time (1-2 days)	Occasionally or a moderate amount of the time (3-4 days)	Most or all of the time (5-7 days)
a. I felt I could not shake off the blues even with help from my family or friends	☐1	☐2	☐3	☐4
b. I felt depressed	☐1	☐2	☐3	☐4
c. I thought my life had been a failure	☐1	☐2	☐3	☐4
d. I felt fearful	☐1	☐2	☐3	☐4
e. My sleep was restless	☐1	☐2	☐3	☐4
f. I felt lonely	☐1	☐2	☐3	☐4
g. I had crying spells	☐1	☐2	☐3	☐4
h. I felt sad	☐1	☐2	☐3	☐4

S32. Have you ever been in trouble with the Gardai or Police (in Ireland or elsewhere) (other than for traffic offences)?

Yes......... ☐1 No .... ☐2 → **Go to S34**

S33. Have you ever been to prison? Yes .... ☐1 No .... ☐2

S34. To the best of your knowledge, has <child> ever tried?

	**Yes, and I know about it**	**Probably**	**Possibly**	**I don't think so**
a. Alcohol	☐1	☐2	☐3	☐4
b. Cigarettes	☐1	☐2	☐3	☐4
c. Cannabis/Marijuana	☐1	☐2	☐3	☐4

S35. Have you spoken to your child personally about the following sexual health issues?

	Yes	No
1. Sex and sexual intercourse	☐1	☐2
2. Sexual feelings, relationships and emotions	☐1	☐2
3. Contraception	☐1	☐2
4. Safer sex/sexually transmitted infections/ venereal diseases	☐1	☐2
5. Sexual orientation (eg. Homosexuality, heterosexuality etc)	☐1	☐2

S36. Can we check, does <child's> biological father/ mother live here with you or elsewhere?

Lives here ☐1 → **Go to S48**
Deceased ☐2 → **Go to S48**
Temporarily lives elsewhere ☐3 → **Go to S48**
Lives elsewhere ☐4 → **Go to S37**

S37. Were you ever married to or did you ever live with <child's> biological father / mother?

Yes, married to ☐1 Yes, lived with ☐2 No ☐3 **Go to S39** Adoptive / Foster parent ☐4 **Go to S48**

S38. What age was the Study Child when you split or separated from their biological father / mother?

____

S39. Do you have a formal or informal parenting arrangement regarding <child> and where he / she lives?

Formal ☐1 Informal ☐2 No parenting arrangement ☐3

S40. Briefly describe that arrangement

____

____

S41. How did you arrive at that arrangement?

Court imposed arrangements ☐1
Formal negotiated arrangements other than legal (e.g. counsellor) ☐2
Mutual agreement with no third party negotiator ☐3

S42. How far does <child's> biological father / mother live from here?

Within ½ hour's drive from here ☐1
Between ½ and 1 hour's drive from here ☐2
More than 1 hour's drive from here ☐3
Outside the country ☐4

S43. How often does <child> have contact with his / her biological father / mother?

Daily ☐1
More than once a week ☐2
Once a week ☐3
Every second week / weekend ☐4
Monthly ☐5
Less than once a month ☐6
No contact ☐7

S44. Does <child's> biological father / mother make ANY financial contribution to your household and the maintenance of <child>? Include any form of financial support such as rent, mortgage, direct maintenance payment etc.

No, he/she never makes any payment ☐1
Yes, he/she makes a regular payment ☐2
Yes, he/she makes payments from time to time ☐3

S45. How often do you talk to <child's> biological father/ mother about <child>?

Every day	Several times a week	About once a week	A few times a month	Several times a year	Never
☐1	☐2	☐3	☐4	☐5	☐6

S46. How well do you get on with <child's> biological father/ mother? Would you say your relationship is?

Very positive	Positive	Neither positive nor negative	Somewhat negative	Very negative
☐1	☐2	☐3	☐4	☐5

S47. We would like to send a short questionnaire to <child's> biological father/ mother. We would be happy to show you the content of this questionnaire before we send it. Would you be able to provide us with contact details for <child's> biological father/ mother?

Yes .... ☐1 → **Please give contact details to interviewer**
No, I do not wish other parent to be contacted .... ☐2
No, I do not have contact details for other parent .... ☐3

S48. THANK YOU VERY MUCH FOR TAKING PART IN THE *GROWING UP IN IRELAND* PROJECT.

SECONDARY CAREGIVER MAIN QUESTIONNAIRE

The Economic and Social Research Institute
Whitaker Square
Sir John Rogerson's Quay
Dublin 2
Ph: 01-863 2000 Fax 01-863 2100

An Roinn Leanaí agus Gnóthaí Óige
Department of Children and Youth Affairs

University of Dublin
Trinity College
College Green
Dublin 2

TRINITY COLLEGE DUBLIN

GROWING UP IN IRELAND – the national longitudinal study of children
STRICTLY CONFIDENTIAL

SECONDARY CAREGIVER QUESTIONNAIRE – 13-Year Main

AREA ☐☐☐☐ **HOUSEHOLD** ☐☐☐

Interviewer Name___________________________ **Interviewer Number** ☐☐☐☐☐

Date ____ _____ _____
day month year

Almost four years have passed since you and your family were interviewed as part of *Growing Up in Ireland.* At that time we explained that we would like to make a return visit to your home for a follow-up interview to see how your child has changed and grown since our last visit. We are now seeking to interview the parents/guardians of <child>. The whole interview with the parents/guardians and child will take about 1½ - 2 hours to complete [INTERVIEWER: Adjust as appropriate for you in the field]. All the information you and your family provide will be treated in the strictest confidence and will not be released in any way which would allow the information you provide to be identified with you or your family. If however, we are told something which might suggest that a child or other vulnerable person is at risk we may have to act on it.

The Department of Health and Children is funding the study through the Department of Children and Youth Affairs, in association with the Department of Social Protection and the Central Statistics Office. The Department of Education and Science is represented on the Steering Group which oversees the Study. A group of researchers led by the Economic and Social Research Institute (ESRI) and The Children's Research Centre at Trinity College Dublin is carrying out the study

Section A - Introduction

[A1 – BLAISE INSTRUCTION – ASK A1 IF NEW PARTER AT TIME 2 OR SECONDARY CAREGIVER WAS NON RESPONDENT AT TIME 1]

A1. [Card A1] Can you please tell me which of the following best describes your relationship to <child>?
[Interviewer use codes only]

1. Biological mother/ father .... ☐1
2. Adoptive mother/ father .... ☐2
3. Step-mother / Step-father / Partner of child's parent ☐3
4. Foster mother / father .... ☐4
5. Grand parent .... ☐5
6. Aunt/uncle .... ☐6
7. Other relative/ in law .... ☐7
8. Unrelated guardian.................................. ☐8

Section B - Parental Health

Now I'd like to ask you a few questions about your own health.

B1. [CARD B1] In general, how would you say your current health is?

Excellent............. ☐1
Very good........... ☐2
Good .... ☐3
Fair .... ☐4
Poor.................... ☐5

B2. Do you have any on-going chronic physical or mental health problem, illness or disability?

Yes .... ☐1 No .... ☐2

B3. What is the nature of this problem, illness or disability? Please describe as fully as possible.
[Int. please record diagnosis – not symptoms of the problem.]

____

____

B4. Since when have you had this problem, illness or disability? ___________ (year) ______(month)

B5. Are you hampered in your daily activities by this problem, illness or disability?

Yes, severely .... ☐1 Yes, to some extent .... ☐2 No .... ☐3

B6. Thinking about your free-time, in general would you say you are…

Very physically active.......................... ☐1
Fairly physically active .... ☐2
Not very physically active.................... ☐3
Not at all physically active .... ☐4

C: FAMILY CONTEXT

Now I'd like to ask you some general questions about your family as a whole.

C1. [Card C1] I am going to read out some statements about the relationship between you and your child. Please listen to each statement and describe the degree to which each of the following statements currently applies.

	Definitely does not apply	Not really	Neutral, not sure	Applies somewhat	Definitely applies
A. I share an affectionate, warm relationship with my child	☐1	☐2	☐3	☐4	☐5
B. My child and I always seem to be struggling with each other..............	☐1	☐2	☐3	☐4	☐5
C. If upset, my child will seek comfort from me.	☐1	☐2	☐3	☐4	☐5
D. My child is uncomfortable with physical affection or touch from me.	☐1	☐2	☐3	☐4	☐5
E. My child values his/her relationship with me.	☐1	☐2	☐3	☐4	☐5
F. When I praise my child, he/she beams with pride.	☐1	☐2	☐3	☐4	☐5
G. My child spontaneously shares information about himself/herself	☐1	☐2	☐3	☐4	☐5
H. My child easily becomes angry at me.	☐1	☐2	☐3	☐4	☐5
I. It is easy to be in tune with what my child is feeling.	☐1	☐2	☐3	☐4	☐5
J. My child remains angry or is resistant after being disciplined	☐1	☐2	☐3	☐4	☐5
K. Dealing with my child drains my energy.	☐1	☐2	☐3	☐4	☐5
L. When my child is in a bad mood, I know we're in for a long and difficult day	☐1	☐2	☐3	☐4	☐5
M. My child's feelings toward me can be unpredictable or can change suddenly	☐1	☐2	☐3	☐4	☐5
N. My child is sneaky or manipulative with me.	☐1	☐2	☐3	☐4	☐5
O. My child openly shares his/her feelings and experiences with me.	☐1	☐2	☐3	☐4	☐5

C2. [Card C2] The following are some questions on your knowledge of what <child> does in his/her free time, where he/she goes, and who he/she has as friends.

	Almost never or never	Not very often	Sometimes	Often	Almost always or always	N/A
A. Do you know what <Study Child> does with his/her free time.	☐1	☐2	☐3	☐4	☐5	☐6
B. Do you know who he/she has as friends during his/her free time.	☐1	☐2	☐3	☐4	☐5	☐6
C. Do you usually know what type of homework he/she has.	☐1	☐2	☐3	☐4	☐5	☐6
D. Do you know what he/she spends his/her money on	☐1	☐2	☐3	☐4	☐5	☐6
E. Do you know when he/she has a test or homework due at school.	☐1	☐2	☐3	☐4	☐5	☐6
F. Do you know how he/she does in different subjects at school.	☐1	☐2	☐3	☐4	☐5	☐6
G. Do you know where he/she goes when out at night with friends	☐1	☐2	☐3	☐4	☐5	☐6
H. Do you know where he/she goes and what he/she does after school.	☐1	☐2	☐3	☐4	☐5	☐6
I. How often in the last month have you had no idea where he/she was.	☐1	☐2	☐3	☐4	☐5	☐6

C3. [CARD C3] The following are some questions about how much <child> actually tells you about what he/she is doing, without being asked.

	Almost never or never	Not very often	Sometimes	Often	Almost always or always	N/A
A. Does he/she spontaneously tell you about his/her friends.	☐1	☐2	☐3	☐4	☐5	☐6
B. Does he/she want to tell you about school (how subjects are going; relationships with teachers etc).	☐1	☐2	☐3	☐4	☐5	☐6
C. Does he/she keep a lot of secrets from you about what he/she is doing in his/her spare time	☐1	☐2	☐3	☐4	☐5	☐6
D. Does he/she hide a lot from you about what he/she is doing during nights and weekends	☐1	☐2	☐3	☐4	☐5	☐6
E. Does he/she like to tell you what he/she has been doing and where he/she went when out for the evening	☐1	☐2	☐3	☐4	☐5	☐6

C4. [CARD C4] Please tell me how strongly you agree or disagree with the following.

	Strongly Disagree	Disagree	Neither agree nor disagree	Agree	Strongly Agree	N/A
**Because of your work responsibilities:**						
A. You have missed out on home or family activities that you would have liked to have taken part in	☐1	☐2	☐3	☐4	☐5	☐6
B. Your family time is less enjoyable and more pressured	☐1	☐2	☐3	☐4	☐5	☐6
**Because of your family responsibilities:**						
C. You have to turn down work activities or opportunities you would prefer to take on	☐1	☐2	☐3	☐4	☐5	☐6
D. The time you spend working is less enjoyable and more pressured	☐1	☐2	☐3	☐4	☐5	☐6

C5. How fairly or unfairly would you say the household tasks are distributed between you and your partner? Would you say…[INT: READ OUT]

Very unfairly .... ☐1 Quite unfairly .... ☐2 Fairly .... ☐3 Don't have partner. .... ☐4

C6. [Show Card C6] I'd like to ask you about the time <child> spends with you including times when others are present. How many days per week do you:

	Every day / 7 days per week	3 to 6 days per week	1 to 2 days per week	1 to 2 times per month	Rarely or never
A. Sit down to eat together	☐1	☐2	☐3	☐4	☐5
B. Play sports, cards or games together	☐1	☐2	☐3	☐4	☐5
C. Talk about things together	☐1	☐2	☐3	☐4	☐5
D. Do household activities together (e.g. gardening, cooking, cleaning, etc)	☐1	☐2	☐3	☐4	☐5
E. Go on an outing together (e.g. going to the cinema, theatre, walking, shopping)	☐1	☐2	☐3	☐4	☐5

C7a. Thinking of an <u>AVERAGE SCHOOL DAY</u>, what amount of time in total would you say you spend with <child> either alone or with others (this could be watching TV, going shopping etc)

_______hours _____minutes

C7b. And thinking of an <u>AVERAGE WEEKEND</u>, what amount of time in total would you say you spend with <child> either alone or with others (this could be watching TV, going shopping etc)

_______hours _____minutes

D: SOCIO-DEMOGRAPHICS

Now some questions about the circumstances of your household.

D1. [Card D1] Looking at Card D1, which of these descriptions *BEST* describes your usual situation in regard to work?

[Int: If respondent is on maternity leave and she has a job which she intends to return to, she should be coded as 0]

0. Currently on maternity leave, but with a job to return to ☐0
1. Employee (incl. apprenticeship or Community Employment) ☐1
2. Self employed outside farming ☐2
3. Farmer ☐3
4. Student full-time ☐4
5. On State training scheme (FAS, Failte Ireland etc.) ☐5
6. Unemployed, actively looking for a job ☐6
7. Long-term sickness or disability ☐7
8. Home duties / looking after home or family ☐8
9. Retired ☐9
10. Other (specify) ______________________ ☐10

[BLAISE CONDITION: IF RESPONDENT NOT WORKING AT WAVE 1 BUT IS WORKING AT WAVE 2 <u>OR</u> RESPONDENT ON MATERNITY LEAVE AT WAVE 1 BUT IS WORKING AT WAVE 2 ASK D2a:]

D2. When did you return to work? ______ mth ________ year

D3. How many hours do you normally work per week, including any regular overtime work? If you work at more than one job, please include the hours in all jobs. _____________ hours

D4. On a typical work day, how much time in minutes do you spend commuting to and from work (outward and return journey combined)?

__________ minutes [Int. if respondent works at home enter '0' for minutes]

D5. [Card D5] What is your occupation in your main job?

In all cases describe the occupation fully and precisely giving the full job title.

Use precise terms such as:	Do not use general terms such as:
RETAIL STORE MANAGER	MANAGER
SECONDARY TEACHER	TEACHER
ELECTRICAL ENGINEER	ENGINEER

Civil servants and local government employees should state their grade e.g. SENIOR ADMINISTRATIVE OFFICER.
Members of the Gardai or Army should state their rank. Teachers should state the branch of teaching e.g. PRIMARY TEACHER.
Clergy and religious orders should give full description e.g. NUN, REGISTERED GENERAL NURSE.

Write in your main OCCUPATION

____

D6. Do you supervise or manage any personnel in your job?

Yes ☐1 No ☐2

D7. How many? ____________________________

D8. How many employees (if any) do you have?__________ employees N A .... ☐99

D9. [Ask only if Farmer at D1.] What is the acreage of the farm? _______________ acres

Go to E1

D10. Apart from holiday or casual work, have you ever had a full-time job? Yes ....☐1 No....☐2 **Go to D17**

D11. In what year did you last work in that full-time job? ________ **year**

D12. When you last worked in that full-time job were you?

Employee (incl. apprenticeship or Community Employment) ....☐1 Self-employed outside farming ..... ☐2 Farmer ....☐3

D13. [Card D13] What was your occupation in that job? (What did you mainly do in your job?) Please describe as fully as possible

In all cases please describe the occupation fully and precisely giving the full job title.

Use precise terms such as:	Do not use general terms such as:
RETAIL STORE MANAGER	MANAGER
SECONDARY TEACHER	TEACHER
ELECTRICAL ENGINEER	ENGINEER

Civil servants and local government employees should state their grade e.g. SENIOR ADMINISTRATIVE OFFICER.
Members of the Gardai or Army should state their rank. Teachers should state the branch of teaching e.g. PRIMARY TEACHER.
Clergy and religious orders should give full description e.g. NUN, REGISTERED GENERAL NURSE.

Write in your main OCCUPATION

____

D14a. Did you supervise or manage any personnel in your job?

Yes ....☐1 No .... ☐2

D14b. How many? ____________________

D15. How many employees (if any) did you have?__________ **employees** N A .... ☐99

D16. [Ask only if Farmer at D12] What was the acreage of the farm? _______________ acres

Go to E1

D17. Do you currently have a part time job outside the home? Yes ....☐1 No........☐2 **Go to D20**

D18. On average, how many hours per week do you work in that part-time job? ____________ **hours**

D19. [Card D19] What is your occupation in that job?

In all cases describe the occupation fully and precisely giving the full job title.

Use precise terms such as:	Do not use general terms such as:
RETAIL STORE MANAGER	MANAGER
SECONDARY TEACHER	TEACHER
ELECTRICAL ENGINEER	ENGINEER

Civil servants and local government employees should state their grade e.g. SENIOR ADMINISTRATIVE OFFICER.
Members of the Gardai or Army should state their rank. Teachers should state the branch of teaching e.g. PRIMARY TEACHER.
Clergy and religious orders should give full description e.g. NUN, REGISTERED GENERAL NURSE.

Write in your main OCCUPATION

____

If a farmer or a farm worker, write in the SIZE of the farm _________ **acres**

D20. [Card D20] From the reasons listed on this card could you tell me the most important reasons for you not working in a paid job outside the home? If more than one reason, please rank them in order of importance, where 1 is the most important reason, up to a maximum of 3.

a. I can't find a job .... ____
b. I chose not to work .... ____
c. I am caring for an elderly or ill relative or friend .... ____
d. I prefer be at home to look after my children myself .. ____
e. I cannot earn enough to pay for childcare .... ____
f. I cannot find suitable childcare .... ____
g. There are no suitable jobs available for me .. ____
h. My family would lose Social Welfare or medical benefits if I was earning .... ____
i. Other reason (specify)________________ ____

E: ABOUT YOU

Now some more questions about yourself

E1. [Card E1] What is the highest level of education (full-time or part-time) which you have completed to date?

1. No formal education .... ☐1
2. Primary education .... ☐2

Second Level

3. Lower Secondary .... ☐3
(Junior/Intermediate/Group Certificate. 'O' Levels/GCSEs, NCVA Foundation Certificate, Basic Skills Training Certificate or equivalent).

4. Upper Secondary .... ☐4
(Leaving Certificate (including Applied and Vocational Programmes). 'A' Levels, NCVA Level 1 Certificate or equivalent

5. Technical or Vocational qualification .... ☐5
(Completed Apprenticeship, NCVA Level 2/3 Certificate, Post-Leaving Certificate Course/FETAC Level 5, Teagasc Certificate/Diploma or equivalent).

6. Both Upper Secondary and Technical or Vocational qualification .... ☐6

Third Level

7. Non Degree .... ☐7
(National Certificate, Diploma NCEA/Institute of Technology or equivalent, Nursing Diploma.)

8. Primary Degree .... ☐8
(Third Level Bachelor Degree)

9. Professional qualification (of Degree status at least) .... ☐9
10. Both a Degree and a Professional qualification .... ☐10
11. Postgraduate Certificate or Diploma .... ☐11
12. Postgraduate Degree (Masters) .... ☐12
13. Doctorate (Ph.D) .... ☐13

E2. At what age did you leave full-time education for the first time? ______ years
[INTERVIEWER: Code as '0' if respondent never undertook full-time education. Code 999 if still in full time education]

E3a. What language do you speak most often at home?

English .... ☐1 Irish………… ☐2 Other .... ☐3

E3b. Can I just check, can you read aloud to a child from a children's story book written in your native language?

Yes .... ☐1 No........................ ☐2

E4. Can I just check, can you read aloud to a child from a children's story book written in English?

Yes .... ☐1 No........................ ☐2

E5. Can you usually read and fill out forms you might have to deal with in English?

Yes .... ☐1 No........................ ☐2

E6. When you buy things in shops with a five or ten euro note, can you usually tell if you have the right change?

Yes .... ☐1 No........................ ☐2

E7. Do you belong to any religion?

Yes .... ☐1 No........................ ☐2

E8. [Card E8] Which religion?

Christian – no denomination .... ☐1
Roman Catholic .... ☐2
Anglican/Church of Ireland/Episcopalian .... ☐3
Other Protestant... ☐4
Jewish .... ☐5
Muslim .... ☐6
Other (please specify)... ☐7

E9.In general, would you describe yourself as a spiritual person?

Not at all........ ☐1 A little......... ☐2 Quite.......... ☐3 Very much so .... ☐4 Extremely ..... ☐5

E10. Are you a citizen of Ireland? Yes......... ☐1 No .... ☐2

E11. What citizenship do you hold? ______________________

E12. Were you born in Ireland? Yes......... ☐1 No .... ☐2

E13. In which country were you born? ______________________

E14. How long ago did you first come to live in Ireland?

Within the last year	1-5 years ago	6-10 years ago	11-20 years ago	More than 20 years ago	Don't Know
☐1	☐2	☐3	☐4	☐5	☐88

E15. [Card E15 What is your ethnic or cultural background?
Please choose ONE section from 1 to 4 then tick the appropriate box.

1. White
   - Irish... ☐1
   - Irish Traveller.. ☐2
   - Any other White background .... ☐3
2. Black or Black Irish
   - African .... ☐4
   - Any other Black background .... ☐5
3. Asian or Asian Irish
   - Chinese .... ☐6
   - Any other Asian background .... ☐7
4. Other, including mixed background..................... ☐8

SECONDARY CAREGIVER SENSITIVE QUESTIONNAIRE

The Economic and Social Research Institute
Whitaker Square
Sir John Rogerson's Quay
Dublin 2
Ph: 01-863 2000 Fax 01-863 2100

An Roinn Leanaí agus Gnóthaí Óige
Department of Children and Youth Affairs

University of Dublin
Trinity College
College Green
Dublin 2

GROWING UP IN IRELAND – the national longitudinal study of children
STRICTLY CONFIDENTIAL

Secondary Caregiver – SUPPLEMENTARY SECTION, 13-Year Main

AREA ☐☐☐☐ **HHOLD** ☐☐☐

Interviewer Name____________________________ **Interviewer Number** ☐☐☐☐☐

Time Section Started ☐☐☐☐ **(24 hour clock)** **Date** ____ ____ ____
day mth year

We have a few final questions which we would like to discuss with you. As some of these may be considered slightly sensitive we have included them in a section for you to complete by yourself. We would ask you to complete this section and return it to the interviewer. Once again, we would like to assure you that ALL THE INFORMATION PROVIDED IS TREATED IN THE STRICTEST CONFIDENCE.

X1. Are you male or female?

Male.............. ☐1   Female .... ☐2

X2. What is your date of birth? __ __ /__ __/ __ __ __ __
DD / MM / YYYY

S1. Are you the biological parent of <child>?

Yes................ ☐1 → **Go to S12**   No................. ☐2 → **Go to S2**

S2. Are you the adoptive parent of <child>?

Yes................ ☐1   No................. ☐2 → **Go to S7**

S3. Was that a domestic or an inter-country adoption?

Domestic............ ☐1   Inter-country .... ☐2

S4. Was this a within family adoption?

Yes ……… ☐1   No …….. ☐2

S5. From which country?

____

S6. What age was <child> when you adopted him/ her? ____________years

NOW PLEASE GO TO S12

S7. Are you the foster parent of <child>?

Yes................ ☐1   No................. ☐2 → **Go to S12**

S8. How long has <child> been with your family? ________ years ________ months

S9. Do you anticipate that this will be a long-term foster placement? Yes ………. ☐1 No ………….. ☐2

S10. How many previous foster placements has <child> been in? ______previous placements Don't Know… ☐99

S11a. Immediately before coming to live with you was <child> living with another foster family, his/her family or in institutional care?

Another foster family........ ☐1   Own family .... ☐2   Institutional care .... ☐3

S11b. Are you related to <child> Yes ………..☐1 No …………..☐2 ⟶**Go to S12**

S11c. How are you related to <child> ____

NOW PLEASE GO TO S12

Because the issue of family life is so important we would now like to ask some questions about your family and marital history.

S12. Can you tell me which of these best describes your current marital status?

Married and living with husband / wife .... ☐1 **Go to S13a**
Married and separated from husband / wife .... ☐2 **Go to S13b**
Divorced .... ☐3 **Go to S13b**
Widowed .... ☐4 **Go to S13b**
Never married .... ☐5 **Go to S15**

S13a. In what year did you marry your husband / wife? ________(year) **Go to S16**

S13b. In what year did you marry your (former) spouse?________(year) **Go to S14**

S14. Since when have you been living apart / spouse deceased? ___________(year) **Go to S15**

S15. May I just check whether you are currently living with someone in the household as a couple?

Yes.....................☐1 No.....................☐2 **Go to S21**

S16. Since when have you and your spouse or partner been living together?_________ (mth) ________(year)

S17. Many couples argue from time to time. Roughly how often would you and your spouse / partner argue?

Most days...☐1 →**Go to S18**
At least once a week............................☐2 →**Go to S18**
Less than once a week ....☐3 →**Go to S18**
Hardly ever...☐4 →**Go to S18**
Never..☐5 →**Go to S19**

S18. When you and your partner argue, how often do you ….

	Almost never/ Never	Not very often	Sometimes	Often	Almost always/ always
Shout or yell at each other	☐1	☐2	☐3	☐4	☐5
Throw something at each other	☐1	☐2	☐3	☐4	☐5
Push, hit or slap each other	☐1	☐2	☐3	☐4	☐5

S19. How often would you say the following happen in your relationship?

	All the time	Most of the time	More often than not	Occasionally	Rarely	Never
You discuss or have considered divorce, separation, or terminating your relationship.........	☐1	☐2	☐3	☐4	☐5	☐6
You think that things between you and your partner are going well	☐1	☐2	☐3	☐4	☐5	☐6
You confide in your mate / partner......................	☐1	☐2	☐3	☐4	☐5	☐6

S20. The numbers below represent different degrees of happiness in your relationship. The middle point, “happy,” represents the degree of happiness of most relationships. Please circle the number which best describes the degree of happiness, all things considered, of your relationship.

0	1	2	3	4	5	6
**Extremely Unhappy**	**Fairly Unhappy**	**A little unhappy**	**Happy**	**Very Happy**	**Extremely Happy**	**Perfect**

S21. Please rate how much you agree or disagree with each of the following statements in relation to how things are for you and the Study Child right now. Remember, there no right or wrong answers, just try to be as honest as possible

	Strongly Disagree	Disagree	Not Sure	Agree	Strongly Agree
A. Caring for my child sometimes takes more time and energy than I have to give.	☐1	☐1	☐3	☐4	☐5
B. I sometimes worry whether I am doing enough for my child.	☐1	☐1	☐3	☐4	☐5
C. The major source of stress in my life is my child.	☐1	☐1	☐3	☐4	☐5
D. Having my child leaves little time and flexibility in my life	☐1	☐1	☐3	☐4	☐5
E. Having my child has been a financial burden	☐1	☐1	☐3	☐4	☐5
F. It is difficult to balance different responsibilities because of my child.	☐1	☐1	☐3	☐4	☐5

[ASK S22 ONLY OF FEMALE RESPONDENTS]

S22. Are you currently pregnant? Yes.......... ☐1 No............ ☐2

S23. Which of the following best describes how often you usually drink alcohol?

1. Never.. ☐1 **Go to S26**
2. Less than once a month.. ☐2
3. 1-2 times a month .... ☐3
4. 1-2 times a week... ☐4
5. 3-4 times a week... ☐5
6. 5-6 times a week... ☐6
7. Every day .... ☐7

If currently drink alcohol between everyday and 1-2 times a week ask:

S24. And in an average week, how many pints of beer/cider, glasses of wine, measures of spirit, and bottles of alcopops would you drink?

(a) Pints of Beer/Cider ___ **(b) Glasses of Wine** ___

(c) Measures of Spirits ___ **(d)Bottles of alcopops** ___

For the following questions please consider that 1 drink = ½ pint of beer or 1 glass of wine or 1 single spirits

[ASK S25a ONLY OF FEMALE RESPONDENTS]

S25a. How often do you have 6 or more alcoholic drinks on one occasion?

Never	Less than monthly	Monthly	Weekly	Daily or almost daily
☐1	☐2	☐3	☐4	☐5

[ASK S25b ONLY OF MALE RESPONDENTS]

S25b. How often do you have 8 or more alcoholic drinks on one occasion?

Never	Less than monthly	Monthly	Weekly	Daily or almost daily
☐1	☐2	☐3	☐4	☐5

S25c. How often during the last year have you been unable to remember what happened the night before because you had been drinking?

Never	Less than monthly	Monthly	Weekly	Daily or almost daily
☐1	☐2	☐3	☐4	☐5

S25d. How often during the last year have you failed to do what was expected of you because of drinking?

Never	Less than monthly	Monthly	Weekly	Daily or almost daily
☐1	☐2	☐3	☐4	☐5

S25e. In the last year has a relative or friend, or a doctor or other health worker been concerned about your drinking or suggested you cut down?

No .... ☐1 Yes, on one occasion.......... ☐2 Yes on more than one occasion .... ☐3

S26. Do you currently smoke daily, occasionally or not at all?

Daily ....☐1 Occasionally ....☐2 Not at all ....☐3

S27. About how many cigarettes or cigars do you smoke on average each day?

____________ [Int. enter '0' if less than 1 on average]

S28. Including yourself, how many members of the household smoke? ____N

S29. Do you take any drugs such as cannabis, marijuana, ecstasy, speed, heroin, methadone, crack or cocaine?

Regularly............... ☐1 Occasionally..........☐2 Not at all .... ☐3

S30a. Since the time of the last interview when <child> was 9 years of age, have you been treated by a medical professional for clinical depression, anxiety, 'nerves' or phobias?

Yes…..... ☐1 No……. ☐2

S30b. Are you currently taking medication for clinical depression, anxiety, 'nerves' or phobias?

Yes…... ☐1 No……. ☐2

S31. Listed below are 8 statements about some of the ways you may have felt or behaved. Please indicate how often you have felt this way *during the past week*.

	Rarely or none of the time (less than 1 day)	Some or a little of the time (1-2 days)	Occasionally or a moderate amount of the time (3-4 days)	Most or all of the time (5-7 days)
a. I felt I could not shake off the blues even with help from my family or friends	☐1	☐2	☐3	☐4
b. I felt depressed	☐1	☐2	☐3	☐4
c. I thought my life had been a failure	☐1	☐2	☐3	☐4
d. I felt fearful	☐1	☐2	☐3	☐4
e. My sleep was restless	☐1	☐2	☐3	☐4
f. I felt lonely	☐1	☐2	☐3	☐4
g. I had crying spells	☐1	☐2	☐3	☐4
h. I felt sad	☐1	☐2	☐3	☐4

S32. Have you ever been in trouble with the Gardai or Police (in Ireland or elsewhere) (other than for traffic offences)?

Yes.........☐1 No ....☐2→**Go to S34**

S33. Have you ever been to prison? Yes ....☐1 No .... ☐2

S34. To the best of your knowledge, has <child> ever tried?

	**Yes, and I know about it**	**Probably**	**Possibly**	**I don't think so**
a. Alcohol	☐1	☐2	☐3	☐4
b. Cigarettes	☐1	☐2	☐3	☐4
c. Cannabis/Marijuana	☐1	☐2	☐3	☐4

S35. Have you spoken to your child personally about the following sexual health issues?

	**Yes**	**No**
1. Sex and sexual intercourse	☐1	☐2
2. Sexual feelings, relationships and emotions	☐1	☐2
3. Contraception	☐1	☐2
4. Safer sex/sexually transmitted infections/ venereal diseases	☐1	☐2
5. Sexual orientation (eg. Homosexuality, heterosexuality etc)	☐1	☐2

S36. Can we check, does <child's> biological father/ mother live here with you or elsewhere?

Lives here ☐1 → **Go to S48**
Deceased ☐2 → **Go to S48**
Temporarily lives elsewhere ☐3 → **Go to S48**
Lives elsewhere ☐4 → **Go to S37**

S37. Were you ever married to or did you ever live with <child's> biological father / mother?

Yes, married to ☐1 Yes, lived with ☐2 No ☐3 **Go to S39** Adoptive / Foster parent ☐4 **Go to S48**

S38. What age was the Study Child when you split or separated from their biological father / mother?

____

S39. Do you have a formal or informal parenting arrangement regarding <child> and where he / she lives?

Formal ☐1 Informal ☐2 No parenting arrangement ☐3

S40. Briefly describe that arrangement

____

____

S41. How did you arrive at that arrangement?

Court imposed arrangements ☐1
Formal negotiated arrangements other than legal (e.g. counsellor) ☐2
Mutual agreement with no third party negotiator ☐3

S42. How far does <child's> biological father / mother live from here?

Within ½ hour's drive from here ☐1
Between ½ and 1 hour's drive from here ☐2
More than 1 hour's drive from here ☐3
Outside the country ☐4

S43. How often does <child> have contact with his / her biological father / mother?

Daily ☐1
More than once a week ☐2
Once a week ☐3
Every second week / weekend ☐4
Monthly ☐5
Less than once a month ☐6
No contact ☐7

S44. Does <child's> biological father / mother make ANY financial contribution to your household and the maintenance of <child>? Include any form of financial support such as rent, mortgage, direct maintenance payment etc.

No, he/she never makes any payment ☐1
Yes, he/she makes a regular payment ☐2
Yes, he/she makes payments from time to time ☐3

S45. How often do you talk to <child's> biological father/ mother about <child>?

Every day	Several times a week	About once a week	A few times a month	Several times a year	Never
☐1	☐2	☐3	☐4	☐5	☐6

S46. How well do you get on with <child's> biological father/ mother? Would you say your relationship is?

Very positive	Positive	Neither positive nor negative	Somewhat negative	Very negative
☐1	☐2	☐3	☐4	☐5

S47. We would like to send a short questionnaire to <child's> biological father/ mother. We would be happy to show you the content of this questionnaire before we send it. Would you be able to provide us with contact details for <child's> biological father/ mother?

Yes ☐1 → **Please give contact details to interviewer**
No, I do not wish other parent to be contacted ☐2
No, I do not have contact details for other parent ☐3

THANK YOU VERY MUCH FOR TAKING PART IN THE *GROWING UP IN IRELAND* PROJECT.

PRIMARY CAREGIVER TWIN QUESTIONNAIRE

The Economic and Social Research Institute
Whitaker Square
Sir John Rogerson's Quay
Dublin 2
Ph: 01-863 2000 Fax 01-863 2100

An Roinn Leanaí agus Gnóthaí Óige
Department of Children and Youth Affairs

University of Dublin
Trinity College
College Green
Dublin 2

GROWING UP IN IRELAND – the national longitudinal study of children
STRICTLY CONFIDENTIAL

PRIMARY CAREGIVER QUESTIONNAIRE – 13-year

TWIN SUPPLEMENT

AREA ☐☐☐☐ **HOUSEHOLD** ☐☐☐

Interviewer Name___________________________ **Interviewer Number** ☐☐☐☐☐

Date ____ ______ ______
Day month year

Child's Name: __________________________ **[1st Name Only]**

[Interviewer: please record, height and weight of the Study Twin below:]

Height: __________ **cms**

Weight: __________ **kgs**

Now I would like to ask you a few questions regarding the Child's health.

A. CHILD'S HEALTH

A1. [Card A1] In general, how would you describe <child's> health in the past year?

Very healthy, no problems .... ☐ 1
Healthy, but a few minor problems .... ☐ 2
Sometimes quite ill .... ☐ 3
Almost always unwell .... ☐ 4

A2. Does <child> have any on-going chronic <u>physical or mental health problem, illness or disability?</u>

Yes .... ☐ 1 No .... ☐ 2

A3. What is the nature of this problem, illness or disability? Please describe as fully as possible.
[Int: Please record <u>diagnosis</u>, <u>not symptoms</u> of the problem]
____
____

A4. Has this problem, illness or disability been diagnosed by a medical professional?

Yes............... ☐ 1 No .... ☐ 2

A5. Since when has <child> had this problem, illness or disability? ______(mth) ______(year)

A6. Is <child> hampered in his/her daily activities by this problem, illness or disability?

Yes, severely .... ☐ 1 Yes, to some extent .... ☐ 2 No........... ☐ 3

A7. In the past year has <child> had any periods when there was wheezing with whistling on his/her chest when he/she breathed?

Yes .... ☐1 No........................ ☐2

A8. How many separate episodes/bouts of wheezing with whistling on his/her chest has <child> had in the past 12 months? _______ N

A9. Has <child> been prescribed medication for this condition (including inhaler, antibiotics, nebuliser) over the last 12 months?

Yes .... ☐1 No........................ ☐2

A10a. Has <child> received a course of antibiotics in the past 12 months?

Yes .... ☐1 No........................ ☐2

A10b. In total, how many courses of antibiotics has <child> received in the past 12 months?

______ **N**

A11. Most children have accidents at some time. In the last 12 months has <child> had an accident or injury that required hospital treatment or admission?

Yes .... ☐1 No .... ☐2

A12. How many separate accidents has <child> ever had that required hospital treatment or admission?
_______________ accidents

A13. How many of these accidents involved bone fractures or breaks? __________________

A14. About how many nights has <child> spent in hospital over his/her lifetime? (Exclude at time of birth)
[INTERVIEWER: IF NONE, ENTER '0' – DO NOT LEAVE BLANK] ________nights

A15. In the last 12 months how many visits has <child> made to the A&E (Accident and Emergency) department of a hospital? [INTERVIEWER: IF 'NONE' ENTER '0' DO NOT LEAVE BLANK] ________ visits

A16. [Card A16] In the last 12 months, how many times have you seen, or talked on the telephone with any of the following about the <child's> physical, emotional or mental health? [Int. if 'none' write '0' do not leave blank]

	N times	Don't know	Refused
A. A general practitioner (GP)	________	☐3	☐4
B. A practice nurse	________	☐3	☐4
C. Another medical doctor e.g. in a hospital	________	☐3	☐4
D. Other professional, psychologist, psychiatrist, counsellor etc	________	☐3	☐4
E. A social worker	________	☐3	☐4

A17. Was there any time during the past 12 months when <child> really needed to consult a GP or specialist but did not?

Yes, there was at least one occasion ...... ☐1 No, there was no such occasion......... ☐2

A18. [Card A18] What was the main reason for not consulting a GP or specialist?

a) You couldn't afford to pay .... ☐1
b) The necessary medical care wasn't available or accessible to you .... ☐2
c) You could not take time off work to visit the doctor with <child> .... ☐3
d) You wanted to wait and see if the problem got better .... ☐4
e) Child refused / fear of doctor .... ☐5
f) Child is still on the waiting list .... ☐6
g) Too far to travel/no means of transport .... ☐7
h) Other (specify) .... ☐8

A19. [Card A19] Which of the following best describes how regularly <child> visits the dentist?

At least once a year ☐1
Once every two years ☐2
Once every three years ☐3
Only when there is a problem ☐4
Never/almost never ☐5

A20. Has <child> ever had:

	Yes	No
(a) Any permanent / secondary teeth filled?	☐1	☐2
(b) Any permanent / secondary teeth pulled?	☐1	☐2

A21. Was there any time during the past 12 months when <child> really needed to consult a dentist but did not?

Yes, there was at least one occasion ☐1 No, there was no such occasion ☐2

A22. [Card A22] What was the main reason for not consulting the dentist?

a) You couldn't afford to pay ☐1
b) The necessary medical care wasn't available or accessible to you ☐2
c) You could not take time off work to visit the dentist with <child> ☐3
d) You wanted to wait and see if the problem got better ☐4
e) Child refused / fear of dentist ☐5
f) Child is still on the waiting list ☐6
g) Too far to travel/no means of transport ☐7
h) Other (specify) ☐8

A23. Does <child> usually have breakfast at home before going to school?

Yes ☐1 No ☐2

A24. [Card A24] Which of these best describes <child's> weight?
[INT: ASK THE RESPONDENT TO USE THE CODES 1-8 AS ON THE CARD IF CHILD IS PRESENT AT TIME OF INTERVIEW]

Very underweight ☐1
Moderately underweight ☐2
Slightly underweight ☐3
About the right weight ☐4
Slightly overweight ☐5
Moderately overweight ☐6
Very overweight. ☐7
Don't know ☐8

A25. [Card A25] How far away is <child's> school from your home (one-way distance)?

Less than ½mile (less than 1km) ☐1
½ to less than 1 mile (1 - less than 2km) ☐2
1-5 miles (2 - less than 8km) ☐3
More than 5 miles away (8km or more) ☐4
Attends boarding school ☐5
Not applicable ☐6

A26. [Card A26] How does <child> usually go to school?

1. He/she walks ☐1
2. By public transport ☐2
3. School bus/coach ☐3
4. By car ☐4
5. Rides a bicycle ☐5
6. Other (please describe) ☐6
7. Not applicable ☐7

B. CHILD'S EMOTIONAL HEALTH AND WELL-BEING

Now I'd like to ask some questions on the Child's emotional health and well-being.

B1. [Card B1] Looking at Card B1, has <child> experienced any of the following since we last interviewed you when he/ she was nine:

[INT: ASK THE RESPONDENT TO USE CODES A-P AS ON THE CARD IF CHILD IS PRESENT AT TIME OF INTERVIEW]

A. Death of a parent .... ☐1
B. Death of a close family member (other than a parent) please specify. ☐2 ______________________
C. Death of close friend .... ☐3
D. Divorce/separation of parents .... ☐4
E. Moving house within Ireland .... ☐5
F. Moving country .... ☐6
G. Stay in foster home/ residential care .... ☐7
H. Serious illness/injury .... ☐8
I. Serious illness/injury of a family member .... ☐9
J. Drug taking/alcoholism in the immediate family .... ☐10
K. Mental disorder in immediate family .... ☐11
L. Your house being broken into .... ☐12
M. Conflict between parents .... ☐13
N. Parent in prison .... ☐14
O. Other disturbing event (please specify) .... ☐15 ______________________
P. None of the above .... ☐16

B2. [Card B2] Listed on Card B2, is a set of statements which could be used to describe <child's> behaviour. For each item, please indicate whether it is Not True, Somewhat True or Certainly True. It would help us if you answered all items as best you can even if you are not absolutely certain. Please give answers on the basis of <child's> behaviour over the last six months. Use answers 1, 2 or 3 as on the card if you like.

	Not True	Somewhat True	Certainly True
A. Considerate of other people's feelings	☐1	☐2	☐3
B. Restless, overactive, cannot stay still for long	☐1	☐2	☐3
C. Often complains of headaches, stomach aches or sickness	☐1	☐2	☐3
D. Shares readily with other children (treats, toys, pencils etc.)	☐1	☐2	☐3
E. Often has temper tantrums or hot tempers	☐1	☐2	☐3
F. Rather solitary, tends to play alone	☐1	☐2	☐3
G. Generally obedient, usually does what adults request	☐1	☐2	☐3
H. Many worries, often seems worried	☐1	☐2	☐3
I. Helpful if someone is hurt, upset or feeling ill	☐1	☐2	☐3
J. Constantly fidgeting or squirming	☐1	☐2	☐3
K. Has at least one good friend	☐1	☐2	☐3
L. Often fights with other children or bullies them	☐1	☐2	☐3
M. Often unhappy, down-hearted or tearful	☐1	☐2	☐3
N. Generally liked by other children	☐1	☐2	☐3
O. Easily distracted, concentration wanders	☐1	☐2	☐3
P. Nervous or clingy in new situations, easily loses confidence	☐1	☐2	☐3
Q. Kind to younger children	☐1	☐2	☐3
R. Often lies or cheats	☐1	☐2	☐3
S. Picked on or bullied by other children	☐1	☐2	☐3
T. Often volunteers to help others (parents, teachers, other children)	☐1	☐2	☐3
U. Thinks things out before acting	☐1	☐2	☐3
V. Steals from home, school or elsewhere	☐1	☐2	☐3
W. Gets on better with adults than with other children	☐1	☐2	☐3
X. Many fears, easily scared	☐1	☐2	☐3
Y. Sees tasks through to the end, good attention span	☐1	☐2	☐3

B3. [Card B3] Listed on card B3 are a number of personality traits that may or may not apply to your child. Please indicate the extent to which you agree or disagree with that statement. You should rate the extent to which the pair of traits applies to him/her, even if one characteristic applies more strongly than the other.

I see my child as:

	Disagree strongly	Disagree moderately	Disagree a little	Neither agree nor disagree	Agree a little	Agree moderately	Agree strongly
Extroverted, enthusiastic	☐1	☐2	☐3	☐4	☐5	☐6	☐7
Critical, quarrelsome	☐1	☐2	☐3	☐4	☐5	☐6	☐7
Dependable, self-disciplined	☐1	☐2	☐3	☐4	☐5	☐6	☐7
Anxious, easily upset	☐1	☐2	☐3	☐4	☐5	☐6	☐7
Open to new experiences, complex	☐1	☐2	☐3	☐4	☐5	☐6	☐7
Reserved, quiet	☐1	☐2	☐3	☐4	☐5	☐6	☐7
Sympathetic, warm	☐1	☐2	☐3	☐4	☐5	☐6	☐7
Disorganized, careless	☐1	☐2	☐3	☐4	☐5	☐6	☐7
Calm, emotionally stable	☐1	☐2	☐3	☐4	☐5	☐6	☐7
Conventional, uncreative	☐1	☐2	☐3	☐4	☐5	☐6	☐7

Now I'd like to ask you some questions about the Child's education

C. CHILD'S EDUCATION – PAST AND CURRENT

C1a. What class did / will <child> start in September 2011?

5th Class	☐1	Go to C1b
6th Class	☐2	Go to C1b
First Year	☐3	Go to C1b
Second Year	☐4	Go to C1b
Child is being home schooled.	☐5	Go to C7
Child attends a special school	☐6	Go to C1b
Child no longer attends school.	☐7	Go to C10

C1b. What school does <child> attend / will attend from September 2011?

Name of school: ___________________________________

Full address of school: ____

____

C1c. In what year did <child> start primary school? September 20_ _

C1d. [Card C1d] How would you describe <child's> current base class – the one they will be in from September 2011? (Tick one box)

Special class	☐1
Class which is mixed ability / randomly allocated	☐2
Higher stream class in streamed school	☐3
Middle stream class in streamed school	☐4
Lower stream class in streamed school	☐5
Not sure / don't know	☐6

[ONLY ASK IF CHILD IS IN 2nd YEAR AT C1a, THEN GO TO C5]

C2. [Card C2] Here are some views about how your child settled into their new school. There are no right or wrong answers. For each statement please tick ONE BOX ONLY to show whether you agree or disagree with these views.

	Strongly agree	Agree	Neither agree nor disagree	Disagree	Strongly disagree
My child settled well into secondary school.	☐1	☐2	☐3	☐4	☐5
My child missed old friends from primary school.	☐1	☐2	☐3	☐4	☐5
My child was anxious about making new friends.	☐1	☐2	☐3	☐4	☐5
My child coped well with the school work.	☐1	☐2	☐3	☐4	☐5
My child made new friends	☐1	☐2	☐3	☐4	☐5
My child is involved in extra-curricular activities.	☐1	☐2	☐3	☐4	☐5
My child gets too much homework at this school.	☐1	☐2	☐3	☐4	☐5

[ONLY ASK IF CHILD IS IN 1st YEAR AT C1a, THEN GO TO C4b]

C3. [Card C3] Here are some views about how your child is settling into their new school. There are no right or wrong answers. For each statement please tick ONE BOX ONLY to show whether you agree or disagree with these views.

	Strongly agree	Agree	Neither agree nor disagree	Disagree	Strongly disagree
My child is settling in well into secondary school	☐1	☐2	☐3	☐4	☐5
My child misses old friends from primary school.	☐1	☐2	☐3	☐4	☐5
My child is anxious about making new friends	☐1	☐2	☐3	☐4	☐5
My child is coping well with the school work.	☐1	☐2	☐3	☐4	☐5
My child has made new friends	☐1	☐2	☐3	☐4	☐5
My child is involved in extra-curricular activities.	☐1	☐2	☐3	☐4	☐5
My child gets too much homework at this school.	☐1	☐2	☐3	☐4	☐5

[ONLY ASK IF CHILD IS IN 5th / 6th CLASS AT C1a, THEN GO TO C5]

C4a. [Card C4a] If your child is still in fifth / sixth class for each statement please tick ONE BOX ONLY to show whether you agree or disagree with these views.

	Strongly agree	Agree	Neither agree nor disagree	Disagree	Strongly disagree
My child is excited about starting secondary school	☐1	☐2	☐3	☐4	☐5
My child is looking forward to making new friends	☐1	☐2	☐3	☐4	☐5
My child is nervous about moving to a new school	☐1	☐2	☐3	☐4	☐5

C4b. Has <child> attended an Open Day at his/her new school Yes ☐1 No ☐2

C5. [Card C5] Over the last 12 months, have you had any contact with the school? (Please include contact you have had with the child's current school or any other school the child attended in the last 12 months) [Please tick 'Yes' or 'No' to each.]

	Yes	No
A. You have attended a parent-teacher meeting	☐1	☐2
B. You have attended a school concert, play or other event (such as sports day)	☐1	☐2
C. You have been to see the principal or another teacher about child's behaviour or school performance	☐1	☐2
D. You have spoken to the principal or another teacher on the phone about child's behaviour or school performance	☐1	☐2

C6a. [Card C6a] Looking at Card C6a, during the last 12 months, about how many days was <child> absent from school for any reason? (Only include days the child was absent when the school was open e.g. do not include days missed because of the school being closed due to bad weather).

- 0 days ☐1
- 1 - 3 days ☐2
- 4 to 6 days ☐3
- 7 to 10 days ☐4
- 11 to 20 days ☐5
- More than 20 days ☐6
- Not in school last year ☐7

C6b. [Card C6b] Looking at Card C6b, what was the main reason for <child> being absent from school?

- Health reasons (illness or injuries) ☐1
- Problems with transportation ☐2
- Problems with the weather ☐3
- A family vacation ☐4
- Refused to go to school ☐5
- A fear of school (school phobia) ☐6
- Suspended from school ☐7
- A problem with a teacher ☐8
- A problem with children at school ☐9
- Difficulties with childcare arrangements ☐10
- Family crisis ☐11
- Child has left school ☐12
- Other (specify) ______________ ☐13

C7. [Card C7] Looking at Card C7, how much time does <child> usually spend doing homework on a weekday during term time?

- 0 to 30 minutes ☐1
- 31 minutes to less than one hour ☐2
- 1 to less than 1.5 hours ☐3
- 1.5 to less than 2 hours ☐4
- 2 to less than 3 hours ☐5
- 3 to less than 4 hours ☐6
- 4 hours or more ☐7
- Doesn't get homework ☐8 Go to C9

C8a. How often do you or your spouse/partner provide help with <child>'s homework? Would you say…[INT: READ OUT]

Always/ Nearly Always	Regularly	Now and Again	Rarely	Never	Never gets homework
☐1	☐2	☐3	☐4	☐5	☐6

C8b. Why is that?

Child doesn't need help	I / We don't have time	I / We are not able to help	Child doesn't want help	Someone else helps
☐1	☐2	☐3	☐4	☐5

C9. [Card C9] Looking at Card C9, taking everything into account, how far do you expect <child> will go in his/her education or training?

Junior Certificate or equivalent ☐1
Leaving Certificate or equivalent ☐2
An apprenticeship or trade ☐3
Diploma/Certificate ☐4
Degree ☐5
Postgraduate/higher degree ☐6
Don't know ☐7

C10. About how many close friends does <child> have?

None....... ☐1   1 .... ☐2   2 or 3......... ☐3   4 or 5 .... ☐4   6 or more ...... ☐5

C11. To your knowledge, has <child> been a victim of bullying in the last 3 months?

Yes............ ☐1   No .... ☐2

C12. [Card C12] Looking at Card C12, what form did the bullying take? [Int. tick all that apply]

A. Physical bullying ☐1
B. Verbal bullying (name calling, hurtful slagging) ☐2
C. Electronic (phone messaging, emails, Facebook, etc) ☐3
D. Graffiti/pinning up notes/passing notes in class ☐4
E. Taking /damaging personal possessions ☐5
F. Sexual comments ☐6
G. Exclusion (being left out). ☐7
H. Gossip, spreading rumours ☐8
I. Threatened or forced to do things s/he didn't want to ☐9
J. Other (specify)________________________ ☐10

C13. [Card C13] How often did the bullying take place?

A. Once or twice ☐1
B. 2 or 3 times a month ☐2
C. About once a week ☐3
D. Several times a week ☐4

C14. Did this upset your child?

A. A lot ☐1
B. A little ☐2
C. Not at all ☐3

C15. [Card C15] Does <child> have any of the following conditions or disabilities? [Tick all that apply]

a. Physical disability or visual or hearing impairment ☐1
b. Specific learning disability (e.g. Dyslexia, Dyscalculia, Dyspraxia) ☐2
c. General learning disabilities (Mild, Moderate, Severe/Profound) ☐3
d. Autism Spectrum Disorders (e.g. Austism, Aspergers syndrome) ☐4
e. Emotional or behavioural disorders (e.g. ADHD (Attention Deficit Hyperactivity Disorder)/ ADD) ☐5
f. Mental health difficulty ☐6
g. Speech or language difficulty (including speech impediment) ☐7
h. Assessed Syndrome (e.g. Down Syndrome, Tourettes Syndrome) ☐8
i. Slow progress (reasons unclear) ☐9
j. Other (please specify) ☐10
k. None of the above ☐11 → **Go to C24**

C16. Has this condition or disability been diagnosed by a medical professional?

Yes .... ☐1 No .... ☐2 Awaiting Consultation .... ☐3

C17. What age was <child> when this condition or disability was first diagnosed? ____________ years

[INT: If condition or disability was diagnosed at time of birth, code as '0']

Ask C18 only of respondents who ticked yes at C15e

C18. Has <child> been prescribed any medication for this condition (e.g. Ritalin, Abilify etc…)?

Yes .... ☐1 No .... ☐2

Ask C19 only of respondents who ticked yes at C15f

C19. Has <child> been prescribed any medication for this condition?

Yes .... ☐1 No .... ☐2

Ask C20 only of respondents who ticked yes at C15G

C20. [Card C20] In which areas does <child> have difficulties? What speech problems does <child> have?
[TICK ALL THAT APPLY]

A. Reluctant to speak .... ☐1
B. Speech not clear to the family .... ☐2
C. Speech not clear to others .... ☐3
D. Speech is developing slowly .... ☐4
E. Difficulty finding words .... ☐5
F. Difficulty putting words together .... ☐6
G. Voice sounds unusual .... ☐7
H. Stutters, stammers .... ☐8
I. Lisp or difficulty pronouncing certain letter combinations .... ☐9
J. Other (please specify) .... ☐10
K. Don't know .... ☐99

C21. [Card C21] Please indicate if <child> receives support from any of the following <u>IN SCHOOL</u>
[Tick all that apply]
In School

Resource Teaching/ Learning Support .... ☐1
Special Needs Assistant .... ☐2
Technical Assistance .... ☐3
Visiting Teacher .... ☐4
Transport Service .... ☐5
Speech and Language Therapist .... ☐6
Behavioural Management Programme .... ☐7
School psychologist .... ☐8
National Educational Psychological Service .... ☐9
Other (please specify)………………. .... ☐10
Doesn't receive any supports .... ☐11

C22. [Card C22] Please indicate if <child> receives support from any of the following <u>OUTSIDE SCHOOL</u>
[Tick all that apply]
Outside School

Speech and Language Therapist .... ☐1
Occupational Therapist .... ☐2
Physiotherapist .... ☐3
Psychologist .... ☐4
Psychiatrist .... ☐5
Extra tuition/private tuition .... ☐6
Other (please specify) ____________________ ..... ☐7
Doesn't receive any supports .... ☐8

C23. In general, how adequate are the supports <child> receives for this/these condition(s) or disability(ies)?

Barely adequate .... ☐1
Adequate .... ☐2
Excellent....................................... ☐3
Doesn't receive any supports......... ☐4

C24. How many books does <child> have access to in the home? Would you say…[INT: READ OUT]

None .... ☐1
1 to 10 .... ☐2
11 to 30 .... ☐3
31 to 50 .... ☐4
51 to 100 .... ☐5
More than 100 .... ☐6

C25. [Card C25] On a normal weekday, during term-time, about how much time does <child> spend using the computer. Please include time before school as well as time after school. DO NOT include time spent using computers in school.

None	☐1	3 hours to less than 5 hours	☐4
Less than an hour	☐2	5 hours to less than 7 hours	☐5
1 hour to less than 3 hours	☐3	7 hours or more	☐6

C26. [Card C26] On a typical weekday, who, if anyone, minds <child> between the time they finish school and 6pm in the evening? (Tick one only; if more than one indicate the type of care where <child> spends MOST time or is the most frequently used)

- They come home and take care of themselves ☐1
- Minded at home by an older sibling ☐2
- Minded at home by you or your spouse/partner ☐3
- Minded at home by a relative ☐4
- Minded at home by another adult (not a relative) ☐5
- Attend an after-school program/club ☐6
- Hang out with friends ☐7
- Other (please specify) ☐8

D: FAMILY CONTEXT

Now some questions about your relationship with <Child>.

D1. [Show Card D1] Looking at Card D1, I am going to read out some statements about the relationship between you and your child. Please listen to each statement and describe the degree to which each of the following statements currently applies.

	Definitely does not apply	Not really	Neutral, not sure	Applies somewhat	Definitely applies
A. I share an affectionate, warm relationship with my child.	☐1	☐2	☐3	☐4	☐5
B. My child and I always seem to be struggling with each other.	☐1	☐2	☐3	☐4	☐5
C. If upset, my child will seek comfort from me.	☐1	☐2	☐3	☐4	☐5
D. My child is uncomfortable with physical affection or touch from me.	☐1	☐2	☐3	☐4	☐5
E. My child values his/her relationship with me.	☐1	☐2	☐3	☐4	☐5
F. When I praise my child, he/she beams with pride.	☐1	☐2	☐3	☐4	☐5
G. My child spontaneously shares information about himself/herself	☐1	☐2	☐3	☐4	☐5
H. My child easily becomes angry at me.	☐1	☐2	☐3	☐4	☐5
I. It is easy to be in tune with what my child is feeling.	☐1	☐2	☐3	☐4	☐5
J. My child remains angry or is resistant after being disciplined	☐1	☐2	☐3	☐4	☐5
K. Dealing with my child drains my energy.	☐1	☐2	☐3	☐4	☐5
L. When my child is in a bad mood, I know we're in for a long and difficult day.	☐1	☐2	☐3	☐4	☐5
M. My child's feelings toward me can be unpredictable or can change suddenly.	☐1	☐2	☐3	☐4	☐5
N. My child is sneaky or manipulative with me.	☐1	☐2	☐3	☐4	☐5
O. My child openly shares his/her feelings and experiences with me.	☐1	☐2	☐3	☐4	☐5

D2. [Card D2] The following are some questions on your knowledge of what <child> does in his/her free time, where he/she goes, and who he/she has as friends.

	**Almost never or never**	**Not very often**	**Sometimes**	**Often**	**Almost always or always**	**N/A**
A. Do you know what <child> does with his/her free time.	☐1	☐2	☐3	☐4	☐5	☐6
B. Do you know who he/she has as friends during his/her free time.	☐1	☐2	☐3	☐4	☐5	☐6
C. Do you usually know what type of homework he/she has.	☐1	☐2	☐3	☐4	☐5	☐6
D. Do you know what he/she spends his/her money on	☐1	☐2	☐3	☐4	☐5	☐6
E. Do you know when he/she has a test or homework due at school.	☐1	☐2	☐3	☐4	☐5	☐6
F. Do you know how he/she does in different subjects at school.	☐1	☐2	☐3	☐4	☐5	☐6
G. Do you know where he/she goes when out at night with friends	☐1	☐2	☐3	☐4	☐5	☐6
H. Do you know where he/she goes and what he/she does after school.	☐1	☐2	☐3	☐4	☐5	☐6
I. How often in the last month have you had no idea where he/she was.	☐1	☐2	☐3	☐4	☐5	☐6

D3. [CARD D3] The following are some questions about how much <child> actually tells you about what he/she is doing, without being asked.

	Almost never or never	Not very often always	Sometimes	Often	Almost always or	N/A
A. Does he/she spontaneously tell you about his/her friends.	☐1	☐2	☐3	☐4	☐5	☐6
B. Does he/she want to tell you about school (how subjects are going; relationships with teachers etc).	☐1	☐2	☐3	☐4	☐5	☐6
C. Does he/she keep a lot of secrets from you about what he/she is doing in his/her spare time	☐1	☐2	☐3	☐4	☐5	☐6
D. Does he/she hide a lot from you about what he/she is doing during nights and weekends	☐1	☐2	☐3	☐4	☐5	☐6
E. Does he/she like to tell you what he/she has been doing and where he/she went when out for the evening..	☐1	☐2	☐3	☐4	☐5	☐6

D4. [Show Card D4] Looking at Card D4, now I'd like to ask you about the time <child> spends with you including times when others are present.   How many days per week do you:

	Every day / 7 days per week	3 to 6 days per week	1 to 2 days per week	1 to 2 times per month	Rarely or never
A. Sit down to eat together	☐1	☐2	☐3	☐4	☐5
B. Play sports, cards or games together	☐1	☐2	☐3	☐4	☐5
C. Talk about things together	☐1	☐2	☐3	☐4	☐5
D. Do household activities together (e.g. gardening, cooking, cleaning, etc)	☐1	☐2	☐3	☐4	☐5
E. Go on an outing together (e.g. going to the cinema, theatre, walking, shopping)	☐1	☐2	☐3	☐4	☐5

D5. [Show Card D5] Looking at Card D5, how often does <child> get together with, see or spend time with the following people (excluding those living in your home)

	Quite a lot	Now and again	Rarely	Live Abroad	Doesn't have
A. Grandparents	☐1	☐2	☐3	☐4	☐5
B. Uncles/Aunts	☐1	☐2	☐3	☐4	☐5
C. Cousins	☐1	☐2	☐3	☐4	☐5
D. Other family members/ close family friends	☐1	☐2	☐3	☐4	☐5

D6. [Show Card D6] I would now like to ask some questions about <child's> behaviour over the last 12 months. Please tell me the extent to which the following statements apply:

	Not at all	Once	2-5 times	6 or more times
A. Often started fights or bullies, threatens or intimidates others	☐1	☐2	☐3	☐4
B. Has used a weapon that could cause serious physical harm to others (eg, a bat, brick, broken bottle, knife)	☐1	☐2	☐3	☐4
C. Has been physically cruel to other people	☐1	☐2	☐3	☐4
D. Has been physically cruel to animals	☐1	☐2	☐3	☐4
E. Deliberately destroyed or damaged property	☐1	☐2	☐3	☐4
F. Has broken into someone else's house, building or car	☐1	☐2	☐3	☐4
G. Has lied to obtain goods or favours (i.e., 'cons' others)	☐1	☐2	☐3	☐4
H. Has stolen items of value without confronting a victim (e.g., shoplifting, but without breaking and entering)	☐1	☐2	☐3	☐4
I. Has stayed out at night despite parental prohibitions	☐1	☐2	☐3	☐4
J. Has run away from home overnight at least twice while living in parental home (or once for a lengthy period)	☐1	☐2	☐3	☐4
K. Has truanted from school	☐1	☐2	☐3	☐4

SECONDARY CAREGIVER TWIN QUESTIONNAIRE

The Economic and Social Research Institute
Whitaker Square
Sir John Rogerson's Quay
Dublin 2
Ph: 01-863 2000 Fax 01-863 2100

An Roinn Leanaí agus Gnóthaí Óige
Department of Children and Youth Affairs

University of Dublin
Trinity College
College Green
Dublin 2

TRINITY COLLEGE DUBLIN

GROWING UP IN IRELAND – the national longitudinal study of children
STRICTLY CONFIDENTIAL
SECONDARY CAREGIVER QUESTIONNAIRE – 13-Year Main

TWIN SUPPLEMENT

AREA ☐☐☐☐ **HOUSEHOLD** ☐☐☐

Interviewer Name____________________________ **Interviewer Number** ☐☐☐☐☐

Date ____ ______ ______
day month year

A: FAMILY CONTEXT

Now I'd like to ask you some general questions about your family as a whole.

A1. [Card A1] I am going to read out some statements about the relationship between you and your child. Please listen to each statement and describe the degree to which each of the following statements currently applies.

	Definitely does not apply	Not really	Neutral, not sure	Applies somewhat	Definitely applies
A. I share an affectionate, warm relationship with my child	☐1	☐2	☐3	☐4	☐5
B. My child and I always seem to be struggling with each other	☐1	☐2	☐3	☐4	☐5
C. If upset, my child will seek comfort from me.	☐1	☐2	☐3	☐4	☐5
D. My child is uncomfortable with physical affection or touch from me.	☐1	☐2	☐3	☐4	☐5
E. My child values his/her relationship with me.	☐1	☐2	☐3	☐4	☐5
F. When I praise my child, he/she beams with pride.	☐1	☐2	☐3	☐4	☐5
G. My child spontaneously shares information about himself/herself	☐1	☐2	☐3	☐4	☐5
H. My child easily becomes angry at me.	☐1	☐2	☐3	☐4	☐5
I. It is easy to be in tune with what my child is feeling.	☐1	☐2	☐3	☐4	☐5
J. My child remains angry or is resistant after being disciplined	☐1	☐2	☐3	☐4	☐5
K. Dealing with my child drains my energy.	☐1	☐2	☐3	☐4	☐5
L. When my child is in a bad mood, I know we're in for a long and difficult day	☐1	☐2	☐3	☐4	☐5
M. My child's feelings toward me can be unpredictable or can change suddenly	☐1	☐2	☐3	☐4	☐5
N. My child is sneaky or manipulative with me.	☐1	☐2	☐3	☐4	☐5
O. My child openly shares his/her feelings and experiences with me.	☐1	☐2	☐3	☐4	☐5

A2. [Card A2] The following are some questions on your knowledge of what <child> does in his/her free time, where he/she goes, and who he/she has as friends.

	Almost never or never	Not very often	Sometimes	Often	Almost always or always	N/A
A. Do you know what <Study Child> does with his/her free time.	☐1	☐2	☐3	☐4	☐5	☐6
B. Do you know who he/she has as friends during his/her free time.	☐1	☐2	☐3	☐4	☐5	☐6
C. Do you usually know what type of homework he/she has.	☐1	☐2	☐3	☐4	☐5	☐6
D. Do you know what he/she spends his/her money on	☐1	☐2	☐3	☐4	☐5	☐6
E. Do you know when he/she has a test or homework due at school.	☐1	☐2	☐3	☐4	☐5	☐6
F. Do you know how he/she does in different subjects at school.	☐1	☐2	☐3	☐4	☐5	☐6
G. Do you know where he/she goes when out at night with friends	☐1	☐2	☐3	☐4	☐5	☐6
H. Do you know where he/she goes and what he/she does after school.	☐1	☐2	☐3	☐4	☐5	☐6
I. How often in the last month have you had no idea where he/she was.	☐1	☐2	☐3	☐4	☐5	☐6

A3. [CARD A3] The following are some questions about how much <child> actually tells you about what he/she is doing, without being asked.

	Almost never or never	Not very often	Sometimes	Often	Almost always or always	N/A
A. Does he/she spontaneously tell you about his/her friends.	☐1	☐2	☐3	☐4	☐5	☐6
B. Does he/she want to tell you about school (how subjects are going; relationships with teachers etc).	☐1	☐2	☐3	☐4	☐5	☐6
C. Does he/she keep a lot of secrets from you about what he/she is doing in his/her spare time	☐1	☐2	☐3	☐4	☐5	☐6
D. Does he/she hide a lot from you about what he/she is doing during nights and weekends	☐1	☐2	☐3	☐4	☐5	☐6
E. Does he/she like to tell you what he/she has been doing and where he/she went when out for the evening	☐1	☐2	☐3	☐4	☐5	☐6

A4. [Show Card A4] I'd like to ask you about the time <child> spends with you including times when others are present. How many days per week do you:

	Every day / 7 days per week	3 to 6 days per week	1 to 2 days per week	1 to 2 times per month	Rarely or never
A. Sit down to eat together	☐1	☐2	☐3	☐4	☐5
B. Play sports, cards or games together	☐1	☐2	☐3	☐4	☐5
C. Talk about things together	☐1	☐2	☐3	☐4	☐5
D. Do household activities together (e.g. gardening, cooking, cleaning, etc)	☐1	☐2	☐3	☐4	☐5
E. Go on an outing together (e.g. going to the cinema, theatre, walking, shopping)	☐1	☐2	☐3	☐4	☐5

A5a. Thinking of an average school day, what amount of time in total would you say you spend with the Study Child either alone or with others (this could be watching TV, going shopping etc)

_______**hours** _____**minutes**

A5b. And thinking of an average weekend, what amount of time in total would you say you spend with the Study Child either alone or with others (this could be watching TV, going shopping etc)

_______**hours** _____**minutes**

YOUNG PERSON MAIN QUESTIONNAIRE

The Economic and Social Research Institute
Whitaker Square
Sir John Rogerson's Quay
Dublin 2
Ph: 01-863 2000 Fax 01-863 2100

An Roinn Leanaí agus Gnóthaí Óige
Department of Children and Youth Affairs

University of Dublin
Trinity College
College Green
Dublin 2

GROWING UP IN IRELAND
– the national longitudinal study of children

STRICTLY CONFIDENTIAL

YOUNG PERSON MAIN QUESTIONNAIRE

AREA ☐☐☐☐ **H'HOLD** ☐☐☐

Interviewer Name___________________________ **Interviewer Number** ☐☐☐☐☐

Date ______ ______ ______
day mnth year

Welcome to the *Growing Up in Ireland* study and thank you for helping us by filling in the questionnaires. We want to find out what it is like to be a 13-year-old in Ireland today. Your answers will help to plan things for young people like yourself.

Some of the questions are about you, your school, your family and friends, how you feel and what you like to do. If you feel that there are any questions which you do not wish to answer, then that's OK.

This is not a test and there are no right or wrong answers. Take your time and try to answer each question the way you really think. If you need help, just let the interviewer know.

We will not tell anyone the answers to your questions. But if you tell us something that makes us worried about you, then we might have to tell someone who could help.

How to fill in your answer on the answer booklet

To fill in a question just tick the box with the answer you want to give

Example:

Do you have any pets? Yes ..... ☑ No .... ☐2

Q1a. What school are you in (from September 2011)? Please fill in the school name and address

School name: ____________________

School address: ____________________

Q1b. What class are you in (from September 2011)?

Home schooled .... ☐1 → **Go to Q12**
5th class .... ☐2
6th class .... ☐3
1st year .... ☐4
2nd year .... ☐5
Other class .... ☐6

(5th class / 6th class →)

. What is your favourite subject? ____________________

. What is your least favourite subject? ____________________

Go to Q5x

(1st year / 2nd year / Other class →)

Q2a. Please tick the subjects you are taking from September 2011. For Irish, English and Maths, please tick which level you are studying.

Irish	Higher ☐1	Ordinary ☐2	Foundation ☐3	Not sure yet ☐4	Don't take Irish ☐5
English	Higher ☐1	Ordinary ☐2	Foundation ☐3	Not sure yet ☐4	
Mathematics	Higher ☐1	Ordinary ☐2	Foundation ☐3	Not sure yet ☐4	

History .... ☐1
Geography .... ☐1
French .... ☐1
German .... ☐1
Spanish .... ☐1
Italian .... ☐1
Art, Craft & Design .... ☐1
Music Science (with Local Studies) .... ☐1
Science .... ☐1
Science (with Local Studies) .... ☐1
Home Economics .... ☐1
Materials Technology (Wood) .... ☐1
Metalwork .... ☐1
Technical Graphics .... ☐1
Business Studies .... ☐1
Typewriting .... ☐1
Environmental and Social Studies (ESS) .... ☐1
Technology .... ☐1
Latin .... ☐1
Ancient Greek Classical Studies .... ☐1
Hebrew Studies .... ☐1
Religious Education .... ☐1
Civic, Social and Political Education (CSPE) .... ☐1
Physical Education .... ☐1
Social, Personal and Health Education (SPHE) .... ☐1
Computer Studies .... ☐1
Other – please specify ____________________ ☐1

Q2b. What is your favourite subject? ____________________

Q2c. What is your least favourite subject? ____________________

Q3. How many of your friends from primary school are in your <u>secondary</u> school? [TICK ONE BOX ONLY]

None.......... ☐1 One .... ☐2 Two .... ☐3 Three or more .... ☐4 Still at primary school ... ☐5

Q4. How many of your friends from primary school are in your <u>class</u>? [TICK ONE BOX ONLY]

None.......... ☐1 One .... ☐2 Two .... ☐3 Three or more .... ☐4 Still at primary school ... ☐5

Q5x. How do you feel about school in general? [TICK ONE BOX ONLY]

I like it very much .... ☐1
I like it quite a bit .... ☐2
I like it a bit .... ☐3
I don't like it very much .... ☐4
I hate it .... ☐5

Q5a. In general, how often do the following things happen to you in school? [TICK ONE BOX ON EACH LINE]

	Very often	Often	A few times	Never
You are told by a teacher that your work is good	☐1	☐2	☐3	☐4
You are encouraged to ask questions in class	☐1	☐2	☐3	☐4
A teacher praises you for answering a question	☐1	☐2	☐3	☐4
You are given out to by a teacher because your work is untidy or not done on time	☐1	☐2	☐3	☐4
You are asked questions in class by the teacher	☐1	☐2	☐3	☐4
You are given out to by a teacher for misbehaving in class	☐1	☐2	☐3	☐4

Q5b. In general, thinking about all your subjects and teachers, how regularly do the following take place in your classes? [TICK ONE BOX ON EACH LINE]

	Very regularly	Quite regularly	Now and again	Never or hardly ever
We copy notes from the board	☐1	☐2	☐3	☐4
I can work in a group with other students	☐1	☐2	☐3	☐4
The teacher reads from the textbook	☐1	☐2	☐3	☐4
The teacher uses a CD or DVD in class	☐1	☐2	☐3	☐4
We use computer facilities in class	☐1	☐2	☐3	☐4
The teacher explains things really well	☐1	☐2	☐3	☐4
The teacher does most of the talking	☐1	☐2	☐3	☐4
I can express my opinions in class	☐1	☐2	☐3	☐4
We have projects to do outside class time	☐1	☐2	☐3	☐4
We get homework	☐1	☐2	☐3	☐4

Q6. On average how much time do you spend doing homework on a normal weekday during term-time? [TICK ONE BOX ONLY]

0 to 30 minutes ☐1
31 minutes to less than one hour ☐2
1 to less than 1.5 hours ☐3
1.5 to less than 2 hours ☐4
2 to less than 3 hours ☐5
3 to less than 4 hours ☐6
4 hours or more ☐7
Don't do homework ☐8

Q7. For each of these subjects, please indicate if you find the subject Difficult, OK, Not Difficult or You Don't Take that Subject. [TICK ONE BOX ON EACH LINE]

	Difficult	OK	Not difficult	Don't take
Maths	☐1	☐2	☐3	☐4
Irish	☐1	☐2	☐3	☐4
English	☐1	☐2	☐3	☐4
Science	☐1	☐2	☐3	☐4

Q8. For each of these subjects, please indicate if you find the subject Interesting, OK, Not interesting or you don't take that subject. [TICK ONE BOX ON EACH LINE]

	Interesting	OK	Not interesting	Don't take
Maths	☐1	☐2	☐3	☐4
Irish	☐1	☐2	☐3	☐4
English	☐1	☐2	☐3	☐4
Science	☐1	☐2	☐3	☐4

Q9a. Some students get extra help at school in some subjects. Over the last 12 months have you received any extra help within school in any subject?

Yes ☐1   No ☐2 → Go to Q10

Q9b. If Yes, what subjects did you get extra help in? [TICK ALL THAT APPLY]

English/Reading ☐1   Maths ☐2   Irish ☐3   Other (please specify) ____________

Q10. Over the last 12 months, how often have the following things happened to you? [TICK ONE BOX ON EACH LINE]

	Never	Now & Again	Quite Often	All the time
I was late for school	☐1	☐2	☐3	☐4
I got into trouble for not following school rules	☐1	☐2	☐3	☐4
I skipped classes or mitched.	☐1	☐2	☐3	☐4
I 'messed' in class	☐1	☐2	☐3	☐4
I had to do extra work as punishment (including lines)	☐1	☐2	☐3	☐4
I had to do detention (after school or at lunch-time)	☐1	☐2	☐3	☐4
I was suspended from school	☐1	☐2	☐3	☐4

Q11. How many days were you absent from school in the last 12 months (when the school was open) _____

Q12. What is the highest qualification you expect to get by the time you finish your education? [TICK ONE BOX ONLY]

Junior Cert.. ☐1
Leaving Cert.. ☐2
Certificate or Diploma (including PLC, apprenticeship) .. ☐3
Degree or higher degree .... ☐4

Q13. On a normal weekday during term-time, about how many hours do you spend watching television, videos or DVDs? Please remember to include time before school as well as time after school?

______ hours ______ minutes None ……. ☐1

Q14. On a normal weekday during term-time, about how many hours do you spend reading for pleasure (books, magazines, newspapers, novels, comics)? [DO NOT INCLUDE TIME SPENT READING AT SCHOOL OR DOING HOMEWORK]

______ hours ______ minutes None ……. ☐1

Q15. On a normal weekday, during term-time, about how much time do you spend using the computer? Please include time before school as well as time after school. [DO NOT INCLUDE TIME SPENT USING COMPUTERS IN SCHOOL]

______ hours ______ minutes None ……. ☐1

Q16. On a normal weekday, during term-time, about how much time do you spend playing video games such as Playstation, X-box, Nintendo, etc.?

______ hours ______ minutes None ……. ☐1

Q17. Are any of the following in your bedroom? [TICK 'YES' OR 'NO' FOR EACH]

	Yes	No
Television	☐1	☐2
Computer or laptop	☐1	☐2
Video / DVD player	☐1	☐2
Games console (Playstation, etc)	☐1	☐2

Q18. Do you have your own mobile phone? Yes.......... ☐1 No .... ☐2

Q19. Do you have a computer at home? Yes........... ☐1 No .... ☐2

Q20. Do you have access to the internet at home, in school or somewhere else? Yes..... ☐1 No .... ☐2 → **Go to Q23**

Q21a. Where/how do you access the internet? [TICK ALL THAT APPLY]

A. At school .... ☐1
B. At home on a PC or laptop in a family room .... ☐2
C. At home on a PC or laptop in your bedroom.......... ☐3
D. Via a games console .... ☐4
E. Via Internet TV / cable in a family room .... ☐5
F. Via mobile phone / ipad or other mobile device...... ☐6
G. Other (please specify) ______________________ ☐7

Q21b. What do you use the internet for? [TICK 'YES' OR 'NO' FOR EACH]

	Yes	No
A. Playing games	☐1	☐2
B. Personal webpage (Facebook, Bebo, Twitter, etc) / instant messaging / emailing....	☐1	☐2
C. Watching movies / downloading music	☐1	☐2
D. Surfing the internet for fun	☐1	☐2
E. Doing homework..	☐1	☐2
F. Surfing the internet for school projects	☐1	☐2

Q22. Are you allowed to use the internet without your parents or another adult checking what you are doing? [TICK ONE BOX ONLY]

Yes always .... ☐1 Yes sometimes................ ☐2 No .... ☐3

Q23. On an average school day, how much time in a day do you spend alone at home while nobody else is home? [TICK ONE BOX ONLY]

None.. ☐1
Less than 1 hour .... ☐2
1 to less than 2 hours a day................................. ☐3
2 to less than 3 hours a day................................. ☐4
3 to less than 4 hours a day......... ☐5
4 to less than 6 hours a day .... ☐6
6 or more hours a day .... ☐7

Q24. The following questions refer to the rules and limits your parents may place on your activities.

A. Do you need your parents' permission before going out on week nights? [TICK ONE BOX ONLY]

Almost never or never	Not very often	Sometimes	Often	Almost always or always	Not applicable / don't do it
☐1	☐2	☐3	☐4	☐5	☐6

B. If you go out on a Saturday evening, do you have to inform your parents beforehand about who you will be with and where you will be going? [TICK ONE BOX ONLY]

Almost never or never	Not very often	Sometimes	Often	Almost always or always	Not applicable / don't do it
☐1	☐2	☐3	☐4	☐5	☐6

C. If you have been out very late one night, do your parents make you explain why and tell them who you were with? [TICK ONE BOX ONLY]

Almost never or never	Not very often	Sometimes	Often	Almost always or always	Not applicable / don't do it
☐1	☐2	☐3	☐4	☐5	☐6

D. Do your parents demand to know where you are in the evenings, who you are going to be with, and what you are going to be doing? [TICK ONE BOX ONLY]

Almost never or never	Not very often	Sometimes	Often	Almost always or always	Not applicable / don't do it
☐1	☐2	☐3	☐4	☐5	☐6

E. Do you have to ask your parents before you can make plans with friends about what you will do on a Saturday night? [TICK ONE BOX ONLY]

Almost never or never	Not very often	Sometimes	Often	Almost always or always	Not applicable / don't do it
☐1	☐2	☐3	☐4	☐5	☐6

F. Do your parents make you tell them how you spend your money? [TICK ONE BOX ONLY]

Almost never or never	Not very often	Sometimes	Often	Almost always or always	Not applicable / don't do it
☐1	☐2	☐3	☐4	☐5	☐6

Q25. How much spending money, if any, do you have to spend each week?

_____**Euro** _____**Cent** None …….☐1 ⟶ **Go to Q27**

Q26. Where do you get this money from? [TICK ALL THAT APPLY]

Regular pocket money ....☐1
Doing chores (or babysitting) in the home ....☐2
Given money by parents when I need it ....☐3
Doing occasional jobs (e.g. babysitting) outside the home ....☐4
Have a regular part-time job ....☐5

Now some questions about exercise and sport.

Q27. How many times in the past <u>14 days</u> have you done at least 20 minutes of exercise <u>hard</u> enough to make you breathe fast and make your heart beat faster? (Hard exercise includes, for example, playing football, jogging, fast cycling). Include time spent in physical education class. [TICK ONE BOX ONLY]

None ....☐1 1 to 2 days ....☐2 3 to 5 days ....☐3 6 to 8 days ....☐4 9 or more days ....☐5

Q28. How many times in the past <u>14 days</u> have you done at least 20 minutes of <u>light</u> exercise that was <u>not hard</u> enough to make you breathe heavily and make your heart beat fast? (Light exercise includes walking or slow cycling). Include time spent in physical education class. [TICK ONE BOX ONLY]

None ....☐1 1 to 2 days ....☐2 3 to 5 days ....☐3 6 to 8 days ....☐4 9 or more days ....☐5

Q29. Outside of your physical education classes, how many team or individual sports or activities did you participate in during the past 12 months (for example, a school or local football/netball team, athletics, tennis etc.)? [TICK ONE BOX ONLY]

None ....☐1 1 activity ....☐2 2 activities ....☐3 3 activities ....☐4 4 or more activities.☐5

Q30. Please tell us the reasons why you choose not to participate in sporting activities? [TICK ALL THAT APPLY]

I do not like team games..☐1
I am no good at games ....☐2
I have no opportunities to play...☐3
I feel people laugh at me because of my size ....☐4
I have a disability or health problem which prevents me from playing ....☐5
I prefer to watch sports on TV ....☐6
I do not fit in with the sporty crowd.........................☐7
I do not like to get dirty or sweaty ....☐8
I am not competitive ....☐9
I prefer to play computer games ....☐10
Other reason (please specify) ______________ ☐11

Q31. Please tick below to indicate (a) how often do you do each of these activities and (b), if you do them, whether or not they are paid for by your parents or by yourself:

	**(a) How often do you do each of these activities?**				**(b) Does this activity have to be paid for?**		
	Never	Less than once a week	1-3 times a week	4 or more times a week	No	Yes, my parents pay for it	Yes, I pay for it myself
A. Play sports or undertake physical activities without a coach or instructor (e.g. biking, skate-boarding etc.)?	☐1	☐2	☐3	☐4	☐1	☐2	☐3
B. Play sports with a coach or instructor, or as part of an organised team, other than in P.E. class? (swimming, soccer, hockey,etc)?	☐1	☐2	☐3	☐4	☐1	☐2	☐3
C. Take part in dance, drama or music lessons	☐1	☐2	☐3	☐4	☐1	☐2	☐3
D. Take part in a homework club (either in school or elsewhere)	☐1	☐2	☐3	☐4	☐1	☐2	☐3
E. Take part in clubs or groups such as Guides or Scouts, youth club, community or church groups	☐1	☐2	☐3	☐4	☐1	☐2	☐3

Q31c. If you do any of the above activities, do you have special responsibilities, such as team leader, captain, secretary, etc.?

Yes ....☐1 No...........☐2 Don't do any of the activities ....☐3

We would now like to ask some questions about the things that you eat.

Q32. Do you usually have something to eat at home before going to school?

Yes ....☐1 No ....☐2

Q33. We would like you to think back to what you ate yesterday. Did you eat each of these foods Once, More than Once, or Not at All? [TICK ONE BOX ON EACH LINE]

	**Once**	**More than Once**	**Not at All**
A. Fresh fruit	☐1	☐2	☐3
B. Cooked vegetables	☐1	☐2	☐3
C. Raw vegetables or salad	☐1	☐2	☐3
D. Hamburger, hot dog, sausage or sausage roll, meat pie	☐1	☐2	☐3
E. Hot chips or french fries	☐1	☐2	☐3
F. Crisps or savoury snacks	☐1	☐2	☐3
G. Biscuits, doughnuts, cake, pie or chocolate	☐1	☐2	☐3
H. Sweets	☐1	☐2	☐3
I. Full-fat cheese / yoghurt / fromage frais	☐1	☐2	☐3
J. Low-fat cheese / low-fat yoghurt	☐1	☐2	☐3
K. Water (tap water / still water / fizzy water)	☐1	☐2	☐3
L. Fizzy drinks / minerals / cordial / squash (diet)	☐1	☐2	☐3
M. Fizzy drinks / minerals / cordial / squash (not diet)	☐1	☐2	☐3
N. Full cream milk	☐1	☐2	☐3
O. Skimmed / semi-skimmed milk	☐1	☐2	☐3

Q34. How often do you brush your teeth? [TICK ONE BOX ONLY]

More than twice a day	☐1	Less often than once a day	☐4
Twice a day	☐2	Rarely	☐5
Once a day	☐3	Not at all	☐6

Q35. Do you do any of these chores at home? [TICK ONE BOX ON EACH LINE]

	Every day	4/5 times a week	2/3 times a week	Less Often	Never
A. Help with cooking for the family	☐1	☐2	☐3	☐4	☐5
B. Hoovering / cleaning	☐1	☐2	☐3	☐4	☐5
C. Helping in the garden	☐1	☐2	☐3	☐4	☐5
D. Washing the dishes / emptying the dishwasher	☐1	☐2	☐3	☐4	☐5
E. Putting out the bin / recycling	☐1	☐2	☐3	☐4	☐5
F. Cleaning the car	☐1	☐2	☐3	☐4	☐5
G. Helping with your younger brothers or sisters	☐1	☐2	☐3	☐4	☐5
H. Helping an elderly or sick relative in the family	☐1	☐2	☐3	☐4	☐5

Q36. How many friends do you normally hang around with? [TICK ONE BOX ONLY]

A. None	☐1 **Go to Q41**	D. Between 6 and 10	☐4 **Go to Q37**
B. One or two	☐2 **Go to Q37**	E. More than 10	☐5 **Go to Q37**
C. Between 3 and 5	☐3 **Go to Q37**		

Q37. How many of these would you describe as CLOSE friends? _____________

Q38. How old are the friends you usually go about with? [TICK ONE BOX ON EACH LINE]

	None	Some	Most or all
A. A year or more younger	☐1	☐2	☐3
B. About the same age	☐1	☐2	☐3
C. A year or two older	☐1	☐2	☐3
D. More than two years older	☐1	☐2	☐3

Q39. How many of your friends have your parents met? [TICK ONE BOX ONLY]

None of them	☐1
Some of them	☐2
Most or all of them	☐3

Q40. This part asks about your feelings about your relationships with your close friends. Please read each statement and tick the ONE number that tells how true the statement is for you now. [TICK ONE BOX ON EACH LINE]

	Almost never or never true	Not very often true	Sometimes true	Often true	Almost always or always true
A. Talking over my problems with friends makes me feel ashamed or foolish	☐1	☐2	☐3	☐4	☐5
B. I wish I had different friends	☐1	☐2	☐3	☐4	☐5
C. My friends understand me	☐1	☐2	☐3	☐4	☐5
D. My friends accept me as I am	☐1	☐2	☐3	☐4	☐5
E. I feel the need to be in touch with my friends more often	☐1	☐2	☐3	☐4	☐5
F. My friends don't understand what I'm going through these days	☐1	☐2	☐3	☐4	☐5
G. I feel alone or apart when I am with my friends	☐1	☐2	☐3	☐4	☐5
H. My friends listen to what I have to say	☐1	☐2	☐3	☐4	☐5
I. I feel my friends are good friends	☐1	☐2	☐3	☐4	☐5
J. My friends are fairly easy to talk to	☐1	☐2	☐3	☐4	☐5
K. When I am angry about something, my friends try to be understanding	☐1	☐2	☐3	☐4	☐5
L. I feel angry with my friends	☐1	☐2	☐3	☐4	☐5
M. I can count on my friends when I need to get something off my chest	☐1	☐2	☐3	☐4	☐5
N. I trust my friends	☐1	☐2	☐3	☐4	☐5
O. My friends respect my feelings	☐1	☐2	☐3	☐4	☐5
P. I get upset a lot more than my friends know about	☐1	☐2	☐3	☐4	☐5
Q. It seems as if my friends are irritated with me for no reason	☐1	☐2	☐3	☐4	☐5

Q41. The next set of questions are about how you have been feeling recently. For each question, please indicate how much you have felt or acted this way in the past two weeks.

If a sentence was true about how you felt or acted most of the time, answer TRUE. it was only sometimes true, answer SOMETIMES. If a sentence was not true about you, answer NOT TRUE.

	TRUE	SOMETIMES	NOT TRUE
A. I felt miserable or unhappy	☐1	☐2	☐3
B. I didn't enjoy anything at all	☐1	☐2	☐3
C. I felt so tired I just sat around and did nothing	☐1	☐2	☐3
D. I was very restless	☐1	☐2	☐3
E. I felt I was no good any more	☐1	☐2	☐3
F. I cried a lot	☐1	☐2	☐3
G. I found it hard to think properly or concentrate	☐1	☐2	☐3
H. I hated myself	☐1	☐2	☐3
I. I was a bad person	☐1	☐2	☐3
J. I felt lonely	☐1	☐2	☐3
K. I thought nobody really loved me	☐1	☐2	☐3
L. I thought I could never be as good as other kids	☐1	☐2	☐3
M. I did everything wrong	☐1	☐2	☐3

Q42. Have you been bullied in the last 3 months?

Yes ☐1 No ☐2 – **Go to Q49**

Q43. How often did this bullying take place? [TICK ONE BOX ONLY]

Once or twice ☐1
2 or 3 times a month ☐2
About once a week ☐3
Several times a week ☐4

Q44. What form did the bullying take? [TICK ALL THAT APPLY]

A. Physical bullying ☐1
B. Verbal bullying (name-calling, hurtful slagging) ☐2
C. Electronic (phone messaging, emails, Facebook, etc) ☐3
D. Graffiti / pinning up notes / passing notes in class ☐4
E. Taking / damaging personal possessions ☐5
F. Exclusion (being left out) ☐6
G. Gossip, spreading rumours ☐7
H. Threatened / forced to do things you didn't want to do ☐8
I. Other please (specify) ________ ☐9

Q45. What was the reason for the bullying? [TICK ALL THAT APPLY]

A. Ethnicity / race / nationality / skin colour ☐1
B. Physical disability ☐2
C. Learning difficulty / disability ☐3
D. Religion ☐4
E. Class performance / seen as star pupil ☐5
F. Teacher's pet ☐6
G. Physical appearance (clothes, glasses, weight, height, etc) ☐6
H. Family background ☐7
I. Seen not to conform to gender roles ☐8
J. Jealousy ☐9
K. Other (please specify) ________ ☐10

Q46. When you were bullied, how did this make you feel? [TICK ONE BOX ON EACH LINE]

	Not at all	A little	A lot
Upset	☐1	☐2	☐3
Afraid	☐1	☐2	☐3
Angry	☐1	☐2	☐3
Wanted to take revenge	☐1	☐2	☐3
Shrugged it off	☐1	☐2	☐3
Isolated	☐1	☐2	☐3
Determined to do something about it	☐1	☐2	☐3
Other (please specify) ________	☐1	☐2	☐3

Q47. Have you told anyone that you have been bullied? Yes ☐1 No ☐2

Q48. Who have you told you have been bullied? [TICK ALL THAT APPLY]

Teacher. ☐1
Parent(s) ☐2
Friend ☐3
Other (please specify) ________ ☐8

Q49. In the last 3 months have you bullied someone?

Yes........☐1 No ....☐2 – **Go to Q54**

Q50. How often did you bully someone? [TICK ONE BOX ONLY]

Once or twice .... ☐1
2 or 3 times a month .... ☐2
About once a week .... ☐3
Several times a week.......... ☐4

Q51. What form did the bullying take? [TICK ALL THAT APPLY]

A. Physical bullying .... ☐1
B. Verbal bullying (name-calling, hurtful slagging)........... ☐2
C. Electronic (phone messaging, emails, Facebook, etc) ☐3
D. Graffiti / pinning up notes / passing notes in class....... ☐4
E. Taking / damaging personal possessions .... ☐5
F. Exclusion (being left out) .... ☐6
G. Gossip, spreading rumours.. ☐7
H. Threatened / forced to do things they didn't want to do ☐8
I. Other (please specify) ______________________ ☐9

Q52. What was the reason for the bullying? [TICK ALL THAT APPLY]

A. Ethnicity / race / nationality / skin colour .... ☐1
B. Physical disability .... ☐2
C. Learning difficulty / disability........................... ☐3
D. Religion.. ☐4
E. Class performance / star pupil......................... ☐5
F. Teacher's pet .... ☐6
G. Physical appearance (clothes, glasses, weight, height, etc) . ☐6
H. Family background.. ☐7
I. Seen not to conform to gender roles .... ☐8
J. Jealousy .... ☐9
K. Other (please specify) ______________________ ☐10

Q53. What caused you to bully someone? [TICK ALL THAT APPLY]

A. Having a bad day.. ☐1
B. Dislike of the person .... ☐2
C. Jealousy of the person .... ☐3
D. To impress friends.. ☐4
E. To be feared .... ☐5
F. Enjoy hurting people .... ☐6
G. To be accepted by the group/gang .... ☐7
H. To get someone back / get revenge........................ ☐8
I. Other (please specify) ______________________ ☐9

And now, some more questions about you …

Q54. How would you describe yourself? [TICK ONE BOX ONLY]

Very skinny.. ☐1
A bit skinny.. ☐2
Just the right size .... ☐3
A bit overweight .... ☐4
Very overweight .... ☐5

Q55. Have you ever exercised to lose weight or to avoid gaining weight?

Yes..........☐1 No ....☐2

Q56. Have you ever eaten less food, fewer calories, or foods low in fat to lose weight or to avoid gaining weight?

Yes..........☐1 No ....☐2

Q57. How often do you weigh yourself? [TICK ONE BOX ONLY]

More than once a day .... ☐1
Every day .... ☐2
Once a week .... ☐3
Once a month...................................... ☐4
Less than once a month....................... ☐5
Never.. ☐6

Q58. Which of the following are you trying to do about your weight? [TICK ONE BOX ONLY]

Lose weight .... ☐1
Gain weight .... ☐2
Stay the same weight... ☐3
I am not trying to do anything about my weight .... ☐4

Q59. When you misbehave, how often do your parents do the following? [TICK ONE BOX ON EACH LINE]

	Always	Sometimes	Never
a. Explain to you what you have done wrong	☐1	☐2	☐3
b. Ignore you	☐1	☐2	☐3
c. Slap or hit you	☐1	☐2	☐3
d. Shout at you	☐1	☐2	☐3
e. Send you out of the room or to your bedroom	☐1	☐2	☐3
f. Stop your treats or pocket money	☐1	☐2	☐3
g. Give out to you	☐1	☐2	☐3
h. Offer you treats to be good	☐1	☐2	☐3
i. Ground you	☐1	☐2	☐3

Q60. Here are some questions about how you feel about yourself. Please tick Yes or No for *each* question. Remember we won't tell anyone your answers.

	Yes	No
1. My classmates make fun of me	☐1	☐2
2. I am a happy person	☐1	☐2
3. It is hard for me to make friends	☐1	☐2
4. I am often sad	☐1	☐2
5. I am smart	☐1	☐2
6. I am shy	☐1	☐2
7. I get nervous when the teacher calls on me	☐1	☐2
8. My looks bother me	☐1	☐2
9. I am a leader in games and sports	☐1	☐2
10. I get worried when I have tests in school	☐1	☐2
11. I am unpopular	☐1	☐2
12. I am well-behaved in school	☐1	☐2
13. It is usually my fault when something goes wrong	☐1	☐2
14. I cause trouble to my family	☐1	☐2
15. I am strong	☐1	☐2
16. I am an important member of my family	☐1	☐2
17. I give up easily	☐1	☐2
18. I am good at school work	☐1	☐2
19. I do many bad things	☐1	☐2
20. I behave badly at home	☐1	☐2
21. I am slow in finishing my school work	☐1	☐2
22. I am an important member of my class	☐1	☐2
23. I am nervous	☐1	☐2
24. I can give a good report in front of the class	☐1	☐2
25. In school I am a dreamer	☐1	☐2
26. My friends like my ideas	☐1	☐2
27. I often get into trouble	☐1	☐2
28. I am lucky	☐1	☐2
29. I worry a lot	☐1	☐2
30. My parents expect too much of me	☐1	☐2
31. I like being the way I am	☐1	☐2
32. I feel left out of things	☐1	☐2
33. I have nice hair	☐1	☐2
34. I often volunteer in school	☐1	☐2
35. I wish I was different	☐1	☐2
36. I hate school	☐1	☐2
37. I am among the last to be chosen for games and sports	☐1	☐2
38. I am often mean to other people	☐1	☐2
39. My classmates in school think I have good ideas	☐1	☐2
40. I am unhappy	☐1	☐2
41. I have many friends	☐1	☐2
42. I am cheerful	☐1	☐2
43. I am dumb about most things	☐1	☐2
44. I am good-looking	☐1	☐2
45. I get into a lot of fights	☐1	☐2
46. I am popular with boys	☐1	☐2
47. People pick on me	☐1	☐2
48. My family is disappointed in me	☐1	☐2

49. I have a pleasant face ☐1 ☐2
50. When I grow up I will be an important person ☐1 ☐2
51. In games and sports, I watch instead of play ☐1 ☐2
52. I forget what I learn ☐1 ☐2
53. I am easy to get along with ☐1 ☐2
54. I am popular with girls ☐1 ☐2
55. I am a good reader ☐1 ☐2
56. I am often afraid ☐1 ☐2
57. I am different from other people ☐1 ☐2
58. I think bad thoughts ☐1 ☐2
59. I cry easily ☐1 ☐2
60. I am a good person ☐1 ☐2

Q61. Looking to the future, if you had your choice, what job would you really like to get?

____

____

Q62a. Were you alone when completing the questionnaire?

Yes ☐1 No ☐2

Q62b. Who else was present in the room with you? [TICK ALL THAT APPLY]

Parent ☐1
Interviewer ☐2
Other adult ☐3
Brother / sister ☐4
Other child ☐5

YOUNG PERSON SENSITIVE QUESTIONNAIRE

(MALE AND FEMALE VERSIONS)

The Economic and Social Research Institute
Whitaker Square
Sir John Rogerson's Quay
Dublin 2
Ph: 01-863 2000 Fax 01-863 2100

An Roinn Leanaí agus Gnóthaí Óige
Department of Children and Youth Affairs

University of Dublin
Trinity College
College Green
Dublin 2

TRINITY COLLEGE DUBLIN

GROWING UP IN IRELAND – the national longitudinal study of children

STRICTLY CONFIDENTIAL

YOUNG PERSON SENSITIVE QUESTIONNAIRE (Male)

AREA ☐☐☐☐ **H'HOLD** ☐☐☐

Once again, thank you for helping us with ***Growing Up in Ireland***. Remember that this is not a test and there are no right or wrong answers. Take your time and try to answer each question the way you really think. If you need help just let the interviewer know.

We will not tell anyone the answers to your questions. But if you tell us something that makes us worried about you, then we might have to tell someone who could help.

1. Are you currently taught Relationships and Sexuality Education (RSE) in your school? Yes ...... ☐1 No ☐2

2. Were you taught Relationships and Sexuality Education (RSE) in primary school?

Yes .... ☐1 No.................. ☐2 Still at Primary School............ ☐2

3a. Have you ever discussed sex and/or relationship issues with your parent(s) / guardian(s)?

Yes .... ☐1 No.................. ☐2

3b.Where would you be MOST likely to go to get information or advice on sex or relationship issues
[TICK ONE BOX ONLY]

Nowhere	☐1	Boyfriend / Girlfriend	☐8
Mum	☐2	Teacher	☐9
Dad	☐3	Internet	☐10
Brother /sister	☐4	Magazines	☐11
Aunts / Uncles	☐5	Books	☐12
Friends	☐6	TV / Films / DVDs	☐13
Cousins	☐7	Other (please specify) ________	☐14

Boys' bodies develop at different rates. We would like to ask you a few questions about your stage of development at the moment.

4. Has your voice changed at all? **[TICK ONE BOX ONLY]**

No, it is the same	☐1	Yes, it is now totally changed	☐3
Yes, occasionally it is a lot lower	☐2	Not sure	☐4

5a. How often in the last year have you done any of the following? **[TICK ONE BOX ON EACH LINE]**

	Never	Once	2 to 5 times	6 or more times
1. Not paid the correct fare on a bus or train	☐1	☐2	☐3	☐4
2. Taken something from a shop or store without paying for it	☐1	☐2	☐3	☐4
3. Behaved badly in public so that people complained and you got into trouble	☐1	☐2	☐3	☐4
4. Stolen or ridden in a stolen car or a van or on a stolen motorbike	☐1	☐2	☐3	☐4
5. Taken money or something else that did not belong to you from school	☐1	☐2	☐3	☐4
6. Carried a knife or weapon with you in case it was needed in a fight	☐1	☐2	☐3	☐4
7. Deliberately damaged or destroyed property that did not belong to you (e.g., windows, cars, streetlights)	☐1	☐2	☐3	☐4
8. Broken into a house or building to steal something	☐1	☐2	☐3	☐4
9. Written things or sprayed paint on things that do not belong to you				

	Never	Once	2 to 5 times	6 or more times
(for example, a phone box, car, building, bus shelter)	☐1	☐2	☐3	☐4
10. Used force, threats or a weapon to get money or something else from somebody	☐1	☐2	☐3	☐4
11. Taken money or something else that did not belong to you from your home without permission	☐1	☐2	☐3	☐4
12. Broken into a car or van to steal something from it	☐1	☐2	☐3	☐4
13. Deliberately set fire or tried to set fire to someone's property or a building (e.g., school or shed)	☐1	☐2	☐3	☐4
14. Hit, kicked or punched someone on purpose in order to hurt or injure them	☐1	☐2	☐3	☐4
15. Been involved in a serious physical fight where someone got badly hurt or needed to see a doctor	☐1	☐2	☐3	☐4

The people responsible for Growing Up in Ireland would like to make it clear that a lot of the activities mentioned are very dangerous and undesirable (especially for a young person like you) and that some of them are illegal.

**5b. Can I ask:**	**No, never**	**Maybe**	**Yes, definitely**
Have you ever heard voices or sounds that no-one else can hear?	☐1	☐2	☐3
Have you ever seen things that other people could not see?	☐1	☐2	☐3
Have you ever thought that people are following you or spying on you?	☐1	☐2	☐3
Some people believe that their thoughts can be read by another person. Have other people ever read your mind?	☐1	☐2	☐3
Have you ever felt that you were under the control of some special power?	☐1	☐2	☐3
Have you ever felt that you have extra-special powers?	☐1	☐2	☐3

6. Have you ever been in trouble with the gardai? Yes ☐1 No ☐2

7a. Have you ever smoked a cigarette?

Yes ☐1 No ☐2 **– Go to Question 8a**

7b. How often do you smoke cigarettes at present?

Every day ☐1
At least once a week but not every day ☐2
Less than once a week ☐3 **– Go to Question 8a**
I do not smoke at present ☐4 **– Go to Question 8a**

7c. How many cigarettes do you usually smoke in a week? ________ cigarettes a week

8a. Have you ever had an alcoholic drink (other than just a few sips)? (That means beer, wine, cider or spirits like vodka, whiskey, etc.)

Yes ☐1 No ☐2 **– Go to Question 9**

8b. During the last year did you have a whole alcoholic drink? (That means beer, wine, cider or spirits like vodka, whiskey, etc.)

Yes ☐1 No ☐2 **– Go to Question 9**

8c. How often do you drink alcohol now? Try to include even those times when you only drink a small amount.

Never ☐1
Rarely ☐2
Only on special occasions ☐3
At least once a month ☐4
At least once a week ☐5
Every day ☐6

8d. Have you ever had so much alcohol that you were really drunk (or felt sick or dizzy)?

No, never ☐1
Yes, once ☐2
Yes, 2-3 times ☐3
Yes, 4-10 times ☐4
Yes, more than 10 times ☐5

9. Have you ever used cannabis? [also called 'hash', 'grass', 'weed' or 'pot']

Yes ☐1 No ☐2

10. Have you ever sniffed glue, or breathed the contents of spray cans, or inhaled any paints or sprays or petrol to get high?

Yes........... ☐1 No.............. ☐2

11. Have you ever used any other drugs (such as ecstasy, speed, heroin, methadone, crack or cocaine)?

Yes........... ☐1 No.............. ☐2

The people responsible for Growing Up in Ireland would like to make it clear that a lot of the activities mentioned in this Questionnaire are very dangerous and undesirable (especially for a young person like you) and that some of them are illegal. Drinking alcohol, taking drugs, fighting and so on always cause lots of damage and pain for everyone involved. If you would like to talk to someone about any of the activities mentioned in this Questionnaire, please let the interviewer know. This may involve talking to your parents/guardians about the matter.

Q12a. Were you alone when completing the questionnaire? Yes .... ☐1 No.................. ☐2

Q12b. Who else was present in the room with you?

Parent ... ☐1 Interviewer ... ☐2 Other adult ... ☐3 Brother / sister .... ☐4 Other child .... ☐5

ESRI
The Economic and Social Research Institute
Whitaker Square
Sir John Rogerson's Quay
Dublin 2
Ph: 01-863 2000 Fax 01-863 2100

An Roinn Leanaí agus Gnóthaí Óige
Department of Children and Youth Affairs

University of Dublin
Trinity College
College Green
Dublin 2

TRINITY COLLEGE DUBLIN

GROWING UP IN IRELAND – the national longitudinal study of children

STRICTLY CONFIDENTIAL

YOUNG PERSON SENSITIVE QUESTIONNAIRE (Female)

AREA ☐☐☐☐ **H'HOLD** ☐☐☐

Once again, thank you for helping us with ***Growing Up in Ireland***. Remember that this is not a test and there are no right or wrong answers. Take your time and try to answer each question the way you really think. If you need help just let the interviewer know.

We will not tell anyone the answers to your questions. But if you tell us something that makes us worried about you, then we might have to tell someone who could help.

1. Are you currently taught Relationships and Sexuality Education (RSE) in your school? Yes ......☐1 No ☐2

2. Were you taught Relationships and Sexuality Education (RSE) in primary school?

Yes ....☐1 No..................☐2 Still at Primary School............☐2

3a. Have you ever discussed sex and/or relationship issues with your parent(s) / guardian(s)?

Yes ....☐1 No..................☐2

3b.Where would you be MOST likely to go to get information or advice on sex or relationship issues?
[TICK ONE BOX ONLY]

Nowhere....................☐1
Mum ....☐2
Dad....................☐3
Brother/sister....................☐4
Aunts/ Uncles....................☐5
Friends ....☐6
Cousins ....☐7
Boyfriend/ Girlfriend ....☐8
Teacher....................☐9
Internet....................☐10
Magazines....................☐11
Books ....☐12
TV/ Films/ DVDs ....☐13
Other (please specify) ________________ ☐14

4a. Girls can start their periods at different ages. Have you started your periods yet?

Yes ....☐1 No ....☐2 – Go to Question 5a

4b. What age were you when you had your first period? ______ years _______months

5a. How often in the last year have you done any of the following? **[TICK ONE BOX ON EACH LINE]**

	Never	Once	2 to 5 times	6 or more times
1. Not paid the correct fare on a bus or train	☐1	☐2	☐3	☐4
2. Taken something from a shop or store without paying for it	☐1	☐2	☐3	☐4
3. Behaved badly in public so that people complained and you got into trouble	☐1	☐2	☐3	☐4
4. Stolen or ridden in a stolen car or a van or on a stolen motorbike	☐1	☐2	☐3	☐4
5. Taken money or something else that did not belong to you from school	☐1	☐2	☐3	☐4
6. Carried a knife or weapon with you in case it was needed in a fight	☐1	☐2	☐3	☐4
7. Deliberately damaged or destroyed property that did not belong to you (e.g., windows, cars, streetlights)	☐1	☐2	☐3	☐4
8. Broken into a house or building to steal something	☐1	☐2	☐3	☐4
9. Written things or sprayed paint on things that do not belong to you				

(for example, a phone box, car, building, bus shelter) ☐1 ☐2 ☐3 ☐4

	Never	Once	2 to 5 times	6 or more times
10. Used force, threats or a weapon to get money or something else from somebody	☐1	☐2	☐3	☐4
11. Taken money or something else that did not belong to you from your home without permission	☐1	☐2	☐3	☐4
12. Broken into a car or van to steal something from it	☐1	☐2	☐3	☐4
13. Deliberately set fire or tried to set fire to someone's property or a building (e.g. school or shed)	☐1	☐2	☐3	☐4
14. Hit, kicked or punched someone on purpose in order to hurt or injure them	☐1	☐2	☐3	☐4
15. Been involved in a serious physical fight where someone got badly hurt or needed to see a doctor	☐1	☐2	☐3	☐4

The people responsible for Growing Up in Ireland would like to make it clear that a lot of the activities mentioned are very dangerous and undesirable (especially for a young person like you) and that some of them are illegal.

**5b. Can I ask:**	**No, never**	**Maybe**	**Yes, definitely**
Have you ever heard voices or sounds that no-one else can hear?	☐1	☐2	☐3
Have you ever seen things that other people could not see?	☐1	☐2	☐3
Have you ever thought that people are following you or spying on you?	☐1	☐2	☐3
Some people believe that their thoughts can be read by another person. Have other people ever read your mind?	☐1	☐2	☐3
Have you ever felt that you were under the control of some special power?	☐1	☐2	☐3
Have you ever felt that you have extra-special powers?	☐1	☐2	☐3

6. Have you ever been in trouble with the gardai? Yes ☐1 No ☐2

7a. Have you ever smoked a cigarette?

Yes ☐1 No ☐2 **– Go to Question 8a**

7b. How often do you smoke cigarettes at present?

Every day ☐1
At least once a week but not every day ☐2
Less than once a week ☐3 **– Go to Question 8a**
I do not smoke at present ☐4 **– Go to Question 8a**

7c. How many cigarettes do you usually smoke in a week? ________ cigarettes a week

8a. Have you ever had an alcoholic drink (other than just a few sips)? (That means beer, wine, cider or spirits like vodka, whiskey, etc.)

Yes ☐1 No ☐2 **– Go to Question 9**

8b. During the last year did you have a whole alcoholic drink? (That means beer, wine, cider or spirits like vodka, whiskey, etc.)

Yes ☐1 No ☐2 **– Go to Question 9**

8c. How often do you drink alcohol now? Try to include even those times when you only drink a small amount.

Never ☐1
Rarely ☐2
Only on special occasions ☐3
At least once a month ☐4
At least once a week ☐5
Every day ☐6

8d. Have you ever had so much alcohol that you were really drunk (or felt sick or dizzy)?

No, never ☐1
Yes, once ☐2
Yes, 2-3 times ☐3
Yes, 4-10 times ☐4
Yes, more than 10 times ☐5

9. Have you ever used cannabis? [also called 'hash', 'grass', 'weed' or 'pot']

Yes...........☐1 No...............☐2

10. Have you ever sniffed glue, or breathed the contents of spray cans, or inhaled any paints or sprays or petrol to get high?

Yes...........☐1 No...............☐2

11. Have you ever used any other drugs (such as ecstasy, speed, heroin, methadone, crack or cocaine)?

Yes...........☐1 No...............☐2

The people responsible for Growing Up in Ireland would like to make it clear that a lot of the activities mentioned in this Questionnaire are very dangerous and undesirable (especially for a young person like you) and that some of them are illegal. Drinking alcohol, taking drugs, fighting and so on always cause lots of damage and pain for everyone involved. If you would like to talk to someone about any of the activities mentioned in this Questionnaire, please let the interviewer know. This may involve talking to your parents/guardians about the matter.

Q12a. Were you alone when completing the Questionnaire? Yes..........☐1 No.................☐2

Q12b. Who else was present in the room with you?

Parent ...☐1 Interviewer ...☐2 Other adult ...☐3 Brother / sister ....☐4 Other child ....☐5

YOUNG PERSON PARENTING INVENTORY –

MUM, DAD, MUM'S PARTNER AND DAD'S PARTNER

The Economic and Social Research Institute
Whitaker Square
Sir John Rogerson's Quay
Dublin 2

An Roinn Leanaí agus Gnóthaí Óige
Department of Children and Youth Affairs

University of Dublin
Trinity College
College Green
Dublin 2

GROWING UP IN IRELAND – the national longitudinal study of children

STRICTLY CONFIDENTIAL

YOUNG PERSON SELF-COMPLETE QUESTIONNAIRE ON MUM (M)

AREA ☐☐☐☐ **H'HOLD** ☐☐☐

Once again, thank you for helping us with ***Growing Up in Ireland***. Remember that this is not a test and there are no right or wrong answers. We will not tell anyone the answers to your questions. But if you tell us something that makes us worried about you, then we might have to tell someone who could help.

<u>We would now like to ask you some questions about your Mum</u>

1. How well do you get on with your Mum?

Very well	Fairly well	You and your Mum do not get on
☐1	☐2	☐3

	Strongly Disagree	Disagree	I'm in Between	Agree	Strongly Agree
2. My Mum doesn't really like me to tell her my troubles	☐1	☐2	☐3	☐4	☐5
3. My Mum hardly ever praises me for doing well	☐1	☐2	☐3	☐4	☐5
4. I can count on my Mum to help me out if I have a problem	☐1	☐2	☐3	☐4	☐5
5. My Mum spends time just talking to me	☐1	☐2	☐3	☐4	☐5
6. My Mum and I do things that are fun together	☐1	☐2	☐3	☐4	☐5
7. My Mum tells me that her ideas are correct and that I shouldn't question them	☐1	☐2	☐3	☐4	☐5
8. My Mum respects my privacy	☐1	☐2	☐3	☐4	☐5
9. My Mum gives me a lot of freedom	☐1	☐2	☐3	☐4	☐5
10. My Mum makes most of the decisions about what I should do	☐1	☐2	☐3	☐4	☐5
11. My Mum believes I have a right to my own point of view	☐1	☐2	☐3	☐4	☐5
12. My Mum really expects me to follow family rules	☐1	☐2	☐3	☐4	☐5
13. My Mum really lets me get away with things	☐1	☐2	☐3	☐4	☐5
14. If I don't behave myself, my Mum will punish me	☐1	☐2	☐3	☐4	☐5
15. My Mum points out ways I could do better	☐1	☐2	☐3	☐4	☐5
16. When I do something wrong, my Mum does not punish me	☐1	☐2	☐3	☐4	☐5

Q17a. Were you alone when completing the questionnaire?

Yes .... ☐1 No............ ☐2

Q17b. Who else was present in the room with you? (Tick all that apply)

Parent .... ☐1
Interviewer .... ☐2
Other adult .... ☐3
Brother / sister .... ☐4
Other child .... ☐5

The Economic and Social Research Institute
Whitaker Square
Sir John Rogerson's Quay
Dublin 2

An Roinn Leanaí agus Gnóthaí Óige
Department of Children and Youth Affairs

University of Dublin
Trinity College
College Green
Dublin 2

GROWING UP IN IRELAND – the national longitudinal study of children
STRICTLY CONFIDENTIAL

YOUNG PERSON SELF-COMPLETE QUESTIONNAIRE ON DAD (D)

AREA ☐☐☐☐ **H'HOLD** ☐☐☐

Once again, thank you for helping us with ***Growing Up in Ireland***. Remember that this is not a test and there are no right or wrong answers. We will not tell anyone the answers to your questions. But if you tell us something that makes us worried about you, then we might have to tell someone who could help.

We would now like to ask you some questions about your Dad

1. How well do you get on with your Dad?

Very well ☐1 .... Fairly well ☐2 .... You and your Dad do not get on ☐3

	Strongly Disagree	Disagree	I'm in Between	Agree	Strongly Agree
2. My Dad doesn't really like me to tell him my troubles	☐1	☐2	☐3	☐4	☐5
3. My Dad hardly ever praises me for doing well	☐1	☐2	☐3	☐4	☐5
4. I can count on my Dad to help me out if I have a problem	☐1	☐2	☐3	☐4	☐5
5. My Dad spends time just talking to me	☐1	☐2	☐3	☐4	☐5
6. My Dad and I do things that are fun together	☐1	☐2	☐3	☐4	☐5
7. My Dad tells me that his ideas are correct and that I shouldn't question them	☐1	☐2	☐3	☐4	☐5
8. My Dad respects my privacy	☐1	☐2	☐3	☐4	☐5
9. My Dad gives me a lot of freedom	☐1	☐2	☐3	☐4	☐5
10. My Dad makes most of the decisions about what I should do	☐1	☐2	☐3	☐4	☐5
11. My Dad believes I have a right to my own point of view	☐1	☐2	☐3	☐4	☐5
12. My Dad really expects me to follow family rules	☐1	☐2	☐3	☐4	☐5
13. My Dad really lets me get away with things	☐1	☐2	☐3	☐4	☐5
14. If I don't behave myself, my Dad will punish me	☐1	☐2	☐3	☐4	☐5
15. My Dad points out ways I could do better	☐1	☐2	☐3	☐4	☐5
16. When I do something wrong, my Dad does not punish me	☐1	☐2	☐3	☐4	☐5

Q17a. Were you alone when completing the questionnaire?

Yes .... ☐1 No............ ☐2

Q17b. Who else was present in the room with you? (Tick all that apply)

Parent .... ☐1
Interviewer .... ☐2
Other adult .... ☐3
Brother / sister .... ☐4
Other child .... ☐5

The Economic and Social Research Institute
Whitaker Square
Sir John Rogerson's Quay
Dublin 2
Ph: 01-863 2000 Fax 01-863 2100

An Roinn Leanaí agus Gnóthaí Óige
Department of Children and Youth Affairs

University of Dublin
Trinity College
College Green
Dublin 2

TRINITY COLLEGE DUBLIN

GROWING UP IN IRELAND – the national longitudinal study of children

STRICTLY CONFIDENTIAL
YOUNG PERSON SELF-COMPLETE QUESTIONNAIRE ON STEP-DAD / MOTHER'S PARTNER (MP)

AREA ☐☐☐☐ **H'HOLD** ☐☐

Once again, thank you for helping us with ***Growing Up in Ireland***. Remember that this is not a test and there are no right or wrong answers. We will not tell anyone the answers to your questions. But if you tell us something that makes us worried about you, then we might have to tell someone who could help.

We would now like to ask you some questions about your step-Dad or your Mum's partner who lives at home with you

1. How well do you get on with him?

Very well	Fairly well	You and your step-Dad/Mum's partner do not get on
☐1	☐2	☐3

	Strongly Disagree	Disagree	I'm in Between	Agree	Strongly Agree
2. He doesn't really like me to tell her my troubles	☐1	☐2	☐3	☐4	☐5
3. He hardly ever praises me for doing well	☐1	☐2	☐3	☐4	☐5
4. I can count on him to help me out if I have a problem	☐1	☐2	☐3	☐4	☐5
5. He spends time just talking to me	☐1	☐2	☐3	☐4	☐5
6. He and I do things that are fun together	☐1	☐2	☐3	☐4	☐5
7. He tells me that his ideas are correct and that I shouldn't question them	☐1	☐2	☐3	☐4	☐5
8. He respects my privacy	☐1	☐2	☐3	☐4	☐5
9. He gives me a lot of freedom	☐1	☐2	☐3	☐4	☐5
10. He makes most of the decisions about what I should do	☐1	☐2	☐3	☐4	☐5
11. He believes I have a right to my own point of view	☐1	☐2	☐3	☐4	☐5
12. He really expects me to follow family rules	☐1	☐2	☐3	☐4	☐5
13. He really lets me get away with things	☐1	☐2	☐3	☐4	☐5
14. If I don't behave myself, he will punish me	☐1	☐2	☐3	☐4	☐5
15. He points out ways I could do better	☐1	☐2	☐3	☐4	☐5
16. When I do something wrong, he does not punish me	☐1	☐2	☐3	☐4	☐5

Q17a. Were you alone when completing the questionnaire?

Yes .... ☐1 No............ ☐2

Q17b. Who else was present in the room with you? (Tick all that apply)

- Parent .... ☐1
- Interviewer .... ☐2
- Other adult .... ☐3
- Brother / sister .... ☐4
- Other child .... ☐5

The Economic and Social Research Institute
Whitaker Square
Sir John Rogerson's Quay
Dublin 2
Ph: 01-863 2000 Fax 01-863 2100

An Roinn Leanaí agus Gnóthaí Óige
Department of Children and Youth Affairs

University of Dublin
Trinity College
College Green
Dublin 2

GROWING UP IN IRELAND – the national longitudinal study of children

STRICTLY CONFIDENTIAL
YOUNG PERSON SELF-COMPLETE QUESTIONNAIRE ON STEP MUM / DAD'S PARTNER (DP)

AREA ☐☐☐☐ **H'HOLD** ☐☐☐

Once again, thank you for helping us with ***Growing Up in Ireland***. Remember that this is not a test and there are no right or wrong answers. We will not tell anyone the answers to your questions. But if you tell us something that makes us worried about you, then we might have to tell someone who could help.

<u>We would now like to ask you some questions about your step-Mum or your Dad's partner who lives at home with you</u>

1. How well do you get on with her?

Very well ☐1 ...... Fairly well ☐2 ...... You and your step-Mum / Dad's partner do not get on ☐3

	Strongly Disagree	Disagree	I'm in Between	Agree	Strongly Agree
2. She doesn't really like me to tell her my troubles	☐1	☐2	☐3	☐4	☐5
3. She hardly ever praises me for doing well	☐1	☐2	☐3	☐4	☐5
4. I can count on her to help me out if I have a problem	☐1	☐2	☐3	☐4	☐5
5. She spends time just talking to me	☐1	☐2	☐3	☐4	☐5
6. She and I do things that are fun together	☐1	☐2	☐3	☐4	☐5
7. She tells me that her ideas are correct and that I shouldn't question them	☐1	☐2	☐3	☐4	☐5
8. She respects my privacy	☐1	☐2	☐3	☐4	☐5
9. She gives me a lot of freedom	☐1	☐2	☐3	☐4	☐5
10. She makes most of the decisions about what I should do	☐1	☐2	☐3	☐4	☐5
11. She believes I have a right to my own point of view	☐1	☐2	☐3	☐4	☐5
12. She really expects me to follow family rules	☐1	☐2	☐3	☐4	☐5
13. She really lets me get away with things	☐1	☐2	☐3	☐4	☐5
14. If I don't behave myself, she will punish me	☐1	☐2	☐3	☐4	☐5
15. She points out ways I could do better	☐1	☐2	☐3	☐4	☐5
16. When I do something wrong, she does not punish me	☐1	☐2	☐3	☐4	☐5

Q17a. Were you alone when completing the questionnaire?

Yes .... ☐1 No............ ☐2

Q17b. Who else was present in the room with you? (Tick all that apply)

Parent .... ☐1
Interviewer .... ☐2
Other adult .... ☐3
Brother / sister .... ☐4
Other child .... ☐5

SECTION B: INTRODUCTORY LETTERS, INFOMATION LEAFLET AND CONSENT FORM USED IN THE CHILD COHORT (AT 13 YEARS)

INTRODUCTORY LETTER TO PARENTS / GUARDIANS

«PCG_title» «PCG_Fn» «PCG_sn»
«addr1»
«addr2»
«addr3»
«ADDR4»
«addr5»

Our ref:«ref»

Dear «PCG_title» «PCG_sn»,

We are writing to you about the ***Growing Up in Ireland*** study. As you may remember, your family participated in this study almost four years ago.

At that time we explained that we would like to make a return visit to your home for a follow-up interview to see how your child had changed and grown since our first visit. The second round of interviews is now about to take place and we would like to invite you to participate.

Growing Up in Ireland is the first and most important study of its kind ever to take place in this country. As well as improving our understanding of children and their development, it will help us to understand the main issues facing families in Ireland today. It will also help in providing advice to the Government on key decisions about future policies and services which will benefit all children and their families in Ireland for many years to come.

The study is being funded by the Department of Children and Youth Affairs, in association with the Department of Social Protection and the Central Statistics Office. The study is being carried out by a group of independent researchers from the Economic & Social Research Institute (ESRI) and Trinity College, Dublin.

As with your first interview, taking part in ***Growing Up in Ireland*** is entirely voluntary. All the information collected in the course of the study is treated in the strictest confidence. Your confidentiality is protected by law. No government department will have access to the information collected.

In the coming days, a member of our fieldwork team will call to your home to talk to you about the study, to explain what your participation involves and to answer any questions you may have. The enclosed information leaflet provides more details on the study.

If you have any queries about the study or your involvement in it, please do not hesitate to contact our Communications Officer (Ms Jillian Heffernan) on 01-896 3378 or any of the ***Growing Up in Ireland*** team at 01-8632000.

Thanking you in anticipation,

Yours sincerely,

James Williams
Research Professor, ESRI
Principal Investigator, ***Growing Up in Ireland***

Sheila Greene
Director, Children's Research Centre, TCD
Co-director, ***Growing Up in Ireland***

INFORMATION SHEET FOR PARENTS / GUARDIANS

INFORMATION FOR PARENTS / GUARDIANS

Almost four years have passed since you and your family kindly agreed to be part of the ***Growing Up in Ireland*** study. As you know, ***Growing Up in Ireland*** is a unique study that follows the progress of the same group of children over time to help improve our understanding of all aspects of children and their development.

We would now like to re-interview you to find out how your child has grown and changed since our last visit, almost four years ago.

A reminder about what *Growing Up in Ireland* is all about …

Growing Up in Ireland, a national, Government-funded study of children, is the first and most important of its kind ever to take place in this country.

The purpose of the study is to improve our understanding of all aspects of children and their development. It will:

- tell us how children develop over time
- help us to find out what factors affect a child's development
- look at what makes for a healthy and happy childhood and what might lead to a less happy one
- help us to discover what it means to be a parent in Ireland today

This information will help the Government to make decisions on what future policies and services will be most beneficial for children and their families in Ireland.

What has been happening since our last visit?

A total of 8,500 nine-year-old children and their families were interviewed for the first phase of ***Growing Up in Ireland***. The first report on this part of the study was published in December 2009.

We have also been busy interviewing the families of 11,000 nine-month-old infants who are also taking part in the study. A report on that part of the study was published in November 2010.

Don't forget that you can keep up to date with all our publications on our website: www.growingup.ie

Why should my family take part in the follow-up interview?

Your continued participation in the study is crucial to help get the most benefit from this research. The real value of this study will come in having *more* information on the *same* children, as this will help us to better understand the changes that take place in children's lives as they grow and, very importantly, why children grow and develop at different rates.

The information collected during the first round of interviews in the main study will be included in a series of reports. The Government can use this information to help make improvements and bring real benefits to children and families for many years to come.

Who is running the study?

Growing Up in Ireland is a Government study. The Department of Children and Youth Affairs is funding it, in association with the Department of Social Protection and the Central Statistics Office.

The Department of Children and Youth Affairs is overseeing and managing the study, which is being carried out by a group of independent researchers led by the Economic & Social Research Institute (ESRI) and Trinity College Dublin.

What happens if I take part in the follow-up interview?

Taking part in the follow-up interview is very simple and is similar to the first interview. An interviewer will contact you to arrange a visit to your home at a time that is convenient for you and your family. As with your first interview, this can be on a weekday, in the evening time if that suits, or during the weekend.

When the interviewer visits your home, you, your child and your spouse/partner (if relevant) will each be asked to fill out separate questionnaires with the interviewer. With your consent, we would also like to administer a short academic assessment test to your child – a little like a school test. This is a standard assessment used widely in research with children. It is straightforward to complete. The results of this test will be kept strictly confidential and will be used only for the purposes of the study. Individual results will not be seen by you or anyone outside the Study Team. The visit to your home will last about 1½ to 2 hours.

If you decide not to take part in the study, it will in no way adversely affect any future health or social care that you or your family will receive from the State.

Confidentiality

As with the previous interview, all the information given to the ***Growing Up in Ireland*** interviewer is treated in the strictest confidence. By this we mean that it could not be associated with you or your family by anyone other than a very small number of the people who are running the project. It will be used exclusively for research purposes.

The information given by you or any member of your family (including your child) in direct answer to the questions on the survey is strictly confidential. That information cannot be used by anyone for any purpose, other than for statistical analysis. Not even you will have access, for example, to the information given by your child. You will not receive any feedback on answers given by your child to the questions which our interviewer asks directly of him/her, regardless of what those answers might be. Similarly, the results of the academic assessment tests which your child completes will not be seen by you, your family or your child's school. However, if the interviewer observes something or is told something outside the answers given to the direct survey questions which causes him/her or the people running the Study to have serious concerns for the welfare of your child or other vulnerable person, they may have to tell someone who can help.

Under no circumstances could anyone in Government or any government agency or department be able to identify information given by you. The study is being carried out under the Statistics Act (1993). This is the same legislation as is used to carry out the Census of Population and ensures complete confidentiality of all information collected.

We will use an ID number on your questionnaire. This will help to ensure that your information is kept anonymous.

The information you provide will have your name, address and other identifying information removed. It will then be stored on a computer so that it will be available to researchers. The information can be used only for research purposes. It would be an offence to use it for any other reason.

What kind of questions will my family be asked?

Similar to our last interview, you and your partner (if relevant) will be asked questions about:

- your child's health and education
- his/her emotional health and wellbeing
- your own health
- your family life and experiences as a parent

You child will be asked questions about:

- his/her home and school life
- his/her interests and the activities he/she enjoys
- his/her relationship with you, siblings and friends

All the questions are very straightforward though some are quite detailed and some will address relatively sensitive issues such as your family's income, your relationship with your partner (if relevant) and so on. The interviewer will be able to help out if you have any concerns or questions about the actual survey questionnaire itself.

Following up in a few years' time:

At this point, it is undecided if there will be a further round of follow-up interviews. However, it is possible that we may wish to return to your household again when your child is 15 years old.

In the meantime, we will keep you up to date on the progress of the study results and the possibility of a further interview through our newsletter ***GUI News***.

Who are the interviewers?

The interviewer who will call to your home is from the Economic & Social Research Institute (ESRI). S/he is an Officer of Statistics appointed by the Central Statistics Office – similar to the interviewers who carry out research on behalf of the Central Statistics Office, including the Census.

Each interviewer carries a photo ID card.

Each interviewer has been specially trained for the study and has been vetted by An Garda Síochána.

The interviewer is not allowed to be alone with your child at any time during her/his visit to your home.

If you are unhappy with the way in which the survey has been conducted or with the interviewer, or would like to confirm her/his identity, please contact the *Growing Up in Ireland* team at 01- 8632000.

What are my rights if I take part?

- **You and your family may choose to withdraw from the study at any time, even after the interviewer has called to your home**. At that stage, if requested, we would delete all information previously collected about you.

- If there are any questions on the questionnaire you do not wish to answer, you do not have to do so.

Your participation counts …

Just as before, taking part in ***Growing Up in Ireland*** is voluntary. Your participation will play a major role in the success of the study.

It is only by carrying out studies such as these that we can understand the role of all caring adults in the life of a child and find out how we can improve the future for all children and families in Ireland.

We hope that you can support us in our work and we would like to thank you, in anticipation, for your help.

Where can I find out more information?

Phone: Freephone 1800 200 434
or contact our Communications Officer, Jillian Heffernan, on 01 896 3378
or call 01 8632000 and ask for the ***Growing Up in Ireland*** team

Visit our website: www.growingup.ie
Email us at growingup@esri.ie

Post to:
Growing Up in Ireland,
Economic & Social Research Institute,
Whitaker Square,
Sir John Rogerson’s Quay,
Dublin 2

Growing Up in Ireland
National Longitudinal Study of Children

CONSENT FORM FOR PARENTS / GUARDIANS

PARENT / GUARDIAN CONSENT FORM

Name of Child: ______________________________ Child's Date of Birth: ________________________
(BLOCK CAPITALS PLEASE)

- I have read and understand the information sheet provided. I understand that I can ask any questions I may have at any time before or during the ***Growing Up in Ireland*** study.
- I consent to my child, and myself, being included in research being conducted for the study.
- I understand that the main aim of the project is to build a bank of information about the lives of children in Ireland today and into the future.
- I understand that a range of information will be collected, including information from my child, my child's other parent, my spouse or partner (where different), as well as my child's school principal.
- I understand that, in the information provided by me and my family, our names, address and other identifying information will be removed. It will then be stored on a computer so that it will be available to researchers. The information can be used only for research purposes. It would be an offence to use it for any other reason.
- I understand that, although I will have access to the information given by me on the questionnaire which I complete, I will not have access to the information given on the questionnaires completed by my child; by my spouse/partner (if relevant); by my child's other parent (where different) or by my child's school principal.
- I will not receive any feedback about the answers given by my child to the questions which the interviewer asks directly of him/her, regardless of what those answers might be.
- I understand, however, that, if the interviewer observes something or is told something, outside the answers given to the direct survey questions, which causes him/her or the people running the study to have serious concerns for the welfare of my child, or any other vulnerable person, they may have to tell someone who can help.
- I understand that the results of the child's academic or cognitive assessment tests taken in the course of the interview are strictly confidential, and that neither I, my family nor my child's school will have access to them. They will be used only for the purposes of the study.
- I understand that, because this study looks at children's development over time, I and my child may be asked to participate in a follow-up study in a few years' time.
- I understand that I may withdraw my participation, and that of my child, at any time, including after the information has been collected.

Name of Parent/Guardian: __________________________________
(BLOCK CAPITALS PLEASE)

Address of Parent/Guardian: ____

(BLOCK CAPITALS PLEASE) ____

Signature of Parent/Guardian: ______________________ Date: ______________________

Contact telephone: __________________

If relevant:
Name of Parent/Guardian not resident in your household: ___________________________________
(BLOCK CAPITALS PLEASE)

Address of Parent/Guardian not resident in your household: ____
(BLOCK CAPITALS PLEASE) ____

Signature of Parent/Guardian not resident in your household: _________________________________

Date: ______________________ Contact telephone: ______________________

AREA: ☐ ☐☐☐ **H'HOLD:** ☐☐☐

CONSENT FORM FOR YOUNG PERSON SENSITIVE QUESTIONNAIRE

Growing Up in Ireland
National Longitudinal Study of Children

ESRI | An Roinn Leanaí agus Gnóthaí Óige / Department of Children and Youth Affairs | TRINITY COLLEGE DUBLIN

PARENT / GUARDIAN CONSENT FORM – Child Sensitive Questionnaire

Name of Child: ____________________________ Child's Date of Birth: ______________________
(BLOCK CAPITALS PLEASE)

In respect of the Child Sensitive Questionnaire:

- I consent to my child completing the questions in the Child Sensitive Questionnaire.
- I agree that the interviewer has provided me with a full and comprehensive explanation of the purpose and structure of the Child Sensitive Questionnaire and has shown me a copy of the blank questionnaire.
- I agree that I have been given an opportunity to ask any questions I may have about the Child Sensitive Questionnaire, and that these questions have been answered to my satisfaction.
- I understand that neither I nor my spouse/partner (where relevant) will have access to the information given by my child in this questionnaire.
- I understand that, as with all other parts of the ***Growing Up in Ireland*** study, I will not receive any feedback about the answers given by my child to the questions which the interviewer asks directly of him/her, regardless of what those answers might be.
- I understand that if the interviewer observes something or is told something, outside the answers given to the direct survey questions, which causes him/her or the people running the study to have serious concerns for the welfare of my child, or any other vulnerable person, they may have to tell someone who can help.

Name of Parent/Guardian: _______________________________
(BLOCK CAPITALS PLEASE)

Address of Parent/Guardian: ____

(BLOCK CAPITALS PLEASE) ____

Signature of Parent/Guardian: _____________________ Date: _____________________

Contact telephone: _________________

If relevant:
Name of Parent/Guardian not resident in your household: _________________________________
(BLOCK CAPITALS PLEASE)

Address of Parent/Guardian not resident in your household: ______________________________________

(BLOCK CAPITALS PLEASE) ____

Signature of Parent/Guardian not resident in your household: ________________________________

Date: _____________________ Contact telephone: _____________________

AREA: ☐☐☐☐ **HHOLD:** ☐☐☐

INFORMATION SHEET FOR YOUNG PERSON

YOUNG PERSON'S INFORMATION LEAFLET

Hey there!

When you were nine years old, you and your parents agreed to take part in a very important project called ***Growing Up in Ireland***. You were one of 8,500 children from across Ireland picked to be part of the study.

You may remember an interviewer from the project calling to your home to ask you some questions about what your life was like and also speaking to your mum and dad about what life as a parent is like.

Now that you have turned 13 years old, we would like to talk to you and your parents again about how things have changed in the last four years – you are much older now, have changed schools and probably have some different interests and hobbies. We would also like you to do a short maths and vocabulary test as part of the survey.

This information leaflet will remind you about what ***Growing Up in Ireland*** is about and what will happen if you agree to take part again. When you have read it, chat to you parents about what you think!

What's Growing Up in Ireland *all about?*

Growing Up in Ireland or 'GUI' is a very important study that aims to find out lots of information about children and young people living in Ireland. The Government has asked us to carry out this exciting project to find out exactly what it is like to be a young person growing up in Ireland today. We think the best way to find this out is to ask young people just like you. So we have picked 8,500 young people from all over the country and are collecting lots of information from them.

Why does the Government need to find out about young people?

This project is really important as it will help the Government to make better decisions about things that affect young people, and to make life better for all the young people and their families in the country.

Why was I picked?

All the young people picked to take part in ***Growing Up in Ireland*** were chosen at random. This was the best way to make sure we included young people from all different kinds of families and from all different parts of the country. That way we can get a complete picture of what it is like to be a young person in any part of Ireland today.

What will the study tell us?

The study will provide us with lots of information about young people's social and physical development, their education, their family, what they do with their friends, their health and so on.

The information collected will be used to advise the Government on the future policies and services that will be of most benefit to young people and their families, and that will help ensure that all families and young people can have the best possible outcomes in life.

Will this information be kept confidential?

All the information provided by you in direct answer to the questions on the survey is strictly confidential. That information cannot be used by anyone for any purpose, other than for statistical analysis. Not even your parent(s)/guardian(s) will have access to it. Similarly, the results of the Maths and Vocabulary tests will not be seen by anyone in your family or your school. However, if the interviewer observes something or is told something outside the answers you give to the direct survey questions which causes him/her or the people running the Study to have serious concerns for your welfare they may have to tell someone who can help.

What are my rights if I take part?

- You **may choose to withdraw from the study at any time, even after you have completed the questionnaire**.

- If there is any question on the questionnaire you do not wish to answer, you do not have to do so.

Your participation counts

Taking part in ***Growing Up in Ireland*** is voluntary. The participation of young people like you will play a major role in the success of the study.

It is only by carrying out studies such as these that we can understand what it is like to be a young person in Ireland today.

We hope that you will be able to help us in our work and we would like to thank you for your time completing our questionnaires.

Where can I find out more information?

Phone: Freephone 1800 200 434
or contact our Communications Officer, Jillian Heffernan, on 01 896 3378
or call 01 8632000 and ask for the ***Growing Up in Ireland*** team

Visit our website: www.growingup.ie
Email us at growingup@esri.ie

Post to:
Growing Up in Ireland,
Economic & Social Research Institute,
Whitaker Square,
Sir John Rogerson's Quay,
Dublin 2

Growing Up in Ireland
National Longitudinal Study of Children

ASSENT FORM FOR YOUNG PERSON

YOUNG PERSON'S ASSENT FORM

Name: ____
(CAPITALS LETTERS PLEASE)

Date of Birth: ____

- I would like to take part in the ***Growing Up in Ireland*** study. I have been given and have read the information leaflet, and have talked to my parents about taking part.
- I understand that my parents (or whoever looks after me) will also be interviewed, about themselves and me.
- I understand that all the information I give on the questionnaire in answer to direct interview questions is strictly confidential.
- I understand that the results of the school tests taken as part of my interview are strictly confidential and they will not be seen by my parents or by anyone in my school and will be used only in the ***Growing Up in Ireland*** study.
- I understand, however, that if the interviewer observes anything or is told something, outside the answers to direct survey questions, which causes him/her or the people running the study to have serious concerns for my welfare, they may have to tell someone who can help.
- I understand that I do not have to answer any questions that I do not want to.
- I understand that I can stop taking part in the study at any time.

Signature: ________________________________ Date: ____________________

AREA: ☐☐☐☐ **HHOLD:** ☐☐☐
