# Supplementary material for: The characteristics, life circumstances and self-concept of 13 year olds with and without disabilities in Ireland: A secondary analysis of the Growing Up in Ireland (GUI) study
Source: PLoS One. 2020 Mar 13;15(3):e0229599. doi: 10.1371/journal.pone.0229599 (PMC7069612; doi:10.1371/journal.pone.0229599)
Supplement: S2 File — (PDF) [file pone.0229599.s002.pdf]

Growing Up in Ireland
National Longitudinal Study of Children

TECHNICAL REPORT
NUMBER 1

Growing Up in Ireland
National Longitudinal Study of Children

CHILD COHORT

Design, Instrumentation and Procedures
for the Child Cohort (at 9 years)

Growing Up in Ireland

National Longitudinal Study of Children

DESIGN, INSTRUMENTATION AND PROCEDURES FOR THE CHILD COHORT

Aisling Murray, Cathal McCrory, Maeve Thornton, James Williams, Amanda Quail, Lorraine Swords, Erika Doyle, Elaine Harris.

Name	Title	Institution
Aisling Murray	Research Fellow	ESRI
Cathal McCrory	Research Fellow	ESRI
Maeve Thornton	Research Fellow	ESRI
James Williams	Research Professor and Principal Investigator, Growing Up in Ireland	ESRI
Amanda Quail	Research Fellow	ESRI
Lorraine Swords	Research Fellow (Children's Research Centre)	TCD
Erika Doyle	Research Fellow (Children's Research Centre)	TCD
Elaine Harris	(formerly) Research Fellow (Children's Research Centre)	TCD

The views expressed in this report are those of the authors and do not necessarily reflect the views of the funders or of either of the two institutions involved in preparing the report.

ESRI

Trinity College Dublin

Office of the Minister for Children and Youth Affairs
Oifig an Aire Leanaí agus Gnóthaí Óige

Copyright © Minister for Health and Children, 2010

Office of the Minister for Children and Youth Affairs
Department of Health and Children
Hawkins House
Hawkins Street
Dublin 2
Tel: +353 (0)1 635 4000
Fax: +353 (0)1 674 3223
E-mail: omc@health.gov.ie
Web: www.omc.gov.ie

All rights reserved. No part of this publication may be reproduced, stored in a retrieval system, or transmitted, in any form or by any means, electronic, mechanical, photocopying, recording or otherwise, without the prior permission in writing of the copyright holder.

For rights of translation or reproduction, applications should be made to the Head of Communications, Office of the Minister for Children and Youth Affairs, Hawkins House, Hawkins Street, Dublin 2, Ireland.

TABLE OF CONTENTS

CHAPTER 1: INTRODUCTION 8
1.1 Introduction 8
1.2 Background and Objectives 8
1.3 Conceptual Framework 11
1.3.1 Summary of Conceptual Framework 11
1.3.2 From Conceptual Framework to Instrumentation 13
1.4 Structure of Report 15

CHAPTER 2: METHODOLOGY/SAMPLING 18
2.1 The Population and Sampling Frame 18
2.1.1 Structure of the National School Sector in Ireland 19
2.1.2 Level of Coverage 22
2.2 Sample Design 23
2.3 Refusal Conversion 24
2.4 Reweighting the Survey Data 25

CHAPTER 3: INPUT TO INSTRUMENTS 27
3.1 Scientific and Policy Advisory Committee 27
3.2 Delphi Process 28
3.2.1 Panel Selection 28
3.2.2 Forward information to the panel of experts 28
3.2.3 The Instrument used in Round One 28
3.2.4 Field Procedures 29
3.3 Children's Advisory Forum 29
3.3.1 The Structure of the CAF Meetings 29
3.3.2 Input of the CAF 31
3.4 Expert Panels 31
3.5 Stakeholder Groups 32
3.6 Other Longitudinal Studies 33
3.6.1 Millennium Cohort Study (MCS) 33
3.6.2 Growing Up in Australia (LSAC) 33
3.6.3 National Longitudinal Survey of Children and Youth (NLSCY) 34
3.6.4 Early Childhood Longitudinal Study (ECLS) 34
3.6.5 Avon Longitudinal Study of Parents and Children (ALSPAC) 34

CHAPTER 4: ETHICAL CONSIDERATIONS 36
4.1 Relevant Acts 36
4.1.1 Data Protection Act 1988, 2003 36
4.1.2 Statistics Act (1993) 36
4.2 Practical Application of Ethical Considerations 37
4.2.1 Informed Consent 37
4.2.2 Reporting Concerns 37
4.2.3 Interviewers Being Alone with Children 37
4.2.4 Confidentiality 37
4.2.5 Avoidance of Embarrassment/Distress 38
4.3 Ethics Committee 38

CHAPTER 5: OVERVIEW OF INSTRUMENTS AND PROCEDURES 40
5.1 School and Pupil Recruitment 40
5.2 Fieldwork and Instruments in the Schools 40
5.3 Household-based Fieldwork and Participation of the Family 41
5.4 Special Procedures 42
5.4.1 Disability 42
5.4.2 Literacy 43
5.4.3 Other languages 43
5.4.4 Twins and Triplets 43
5.5 CAPI Procedure 43

5.6 Gifts to Respondents 44

CHAPTER 6: INSTRUMENTS USED IN THE SCHOOL 46
6.1 School Record Sheet 46
6.2 Principal's Questionnaire 46
6.3 Teacher-on-Self Questionnaire 49
6.4 Teacher-on-Pupil Questionnaire 50
6.5 Direct Assessments of Children 51
6.5.1 Piers-Harris Children's Self-Concept Scale (2nd Edition) 51
6.5.2 Drumcondra Tests in Reading Vocabulary and Maths 51

CHAPTER 7: INSTRUMENTS COMPLETED BY MOTHER/LONE FATHER AND FATHER/PARTNER 53
7.1 Mother/Lone father questionnaire 53
7.1.1 Section A – Background information 53
7.1.2 Section B - Child's Health 54
7.1.3 Section C. Child's Use of Health Services 56
7.1.4 Section D – Child's Diet and Exercise 57
7.1.5 Section E – Respondent's Health 59
7.1.6 Section F – Respondent's Lifestyle 59
7.1.7 Section G – Child's Activities 60
7.1.8 Section H – Child's Emotional Health and Wellbeing 61
7.1.9 Section J – Child's Education – Past and Current 61
7.1.10 Section K – Family Context 63
7.1.11 Section L – Sociodemographics 64
7.1.12 Section M – Neighbourhood and Community 66
7.2 Father/Partner Questionnaire 67
7.2.1 Section A – Introduction 67
7.2.2 Section B – Respondent's Health 67
7.2.3 Section C – Respondent's Lifestyle 67
7.2.4 Section D – Family Context 67
7.2.5 Section E – Sociodemographics 68
7.3 Sensitive Supplementary Section 68

CHAPTER 8: INSTRUMENTS COMPLETED BY CHILDREN 71
8.1 The Child's Questionnaire 71
8.2 Child Main Questionnaire 71
8.2.1 Section A: School 71
8.2.2 Section B: Food 72
8.2.3 Section C: Activities 72
8.2.4 Section D: Likes and Dislikes 74
8.3 Sensitive Questionnaires for Nine-Year-Old Children 74
8.3.1 Core Sensitive Questionnaire 75
8.3.1.1 Section A: Where You Live 75
8.3.1.2 Section B: School 75
8.3.1.3 Section C: Family 76
8.3.2 Supplementary Sensitive Questionnaires 76
8.4 Self-Completion Time Use Diary 77

CHAPTER 9: OTHER INSTRUMENTS 80
9.1 Non-resident parent questionnaire 80
9.2 Carer Questionnaires 82
9.2.1 Home-Based Carer Questionnaire 82
9.2.2 Centre-Based Carer Questionnaire 83
9.3 Other Instruments 84
9.3.1 Follow-up Information 84
9.3.2 Height and Weight of Main Respondents 84
9.3.3 GPS Co-ordinates 85
9.3.4 Work Assignment Sheet 85

9.3.5 Interviewer Observations 85

CHAPTER 10: SCALES AND OTHER STANDARDISED MEASURES 87
10.1 Scales 87
10.1.1 Reliability 87
10.1.1.1 Internal consistency 87
10.1.1.2 Test-retest reliability 88
10.1.1.3 Split Half reliability 88
10.1.2 Validity 88
10.2 Measures Used in the School 89
10.2.1 Piers-Harris Children's Self-Concept Scale, 2nd Edition (Piers-Harris 2) 89
10.2.2 Strengths and Difficulties Questionnaire (SDQ) 90
10.3 Drumcondra Maths and Reading Tests 92
10.3.1 Drumcondra Primary Maths Test – Revised – DPMT-R LEVEL 3 92
10.3.2 Differences in Revised Level 2 Maths Test (DPMT-R) compared to Level 3 94
10.3.3 Differences in Revised Level 4 Maths Test (DPMT-R) compared to Level 3 94
10.4.1 Drumcondra Primary Reading Test – Revised – DPMT-R LEVEL 3 95
10.4.2 Differences in Revised Level 2 Reading Testcompared to Level 3 96
10.4.3 Differences in Revised Level 4 Reading Test compared to Level 3 96
10.4 Measures Used in the Home 96
10.4.1 Basic Deprivation Scale 96
10.4.2 Centre for Epidemiological Studies Depression Scale (8-item) (CESD-8) 97
10.4.3 The Seven-Item Short Form of the Dyadic Adjustment Scale (DAS-7) 99
10.4.4 Emotionality, Activity and Sociability Temperament Questionnaire (EAS) 100
10.4.5 Parenting Style Inventory – II (PSI-II) 101
10.4.7 The Strengths and Difficulties Questionnaire (SDQ) 102
10.5 Relationship of Scales and Standardised Measures to Child Outcomes 103

CHAPTER 11: TRIANGULATION AND DISTRIBUTION OF VARIABLES 106
11.1 Triangulation 106
11.2 Distribution of Outcomes and Explanatory Variables 109
11.3 The Longitudinal Approach 111

CHAPTER 12: SUMMARY 114

APPENDICES – TABLE OF CONTENTS

Appendix A	Information Sheet for Principals<br>Information Sheet for Teacher
Appendix B	Information Sheet for Parents<br>Information Sheet for Child<br>Consent Form for Parents<br>Consent Form for Child
Appendix C	School Record Sheet
Appendix D	Principal's Questionnaire
Appendix E	Teacher-on-Self Questionnaire
Appendix F	Teacher-on-Pupil Questionnaire
Appendix G	Mother/Lone Father Questionnaire – incl. Sensitive Section
Appendix H	Father Questionnaire – incl. Sensitive Section
Appendix I	Child Questionnaire – incl. Sensitive Sections
Appendix J	Non-resident Parent Questionnaire
Appendix K	Carer Questionnaires
Appendix L	Time-Use Diary
Appendix M	Questionnaires for 'Second' Twin
Appendix N	Work Assignment Sheet
Appendix O	Drumcondra English Reading Test – Level 3
Appendix P	Drumcondra Mathematics Test – Level 3
Appendix Q	Piers-Harris Children's Self-Concept Questionnaire 2

Chapter 1

INTRODUCTION

CHAPTER 1: INTRODUCTION

1.1 INTRODUCTION

Growing Up in Ireland – *the National Longitudinal Study of Children* is a study of the factors which contribute to or undermine the wellbeing of children in 21st Century Ireland. The project involves studying two main cohorts of children with a view to improving our understanding of their development across a range of domains. The first cohort, the child cohort, focuses on nine-year-olds, the second, the infant cohort, on infants of nine months of age. The child cohort is based on a nationally representative sample of 8,500 nine-year-olds and the infant cohort on a national sample of 11,000 infants and their families. The survey is longitudinal in nature with both cohorts being interviewed twice over the course of the project. The older cohort and their parents/guardians are interviewed at 9 and at 13 years of age. The parents of the infant cohort are interviewed when their children are nine months old and subsequently when they are three years old.

The child cohort represents 8,500 children who were born between 1 November 1997 and 31 October 1998. Data collection for this group took place between August 2007 and May 2008. The child cohort is made up of the families of 11,000 children. The first wave of data collection for that group took place from September 2008 to the end of March 2009.

This report describes in detail the design, instruments and procedures used only in respect of the child cohort. The focus is on the nature and content of the questionnaires and other instrumentation, along with a general consideration of operational procedures. The infant cohort is the subject of a parallel set of reports.

In the current chapter, we provide the context for the rest of the document. We begin by describing the background and objectives of the study, our interpretation of its requirements and how these have been met by the Study Team. We then move on to a brief summary of the conceptual framework underlying ***Growing Up in Ireland*** and how this is reflected in the instrumentation.

1.2 BACKGROUND AND OBJECTIVES

Growing Up in Ireland provides a very important input to the implementation of *The National Children's Strategy* – a major national plan for children, published in 2000 by the Department of Health and Children. The principal objective of the study is to provide evidence-based research addressing the wellbeing of children and childhood. This increased understanding of the determinants and drivers of wellbeing and its change and transformation over time will be used to assist in policy formation and the design and delivery of services for children and their families as set out in the National Children's Strategy (2000). ***Growing Up in Ireland*** is a key element in the Strategy, especially with regard to its second goal which notes *that* 'Children will be better understood; their lives will benefit from evaluation, research and information on their needs, rights and the effectiveness of services'.

Growing Up in Ireland has been commissioned by the Irish Government. It is funded by the Department of Health and Children through the Office of the Minister for Children (OMC), in association with the Department of Social and Family Affairs and the Central Statistics Office. Detailed recommendations for the design of a National Longitudinal Children's Study were first presented in a paper entitled *Design of the National Children's Strategy – Longitudinal Study of Children (Collins, 2001)*. The current study stems from a Request for Tender which was issued by the Department of Health and Children in December 2004. After an assessment and evaluation process throughout 2005 and early 2006, work on the project

began in April 2006 by a research consortium led by the Economic and Social Research Institute (ESRI) and Trinity College, Dublin (TCD).
Growing Up in Ireland is designed to describe and analyse what it means to be a child in Ireland today and to understand the factors associated with children's wellbeing, including those impacting on their physical health and development, their social/emotional/behavioural wellbeing, and their educational achievements/intellectual capacities. While children's current wellbeing is of immense importance, researchers are also cognisant of the future outcomes for the child as they develop into young adults. The longitudinal nature of the project facilitates the recording of current data with a view to using them to assist in understanding future outcomes. By gathering comprehensive data on childhood development the study will provide a statistical basis for policy formation and applied research across all aspects of children's development – currently and into the future.

The Study has nine over-arching objectives[1]. Each of these, with the Study Team's interpretation, is set out below.

1. **To describe the lives of Irish children, to establish what is typical and normal as well as what is atypical and problematic**
   At each data wave we attempt to identify the developmental status of the children sampled in relation to all the key indicators of wellbeing, quantitative and qualitative. The variability on key indicators and determinants of variability is critical to this, with a view to defining, for example, normality, borderline problematic status and problematic status. In doing so we intend to compare children in Ireland with international norms and, where available, their indicators of developmental status with those of their international peer-group

2. **To chart the development of Irish children over time, to examine the progress and wellbeing of children at critical periods from birth to adulthood**
   Within the confines of the initial seven-year period set out for the project, the Study Team will attempt to identify those changes that occur between data waves on key indicators, and to identify the developmental trajectories of markers of child development and wellbeing. A key consideration of this is the variability in the rate of progression of children in the cohort. Aside from critical normative events and transitions (e.g. starting primary school) issues addressed will include what has occurred to the children in relation to non-normative life events (such as parental death and separation).

3. **To identify the key factors that, independently of others, most help or hinder children's development**
   This involves the identification of the factors which are most strongly correlated with child wellbeing and to investigate whether these factors are child-and/or environmentally-oriented. A key aspect of the conceptual framework underlying ***Growing Up in Ireland*** is the interaction between individuals and their environments that result in variations of outcomes: the environment not only acts on the child but the child also affects change in his/her environment. This framework also acknowledges the importance of identifying moderating and mediating variables, as well as the influence of the timing of particular events.

4. **To establish the effects of early child experiences on later life**
   The primary focus with regard to the child cohort will be based on retrospective data, principally recorded from the child's parents/guardians.

[1] *Request for Tenders (RFT) for Proposals to Undertake a National Longitudinal Study of Children in the Republic of Ireland*, issued by the National Children's Office of the Department of Health and Children and the Department of Social and Family Affairs, December 2005, p. 20.

The issues involved here relate to those factors and circumstances in the early years of life which predict to good or poor outcomes at the later stages of development – middle childhood and beyond.

5. **To map dimensions of variation in children's lives**
To fully map out the dimensions of variation in children's lives we will describe the nature, range and patterns of distribution of all variables. This will include a consideration of the variability within the cohort in developmental status, progression and outcomes, and, in particular, how variables such as class, family structure, gender, level of educational attainment of parent(s), ethnicity, etc. predict to differences in developmental progress and outcomes.

6. **To identify the persistent adverse effects that lead to social disadvantage and exclusion, educational difficulties, ill health and deprivation**
The work of Rutter and Bergman (1988) and others on using longitudinal data to understand psychosocial risk will be particularly useful in framing specific questions in this field. In particular, we aim to provide an appropriate range of variables to facilitate the identification of factors, operating singly or in combination, which are associated with negative outcomes for children. This should allow us to identify whether or not there are factors or combinations of factors which predict specific types of negative outcomes, such as social disadvantage and exclusion, educational difficulties, ill-health and deprivation. This in turn will permit us to address whether or not there are different pathways to similar negative outcomes, and to isolate those categories of children and their characteristics that are most at risk for adverse development.

7. **To obtain children's views and opinions on their lives**
Children's views will be elicited through questions amenable to quantitative analysis but also, importantly, through open-ended questions that give expression to children's voices and allow them to give their views, and talk about their experiences using their own words. In order to capture the richness of children's experience of their worlds, the study incorporates a nested qualitative study with a particular focus on the use of methods which can elicit children's experience, perspectives and voice. In particular, the Study Team will address what children in Ireland think are the important issues in their lives, what is their experience of family life and what is their experience of the key institutions that impinge on their daily lives, for example school, neighbourhood, church, etc.[2]

8. **To provide a bank of data on the whole child**
Although the current report focuses only on the quantitative component of the older cohort, the study also involves substantial qualitative elements. A longitudinal qualitative sample of 120 households (nine-year-olds and their families) will be included in the study. By combining quantitative and qualitative approaches we will be able to add significantly to the overall bank of data available on the whole child in 21st Century Ireland. The qualitative study will be the subject of a separate report.

9. **To provide evidence for the creation of effective and responsive policies and services for children and families**
The focus of the project throughout will be generating evidence through research, with a view to making the information available to policy makers to assist them in the formation of child-oriented policies and to target services more accurately at all

[2] The qualitative studies carried out with both cohorts are the subject of other reports in this series.

children and families in Ireland, particularly those most in need of assistance (in the broadest sense of the term).

1.3 CONCEPTUAL FRAMEWORK

1.3.1 SUMMARY OF CONCEPTUAL FRAMEWORK[3]

The study is adopting a dynamic systems perspective founded upon five insights from different disciplines: (i) ecology, (ii) dynamic connectedness, (iii) probabilism, (iv) period effects, and (v) the active role or agency of the child in the developmental process. The bioecological model of Urie Bronfenbrenner (Bronfenbrenner 1979; Bronfenbrenner and Morris, 2006) is a key tool in creating this perspective.

The child's relationships operate both within and outside the household, in the school, through the workplace, and in the wider community. As discussed in detail in GUI Literature Review Series Paper No. 1 and summarised in Figure 1.1 below, Bronfenbrenner illustrates the intimate relationship between the *microsystem*, the face-to-face interactions which the child experiences, and the *mesosystem*, which encompasses the links between the different actors in the micro-system, i.e. the relationship between parents, between home and school or between close family and extended kin.

Outside the mesosystem in Bronfenbrenner's model sits the *exosystem*. This comprises the structures, institutions and settings that, whilst not in direct contact with the child, exert an important influence upon his/her quality of life and outcomes. Examples of determinants within the exosystem would be the Departments of State that have an important impact on child wellbeing in areas such as education, health or welfare. The last ring of Bronfenbrenner's schema is the *macrosystem*, which consists of the culture-specific ideologies, attitudes and beliefs that shape the society's structures and practices. Together these different levels provide a taxonomy of factors that may influence the experiences and wellbeing of a child as he/she develops from birth to adulthood.

Figure 1.1: Bronfenbrenner's Ecological Perspective on Child Development

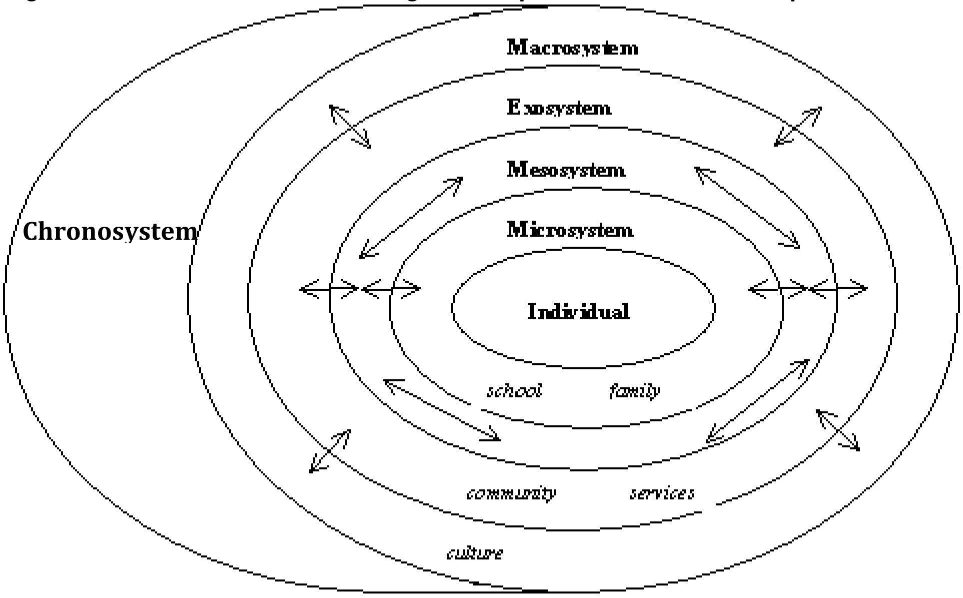

[3] A detailed discussion of the conceptual framework used in the study is the subject of GUI Literature Review Series Paper No.1

Figure 1.2 provides a schematic summary of the study's overall view of the complex multi-directional and recursive relationships between the child on the one hand and, on the other, the environments and actors within which and with whom he/she operates, relates and interacts.

Hypothesised relationships between Child Characteristics, Child Outcomes and Contextual Variables in *Growing Up in Ireland*

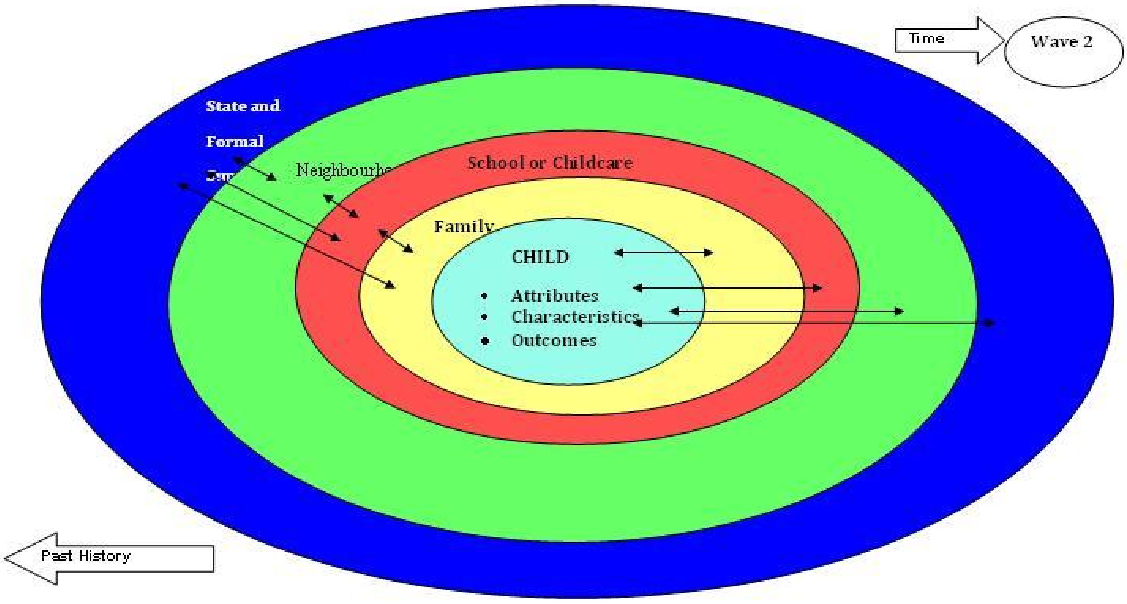

	Child characteristics and outcomes		School/Childcare Characteristics and Child's relationships within School or Childcare		State and Formal Structures<br><br>Children's contact with these structures/services/institutions
	Family Characteristics and Child's Close Relationships		Neighbourhood characteristics and Child/Family's interaction with neighbourhood		

From this one can see that we extend outward from the individual child to: close relationships in the home and the school (*microsystem*), to the relationship between the elements of the microsystem such as between parents (*mesosystem*), to the institutions and settings that influence the microsystem such as health services (*exosystem*), and finally to all the actions and interactions take place under the influence of more global forces such as cultural beliefs and general economic prosperity (*macrosystem*). Table 1.1 gives examples of variables used in ***Growing Up in Ireland*** that are relevant to each layer in Bronfenbrenner's bioecological model, along with relevant section headings indicating where each variable is discussed within the current report.

Table 1.1: Examples of variables in Growing Up in Ireland in each layer of the bioecological model

Layer	Example Variables (and section numbers)
Child – centre of the system	Temperament (7.1.8), Health (7.1.2), Gender, Academic ability (10.3), Ethnicity (7.1.11)
Microsystem – face-to-face interactions experienced by the child, school and family	Parent's attributes in relation to Health (7.1.5), Education (7.1.11), Lifestyle (7.1.6), Parenting style (8.3.2), Size of household (7.1.1), Family structure (7.1.1), Child's relationship with peers including bullying (7.1.9, 8.3.1), Characteristics of classroom and teacher (6.3)
Mesosystem – links between actors in the microsystem i.e. between parents, home and school, close and extended family, family-work spaces, etc.	Parents' marital relationship (7.3), Parental involvement with school (6.4), Parental involvement with people in community including extended family (7.1.12), Parental occupation (7.1.11) and work-life balance (7.1.10)
Exosystem – formal and informal State, community and neighbourhood structures, institutions and settings which impact on child	Access to health care, school policy (6.2), Social welfare support (7.1.11)
Macrosystem – cultural-specific ideology, attitudes, beliefs, social mores, etc.	Citizenship/nationality (7.1.11), Church and religion (7.1.10), supplemented by information form other sources such as Government policy documents

1.3.2 FROM CONCEPTUAL FRAMEWORK TO INSTRUMENTATION

The project has been designed to record details about the array of factors which have been previously identified or hypothesised as having an influence on the child's developmental outcomes in all spheres of his/her life. As noted by Sanson et al. (2005), 'an outcome is an attribute of the child at a particular point in time' (p.5). Outcomes will generally be influenced by a range of inputs, a few of the more important of which include parenting, education and the health services. Furthermore, children's own attributes, and their behaviour and attitudes will also act as influences on later outcomes. The child with positive behaviours and attitudes may elicit a very different parenting style than those with more negative ones. This, in turn, will impact on subsequent outcomes. As outlined in GUI Literature Review Series Paper No. 1 (the Background and Conceptual Framework) the child outcomes focused on in ***Growing Up in Ireland*** are

- physical health and development
- social/emotional/behavioural wellbeing
- educational achievement and intellectual capacity.

As will be described in detail in Chapters 5 to 10 below, the questionnaires were structured in such a way as to record the relevant information from the various layers of the Study Child's world, including the child's personal relationships with the family, the school, the neighbourhood, etc., and taking account of formal and informal supports in each case.

In adopting the 'whole child' perspective and a rounded view of child wellbeing, it was clearly impossible to record everything that was desirable. Choices had to be made as to what measures and variables were included and excluded. The criteria used in making decisions on inclusion/exclusions were as follows

- *Importance:* Were there scientific grounds for believing that the variable exerts a substantial influence on one or more outcomes or dimensions of child?

- *Measurability:* Could the variable be validly, reliably and ethically measured using the methods of large-scale survey research?

- *Policy relevance:* Was the variable actionable through public policy?

- *Policy urgency:* Was it acknowledged that the area of public policy to which the variable was relevant needed an evidence base for reform?

- *Prevalence and variance:* Was the variable sufficiently prevalent in the population to yield an analyzable level of variance in the available sample?

- *Added value:* Did the variable relate to influences on child wellbeing that were inadequately covered by other research.

Selection of outcome and input variables had to be followed by the selection of indicators that would operationalise and measure the information in question, since these indicators can be interpreted in a range of different ways. The criteria used to select indicators were as follows:

- *Robustness:* Did the indicator provide a measure of the construct/variable of interest that had been proven to be valid and reliable? With this in mind we attempted to concentrate on items that had been previously tested in survey work, particularly in longitudinal cohort studies.

- *Ethical acceptability:* Did the indicator meet relevant ethical standards as set by the review process?

- *Acceptability to respondent:* Would the indicator be likely to deter participation or increase attrition among the study respondents by increasing response burden?

- *Age appropriateness:* Were age-appropriate variants of the indicator available or could they be designed, taking account of the need to maintain consistency in measurement across cohorts and across time?

- *Time efficiency:* Did the indicator involve as little interview time as possible, taking account of the importance of the variable and the requirement for robust measurement?
- *International use:* Was the indicator successfully used in research in other countries, particularly in comparable studies such as the UK Millennium Cohort Study and Growing Up in Australia?

- *Use in Ireland:* Was the indicator successfully used in previous research in Ireland?

- *Value for target setting/impact assessment:* Could the indicator be used to set targets for policy and/or to measure the impact of policy interventions?

The individual child is clearly the key participant in ***Growing Up in Ireland***. Not only were parents/guardians interviewed about the child, but the child's own experiences, views and opinions were also sought in a personal interview with him/herself. Furthermore, children, through their participation in the Children's Advisory Forum, were important contributors to the development of the instruments used in the study (see Section 3.3 for further details).

The Study Team has also sought to record information from as many other informants as possible in the various environments in which the child operates. As well as interviewing the parental figures in the home, ***Growing Up in Ireland*** gathered information directly from the child's teacher and principal, and non-resident parents and regular carers (where appropriate).

The broad range of information gathered in the study reflects the acknowledged importance of the proximal and distal contexts in the child's life. Information has been gathered about the child's health, education, activities, family relationships, temperament, access to service and opportunities, and the local area. Information was also gathered about parental health, education and ethnicity, thus facilitating consideration of the influence of parental characteristics and behaviour on the child's development. Collecting data on significant events in the child's life, and the longitudinal aspect of the study, will contribute to research on individual pathways and trajectories. It should be noted, however, that the longitudinal approach is particularly valuable where there are three or more data collection points. The geocoding of children's homes will provide researchers and others with the potential to look at the impact of various environmental conditions on child outcomes in the future.

1.4 STRUCTURE OF REPORT

As noted above the main objectives of this report are as follows

- to outline the sample design and explain the procedures for respondent selection
- to describe the broad outline of how the instruments were developed, including a discussion of the main inputs to instrumentation from the Scientific and Policy Advisory Committee, the Delphi consultation process, the Children's Advisory Forum, and the Panels of Experts being coordinated by the Study Team
- to discuss the ethical review procedures for the study
- to describe fieldwork procedures
- to provide a detailed breakdown of the main instruments used at all levels of the study including the broad domains of interest, specific variables of interest, and information on scales used in the study, along with a rationale for the use of each
- to present, in the appendices, the various instruments and related documents used in the study. (Appendices are bound separately in an accompanying document.)
- to provide a platform or reference point for subsequent waves of the study in terms of operational procedures and substantive input, providing, hopefully, a benchmark against which change and improvement in subsequent rounds of the survey may be measured.

To this end, the report has 10 subsequent chapters. Chapter Two summarises sample design and sampling. Chapter Three outlines the inputs to the instrumentation from various advisory groups and other interested parties. Chapter Four looks at ethical considerations, in particular the ethical review procedure. In Chapter Five we present a broad overview of the various levels of instruments and questionnaires used in the survey aspect of Wave One of the child cohort. Subsequent chapters are divided into the main areas and units of data capture. Chapter Six details all of the instruments used at the school level. This includes a review of the School Record Sheet – the document used by the school to generate the names of eligible children for inclusion in the population under study. It also details the nature and contents of the individual questionnaires implemented with the school principal and teachers, as well as the academic assessment tests used. Chapter Seven considers, in detail, the main questionnaire used in the study – the Mother/Lone Father instrument. Chapter Eight outlines the various levels of instrumentation administered to the child. Chapter Nine summarises all of the other instruments used, including those sent to the non-resident parents, the non-cohort givers, the direct measurement of height and weight and, the GPS coordinates of respondents by the interviewer. Chapter Ten presents a discussion of the scales and other standardised measures used in the project. Finally, a concluding chapter is presented in Chapter Eleven.

Chapter 2

METHODOLOGY/SAMPLING

CHAPTER 2: METHODOLOGY/SAMPLING

In this chapter we outline the methodology and sample design issues associated with the project. We begin by considering some sampling frame issues followed by a discussion of the population of schools and pupils in Ireland. We then discuss the sample design itself, before moving on to the procedures for refusal conversion. Finally we outline our plans for reweighting the data prior to analysis.

2.1 THE POPULATION AND SAMPLING FRAME

Our overall objective was to interview a random sample of 8,500 nine-year-old children and their parents/guardians. The sample design requirements further specified that the sample should be regionally representative with no spatial bias, consequently no over-sampling or booster sampling of special groups was required. There was a total resident population of 56,500 nine-year-olds registered in the Census of Population in 2006, so a sample size of 8,500 represents approximately 14%, or about one in every seven of the nine-year-olds resident in the country.

As with all sample design strategies, the first issue was the identification of an appropriate sampling frame. Ideally, the population frame should contain all nine-year-old children who are resident in Ireland with each valid element in the population being registered only once. A number of alternative frames were considered in the early stages of the study including the feasibility of linking to the Child Benefit Register[4] (CBR). Following discussions with the Department of Social and Family Affairs and others, however, it was decided that other sources had more potential in offering comprehensive and up-to-date contact details in respect of the child cohort. One such approach was to use the national education system as a point of entry to the cohort in question. Based on data provided by the Department of Education and Science, a comprehensive listing of all schools (both public and private) was generated. In addition to detailing the total number of enrolments in each school by age and gender, this database also recorded information on the characteristics of the school such as region, disadvantaged status, size, school type, denominational status, and gender mix. These classificatory variables were important for pre-stratification purposes prior to sample selection, which were, in turn, important for meeting the study's objectives of describing the lives of children (#1), mapping dimensions of variation in children's lives (#5) and identifying persistent adverse effects that lead to educational difficulties (#6).

In addition to providing a comprehensive record of nine-year-old children, the National School System offered a number of other operational and analytical benefits over other sampling frames, such as the Child Benefit Register. Using the school as the primary unit allowed for direct access to the principal and teachers, who were key study informants, and facilitated the completion of the school and teacher questionnaires and related classificatory variables on the child's school environment. Secondly, it facilitated the completion of the academic achievement tests in a group self-completion setting, thus reducing respondent burden and contact time in the home. Thirdly, natural clustering afforded by the primary school system (as most pupils will live within a relatively restricted geographical catchment area) provided an opportunity for modelling and identifying multi-level effects at the community, school, class, family, and child levels.

The sample design for the child cohort in ***Growing Up in Ireland*** was based on a two-stage selection process in which the school was the primary sampling unit with the children within school being the secondary units. In the context of this study the school forms a very efficient natural clustering of children and so substantially facilitates sample selection and processing. It is also efficient from a

---

[4] Child Benefit is a social welfare entitlement which is payable to the parents or guardians of children under 16 years of age, or under 19 years of age if the child is in full-time education, youth training or has a disability. It is payable to parents/guardians who are 'habitually resident' in the State. Non-EU/EEA citizens who are legally working in Ireland may qualify if the child is also resident in the State.

resource perspective. As noted by Kish (1965), a number of criteria should be met in implementing a two-stage design:

- One should attempt to maximise the number of Primary Sampling Units (schools in our case). We sampled from over 850 of the 3,200 primary level schools in the country.
- The units should have clear and distinct boundaries. This caveat usually applies in area-based clustered samples where boundary definition often becomes an issue. In Growing Up in Ireland the PSU is the school, which has clearly defined boundaries.
- There should be uniformity of size among the PSU's. This is not possible with the schools, though there is quite a degree of uniformity among the majority of schools with 68% having less than 40 nine-year-olds. Selection of larger schools with probability proportionate to size and with the upper threshold of 40 pupils per school attenuates the lack of uniformity of size.
- PSUs should be well recognized administrative units. The school is clearly a well recognized and discrete administrative unit.

In selecting PSUs one should attempt to ensure that they reflect the full range of variability as is encountered in the population as a whole, whilst simultaneously attempting to ensure that they are as homogeneous as possible across all PSUs. In other words, the PSUs should be set up in such a way as to maximize within-cluster heterogeneity and also between-cluster homogeneity (making each PSU as similar to each other in terms of variability and variance as possible). The closer these two criteria are met the closer the design effects will be to unity. In the context of ***Growing Up in Ireland*** it is clearly difficult to influence the heterogeneity of the schools (PSUs). Given that schools have a relatively restricted geographical hinterland, the full variability of children's characteristics across the population as a whole will not, necessarily, be reflected in the PSUs. Nonetheless, this has been counterbalanced by the number of PSUs (schools) selected.

In the context of the older cohort in ***Growing Up in Ireland*** there was, in fact, very little alternative in terms of sample design. The schools provide such a naturally occurring clustering of pupils that it would have been difficult to justify any other design. In addition to efficiency arguments, the use of the school as the PSU will substantially facilitate multi-level modelling to identify significant effects at the individual, family, school, and neighbourhood levels.

2.1.1 STRUCTURE OF THE NATIONAL SCHOOL SECTOR IN IRELAND

The primary education sector in Ireland (known as the National School sector) is made up of three different types of school. The major constituent is the 3,160 mainstream National Schools which are supported by the Department of Education and Science. There is an additional group of just over 120 special schools which draw their funding from the Department of Education and Science, and have an enrolment made up of children with learning or physical disabilities, children of traveller families and children with other special needs. Finally, there is a third group of privately funded National Schools (just over 40) which are fee-paying and, as such, do not receive financial subvention from the Department of Education and Science.

Table 2.1 summarises the distribution of primary schools in Ireland according to type of school and number of nine-year-olds. From Section B of the table one can see that a large proportion of schools contain a relatively small number of nine-year-olds. In the mainstream sector 20.8% have 1-5 nine-year-olds, a further 23.4% have 6-10 nine-year-olds, and so on. The table shows that almost two-thirds of mainstream national schools (62.7%) have 15 or fewer nine-year-olds. At the other end of the distribution only 8.7% of mainstream schools had more than 40 pupils aged nine years.

Table 2.1: Distribution of 3 main types of national schools in Ireland (2005/2006 academic year)

	**A. Number of Schools**			**B. Percentage of Schools**		
**No. of 9 year old**	**Mainstream**	**Special**	**Private**	**Mainstream**	**Special**	**Private**
Zero	116	33	14	3.7%	26.6%	33.3%
1-5	656	64	6	20.8%	51.6%	14.3%
6-10	739	23	4	23.4%	18.5%	9.5%
11-15	467	3	4	14.8%	2.4%	9.5%
16-30	695	1	10	22.0%	0.8%	23.8%
31-40	212	0	1	6.7%	0.0%	2.4%
41-50	91	0	1	2.9%	0.0%	2.4%
51+	184	0	2	5.8%	0.0%	4.8%
Total schools	3160	124	42	100%	100%	100%
Schools with 9 year olds	3,044	91	28			

This highly skewed distribution of schools in terms of number of nine-year-olds is even more apparent in the special and private school sectors. As many as 26.6% of Special Schools and 33.3 % of the 42 Private Schools had no nine-year-olds in 2005–6. A total of 96.7% of Special Schools contained 10 or fewer nine-year-olds. Similarly, 66.6 % of Private Schools contained 15 or fewer relevant pupils.

A further interesting aspect of Table 2.1 is the total of 116 Mainstream Schools, 33 Special Schools and 14 Private Schools which record having no nine-year-old pupils. We have most comprehensive information in respect of the Mainstream Schools and so can explore the nature of the 116 schools in question. We find that 80 of these are classified by the Department of Education and Science as 'Infants Only'. These 80 can reasonably be excluded from the valid target sample of schools for selection purposes. The other 30 schools which have no children within the age range were small schools which simply did not have any nine-year-olds in the 2005–6 academic year but which may have had some in subsequent school years. Accordingly, although their exclusion would not have any significant (or any) impact on the overall sample, we included them in the population frame used for sample selection, albeit with a high probability of including schools which would not in fact contain valid pupils within the age range. Inclusion of such schools did not adversely impact on the statistical structure of the sample. In statistical terms these are 'dead-wood' elements in the population. They did, of course, have resource implications in so far as interviewers as well as Head Office staff were contacting and approaching schools with no children within the age range.

We had no information on the 33 Special Schools or the 14 Private Schools in terms of being 'Infants Only' or otherwise. The 33 Special Schools which had no nine-year-old pupils probably reflect the age cycle and general demographics among pupils in a given year; the Special Schools in question probably did not have any nine-year-olds in 2005–6 but may have had some in subsequent years. With a view to being as inclusive as possible (even at the risk of including invalid population elements in the population) these 47 schools were included in the population frame for sampling purposes.

Table 2.2: Distribution of nine-year-old children according to three main types of national schools in Ireland (2005–6 academic year)

	**A. Number of Children**			**B. Percentage of Children**		
**No. of 9 year olds**	**Mainstream**	**Special**	**Private**	**Mainstream**	**Special**	**Private**
Zero						
1-10	8,007	352	56	14.8%	84.9%	9.7%
11-15	5,938	35	55	11.0%	8.4%	9.5%
16-30	15,310	28	231	28.3%	6.7%	39.9%
31-40	7,478	0	32	13.8%	0.0%	5.5%
41-50	4,132	0	49	7.6%	0.0%	8.5%
51+	13,246	0	156	24.5%	0.0%	26.9%
Total no. nine-year-olds	54,111	415	579	100%	100%	100%

Accordingly, notwithstanding the problem of false positives, in the interests of ensuring that the population frame was as comprehensive as possible, we included in the sampling frame all Mainstream Schools, except for the 80 which recorded having no nine-year-olds in 2005–6 and which have also been classified as being ‘Infants Only’. In addition, all Special and Private schools were included.

Table 2.2 details the distribution of nine-year-old children in Ireland according to school type and size category. The total number of nine-year-old enrolments as per the Department of Education and Science database stood at 55,105 in 2005–6. Of these, 54,111 were in the mainstream national sector, 415 were in Private Schools, and 579 were enrolled in Special Schools. One can see from Section B of the table that 67.9 % of nine-year-old children were in mainstream national schools containing 40 or fewer target children. Levels of concentration in Private Schools are fairly similar with 64.6 % of nine-year-olds in schools with 40 or fewer target children. Finally, one can see that all nine-year-old children within the Special Schools sector were in schools which have fewer than 30 children in the relevant age bracket.

Table 2.3: Total number of all mainstream, special and private national schools which recorded having nine-year-olds in 2005–6 classified by the number of nine-year-olds and region

**Mainstream**	**Border**	**Dublin**	**Mid-East**	**Midland**	**Mid-West**	**South-East**	**South-West**	**West**	**Don’t Know**	**Total**
Size Cat. (nine-year-olds)										
Zero	0.0%	0.0%	0.0%	0.0%	0.0%	0.0%	0.0%	0.0%	0.0%	0.0%
1-10	54.3%	15.9%	32.3%	48.4%	52.8%	42.0%	44.9%	65.6%	0.0%	45.9%
11-15	15.2%	9.7%	15.9%	18.3%	15.2%	18.3%	17.0%	14.2%	0.0%	15.3%
16-30	22.5%	27.5%	28.3%	23.3%	22.0%	25.3%	23.5%	14.4%	0.0%	22.8%
31-40	4.3%	16.7%	7.6%	6.7%	3.4%	6.5%	8.1%	3.6%	0.0%	7.0%
41-50	1.0%	7.5%	5.1%	2.5%	2.2%	3.8%	2.3%	1.2%	0.0%	3.0%
51+	2.7%	22.6%	10.9%	0.8%	4.3%	4.3%	4.2%	1.0%	0.0%	6.0%
Total with nine-year-olds	100.0%	100.0%	100.0%	100.0%	100.0%	100.0%	100.0%	100.0%	0.0%	100.0%
(n) of schools	**488**	**371**	**276**	**240**	**322**	**372**	**481**	**494**	**0**	**3,044**

**Special Schools**										
Size Cat. (nine-year-olds)										
Zero	0.0%	0.0%	0.0%	0.0%	0.0%	0.0%	0.0%	0.0%	0.0%	0.0%
1-10	100.0%	91.5%	100.0%	100.0%	100.0%	90.9%	100.0%	100.0%	0.0%	95.6%
11-15	0.0%	5.7%	0.0%	0.0%	0.0%	9.1%	0.0%	0.0%	0.0%	3.3%
16-30	0.0%	2.9%	0.0%	0.0%	0.0%	0.0%	0.0%	0.0%	0.0%	1.1%
31-40	0.0%	0.0%	0.0%	0.0%	0.0%	0.0%	0.0%	0.0%	0.0%	0.0%
41-50	0.0%	0.0%	0.0%	0.0%	0.0%	0.0%	0.0%	0.0%	0.0%	0.0%
51+	0.0%	0.0%	0.0%	0.0%	0.0%	0.0%	0.0%	0.0%	0.0%	0.0%
Total with nine-year-olds	100.0%	100.0%	100.0%	100.0%	100.0%	100.0%	100.0%	100.0%	0.0%	100.0%
(n) of schools	**8**	**35**	**9**	**4**	**6**	**11**	**11**	**7**	**0**	**91**
**Private**										
Size Cat. (nine-year-olds)										
Zero	0.0%	0.0%	0.0%	0.0%	0.0%	0.0%	0.0%	0.0%	0.0%	0.0%
1-10	0.0%	25.0%	60.0%	0.0%	0.0%	100.0%	0.0%	0.0%	50.0%	35.7%
11-15	0.0%	0.0%	20.0%	0.0%	0.0%	0.0%	66.7%	0.0%	16.7%	14.3%
16-30	0.0%	50.0%	20.0%	0.0%	100.0%	0.0%	33.3%	0.0%	16.7%	35.7%
31-40	0.0%	8.3%	0.0%	0.0%	0.0%	0.0%	0.0%	0.0%	0.0%	3.6%
41-50	0.0%	8.3%	0.0%	0.0%	0.0%	0.0%	0.0%	0.0%	0.0%	3.6%
51+	0.0%	8.3%	0.0%	0.0%	0.0%	0.0%	0.0%	0.0%	16.7%	7.1%
Total with nine-year-olds	0.0%	100.0%	100.0%	0.0%	100.0%	100.0%	100.0%	0.0%	100.0%	100.0%
(n) of schools	**0**	**12**	**5**	**0**	**1**	**1**	**3**	**0**	**6**	**28**

Table 2.3 outlines the regional distribution of the three categories of school collapsed by size category. It is evident that the size structure of schools in all three sectors varies substantially by region. The Dublin region is particularly noteworthy in terms of the proportion of mainstream national schools which fall into the two largest size categories. Thus, while 46.9% of schools in Dublin have 30 or more nine-year-old pupils, this compares with only 8% in the Border region, approximately 10% in the Midwest and Midlands region, and around 14% in the other regions.

2.1.2 LEVEL OF COVERAGE

If one is to use the database of schools as a frame for the first stage of sample selection, the population of children in the education system should be comprehensive with few exclusions. The Departmental estimates for the year 2005–6 indicate that of approximately 55,100 nine-year-olds were in the school system. This compares with the Census of Population figure of 56,500 for usually resident nine-year-olds from the enumeration conducted on 23rd April 2006. This suggests a high degree of concurrence between the Census and Departmental estimates. A few factors may explain the differences in the figures from the two sources. First, the Departmental figures contain some *estimates* in respect of schools which had not returned actual figures for the database. Secondly, there were differences in reference period. The Departmental figures relate to the school year 2005–6 and the schools returned the data early in that academic year. The Census night relates to the end of the following April. Thirdly, the Departmental figures did not include children who are being home-educated, although this would account for only a small absolute number of nine-year-olds. According to figures provided by the National Educational and Welfare Board (NEWB), there were approximately 1500–2000 children between the ages of 4 and 16 being educated at home in 2006. On this basis the actual number of

children in any year group is likely to be small, averaging about 150 children[5]. On balance, taking the above factors into account, the Study Team considers that the divergence between the Census and Departmental figures is very small indeed and that the Census data clearly validate the comprehensiveness of the population frame based on the Departmental lists of schools.

The number of children in care is frequently raised as an important issue in terms of the comprehensiveness of a population frame based on the National School system. Table 2.4 outlines details on the number of nine-year-old children in different types of care in the country. From this one can see that the total number of relevant children involved was relatively small – 288 in total in the year 2004. One of the most significant features of the table is that almost all of the children in care are in some form of foster setting. Only 11 nine-year-old children are in residential care. All children in a foster environment receive primary education in the system and so are (at least in principle) included in the proposed population frame.

Table 2.4: Distribution of nine-year-old children in care by type of care in 2004

Type of Care	***Number***	***%***
General Foster Care	198	68.8
Special Foster Care	1	0.3
Relative Foster Care	74	25.7
Pre-adoptive Placement	1	0.3
General Residential Care	10	3.5
High Support Residential Care	1	0.3
At home under Care Order	3	1.0
TOTAL	288	100.0

After: Table 2.2, Preliminary analysis of childcare interim dataset, 2004, Dept. of Health and Children

2.2 SAMPLE DESIGN

In deciding on the sample design an appropriate balance had to be struck between the number of schools recruited into the sample and the number of children selected within each of the sampled schools. Given the relatively small size of most of the national schools in the country, it was considered feasible to attempt to recruit all children in small schools (less than 40 nine-year-olds) included in the sample. From an operational perspective it becomes more problematic to do so in larger schools, where the amount of administrative and other work involved is likely to act as a major disincentive for principals and teachers in participating in the survey. To address this issue an upper threshold of participating children in any given school was introduced.

During the initial stages of sample design the Study Team experimented with different combinations of numbers of sampled schools and ranges of threshold values (at the pupil level) to determine how these alternative combinations of sampling points (schools) and pupils would affect the likely composition of the resultant sample. On the basis of these simulations it was decided that an upper threshold of 40 should be imposed on the number of pupils to be recruited from any given school. Since national schools will draw their pupil base from a relatively localised area, one can reasonably expect that within-school variability of pupil characteristics will be less than between-school variability.

[5] Personal communication from NEWB

Accordingly, increasing the number of primary sampling points (schools) is clearly highly desirable from a statistical perspective. By introducing the within-school pupil threshold, this can be achieved whilst simultaneously minimising the respondent burden for the principal and staff involved.

In selecting the sample the population of schools was split into two subgroups depending on whether or not they were identified from Departmental records as having more or fewer than 40 nine-year-old children. Among the schools containing fewer than 40 nine-year-olds, the sample was selected on a systemic stratified basis. The population frame was pre-stratified according to the following criteria

- County
- Disadvantaged status
- Denominational status
- Categorical size (i.e. the total number of nine-year-old pupils)
- Gender mix.

Among the schools with more than 40 nine-year-olds, we used a design based on probability proportionate to size (pps) for the school – where the size of school was measured in terms of the number of nine-year-olds. Adopting this approach for the larger schools meant that the larger the school the higher was its selection probability. This slightly higher selection probability at the stage of selecting the school was counterbalanced at the second stage of selecting pupils within the school. At this latter stage, the selection probability of the individual pupil was negatively related to number of pupils in the school. Accordingly, a pupil who came from a larger school had a marginally higher probability that his/her school would be included at the first stage of sample selection. At the second stage of selection, however, this was counterbalanced by each child having a slightly lower selection probability, as not all children in the school were included in the final sample (given the maximum threshold of 40 children).

As noted above, when selected for the sample we attempted to recruit all children within the age range from schools with 40 or fewer nine-year-olds. In situations in which the school listed more than 40, the principals were provided with a set of random numbers to select which pupils to include/exclude from the sample[6]. Given the size structure of the school population, it was estimated that principals would have to sample from students above the 40 pupil threshold only in 10% of schools.

2.3 REFUSAL CONVERSION

Great efforts were made by the Study Team to ensure the highest possible response rates. In participating schools, at least two rounds of information and consent forms were originally issued to all families. Furthermore, in May and June 2007 schools with low pupil response rates were identified and requested to issue a third set of information and consent forms to non-participating families. Although this was reasonably well received by the majority of the principals in question, many did point out that they had previously done everything possible to encourage families to participate in the project, such as contacting the non-responding parents either in person, by phone or by text to encourage them to participate in the survey, and that further shots of information leaflets and consent forms would not be productive.

With a view to increasing the response rate at both the school and pupil levels the Study Team carried out a two-phase refusal conversion exercise in September 2007 when the schools re-opened for the new

---

[6] Because in the majority of schools the number of children *excluded* was generally much smaller than the number included the Principal was actually instructed in how to use the random number table to *exclude* a usually small number of children from the study. In a very small number of schools where there were more than 150 eligible children, a random number table was used to select 40 children to be *included* in the sample.

academic year. This exercise had two objectives. First, we attempted to increase the within-school response rate of participating schools. This aspect of conversion targeted the schools with the highest levels of non-response. The interviewers returned to the schools in question and requested the principal to issue a final set of information and consent forms to the non-respondent children who were originally recorded in the School Record Sheet as falling within age range but who were not recruited into the sample. New, slightly shorter, versions of the information sheets were prepared for this exercise as it was felt that potential respondents were perhaps overwhelmed by the volume of information in the first information packs and that this might have adversely impacted on response rates. The experience of the September exercise conversion mirrored the experience of earlier in the year. Principals, although willing to help, felt that they had already maximised response rates. The conversion exercise yielded an additional 130 children, increasing the response rate in some schools but with little impact on the overall rate.

The second component of refusal conversion was to increase the school-level response rate by approaching some of the non-respondent schools and asking them to reconsider. We specifically targeted somewhat larger, Dublin-based schools which have been designated as disadvantaged as the effective sample of children was slightly under-represented among these schools. Again, success was limited in this exercise. We approached a total of 67 schools which had initially refused to participate in the study, but only 6 of these agreed to re-consider their position. This low level of refusal conversion in September/October 2007 reflects the substantial efforts already made to convert the refusals in May/June.

2.4 REWEIGHTING THE SURVEY DATA

All survey data from the project will be reweighted and grossed in advance of analysis and deposit in the national data archive. The fine details of reweighting have yet to be worked out as the full dataset becomes available. It will, however, be based on some relatively standard form of iterative procedure for adjusting the effective sample to column marginals. Where appropriate, the sample design will obviously be incorporated into the reweighting procedure. At this stage we would envisage that this would involve a two-phased weighting scheme to reflect the school characteristics of the pupil and so adjust for the design and selection effects outlined above. It is anticipated that the main external controls for the marginals were extracted from the Census of Population, 2006 along with estimates from the European Union Survey of Income and Living Conditions (EU-SILC), of particular relevance in adjusting for income and related characteristics of the household. The weighting system used is known as GROSS. This is a minimum information algorithm that fits population marginals in a regression framework and adjusts the sample estimates to ensure that they produce estimates which match human population parameters. It has been used extensively by the ESRI over many years to provide weighted estimates of almost all of its surveys (see, for example, Gomulka 1992, 1994).

Chapter 3

INPUT TO INSTUMENTS

CHAPTER 3: INPUT TO INSTRUMENTS

In this chapter we describe the various groups of experts and others who have had input to the development of the instruments and procedures used in ***Growing Up in Ireland***. We also describe the processes by which that input has been received. First of all, we consider the Scientific and Policy Advisory Committee. This group is followed by descriptions of the Delphi Process, the Children's Advisory Forum, the Expert Panels, and Stakeholder Groups. Lastly we consider the other longitudinal studies from which we have drawn some of the items in the various instruments. These processes, particularly the Scientific and Policy Advisory Committee, were important in meeting study objective #9 regarding the provision of evidence for the creation of effective and responsive policies and services.

3.1 SCIENTIFIC AND POLICY ADVISORY COMMITTEE

The study received input from the Scientific and Policy Advisory Committee (SPAC). This is a non-executive group that provided scientific and policy advice on the content and best practice of the design, implementation and roll-out of the study. Its 10 members were selected from a very broad range of backgrounds in areas related to children and large-scale longitudinal national surveys – both substantive and technical. Members were selected on the basis of their expertise in

- policy and policy formulation as it impacts on children and families in Ireland
- the substantive area of childhood, and research into issues relating to childhood and children
- technical and statistical areas of particular relevance to the operation of a complex longitudinal study comparable to ***Growing Up in Ireland.***

The Scientific and Policy Advisory Committee meets approximately three to four times per year and has the following Terms of Reference:

- Review and advise on protocols and procedures in the context of best international practice for large-scale longitudinal projects similar to ***Growing Up in Ireland.***
- Advise on relevant policy and research issues as they relate to children and their families in the changing Ireland of the 21st century.
- Review and advise on draft questionnaires and other instruments to ensure that these reflect the policy and substantive issues identified as being of importance to the study.
- Review summary results and their interpretation (in policy and substantive terms) as they emerge from the study.

The Committee is chaired by the Co-directors of the Study Team with other members of the Study Team Management Group in attendance. The composition of the SPAC reflects its primary objective of providing independent policy, methodological and substantive input to the development and implementation of the project. Committee members have been drawn from a number of areas with widely varying specialisms as follows

- Policy specialist, Department of Social and Family Affairs
- Policy specialist, Department of Education and Science
- Policy specialist, Department of Health and Children
- Senior policy analyst, National Economic and Social Forum
- Senior methodologist, quantitative surveys
- Senior legal expert, child and family issues and academic
- Senior epidemiologist and public health specialist and academic
- Senior health promotion researcher and academic
- Senior social policy analyst and academic
- Senior educational researcher and academic
- Senior researcher, child and family support and academic.

The SPAC gave extensive feedback on the instrumentation for the older cohort, the experience of the pilot for that cohort, and the qualitative work to be carried out with the children and their families. In addition, it has provided advice on the development of the instrumentation and pre-pilot work for the infants – the subject of a parallel report on that cohort.

3.2 DELPHI PROCESS

A two-round Delphi process was implemented as part of instrument development. This involved administering a questionnaire to elicit the views of an expert panel on the salient topics for inclusion in the instruments to be used with the nine-year-old children and their families. Various aspects of the Delphi process were agreed with the Project Team and Steering Group in the following important areas

- Panel selection
- Nature, format and content of First Round instrument
- Field procedures.

Each is briefly discussed below.

3.2.1 PANEL SELECTION

A purposeful selection procedure was adopted to generate the panel of experts for the Delphi process. A panel of 71 experts was generated for the first round of the Delphi process, the experts in most cases being drawn from among policy-makers (including all 15 government departments), statutory agencies, service providers, non-government organisations and the voluntary sector, with a focus on the wellbeing of young children. In the first instance the Study Team developed an initial listing of experts. This initial listing was cleared, with amendments, by the Project Team and Steering Group for the project. All suggestions from the various other components of project governance were included in the final list of panel members used for the project.

3.2.2 FORWARD INFORMATION TO THE PANEL OF EXPERTS

In the first round of the process a short note outlining (i) the background and objectives of ***Growing Up in Ireland*** and (ii) the Delphi consultation process was sent to panel members with their questionnaire. All participants were also invited to an information seminar on the process held by the Study Team. This seminar included an overview presentation of the project along with details of how the Delphi consultation process worked, followed by a Question and Answer session.

3.2.3 THE INSTRUMENT USED IN ROUND ONE

Given the general experience of Delphi processes identified from the literature we decided to adopt a semi-structured approach in the first round of the process. A total of six broad domains or potential research topics were initially identified as follows

1. Child's health and development
2. Child's functioning
3. Parenting/Family Context
4. Child's education
5. Community/Neighbourhood
6. Sociodemographic characteristics

Each broad domain contained a number of individual research areas or topics. A total of 41 specified research topics was included across all six domains. The pre-coded topics were complemented by providing substantial space on the questionnaire for respondents to add, at their own discretion, any topic, areas or comments which they felt had been overlooked or not sufficiently covered by the pre-coded areas provided.

Each respondent was asked to assign a score of 0 to 5 in respect of each of the items included on the questionnaire in terms of their perceived importance*.* Respondents were told that a score of '0' indicated that the items in question was 'of no importance' while a score of '5' indicated that the item was 'extremely important'.

3.2.4 FIELD PROCEDURES

The first mail shot in the first round of the Delphi questionnaire was issued by post. Included with the initial mail shot was an introductory letter, an information sheet on ***Growing Up in Ireland*** and the Delphi process in general, a copy of the questionnaire, and a pre-paid envelope for the return of the completed survey.

This was followed by intensive phone follow-up to ensure that the questionnaire had been received and that it had gone to the most appropriate person in the relevant organisation. Phone follow-up continued on an on-going basis throughout fieldwork. In the course of the phone phase, additional copies of the questionnaire were issued by a combination of post and email. A dedicated email account (NLSCI@esri.ie) was set up for this latter purpose.

Successfully completed questionnaires were secured form 89 % of respondents to the first round of the Delphi. All of these were included in the second round, which had a response rate of 94 %.

3.3 CHILDREN'S ADVISORY FORUM

Growing Up in Ireland is a study of children, for children and with children. To this end the Children's Advisory Forum (CAF) was set up to make sure the voices of children were heard within the study[7]. The role of the CAF is to advise the Study Team on how best to run the study and to make sure that the views and opinions of children are appropriately incorporated into the design and development of the study. It forms a key part of study objective #7 to obtain children's views and opinions on their lives.

The CAF is made up of 84 children who sit on 12 committees in schools throughout Ireland. Seven boys and girls sit on each committee. The schools in which the committees sit are spread across several regions including Limerick, Cork, Westmeath, Dublin, and Wicklow. These regions were chosen in order to represent all types of schools in terms of urban/rural location, religious denomination, designated disadvantaged status or otherwise, socio-economic composition of the school, and co-educational/single-gender composition.

3.3.1 THE STRUCTURE OF THE CAF MEETINGS

Initial meetings with the CAF School committees were held on site in each school during November 2006–February 2007. The initial school meetings had the following aims

- To introduce the children to the two CAF facilitators and the external evaluator
- To introduce the children to the background and objectives of the ***Growing Up in Ireland*** study

[7] The CAF was a requirement of the governance structure set out in the Request for Tender for the Project.

- To provide the children with more information about the role of CAF
- To hear the children's perspectives on some preliminary issues relating to the design of the study.

The school meetings were structured as follows

- Ice Breaker Exercises
- Introducing ***Growing Up in Ireland*** and the CAF
- Input into the Study Design
- Evaluating the Meeting.

In addition to the school meetings, four regional meetings were held with the CAF children between March and April 2007 with a view to eliciting the children's views on a number of potential qualitative methods for use in the main qualitative study with nine-year-olds. The regional meetings offered the children a chance to meet with children from the other schools in their location who were participating in the CAF process. At each regional meeting 21 children participated. At least four facilitators and the external evaluator attended the regional meetings. Each meeting was held in a venue away from school to give the children the opportunity to participate together in a neutral location. The regional meetings lasted three hours.

The regional meetings were structured as follows

- Group Ice Breaker

- Workshop Groups

- The Time Capsule
    - At the start of the first workshop all the children were introduced to the concept of creating a time capsule. It was explained that the facilitators wanted to find out lots of things about nine-year-olds and to explore how things change for children by the time they are thirteen. The children were given a poster tube and asked to create a time capsule in which the materials they used during the workshop could be stored and given to the facilitators. The children were given time at the start and the end of the workshops to decorate their time capsules.

- The Passport
    - Each child completed a blank passport card. On the passport card the children filled in their name, age, hometown and answered questions about their favourite band, movie, food, colour, hobby, the one thing they like the most and the one thing they hate the most. The children were asked to swap their completed passport cards with the person beside them and introduce themselves to each other.

- The Workshop Themes
    - To provide the facilitators with the opportunity to pilot a number of different qualitative techniques, the three smaller groups each worked on different themes using a variety of materials for their two workshops. Each group was assigned two themes to explore from the following domains: Child's Health and Physical Development, Education, Family and Parenting, Community and Neighbourhood, Child's Functioning, and Relationships and Growing Up.

- Ending the Regional Meetings

3.3.2 INPUT OF THE CAF

The CAF children have had a number of inputs to the study for far, including

- providing their opinions and recommendations on a logo for ***Growing Up in Ireland***
- assessing the quality of an information leaflet for children
- suggesting topics for the questionnaire which was administered to the nine-year-old participants of the Study
- participation in pre-testing and piloting of the Child Questionnaire.

The children's suggestions were generally incorporated into the relevant questionnaires used in the quantitative study. These suggestions included questions on hobbies, what nine-year-olds do in their free time, pupil-teacher relationships, bullying, the extent of friendship networks, food and drinks, brothers and sisters, chores undertaken around the home, and what they like about where they live.

Feedback on a draft of the child questionnaire, which was incorporated into the questionnaire included

- adding a 'sometimes' option to some questions, as they felt that a mere 'yes/no' answer was too restrictive
- providing an explanation of what is meant by a 'chat room' and 'instant messaging'
- including an option of 'emptying the dishwasher' along with 'washing dishes' in question 20 about chores.

In addition to the inclusion of substantive issues, the children's views on changes to question wording and response categories were included. In pre-testing, several children felt that questions on smoking and drinking posed them difficulties and that it would be unlikely that they would be answered correctly. (These questions were dropped before main fieldwork began.)

In relation to the general administration of the child's questionnaire, a proportion of the CAF children completed the audio version (see section 5.4.2). Although several of the children felt the pace of the audio was too slow and that it might be frustrating for some children to complete the questionnaire in this way, the option of audio assistance was retained for children who needed help with reading.

3.4 EXPERT PANELS

Four expert panels assembled by the Study Team contributed to the design and instrumentation used in ***Growing Up in Ireland***. The four panels are headed by members of the Study Management Team in the position of Theme Director(s) as follows:

- Health and Health Policy – Prof. Tom O'Dowd (TCD) and Prof. Richard Layte (ESRI)
- Child Development and Education – Prof. Sheila Greene (TCD)
- Social Context and Social Institutions – Prof. Chris Whelan (ESRI) and Prof. Brendan Whelan (ESRI)
- Methodology and Design – Prof. James Williams (ESRI).

The panels of experts are made up of specialists drawn from a very wide range of backgrounds, including the following:

• Public health and primary care	• Social economics
• Psychology – all aspects	• Epidemiology – health behaviours
• Poverty, social exclusion and health economics	• Smoking and alcohol consumption
• Sampling and survey methodology	• Diet and nutrition
• Tax, benefits, poverty and deprivation	• Oral health
• Youth research and policy	• Paediatrics
• Social development and social policy	• Child Psychiatry
• Family, gender and the labour market	• Exercise and health
• Social policy	• Health psychology
• Early childhood development	• Diet and nutrition
• Educational development	• Genetic psychiatry
• Family and gender	• Family, gender and demography
• Social mobility and the labour market	• Criminology and social psychology
• Social mobility and educational disadvantage	• Health statistics
• Ethics in research – with particular relevance to research with children.	

The expert panels were consulted throughout the development phase of the project and on an on-going basis. They were initially requested to suggest domains, topics and questions which were of particular relevance to their specific area of expertise. They were asked to provide references to other studies that had previously covered these areas or could justify the inclusion of innovative question topics. Draft versions of the questionnaires were sent for comment to the panel members. They were also consulted after the pilot, and based on the experience and results of the pilot were asked to provide suggestions on streamlining and prioritising questions for deletion from excessively long draft instruments used in the pilot phase.

3.5 STAKEHOLDER GROUPS

Members of the study team also met with stakeholder groups, and feedback from these meetings was incorporated into the development of the instrumentation and the design of the project in general. The Study Team worked particularly closely with the funding bodies and associated Government departments, which include

- The Office of the Minister for Children
- The Central Statistics Office
- The Department of Social and Family Affairs

Representatives from these Government departments and agencies sit on the Project Team which oversees ***Growing Up in Ireland***. An extremely important component of that group comprises two international advisors who were previously instrumental in the design, development and implementation of the Longitudinal Study of Australian Children (LSAC) and the National Longitudinal Study of Children and Youth (NLSCY) in Canada. The interdepartmental Project Team is chaired by the Office of the Minister for Children. The Co-directors of the Study meet on a monthly basis with the full Project Team.

The overall Steering Group for the project involves a further high-level inter-departmental group of senior officials from the Department of Health and Children, the Office of the Minister for Children, the

Departments of Social and Family Affairs, the Department of Education and Science and the Central Statistics Office. The Co-directors of the project meet with the Steering Group approximately each quarter, principally for sign-off on particularly significant milestones, such as instrument development, pilot and/or Dress Rehearsal stages. The Steering Group is chaired by the Director of the Office of the Minister for Children.

The input from the funding stakeholders and Project Team was in addition to consultations with other stakeholder groups who advised us on their own particular areas of interest and expertise. For example, Treoir were particularly interested in recording information on non-resident fathers, and these views supported our plan to administer a postal, self-complete non-resident parent questionnaire. The Irish National Teachers Organisation and the Irish Primary Principals' Network gave us advice on the school-based component of the study and actively supported the project with the teachers and principals involved. These groups included

- The Irish National Teachers Organisation
- The National Parents Council Primary
- One Parent
- The Children's Rights Alliance
- The Ombudsman for Children
- Parental Equality
- The Irish Primary Principals' Network
- Treoir.

3.6 OTHER LONGITUDINAL STUDIES

In developing the instrumentation the Study Team tried to synchronise with contemporary longitudinal child cohort studies, both in order to enable later comparison and to draw on the benefits of including items previously used in other studies. Where items for ***Growing Up in Ireland*** were based on questions used in other studies we have indicated our source in the text[8]. Below, we provide some background information on the main studies from which we have drawn items.

3.6.1 MILLENNIUM COHORT STUDY (MCS)

The Millennium Cohort Study is a longitudinal study of 18,819 children born in the UK over 12 months from 1 September 2000 in England and Wales and 1 December 2000 in Scotland and Northern Ireland. The first sweep took place when the children were nine months old, the second sweep at age three years, and the third sweep is underway as the children start school. The study looks at a broad range of issues such as poverty and wealth, and quality of family life. The MCS is implemented by a consortium headed by the Centre for Longitudinal Studies at the University of London. The main MCS website can be accessed at http://www.cls.ioe.ac.uk.

3.6.2 GROWING UP IN AUSTRALIA (LSAC)

Growing Up in Australia (Longitudinal Study of Australian Children) is a longitudinal study of children with two nationally representative cohorts, each of 5000 children: one aged under 12 months in 2003/4 and the other aged 4 years in the same year. The younger cohort will be followed until aged 6–7 years and the older cohort until they reach 10–11 years. The study has a wide multi-disciplinary brief with a heavy

[8] We would point out that many items and questions have been adapted from numerous child cohort studies. Throughout Chapters 6 to 9, we generally cite the main source of each item. The Study Team is aware that in many instances the cohort study quoted may not have been the original developer of the item. Contact was established with all of the main sources to discuss our use of items from the relevant questionnaires.

emphasis on policy relevance. Personal visits to households are interspersed with mailings of self-complete questionnaires (0.5 waves). The study is currently on Wave 2.5. Growing Up in Australia is co-ordinated by the Australian Institute of Family Studies in Melbourne. Their website can be found at http://www.aifs.gov.au/growingup/home.html

3.6.3 NATIONAL LONGITUDINAL SURVEY OF CHILDREN AND YOUTH (NLSCY)

The National Longitudinal Survey of Children and Youth (NLSCY) is a longitudinal study of Canadian children from birth to early adulthood. The study's brief is to collect information on factors affecting a child's social, emotional and behavioural development, and to monitor the impact of these factors over time. Data are collected every two years starting in 1994 with a national sample of children aged between 0 –11 years. There have been 2 further cohorts added, those who were under 12 months at Cycle 4 in 2000 and those who were under 12 months at Cycle 5 in 2002. At the last cycle in 2004/5 (Cycle 6) there were 26,000 children in the sample. The study is run by Statistics Canada. The NLSCY website can be found at http://www.statcan.ca/cgibin/imdb/p2SV.pl?Function=getSurvey&SDDS=4450&lang=en&db=IMDB&dbg=f&adm=8&dis=2.

3.6.4 EARLY CHILDHOOD LONGITUDINAL STUDY (ECLS)

The Early Childhood Longitudinal Study is an American study of the early years of child development with two cohorts. The birth cohort has a nationally representative sample of 14,000 born in 2001 who are being followed until they enter kindergarten. It is 'designed to provide decision-makers, researchers, child care providers, teachers, and parents with detailed information about children's early life experiences'. Data were collected from these children at 9 months, 2 years (2003), pre-school (2005), and 2006 when most children were eligible for kindergarten entry. Further data from the minority who are only entering kindergarten in 2007 will be collected at that stage.

The kindergarten cohort focuses on the kindergarten class of 1998/9 and follows these 21,000 children until they reach middle-school (8th grade) in 2007. The study focuses on early school experiences and interaction with individual, family, school and community influences. Data are collected at intervals of kindergarten (age 5), 1st, 3rd, 5th and 8th grades. The Early Childhood Longitudinal Study is run by the National Centre of Education Statistics, Institute of Education Sciences at the US Department of Education. The website for the ECLS is at http://nces.ed.gov/ecls/index.asp.

3.6.5 AVON LONGITUDINAL STUDY OF PARENTS AND CHILDREN (ALSPAC)

The Avon Longitudinal Study of Parents and Children has, primarily, a health and development related focus. The stated main goal is 'to understand the ways in which the physical and social environment interact, over time, with the genetic inheritance to affect the child's health, behaviour and development'. Data collection from questionnaires is supplemented with biological samples (hair, etc.), DNA samples, access to medical records, and direct assessments. From an initial sample of 14,541 pregnancies there were 13,971 infants at age 12 months. All pregnant mothers were resident in the Avon area of South West England with an expected delivery date between 1 April 1991 and 31 December 1992. Self-complete questionnaires were sent to mothers every few months in the early years, and additional questionnaires were sent to the child himself/herself, starting in the 65th month. In later childhood, questionnaires were sent quarterly and children were asked to present for assessment every year. The study plans to monitor the children into adulthood. ALSPAC is run by a dedicated team based at the University of Bristol. The ALSPAC website is at http://www.alspac.bris.ac.uk/welcome/index.shtml.

Chapter 4

ETHICAL CONSIDERATIONS

CHAPTER 4: ETHICAL CONSIDERATIONS

The importance of ethics in research is receiving wider acknowledgement than ever before. In a study of children and families it becomes an even more prominent priority. The Study Team identified a number of ethical issues and put procedures in place to deal with them. The Study Team also had to be mindful of its obligations under the relevant acts in Irish legislation. The current chapter summarises the pertinent parts of legislation and describes the way in which our ethical guidelines were put into practice. We finish with a short description of the role of the Research Ethics Committee. The primary concern at all times was the protection of child participants in the Study. Procedures relating to child protection were informed by the *Children First Guidelines* (Department of Health and Children, 1999). All interviewers, as well as other staff working on ***Growing Up in Ireland***, were security vetted by An Garda Siochana (the Irish Police Service). A full module on ethics was included in the interviewers' training course.

4.1 RELEVANT ACTS

Three acts are of particular relevance for this Study: the Data Protection Acts 1988, 2003, and the Statistics Act, 1993.

4.1.1 DATA PROTECTION ACT 1988, 2003

Data protection concerns the integrity, protection, storage and use of information collected from and about individuals. Under the Data Protection Acts, 1988, 2003, the Study Team undertook the following obligations:

1. *Fair obtaining and processing:* Respondents must be fully aware of the identity of the persons who are collecting the information, the use to which it will be put, and the purpose or bodies to whom it will be disclosed. See Section 4.2.1 on informed consent for further discussion.
2. *Specifying the purpose:* Information may not be kept about people unless it is held for a specific, lawful and clearly stated purpose.
3. *Further processing of personal information:* If personal information is obtained for a particular purpose the data may nor be used for any other purpose or divulged to a third party, except in ways that are compatible with the specified purpose.
4. *Security of personal data:* Stringent procedures are implemented in both the ESRI and TCD to ensure that computer and security of data is preserved at all times.
5. *Accurate and up-to-date*: Personal information which is kept must be accurate and up-to-date.
6. *Adequate, relevant and not excessive*: The data shall be adequate, relevant and not excessive in relation to the purpose or purposes for which they were collected or are processed.
7. *Protection of personal data*: The data shall not be kept for longer than is necessary for that purpose or purposes. See Section 4.2.4 on confidentiality for further discussion.
8. *Right of access to personal data*: Any individual about whom information is kept has a right to see a copy of the data, a description of the purposes for which the data are being held, and a description of those to whom the data may be disclosed. See Section 4.2.4 on confidentiality for further information.

4.1.2 STATISTICS ACT (1993)

Growing Up in Ireland is being conducted within the framework of the Statistics Act, 1993. This is the legislation underpinning the work of the Central Statistics Office (CSO). The study has been brought under the scope of the act in accordance with Section 11, whereby the Office is permitted to make arrangements with other public authorities for the conduct of statistical inquiries. While the act facilitates access to certain data sources for the purposes of the Study, the most important implication is that it provides a strong legal basis for the protection of *all* information collected against unlawful disclosure.

Under the Act all information collected must be treated as strictly confidential and used for statistical purposes only. All persons working on the Study are appointed Officers of Statistics. As such they are legally obliged not to disclose, except for the purposes of the Study, any matter which comes to their knowledge relating to any person, family, household or undertaking in the course of their statistical work.

Results of the study will be published in aggregate form and all necessary steps will be taken to ensure that details relating to an identifiable person are not inadvertently divulged.

4.2 PRACTICAL APPLICATION OF ETHICAL CONSIDERATIONS

4.2.1 INFORMED CONSENT

Detailed information sheets were prepared for all potential participants in the study, including parents, children, teachers, Principals, non-resident parents, and regular carers. These sheets described the type of information that would be gathered, what would be involved for participants, the longitudinal nature of the study, and details about the researchers and funding bodies. All participants were informed of the voluntary nature of the study and of their right to refuse to answer any questions that they did not wish to answer. Signed consent was obtained from a parent/guardian and the Study Child concerned before any data were collected about that child.

4.2.2 REPORTING CONCERNS

Interviewers were instructed to report to the Study Team all events or observations which caused them concern during the course of their work on an Incident Report Form, especially with regard to the protection of children or other vulnerable persons. All reported incidents were then considered by, and acted upon as necessary, by the Project Directors. Interviewers were provided with an out-of-hours emergency phone number at which they could contact a Project Director if they had serious concerns.

4.2.3 INTERVIEWERS BEING ALONE WITH CHILDREN

It was stressed upon interviewers during training that they must not be left alone with any child while conducting the fieldwork, even for a few minutes. This guideline was also clearly stated in the information sheet provided to parents in advance of their consent into the Study. Interviewers were encouraged to suspend an interview and return at a later date or time if a parent/guardian or other adult found it necessary to leave an interviewer with a child – even for a short period. Interviewers were also prohibited from having any physical contact with the Study Child, for example during the height and weight measurements.

4.2.4 CONFIDENTIALITY

All interviewers and other staff working on the project were appointed as Officers of Statistics by the Central Statistics Office. This imposed a legal obligation on them to preserve and protect the confidentiality of all information they received in the course of the Study. Under the Statistics Act (1993), (see Section 4.1.2 above), a breach of confidentiality is a criminal offence. At interviewer training it was emphasised that not all breaches of confidentiality are malicious in nature. Many can occur through thoughtless or careless comments made to third parties after the interview has been completed. It was emphasised that comments to third parties included comments to other household members. For example, a reference to a respondent about issues raised by a previously interviewed spouse or partner would constitute a breach of confidentiality.

It was also emphasised in training that carelessness in handling survey materials (especially those containing respondent contact information) could constitute an effective non-malicious breach of confidentiality. Interviewers were instructed, for example, to protect the confidentiality of their computer password at all times and not to carry Work Sheets containing respondent’s addresses except when

necessary. Only Work Sheets and related materials relevant to the specific households currently being interviewed should be taken into the field.

Access to the non-anonymised datasets is severely restricted and great care will be taken to remove any identifying information from the anonymised dataset. No Government department or agency will have access to identifiable information, and the Central Statistics Office will be the only body other than the ESRI to hold a copy of the non-anonymised dataset. In addition, the following steps have been taken to ensure the confidentiality of information given as part of ***Growing Up in Ireland***.

- Use of numerical codes on all electronic and paper questionnaires
- Use of passwords and user names on laptops
- ‘Strip-down’ of laptops to prevent inadvertent connection to a wireless network
- Encryption of all electronic information transferred by interviewers to a dedicated secure server in the ESRI
- Separate mailings of paper questionnaires and Work Assignment Sheets – the latter containing contact information.
- Operating under the Statistics Act (1993) to ensure that the information obtained can only be used for purposes of statistical compilation and analysis.
- Restricting the access of respondents to only the information that they themselves have provided – no individual is allowed to see another person’s answers, even if that person has recorded details in respect of the individual in question, for example neither Study Children nor their parents have access to what a teacher has recorded about a pupil.

4.2.5 AVOIDANCE OF EMBARRASSMENT/DISTRESS

Pro-actively avoiding the possibility of causing embarrassment or distress is intrinsically linked to the maintenance of confidentiality both within and outside the home. Within the home, sensitive questions concerning the marital/parental relationship, for example, were self-completed by the respondents on a paper questionnaire rather than being asked aloud by an interviewer (unless requested). Interviewers were prohibited from getting involved in any family disputes or giving advice, regardless of any other qualifications or experience they had in such matters. Interviewers were, however, provided with a list of helpline numbers for a variety of agencies, which they could pass on to respondents if asked.

4.3 ETHICS COMMITTEE

The quantitative phase of the child cohort was carried out under ethical approval granted by the Research Ethics Committee of the Health Research Board. The pilot and main studies underwent separate review procedures. Reports on the pilot study in the schools and in the home were submitted promptly to the Committee. The Ethics Committee was very active in its consideration of all of the materials and procedures used in ***Growing Up in Ireland***. For example, they made substantial contributions to the content and layout of information sheets, as well as recommendations in relation to the instruments themselves. The Study Team met with the Ethics Committee to discuss the project on several occasions, and all recommendations were acted upon before a final version of all materials and procedures was agreed and implemented.

Chapter 5

OVERVIEW OF INSTRUMENTS AND PROCEEDURES

CHAPTER 5: OVERVIEW OF INSTRUMENTS AND PROCEDURES

This chapter provides an overview of general procedures, instruments and respondents. Sections 5.1 and 5.2 outline the school phases of recruitment and fieldwork respectively. Fieldwork in the home is summarised in Section 5.3. Special procedures are described in Section 5.4. Minimal details on instruments are provided in this chapter as its purpose is to provide a broad overview of the various levels of instrumentation and their administration before going into the detail of their substantive content in subsequent chapters; cross-references are provided to more detailed descriptions elsewhere in this document, where relevant.

5.1 SCHOOL AND PUPIL RECRUITMENT

Work on sample recruitment in the schools began in March 2007, after ethical approval was secured in February. Following the school's selection into the sample, the Study Team despatched an information pack containing detailed information about the Study for the principal and teachers. A sample pack is contained in Appendix A.

The interviewer assigned to the school then arranged an appointment with the principal to discuss the school's potential involvement in the Study. On agreeing to participate the principal and relevant staff completed a School Record Sheet. This recorded the names and other basic details of all children in the school whose dates of birth fell within the specified age range[9]. In selecting children for inclusion in the study, school principals were instructed to include children who were born between 1 November 1997 and 31 October 1998.

Information packs, including consent forms, were despatched to selected children and their parents/guardians through the school. A copy of the content of these packs can be found in Appendix B. Parents/guardians were asked to return completed consent forms (one each for a parent/guardian and child) to the school. The completed forms were then collected and returned to the Study Team by the interviewer. These consent forms contained the address and contact details which were then used to make direct contact with parents and arrange interviews. Refusal conversions among families and children involved the schools in issuing further information packs to non-respondent families, details of which can be found in Chapter 2.

A series of national and local radio advertisements were run when the recruitment process was underway in the schools. These consisted of a week-long run of advertisements on national radio stations (RTE, and Today FM) and also on a selection of local radio stations throughout the country. Each set, on RTE, Today FM, and also on the group of local networks, was run for three one-week periods.

5.2 FIELDWORK AND INSTRUMENTS IN THE SCHOOLS

Once recruited into the sample the school was asked to complete the following instruments and documentation:

- The Principal Questionnaire, which recorded general information on school characteristics including size, challenges, ethos, etc., and some personal details about the principal.

---

[9] See Chapter 2 for further information on recruitment of the sample.

- The Teacher-on-self Questionnaire, which recorded general information about the class(room) characteristics such as size, curriculum, preferred teaching methods, etc., and some personal details about the teacher.
- The Teacher-on-child Questionnaire, which recorded specific information about the study child such as temperament, academic performance, and school preparedness.

In addition, the interviewer administered the following academic tests and instruments to the children in group self-completion sessions carried out in the schools:

- The Druncondra English reading and Maths test – curriculum-based, standardised tests used to indicate level of ability in reading and maths.
- The Piers Harris 2 self-concept questionnaire - a self-complete booklet measuring positive/negative self-concept.

5.3 HOUSEHOLD-BASED FIELDWORK AND PARTICIPATION OF THE FAMILY

On completion of the school-based phase of the project, the participating households were assigned to an interviewer for the household-based component of the study. A letter of introduction was sent a few days in advance of the interviewer's first contact with the family. In most cases a telephone number had been provided by the family on the consent form returned to the school. Where available, this number was used by the interviewer to make the first personal contact with the family in order to arrange an appointment for interview. If no phone number was available, the interviewer made a personal visit to the house.

The informants in the home were, in all cases, the primary caregiver (usually the mother) and the Study Child. Where relevant, the resident spouse/partner of the primary caregiver was also interviewed in the home; that person was often, but not necessarily, the father of the Study Child. The main interviews were administered on a CAPI (Computer Assisted Personal Interview) basis for all household respondents. More sensitive questions were extracted and were administered to respondents on a self-complete paper supplement.

The interviewer training emphasised the need to establish a good rapport with the Study Child as a priority for the interview. Interviewers were instructed to try and gain the confidence of each child and develop a rapport with him/her before commencing the formal interview process. All interviewers were made aware of the power imbalance that may exist between the child and the interviewer, how many children might feel a sense of obedience towards adults, and how they can be inclined to provide answers that they think are expected or will please the interviewer. Training was provided to encourage interviewers to think about the interview process from the child's perspective. The interviewers were trained to allow the parent to make the initial introduction between the child and the interviewer. All interviewers were encouraged to put the child at ease by asking about their favourite activities, welcoming them into the study and explaining that their answers were very important to allow the government to plan things for young people. The types of questions were briefly explained and the child was told that they did not have to answer any questions that they did not wish to. They were also reassured that the child's questionnaire was not a test and that they should try to answer the question in their own words. If the child needed help to understand a question the interviewer was instructed to explain the question but not to prompt an answer. If for any reason the child became upset or ill the interviewer was instructed to ask the parent/guardian to intervene and comfort the child. It was only when the interviewer was satisfied that the child was happy that the interview was resumed. If the child seemed tired at any time the interviewer asked them if they would like a short break or if they were happy to continue, and if the child signalled that they wished to terminate the interview it was ended immediately.

The following is a complete list of all instruments associated with the household

1* **Mother/Lone Father questionnaire (main and supplementary sections)**
2 **Father/Partner questionnaire (main and supplementary sections)**
3* **Child questionnaire (main and supplementary sections)**
4* **One-day time-use diary**
5 **Questionnaire modules for Twins and Triplets**
6* **Follow-up information**
7* **Height and weight of main participants**
8* **GPS co-ordinates**
9* **The Work Assignment Sheet**
10* **Interviewer Observations**
11^ **Non-resident parent questionnaire**
12.1^ **Carer (home-based) questionnaire**
12.2^ **Carer (centre-based) questionnaire.**

*These core items were completed for all households.

^Items 11, 12.1 and 12.2 were issued by Study Team on a postal basis and self-completed by the non-resident parent/regular carer, where relevant.

Detailed descriptions of all instruments are provided in the following chapters

- Chapter 6 – School instruments
- Chapter 7 – Parent/guardian questionnaires
- Chapter 8 – Child questionnaires
- Chapter 9 – Other instruments
- Chapter 10 – Scales and standard measures.

5.4 SPECIAL PROCEDURES

Growing Up in Ireland aims to be as inclusive as possible. Putting special procedures in place to achieve a high level of inclusion was important for achieving the study objectives relating to describing the lives of Irish children (#1), mapping variation in children's lives (#5) and providing an evidence base for the creation of policies and services (#9).

5.4.1 DISABILITY

Adults with vision problems were interviewed using CAPI (Computer Assisted Personal Interview) for the main interview and PAPI (Pen and Paper Interview) for the sensitive supplement, subject to their agreement. Children with vision problems (for whom self-completion of the sensitive questionnaire was problematic) completed a main CAPI interview in the usual way and completed the sensitive supplement on a PAPI basis in the presence of two interviewers, rather than in the presence of a parent and interviewer (subject to the agreement of the parent). See Sections 4.2.1 and 5.2 on obtaining informed consent.

Deaf children and adults self-completed all questionnaires on a pen-and-paper basis.

Every effort was made to maximise the participation in the Study of children with learning disabilities or other special needs to the best of their individual abilities, in consultation with parents and teachers. The ultimate decision as to their inclusion and the extent of that inclusion rested with the parent(s)/guardian(s) of the Study Child in question.

5.4.2 LITERACY

Adults with literacy problems were given the option to have the self-complete questionnaires administered by the interviewer on a PAPI basis. In some cases the interviewer had been aware of literacy problems via the school or the parents/guardians themselves. There were two questions on literacy in the main interview for both the Mother/Lone Father and Father/Partner, which also served as an indicator to the interviewer that they might need to administer the sensitive questionnaire on a PAPI basis to that respondent. Children with literacy problems were given an audio soundtrack on CD to assist them in completing the sensitive supplements: children listened to the questions being read out on the CD and indicated their answers on the paper questionnaire. All children attempted the Drumcondra tests unless the parent/guardian or teacher advised the interviewer that an attempt would cause distress to the Study Child.

5.4.3 OTHER LANGUAGES

Information sheets and questionnaires were translated in advance into Irish, Romanian, Russian, French and Polish, which were then self-completed by respondents on pen-and-paper during a home visit. Questionnaires were translated into other languages on a case-by-case basis. A translator was provided to households on request. Information sheets were also available in Braille, audio and large font formats.

Irish language versions of the Teacher and Principal questionnaires were provided to schools on request.

5.4.4 TWINS AND TRIPLETS

In households where there were nine-year-old twins or triplets, the adult respondents completed one main Mother/Lone Father and Father/Partner interview on CAPI and answered child-related questions in relation to one of the twins. They then completed a ‘Twin Module’ for the second and subsequent children on a PAPI (Pen and Paper-Interview) basis. These modules repeated only the child-related questions, this time to be answered in relation to the second twin or triplet. The modules also contained some specific questions on parenting twins, such as identical/fraternal status, age at which differences were noticed, and so on. The questionnaires are contained in Appendix M.

For child respondents, each twin/triplet completed a full child interview with sensitive supplements. The first child respondent completed the main interview on CAPI and self-completed the sensitive supplements, as in the case of single children. For subsequent children, the interviewer recorded answers for the main interview on a paper questionnaire rather than on the laptop. The interviewer attempted to administer twin modules and child interviews in all households with nine-year-old twins/triplets, even when the existence of a twin was not known prior to the visit to the household.

5.5 CAPI PROCEDURE

Interviewers administered the main questionnaires using a laptop (*Model: IBM Thinkpad, Lenovo X60*). Each question appeared on the computer screen for the interviewer to read out with space for an answer option to be recorded. Answers were recorded, in the main, by entering the number associated with the selected answer option using the keyboard. Answers can, however, also be recorded using an integral mouse or by entering free text where appropriate. The questionnaire was programmed using BLAISE software. This program facilitated the routing of questions (skipping non-applicable questions, for example), and the inclusion of hard and soft cross-variable and range checks to alert interviewers to improbable or impossible answers or conflicts between answers.

Respondents were shown an extensive range of prompt cards with the available answer options. These were particularly important for longer lists of options or items in a scale. Interviews could be suspended and returned to at later time according to the requirements of the respondent, for example if an

unexpected visitor called to the house during an interview. Completed interviews were outputted as ASCII files from BLAISE, and were encrypted and uploaded to a dedicated server in the ESRI by the interviewers across the phone line. They were then decrypted and rebuilt to produce a SPSS file for preliminary analysis of the data.

5.6 GIFTS TO RESPONDENTS

Small gifts were offered to child, teacher and principal respondents as tokens of appreciation for their participation in ***Growing Up in Ireland***. The gifts were only offered post-participation so that they would not act as an incentive or inducement to take part. Teachers and principals were sent €25 worth of book tokens each. Study Children were given pencil case sets. Parents/guardians were asked for permission to offer the gifts before they were presented to children. Interviewers were also provided with a supply of colouring pencil sets to give to any siblings who might be upset at being left out.

Chapter 6

INSTUMENTS USED IN THE SCHOOL

CHAPTER 6: INSTRUMENTS USED IN THE SCHOOL

Following the general overview of school and pupil recruitment, procedures and instruments used in the schools and special procedures in relation to disability and literacy presented in Chapter 5, this chapter will describe the questionnaires and instruments used with the school staff and pupils participating in the study. The school, in addition to the home, is an important part of the child's microsystem (see section 1.3.1), and gathering data from and about the school contributes principally to study objective #8 to provide a bank of data on the whole child. Where no question sources are specified, these questions have been developed by ***Growing Up in Ireland***, typically in conjunction with the expert panels (section 3.4).

Once recruited into the sample, the school was asked to complete the following instruments and documentation

- School record sheet
- Principal questionnaire
- Teacher-on-Self questionnaire
- Teacher-on-Child questionnaire.

In addition, the interviewer administered the following academic tests and instruments to the children

- Drumcondra Reading Vocabulary and Maths tests
- Piers-Harris Children's Self-Concept Scale, 2nd edition (Piers-Harris 2).

6.1 SCHOOL RECORD SHEET

Once a sampled school agreed to participate in the Study, the principal filled out the details of all pupils falling within the specified date-of-birth range on the School Record Sheet (see copy in Appendix C). This sheet recorded details on the number of eligible children, names of teachers in whose classes these children were being taught, whether or not the child had a learning or other difficulty, and whether or not English was the first language of the child. This sheet was used to record the population of eligible children and also to assign to them appropriate ID numbers to be used throughout the project. In addition, it assigned ID numbers to the relevant teachers to allow linkage of the teacher questionnaires to the pupil details. The random numbers table, used to ensure that a maximum random sample of 40 children was selected from a given school, was included on the last page of the School Record sheet (see Section 2.2 for further information on the selection process).

6.2 PRINCIPAL'S QUESTIONNAIRE

The principal was asked to complete one questionnaire which recorded details in respect of the school. In addition to capturing basic demographic information such as the number of pupils and the number of staff, the questionnaire measured a variety of important school-level variables such as the adequacy of facilities and resources, the prevailing value system and ethos of the school, as well as various aspects of school climate. This information will be of value in performing between-school comparisons of educational outcomes. The Principal's Questionnaire is contained in Appendix D.

**Q1 - Q3**	**Personal Information** – These items captured basic descriptive information in respect of the principal such as age, gender, the number of years he/she has been principal at their current school, and the number of years as principal in other primary schools.
**Q6 - Q8**	**Staffing Resources** – The questions on staffing resources included the number of teaching and administrative staff employed in the school on a full-time and a part-time

	basis, and whether the school had additional capacities in terms of learning supports such as resource teachers, special needs assistants, etc.
**Q9 - Q12**	**Classroom Provision** – Information was collected on the number of permanent and temporary classrooms in the school, the number of classes across all year groups, and the number of children the school was designed to accommodate. There is continuing dispute in the literature concerning the impact of educational inputs (staffing levels, class size, etc.) to educational outcomes at the school level. Although Hanushek (1997; 2003) has argued that there is little evidence to support the idea that resources are positively related to educational outcomes, there is good evidence summarised in Greenwald, Hedges and Laine (1996) and Krueger (2003) that school resources such as per-pupil expenditure, teacher-pupil ratio and class size are systematically related to student achievement.
**Q14**	**Adequacy of School facilities and resources** – This question was adapted from the Early Childhood Longitudinal Study and is designed to assess the adequacy of the school's facilities and resources across 17 areas (e.g. number of teachers, number of classrooms) with responses indicated on a four-point likert scale ranging from *poor* through *excellent*. Seven of the original Early Childhood Longitudinal Study items were retained and supplemented with ten additional items provided by the education panel of experts. There is evidence summarised in Schneider (2002) that student achievement is correlated with better school facilities, such as newer school buildings and modern libraries and laboratories.
**Q15**	**Free school meal provision –** This question relates to whether the school provides a breakfast club or free meals at lunchtime. This is frequently used as a proxy for disadvantage.
**Q16 - Q18**	**Computer resources in the school** – Details collected included the total number of computers available in the school, the number of these that can be used by the pupils, and whether there is a dedicated computer room in the school. The issue of whether the provision of computers in the school has any positive effects on school-level educational attainment, independent of other socio-economic covariates is under-researched and warrants further investigation.
**Q19**	**Ethos of the school –** This question measures the importance of different activities (e.g. Irish language and culture, sports) to the prevailing ethos of the school and is designed to explore variation across different types of school and by gender.
**Q20**	**School-community relationships –** This is a question on whether the school buildings and facilities are open to the local community outside of school hours.
**Q21**	**Classroom Composition –** This question records information in respect of the number of children who are foreign nationals or are from families in the Travelling Community, as well as the number of children with sensory, language and learning difficulties. Studies have consistently shown that the background of fellow students has a strong impact on educational outcomes, and that both ability-mix and social-mix have an effect on pupil progress and achievement (Rutter & Maughan, 2002)
**Q22 - Q23**	**School attendance levels** – The school returns these figures to the Department of Education and Science on an annual basis and they relate to the average daily attendance for the school year, and the proportion of pupils who missed 20 days or more. Research points to the strong link between attendance and educational outcome (Lamdin, 1996), and studies have found that schools with higher rates of daily attendance tend to out-perform schools with lower attendance on achievement tests (Roby, 2004).
**Q24**	**School Catchment Area** – This question asked about the proportion of students who live within a 20-minute walk from the school. The extent to which students are drawn from a local catchment area gives an indication of the utility of using the District Electoral Division for small area population analyses.
**Q25 - Q26**	**Emotional/behavioural problems and school supports** – Question 25 is related to the level of interpersonal supports within the school for children with

	emotional/behavioural problems and the extent to which they adopt a whole-school approach. Question 26 was previously used in the ESRI Survey of Disability in Second-Level (schools) and recorded details on the proportion of students who have such literacy, numeracy or behavioural problems as to adversely impact on their educational development. A high incidence of children with these types of problems may be indicative of a challenging teaching and learning environment.
**Q27**	**School Community Liaison Co-ordinator** – This item recorded whether the school has a school community liaison co-ordinator.
**Q28 - Q33**	**Admission and streaming criteria** – This set of questions was designed to assess the degree to which the school is selective in its admission criteria. The increasing pressure on school places within large urban areas has prompted interest in the extent to which there is 'selection' within the primary school sector, and whether this is differentially related to educational outcomes at the school level.
**Q34 - Q35**	**Engagement with parents** – Information was collected on whether the school holds a formal parent-teacher meeting at least once a year and the proportion of parents in attendance. Parental involvement is often considered a measure of school climate (Ma, 1999) and high parental involvement is considered a correlate of school effectiveness (Marzano, 2002).
**Q36 - Q37**	**Curricular and extra-curricular activities** – These questions are related to the importance attached by the school to a range of curricular and extra-curricular activities. Research with adolescents has shown that pupil involvement in extra-curricular activities, such as sport and music, can help foster a positive school climate and may be related to positive educational outcomes (Fullarton, 2002; Alva, Elmore, Nord, & Zill, 2004).
**Q38 Q40**	**Disciplinary policy within the school –** Question 38 was adapted from the British Cohort Study (1970) and asked about the frequency with which various forms of discipline are applied within the school. Question 39 asked whether the school had a formal policy on discipline and question 40 asked to what extent teachers, parents and pupils were involved in developing the policy. Previous research in Ireland with secondary-level students has shown that a 'strict but fair' and consistent disciplinary policy is associated with better school results and higher levels of pupil retention. More effective schools have been found to involve parents early in the disciplinary process and to adopt a whole-school approach to it (Smyth, 1999). Moreover, research suggests that when rules, sanctions and procedures are developed with input from students and teachers, this contributes to a sense of ownership and belongingness that is conducive to learning (Cotton, 2000).
**Q41 - Q43**	**Bullying within the school** – These items asked the principal to what extent bullying was a problem in the school and whether the school had an explicit anti-bullying policy, or a written policy on bullying. School bullying has become a topic of public concern and considerable research in various countries around the world in the last two decades (Smith & Ananiadou, 2003), and research indicates that schools which employ a formal anti-bullying strategy tend to have lower rates of bullying (Fekkes, Piipers, & Verloove-Vanhorick, 2006).
**Q44 - Q48**	**Principal's perception of general school climate** – Question 44 was adapted from the teacher schedule for 'Do Schools Differ' (Smyth, 1999) and relates to the principal's general perception of teachers within the school. Questions 45 – 47 asked how the scale of day-to-day problems and general environment in the school compares with other primary schools in the country, and question 48 is related to the degree of satisfaction that the principal derives from his/her job. Previous research in Ireland indicates that less academically effective schools are characterised by less positive relations between management and staff and less supportive relations among colleagues (Smyth, 2004).

6.3 TEACHER-ON-SELF QUESTIONNAIRE

The purpose was to record background details on the teacher himself/herself, such as age, gender, qualifications, teaching methods adopted in class, etc. In addition, the teacher-on-self questionnaire recorded information at classroom level on topics such as curriculum, teacher's homework policy, teaching methods and class composition. This questionnaire was filled out on a self-completion basis (see Appendix E).

**Q1 - Q7**	**Background characteristics of the teacher** – Personal information collected about the teacher included gender, age, qualifications and continuing professional development.
**Q8 - Q10**	**Basic characteristics of the class** – These questions recorded information on the Study Child's class, including size, year groups and number of children with Special Needs. This information relates to the type of teaching challenges that the teacher may have to deal with in the classroom and the level of support he/she received from Special Needs Assistants.
**Q11**	**Subjects undertaken** – Details were recorded on the range subjects undertaken by the pupils in the Study Child's class and the time spent on each subject in a week. This information is related to the breadth of the curriculum.
**Q12 - Q17**	**Teaching methods** – The teacher was asked to record details on his/her teaching methods, including aspects of interactive and passive teaching techniques such as play or taking down notes. Planning of teaching and the extent to which it is tailored to the needs of the students may be significant in pupil achievement. These questions have been adapted from instruments used in 'The Transition Year Programme', 'Moving Up', and 'Coeducation and Gender Equality' studies (Smyth, Byrne and Hannan, 2004; Smyth, McCoy and Darmody, 2004; Hannan, Smyth, McCullagh, O'Leary & McMahon, 1996).
**Q13 - Q15**	**Computers in the classroom** – The teacher was asked about access and use of computers in the Study Child's classroom. Complementary questions are asked in the Mother/Lone Father Questionnaire regarding computer access at home. An Irish study reported that while 80% of Irish respondents had internet access only 12% of Irish children used it daily (NCTE, 2003). It is not known whether computer and internet provision at home has a differential effect on performance in school. Computer literacy may also have a positive impact on education or career outcomes. Usage can be monitored longitudinally in Wave 2, and inform proposed Government policy on provision of laptops to secondary school students.
**Q16**	**Time assigned to homework** – In Ireland this has been found to be highly predictive of student performance at second level. A recent study of 15-year-old students' experiences indicates that more time on homework and study resulted in positive academic outcomes (Smyth, Dunne, Darmody & McCoy, 2007). Engagement with homework emerged as a more crucial factor in subsequent achievement than engagement with schoolwork.
**Q18**	**Teacher control and input to decision-making in the classroom** – This question asked about perceived control over various aspects of teaching including selection of subjects and year group, teaching methods and discipline. Previous research in Ireland has found that greater teacher involvement in decision-making in the classroom leads to benefits in terms of satisfaction and student achievement (Smyth, McCoy & Darmody, 2004; Smyth, Byrne & Hannan, 2004)**.**
**Q19, Q22 - Q23**	**Teacher's perception of school** – These items recorded details on the teacher's perception of how happy the school environment was for pupils and for the teacher himself/herself. The rationale is that school climate will be linked to performance. These questions were adapted from Smyth (1999) and Early Childhood Longitudinal Study (2000). School organisation and ethos can make a difference to student attendance. Students appear to respond to positive interaction with teachers and to

	teacher expectations in terms of their attendance levels (McCoy, Darmody, Smyth & Dunne, 2007).
**Q20 - Q21**	**Parental attendance at parent-teacher or school meetings** – These items recorded details on the level of involvement of parents in the school and their interest in the child's education. Little research has been done on parent involvement at primary school level. These questions complemented Question 11 on the Teacher-on-Pupil questionnaire (see Section 6.4). Pupil and parental involvement in schools in the UK have been associated with effectiveness, particularly for schools in disadvantaged areas (National Commission on Education, 1996) and with higher performance and lower absenteeism (Mortimer et al. 1988).

6.4 TEACHER-ON-PUPIL QUESTIONNAIRE

In addition to the Teacher-on-Self Questionnaire, each teacher was asked to self-complete a separate questionnaire for each Study Child in his/her class. The Teacher-on-Pupil Questionnaire focused on the individual Study Child including the Study Child's behaviour, and teacher's assessment of school preparedness and ability (see Appendix F). In some cases, the principal was also a teacher of the Study Child.

**Q1 - Q6**	**Characteristics of Study Child** – Basic information was recorded on the Study Child including gender, date of birth, absenteeism, and how long the teacher had known the child.
**Q7**	**Attending school in an appropriate state** - These questions recorded the frequency of the Study Child arriving at school in an appropriate state for school, including being adequately dressed for weather conditions, being hungry, lacking cleanliness, etc. Attending in an inappropriate state may be associated with misbehaviour, low achievement and performance, and may also be an indicator of neglect. This question has been adapted from the Early Childhood Longitudinal Study. A recent study of four Dublin primary schools which were designated with disadvantaged status, found that almost one in five pupils (18%) said that they were often 'too hungry to do their work in school' (Downes, Maunsell & Ivers, 2006)
**Q8**	**Completion of homework** – This item recorded the frequency with which Study Child had homework completed/not completed. Parent's involvement in homework appears to influence student success by supporting positive a perception of personal competence, positive attitudes to homework and self-regulatory skills (Hoover-Dempsey, Battiato, Walker, et al., 2001).
**Q9**	**Strengths and Difficulties Questionnaire (SDQ)** – This was the standard SDQ completed by the teacher to measure the Study Child's behaviours in the classroom. The SDQ was chosen because of its single short form, which is suitable for both parents and teachers. It was also used in the Mother/Lone Father Questionnaire (see H2 in Chapter 7).
**Q10**	**Performance relative to other students in the class** – The teacher was asked to rate the Study Child's academic performance on a range of subjects/abilities relative to other children in his/her class. This information complements the estimates of performance in reading and writing provided by both the Mother/Lone Father and the Study Child himself/herself.
**Q11**	**Attendance of Study Child's parents/guardians at parent-teacher meetings** – This information is related to parental engagement with the Study Child's education. A recurrent concern for teachers and Home School Community Liaison co-ordinators is the non-involvement of marginalised parents in their children's education (Mulkerrins, 2007). O'Neill (1992) found that working-class parents may be reluctant to get involved in their child's education because they do not feel confident in dealing with teachers.

Q12 - Q14	Conditions that limit activities – These items recorded whether or not the Study Child had any disability (physical, sensory or learning) problem or other characteristic that limited his/her participation in school, and the associated supports which he/she received from the school. This is a measure of the supportiveness or otherwise of the structures within the school for those who need them. The National Council for Special Education was set up in 2003 to facilitate the inclusion of the child with special education needs in the school system. Guidelines on the Individual Education Plan Process have been published by the NCSE (2006), and research is ongoing on the preparation of teachers with regard to inclusive pedagogy. The quality of support teachers obtained in the primary school classroom is, however, unknown.

6.5 DIRECT ASSESSMENTS OF CHILDREN

6.5.1 PIERS-HARRIS CHILDREN'S SELF-CONCEPT SCALE (2ND EDITION)

The Piers-Harris Children's Self-Concept Scale (2nd Ed.) is a 60-item scale that measures self-concept. It was self-completed by the majority of children in booklet form after the academic assessment in the school, although, for administrative reasons, some children completed it at home. Detailed information on this scale is provided in Chapter 10, Section 10.2.1.

6.5.2 DRUMCONDRA TESTS IN READING VOCABULARY AND MATHS

Children completed two academic assessments in group settings within the school. These were the Vocabulary part of the Drumcondra Primary Reading Test – Revised, and Part 1 of the Drumcondra Primary Maths Test – Revised. The children completed Level 2, 3 or 4 for each test depending on which class level they were in. More extensive details on both tests are given in Chapter 10, Section 10.3.

Chapter 7

INSTRUMENTS COMPLETED BY MOTHER/LONE FATHER AND FATHER/PARTNER

CHAPTER 7: INSTRUMENTS COMPLETED BY MOTHER/LONE FATHER AND FATHER/PARTNER

The home component of the study involved personally administered interviews with the parent(s) of the Study Child as well as interviews with the child him/herself. Further information was sought at this stage on postal contact details for a non-resident parent and/or a carer, if appropriate. The questionnaires used specifically with the Mother or Lone Father of the Study Child are discussed in detail. The questionnaire used with the resident Father/Partner is also described in brief, as most of the questions are previously described in the context of the Mother/Lone Father questionnaire. Where no question sources are specified, these questions have been developed by ***Growing Up in Ireland***, typically in conjunction with the expert panels (section 3.4). The main questionnaire was completed on a CAPI basis for both respondents, with sensitive questions being self-completed on a paper supplement.

7.1 MOTHER/LONE FATHER QUESTIONNAIRE

The Mother/Lone Father Questionnaire consisted of 11 sections, each of which equates broadly to a domain of interest. Each section was further divided into general areas of interest based on groups of questions. These are briefly described below. The questionnaire is provided in Appendix G. The Mother/Lone Father questionnaire is a crucial tool in meeting all of the study objectives, with the exception of those concerned with obtaining children's views and opinions (see section 1.2).

7.1.1 SECTION A – BACKGROUND INFORMATION

This section captured personal or descriptive demographic and related information in relation to the household.

**A1 - A3**	**Relationship of respondent to the Study Child**
**A4 - A5**	**Household Composition –** This table recorded personal details in respect of each person resident in the household. This information is important in terms of ascertaining family composition and structure, since research suggests different outcomes for children raised in different family compositions and structures. For example, children raised in single-parent families or stepfamilies have been shown to have more behavioural problems on average than children raised in intact families with two biological parents (Carlson & Corcoran, 2001; O'Connor & Jenkins, 2000). However, it is also important that these data are used in conjunction with information on both the current and previous marital status of the main caregiver and intra-familial relationships (discussed below) in order to obtain a more accurate view of the family structural variables that influence the child.
**X1a - X1e**	**Multiple births –** This question recorded information on twins or triplets including whether they attended the same school and lived in the same household as the Study Child. It was used as a filter question to direct the interviewer to administer the twin module of the Study if appropriate.

7.1.2 SECTION B - CHILD'S HEALTH

This module focused on the current and past health of the Study Child, including prenatal care and details of the birth.

**B1 - B2**	**Birth-weight and Gestational age –** These questions recorded details on birth-weight and gestational age at birth. Birth-weight has served as a leading indicator of infant health, and low birth-weight (LBW) is associated with increased risk for neonatal morbidity, including neonatal hospitalisation. As a group, LBW children experience more health problems than normal birth-weight children, including increased incidence of chronic conditions and health-related limitations in daily living (Hack, Klein & Taylor, 1995). In addition, there is accumulating evidence from a number of prospective and cross-sectional studies which suggests that low birth-weight is associated with cardiovascular disease in later life (Leon, Lithell, Vagero, et al., 1998) and a range of other adverse behavioural (Kelly, Nazroo, McMunn, et al., 2001) and educational (Breslau, Paneth & Lucia, 2004) outcomes. Question B2 was previously used in Growing Up in Australia.
**B3 - B4b**	**Circumstances of the birth –** These questions recorded details of the birth including the mode of delivery, whether the child was admitted to a neonatal intensive care unit (NICU) or special care nursery after he/she was born, and the length of hospitalisation. Question B4 was taken from Growing Up in Australia and B5 was adapted from the Millennium Cohort Study. Some studies have linked prenatal and perinatal complications and interventions, such as forceps, breech delivery and long labour, with delinquent behaviour in adolescence and adulthood (Kandel & Mednick, 1991; Raine, Brennan, and Mednick, 1994). This association was particularly notable when complications were combined with maternal rejection and separation from caregiver in the first year of life.<br><br>The validity and reliability of retrospective reports obtained almost 10 years after the event is obviously an important empirical issue. Walton, Murray, Gallagher, Cran, Savage & Boreham (2000) examined the validity of parental recall of birth-weight in Northern Ireland by comparing archived medical records with retrospective records obtained 12-15 years after the event and found that 84.8% of parents recalled birth-weight accurately to within 227 grams (1/2 lb), which was less than one-half of a standard deviation. Similarly, McCormick & Brooks-Gunn (1999) examined maternal recall of birth-weight, gestation, and neonatal hospitalisations 8-10 years after initial assessment and reported substantial agreement for categorical comparisons with kappa values ranging from 0.85 to 0.94.
**B5 - B7**	**Prenatal smoking and drinking status –** Questions B5 and B6 explored prenatal smoking patterns including the frequency and quantity of consumption, while B7 was designed to gauge the frequency of prenatal alcohol consumption. Smoking and drinking during the prenatal period is associated with increased risk for a range of adverse developmental outcomes including low birth-weight, diminished respiratory function and a range of psychomotor and neurobehavioural deficits. This assumes added importance in the Irish context given the heavy prenatal usage patterns among Irish women (Shaarani, Walsh, Khawaja, Collins, Byrne, Geary & Harrison, in press).<br><br>Kenkel, Lillard & Mathios (2003) explored the validity of retrospective reports of smoking data over a long period using data from the Canadian National Longitudinal Study of Children and Youth. They found a high level of agreement between retrospective reports of smoking status obtained from the 1992, 1994 and 1998 waves of the study with contemporaneously acquired 1984 smoking data. The results were fairly consistent across all three waves with 93% of non-smokers and 75% of smokers

	in the retrospective reports correctly recalling contemporaneous smoking status. Misclassification errors were rare for non-smokers and heavy smokers, but tended to be higher for those who contemporaneously reported that they were light smokers. The authors surmised that retrospective reports appear to provide '… reasonably valid measures of life-time smoking status'. (p.1312). Similarly, Yawn, Suman and Jacobsen (1998) examined maternal recall of prenatal smoking status 10-15 years after the event and found 99.5% concurrence between retrospective report and medical records (kappa = 0.88).
**B8 - B9**	**Breastfeeding status of the Study Child** – These questions collected information on the incidence and duration of breastfeeding. Although increasing in recent years Irish breastfeeding rates are substantially below international levels, research points to sociodemographic variations in breastfeeding uptake (Department of Health & Children, 2006). Breastfeeding during infancy is believed to confer a number of long-term health benefits including reduced risk of asthma (Zeiger 2003), atopic diseases (Van Odijk, Kull, Bores, et al., 2003) and childhood obesity (Arenz, Ruckerl, Koletzko & von Kries, 2004). It has also been linked with higher cognitive developmental test scores through childhood and into adolescence independent of other covariates (Anderson, Johnstone & Rimley, 1999).<br><br>Li, Scanlon & Serdula (2005) examined the validity and reliability of maternal recall of breastfeeding practice across 11 studies with variable recall periods. They found that retrospective report could yield accurate estimates of breastfeeding initiation and duration, particularly when the recall period was within the first three years. Very few studies have examined the validity of maternal recall over more extended periods, though one study found strong concurrence for initiation (85% correctly identified) when infant clinic records where compared with retrospective report 15 years after the event. However, reporting errors were much higher for breastfeeding duration with only 37% of the sample accurately recalling breastfeeding duration to within one month (Tienboon, Rutishauser & Wahlqvist, 1994).
**B10**	**Child's general health status** – This item was derived from the Living in Ireland survey and serves as an outcome measure of child's general health status with responses indicated on a four-point Likert scale ranging from *very healthy* through *almost always unwell*. There is good evidence, summarised in Blaxter (1989), that such measures are close analogues of clinically assessed health status. Moreover, Haas (2007) has demonstrated the predictive validity of this type of question as a longitudinal indicator of adult health outcomes. Compared with 'excellent', 'very good', or 'good' childhood health, 'poor' childhood health was associated with a three-fold risk of poor adult self-rated health and a two-fold risk of a work-limiting disability or a chronic health condition, without taking account of childhood and current socio-economic status and health-related risk behaviours.
**B11 - B16**	**Chronic physical or mental health problems, illness or disability** – These questions were derived from the European Community Household Panel survey (ECHP – also known as the Living in Ireland survey 1994-2001), and explore the nature, duration and constraints of the illness or disability, as well as the history of any past problems. The presence of childhood illness or disability can have a huge impact on a child's quality of life and that of the family (Eiser, 1997). Chronic illnesses or disabilities affect the lives of children through the limitations they impose on daily life, such as interruptions in regular schooling and restricted opportunities for participation, which can in turn lead to adverse educational and psychosocial outcomes (Newacheck & Halfon, 1998). There is also evidence that these detrimental impacts can project into adulthood. The Living in Ireland survey found that people with illnesses or disabilities fare much worse across a number of outcomes relative to others in their own age group, including lower levels of educational attainment and labour market participation, increased risk of poverty, fewer social supports, and lower rates of social participation

	(Gannon & Nolan, 2005).
**B17 - B18**	**Child's history of accidents** – Question B17 was derived from the Millennium Cohort Study and recorded whether the Study Child had ever had an accident that required hospital treatment or admission. This is important because there is a clear social gradient in terms of risk with children from lower social class backgrounds experiencing a greater number and severity of accidents (Alwash & McCarthy, 1988; Roberts & Power, 1996). B18 asked about the *number* of these accidents which were bone fractures or breaks, and was designed to explore the putative link between calcium deficiencies and increased risk of fractures in childhood (Greer & Krebs, 2006).

7.1.3 SECTION C. CHILD'S USE OF HEALTH SERVICES

This section addressed the Study Child's healthcare utilisation, including healthcare requirements and perceived barriers to access, hospital visits and contact with healthcare professionals.

**C1 - C2**	**Hospital and Accident & Emergency Utilisation** – These items recorded the number of nights spent in hospital over the Study Child's lifetime (excluding at time of birth), as well as the number of A & E visits in the last 12 months.
**C3**	**Frequency of contact with Healthcare professionals** – This question was adapted from the National Longitudinal Survey of Children and Youth and dealt with healthcare services initiated by the mother on behalf of the Study Child, including GP's, medical doctors and other professional specialists (e.g. a psychologist). The importance of private care and the extent of fee-paying in Irish healthcare has led many to argue that the system is not available to all on the basis of need alone, but, rather, that personal circumstances determine the availability, extent of and speed of treatment. This, and related questions, will allow for an examination of the equity of utilisation (for a given level of need) among children across different social groups (see e.g. Layte, 2004).
**C4 - C7**	Healthcare access – These questions were adapted from the National Survey of Children's Health, 2003 and asked whether the Study Child required medical/dental treatment in the preceding 12 months, along with perceived barriers to access. This is important from a public policy and planning perspective, particularly where socioeconomic or geographic factors limit access, since a delay in seeking or receiving healthcare is associated with more complications from, and sequelae to, illness (Starfield & Budetti, 1985).
**C8 - C9**	**Oral healthcare and frequency of dental visits** – These items asked about the regularity of brushing and frequency of attendance at dental clinics. Despite a reduction in the prevalence of dental caries in Ireland, there is still a large number of Irish children who have poor oral healthcare (Friel, Hope, Kelleher, Comer & Sadlier, 2002), and Ireland continues to rank below the average for frequency of brushing according to the 2001/02 Health Behaviour in School-aged Children (HBSC) report (Maes, Maser & Honkala, 2004). The recent all-Ireland survey of children's oral health points to social class differences in oral health with children from deprived backgrounds (indexed using medical card status in the South and social welfare receipt in the North) experiencing more decay than children from more affluent backgrounds (Nunn, 2006).
**C10 - C13**	**Visual and auditory problems** – These questions were adapted from the Millennium Cohort Study and recorded details on current or past problems in the visual or auditory domains, including whether the Study Child had been treated for the problem. Unidentified or untreated visual problems such as hyperopia are associated with impaired reading progress (Williams, Latif, Hannington & Watkins, 2005), while uncorrected hearing problems may interfere with the development of speech and language skills (Bess, Dodd-Murphy & Parker, 1998).

**C15 - C16**	**Speech and communication difficulties** – Question C15 asked about the severity of the speech problem and was adapted from the Parents Evaluation of Developmental Status (Glascoe, 2006). The response categories 'yes' and 'a little' in the original are not mutually exclusive and have been altered slightly to read 'yes, a little' and 'yes, a lot', which will yield a more quantitative estimate of the extent of the problem. Question C16 was adapted from Growing Up in Australia and explored the nature of the speech or communication difficulty. The two response categories dealing with *understanding* speech have been removed and 'stutters, stammers or lisps' has been disaggregated into two response categories for analytical purposes. Speech and communication difficulties are important because speech impairment is associated with adverse educational outcomes (Nathan, Stackhouse, Goulandris & Snowling, 2004) and with difficulties in the area of social and behavioural development (Redmond & Rice, 1998).
**C16 - C18**	**Mobility problems and supports –** These items related to whether the child had a condition that restricted mobility and the type of physical supports they required in order to be able to move around.
**C19**	**Handedness of the Study Child** – Research examining the relationship between handedness and developmental outcomes has shown that left-handed children perform worse across a range of developmental outcomes independent of other covariates (Johnson, Shah & Shields, 2007).

7.1.4 SECTION D – CHILD'S DIET AND EXERCISE

This section recorded information from the parent/guardian about the Study Child's eating and exercise habits.

**D1**	**Study Child's diet** - A 20-item food frequency questionnaire was designed to obtain information relating to the Study Child's dietary intake over a 24-hour period. Twelve of the items were derived from Growing Up in Australia, which were in turn adapted from the Sallis' Amherst Questionnaire (2001), and the eight additional items were added following consultation with the expert health panel set up by the Study Team. It will provide a semi-quantitative measure of children's dietary intake along a number of dimensions (fruit and vegetable, protein, carbohydrates, calcium, and fats and sugars consumption) which are important for assessing the quality of the Study Child's diet. This area is of interest because the incidence of children who are overweight or obese in Ireland has increased rapidly in recent years (Griffin, Younger, & Flynn, 2004; National Task Force on Obesity, 2005) and studies suggest that the mismatch between energy intake and energy expenditure is a major contributory factor (Livingstone, 2001).
**D2**	**Child's milk intake** – This item quantified how much milk the Study Child consumed in the 24 hours preceding the survey. Animal source foods can provide children with a variety of micronutrients that are difficult to obtain in adequate quantities from plant source foods alone. Negative health outcomes associated with inadequate intake of these nutrients include anaemia, poor growth, rickets and impaired cognitive performance (Murphy & Allen, 2003). Furthermore, Kalkwarf, Khoury, and Lanphear (2003) found that women with low milk intake during childhood and adolescence have less bone mass in adulthood and are at greater risk of fracture.
**D3 - D4**	**Eating breakfast (if eaten and what foods)** – These items provided a description of the number of children eating a proper breakfast and the relationship, if any, to school performance and current/future health status. Breakfast, as part of a healthy diet and lifestyle, can impact positively on children's health and wellbeing. For example, Nicklas, Bao, Webber and Berenson (1993) looked at breakfast consumption patterns

	for 467 ten-year-old children. Eating a breakfast made a significant contribution to the child's mean daily nutrient intake, in that a higher percentage of children who did not eat breakfast, compared with those who did, did not meet two-thirds of the Recommended Dietary Allowance for vitamins and minerals. Additionally, Rampersaud, Pereira, Girard, Adams & Metzl (2005) reviewed the results of 47 studies that examined the association between breakfast consumption and nutritional adequacy, body weight and academic performance in children and adolescents. Children who reported consistently eating breakfast had superior nutritional profiles than peers who consistently skipped breakfast. Although children who ate breakfast tended to consume more daily calories, they were less likely to be overweight. The authors also reported that eating breakfast may improve cognitive function related to memory, test grades and school attendance.
**D5 - D7**	**Eating an evening meal, including people with whom Study Child eats and whether he/she sits at a table** – These items provided a description of family eating habits and will be used in the analysis of the implications of a regular evening meal for current and future health status. Videon and Manning (2003) have noted that, among a sample of adolescents, parental presence at the evening meal is positively associated with higher consumption of fruits, vegetables, and dairy foods. The authors suggest that nutrition and health professionals should educate parents about the role of family mealtimes for healthy nutrition.
**D8**	**Special diets including vegetarian, coeliac** – The parent/guardian is asked about any special diets of the Study Child, for example vegetarianism, with an opportunity to record other diets that involved avoidance of food allergens. Kosonen, Rimpelä, Rauma, et al., (2005) studied the consumption of disease-related and non-disease-related diets among Finnish adolescents during 1979–2001. The increase observed over this period for both types of special diets was explained in terms of an increased prevalence of certain diseases (allergies, diabetes) and an increase in vegetarianism due to fads, ideals created by media, and growing ecological awareness.
**D9**	**Parent's perception of Study Child's weight** – This item was derived from the Growing up in Australia study. This perception may be compared with actual weight, the Study Child's own perception of his/her weight, and the parent's perception of his/her own weight. Perception of weight may not be in line with actual weight measurements. For example, Etelson, Brand, Patrick & Shirali (2003) noted that parents surveyed who had overweight children did not differ from other parents in their level of concern about excess weight as a health risk, and tended to underestimate their children's weight.
**D10 - D11**	**Engagement in hard and light exercise** – These two questions were adapted from the Leisure Time Exercise Questionnaire (Godin & Shepard, 1985) and assess the frequency with which the Study Child engages in moderate and strenuous physical activity. This self-report measure has been shown to demonstrate concurrent validity with measures of maximum oxygen intake (VO2 max) and muscular endurance (Godin, Jobin & Bouillon, 1986), and acceptable test-retest reliability (Sallis, Buono, Roby, et al., 1993). It is widely believed that exercise habits established in early childhood can track into adulthood (e.g. Rimal, 2003) and research has demonstrated that physical exercise serves an important function in preventing the development of cardiac disease and other related vascular disorder in later life.
**D12 - D1**	**Travel to school, including distance, transport and time taken (to and from separately)** – Information is collected on travel to and from school which has implications for opportunities to exercise and the relationship of this with current and future health. It is estimated that 73% of Irish primary school children use motorised transport to get to and from school (Fahey, Delaney, & Gannon, 2005).

7.1.5 SECTION E – RESPONDENT'S HEALTH

This section focused on the respondent's own health, in particular, any chronic health conditions.

**E1**	**General health status of respondent** – This item was derived from the Short Form 12 Health Survey which measures generic health concepts and health related quality of life. The item tapped the general health status of the parent on a five-point likert scale ranging from excellent through poor and will serve as a key explanatory variable.
**E2 - E8**	**Chronic physical or mental health problems, illness or disability** – These questions mirror B11 – B16 as discussed above. Chronic health problems, illness or disability of the parent/guardian or of another family member may affect his/her ability to provide care for the Study Child, and may impact on the resources available to the Study Child.
**E9 - E11**	**Healthcare Insurance** – These questions recorded information in respect of the family's medical insurance cover, including the provision of private health insurance. They were adapted from numerous Irish studies, including the Living in Ireland survey 1994-2001, and Watson & Williams (2000). They may provide some valuable explanatory information about the variation in access to, and utilisation of, health services, as well as variation in health status.
**E12 - E1**	**Current pregnancy status** – Female respondents were asked about their current pregnancy status as this information is important for understanding the respondents' current general health status and in qualifying body mass indices.

7.1.6 SECTION F – RESPONDENT'S LIFESTYLE

This section focused on the respondent's health-related behaviours including smoking and drinking habits.

**F1 - F3**	**Respondent's current and past smoking patterns** – These items were derived from the Living in Ireland survey and ask about current (or past) smoking and the extent of smoking. This is of interest because parental modelling is believed to exert a significant influence on children's health-risk behaviours and has been found to predict the onset of adolescent smoking (Flay, Hu, Siddiqui, et al., 1994). Although the validity of self-reported smoking has been challenged on the grounds that smokers are inclined to underestimate the amount that they smoke or deny their smoking status, studies have found that misclassification rates tend to be small in the general population (Studts, Ghate, Gil, et al., 2006). Moreover, Patrick, Cheadle, Thompson, et al.'s (1994) meta-analysis of 51 studies comparing self-reported smoking with direct biochemical measures found high levels of sensitivity (87%) and specificity (89%) for self-report averaged across studies, which reinforces the validity of self-reports, given that alternate techniques (e.g. analysis of urinary cotinine) are not operationally feasible.
**F4**	**Study Child's exposure to environmental tobacco smoke** – This question was designed to measure the Study Child's exposure to environmental tobacco smoke (ETS) in the home. Exposure to ETS is associated with increased risk for lower respiratory tract infections and a series of other related illnesses (Li, Peat, Xuan & Berry, 1999).
**F5 - F6**	**Respondent's current alcohol consumption** – These items were adapted from the Millennium Cohort Study and were designed to measure the frequency of drinking as well as the quantity of consumption of wine, beer and spirits in an 'average' week. This is of interest from a longitudinal perspective because it relates to the influence of

	parents as socialising agents of children's health-related behaviour, particularly since Ireland has the highest per capita consumption of alcohol in the EU (Eurostat, 2003). There is evidence, summarised in Gruenewald and Johnson (2006), that self-reports of drinking quantity and frequency show good concordance with other methods (e.g. timeline follow back procedures), while test-retest reliabilities for wine, beer and spirit consumption ranged from 0.59 to 0.99 one year after initial assessment.
**F7 - F8**	**Respondent's perception of weight and dieting behaviour –** Question F7 was derived from Growing Up in Australia and asked the respondent about his/her perception of his/her own weight on a seven-point scale varying from *very underweight* to *very overweight*, while F8 was designed to obtain a frequency measure of dieting behaviour. This is important from a longitudinal perspective in attempting to understand the precursors of eating disorders in children. For example, research suggests that parents' modelling of concerns about weight and shape may influence children's dietary behaviour (Smolak, Levine & Schermer, 1999).

7.1.7 SECTION G – CHILD'S ACTIVITIES

This section focused on the amount of time spent engaged in leisure-time activities.

**G1 - G4**	**Amount of time spent engaged in various leisure time activities** – These questions asked about the time and nature of the child's engagement in pastimes such as non-school related reading, watching TV/DVDs, using computers, or playing video games. There is evidence to suggest that the type and amount of time spent in various leisure time activities may be differentially related to developmental outcomes. Obesity, for example, has been linked to a number of sedentary leisure pursuits including excessive television viewing and computer game use (Gortmaker, Must, Sobol, et al,, 1996; Robinson, 1999), while leisure time reading is positively related to tests of verbal ability and reading achievement (Anderson, Fielding & Wilson, 1998; Cullinan, 2000). These questions were based on items in the Millennium Cohort Study and the National Survey of Children's Health.
**G5**	**Electronic equipment in the Study Child's bedroom** – This question asked whether the Study Child had a TV, a DVD player, a computer or a games console in the bedroom. This information is related to access to unsupervised material in the media and on the internet. For example, playing violent video games has been linked to increases in aggressive behaviour in children and young adults (Anderson & Bushman, 2001). In what appears to be a response to growing public concern about risks for children associated with the internet, the Government is to establish an Office for Internet Safety and an Internet Safety Advisory Council.
**G6**	**Amount of pocket-money received** – The respondent was asked how much money the Study Child was given to spend on himself/herself in an average week. In adolescents, at least, greater pocket money is associated with increased risk of drinking alcohol (Bellis, et al., reported by BioMed Central, 12 May 2007) and smoking cigarettes (Scragg, Laugesen & Robinson, 2002).

7.1.8 SECTION H – CHILD'S EMOTIONAL HEALTH AND WELLBEING

This section focused on important aspects of the Study Child's emotional health and wellbeing. The section explored (negative) life events *ever* experienced by the Study Child, parental perceptions of different aspects of the Study Child's behaviour, with a focus on both strengths and difficulties, and a measure of the Study Child's temperament.

**H1**	**Life events –** This question provided a list of potentially disturbing and/or traumatic events from moving house to the death of a parent. The respondent also had the opportunity to describe a disturbing event not covered in the list. The nature and number of such events experienced by the Study Child may have implications for current and future wellbeing. For example, experience of parental separation has been associated with increases in behavioural/emotional problems (e.g. Cheng, Dunn, O'Connor, & Golding, 2006). This question was adapted from the National Longitudinal Survey of Children and Youth.
**H2**	**Study Child's behaviour –** This 25-item scale was the Strengths and Difficulties Questionnaire (SDQ), which asked the parent/guardian to say how true each statement is of the Study Child. The scale explored the prevalence of hyperactivity, emotional symptoms, conduct problems, peer relationship problems, and pro-social behaviour. The Study Child's teacher also completed this measure. The SDQ is discussed in more detail in Chapter 10, Section 10.2.2.
**H3**	**Study Child's temperament –** This 20-item scale was the Emotionality, Activity and Sociability questionnaire (EAS), which provides a measurement of temperament for the Study Child. The parent/guardian was asked to rate how characteristic each temperament description/statement was of the Study Child. The EAS is discussed in more detail in Chapter 10, Section 10.4.4.

7.1.9 SECTION J – CHILD'S EDUCATION – PAST AND CURRENT

This section focused on

- childcare
- education (including parental knowledge of and involvement in the child's education)
- parental perceptions of the Study Child's school performance and future expectations
- knowledge of Study Child being bullied
- identification and diagnosis of specific learning difficulty, communication or co-ordination disorder experienced by the Study Child.

J1 - J5	**Childcare** – The questions on current childcare arrangements, derived from the Quarterly National Household Survey (QNHS), recorded information on the nature of childcare arrangements, including type of care and location, how much time per week the Study Child spent in his/her main form of childcare, and how much it cost. In the case of those not cared for by parents or relatives, some findings to date indicate an improvement in school attendance levels for those involved in a range of in-school provisions as well as after school activities. For example, two Irish studies point to the benefits for pupils who participate in after-school clubs (Murphy, 2001; Richie, 1999), while other studies indicate that there is a reduction in the opportunities for young people to get into trouble when they are properly supervised after school hours (e.g., Pettit, Laird, Bates & Dodge, 1997). There is also a retrospective question on whether or not the Study Child experienced regular non-parental care before starting primary school.

**J6, J15 - J16**	**Activities and close friends** – These questions described the child's organised activities outside school and time spent with, and the number of, close friends. The section also recorded whether organised activities had to be paid for. These give some measure of how the Study Child spends his/her personal time, how much interaction they have with peers and to what extent children from lower income families may have reduced participation opportunities. Questions J15 and J16 were based on questions asked in the National Longitudinal Survey of Youth and Children.
**J7, J10, J11, J12 - J14, J17, J25 - J26**	**Parental involvement in the Study Child's education –** This series of questions explored parental involvement in the Study Child's education and asked about<br>• occurrence of formal meetings with the child's teacher over the past year, although this may also be a function of the particular school and its ethos (discussed in detail in Chapter 6 on school instruments)<br>• parental knowledge of and help with homework<br>• perception of how the Study Child is doing in school<br>• access to books in the home<br>• use of public library facilities<br>• and expectations for the child's future.<br><br>This information will enable the links between parental involvement in the Study Child's education and actual school performance to be explored through analysis of the academic achievement tests undertaken by the children. Higher parental involvement in child education has been linked to significant effects on school achievement into adolescence (Feinstein, 1999). Questions J7, J10-11, J13-14 and J17 were based on questions asked in the National Longitudinal Survey of Children and Youth, while J25 was taken from Growing Up in Australia.
**J8 - J9**	**School absenteeism** – These questions collected information on absenteeism including the number of days the Study Child was absent from school in the last school year and the main reasons for this absence. The information on absenteeism provided by the parent/guardian can be compared to that provided by the Study Child's teacher. Absenteeism has been linked to other important factors such as the disadvantaged status of the school (O'Briain, 2006), as well as family, school and community factors that can impact on levels of school disaffection at second level (e.g., Meece, Anderman & Anderman, 2006; Edward & Malcolm, 2002; Dalziel & Henthorne, 2005). Absenteeism is of particular concern since it is linked to lower grades and decreased gains in learning (Kearney, 2003; Lamdin, 1996; Truby, 2001) and is one of the strongest predictors of early school leaving (NEWB, 2005). Conversely, school attendance is correlated with increased academic success. These questions were based on items in the National Longitudinal Survey of Children and Youth.
**J18 - J20**	**Bullying behaviour -** These questions were developed by the Study Team and explore the prevalence, type (physical, verbal, etc.) and nature (ethnicity, disability) of bullying experienced by the Study Child during the past year. The types of bullying were developed in conjunction with the Anti-bullying centre based at Trinity College Dublin. Bullying has been linked to many problems including diminished school performance, poor mental health, delinquent behaviour and future criminality (Parada, et al., 2005). Results from the current study will serve to expand the existing Irish research base on numbers of children experiencing bullying (e.g. O'Moore, et al., 1997) and the nature of that bullying, as well as helping to clarify whether victims of bullying experience poorer outcomes than their peers. The Study Children themselves were also asked questions about their experience of bullying both as victim and perpetrator (see Chapter 8, Section 8.3.1.2, B18 – B21a).

**J21 - J24**	**Learning or other difficulties** – These questions asked about the specific learning difficulties, communication or co-ordination disorders that the Study Child may have, their nature, and if and/or when a professional diagnosed them. This information is important in exploring the impact of these difficulties on the child, in terms of all aspects of their development. For example, not only may educational achievement be compromised but children seen as ‘different’, such as those with learning difficulties, may be at particular risk of bullying (Bee & Boyd, 2007). Information gathered in the current study will give some indication of the prevalence of learning difficulties among Irish school children, and the longitudinal design will help us to assess how such children are affected now and when they make the transition to secondary school. These questions supplement information provided by the teacher on the Study Child’s limitations and whether he/she receives any within-school supports to help overcome these limitations (see Chapter 6.3, questions 12-14).

7.1.10 SECTION K – FAMILY CONTEXT

This section dealt with the family context in which the Study Child lives, including the parent’s relationship with the Study Child, discipline practices, time spent as a family doing activities, work-life balance and religiosity. Information on child conduct was also gathered in this section.

**K1 - K2**	**Parent-child relationship** – These questions concerned the parent/guardian’s emotional relationship with the Study Child. K2 was a standardised measure called the Child-Parent Relationship Scale (Pianta, 1992). It is made up of 30 statements about the child and the parent’s interactions with him/her, the applicability of which the parent rates on a five-point scale. More information about this scale is given in Chapter 10, Section 10.4.6. Positive and supportive interactions between parents and children have been shown to impact positively on social behaviour, school grades and externalising behaviours (O’Connor, Hetherington, & Clingempeel, 1997). The parent-child relationship may be affected by the quality of the marital relationship, and vice-versa (Erel & Burman, 1995, McKeown, et al., 2003).
**K3**	**Parental discipline** – This question collected information on which, and how consistently (*never* to *always*), parents use particular discipline methods. Discipline methods are seen as an important aspect of parenting and are considered as having an important influence on child behaviour and development (Grusec & Goodnow, 1994). Distinctions have been drawn between inductive techniques (such as explaining why a particular act is wrong) and punishment (e.g. smacking or shouting), with the former possibly more effective at internalising moral rules (Kerr, Lopez, Olson & Sameroff, 2004). There has been increasing debate in the media and in the academic literature about the effects of smacking, with most, but not all, studies reporting negative effects of using smacking as a discipline strategy (e.g. Gershoff, 2002). The same question was asked of the Study Children so that their reports can be contrasted with those of the parent/guardian. Further questions on parenting style were asked of the Study Children (see Chapter 8, Section 8.3.2). This question was adapted from the Millennium Cohort Study.
**K4 - K5**	**Family time together** – These questions were about time spent doing various activities together as a family such as eating a meal, playing games and going shopping, and how often Study Child spends time with or sees relatives. Parents influence their children by providing structure in their daily lives. Some findings have shown that regular, predictable routines, and time spent together impact more positively on children than when their family life is less organised (Boyce, et al., 1983). Since previous research in this area is limited, there is an opportunity for the current study to add to the research base. K4 and K5 were based on questions in the National Longitudinal Survey of Children and Youth, and Growing Up in Australia, respectively.

**K6**	**Work-life balance** – These questions related to work-life balance; not just the impact of work on family, but also of family on work. The issue of work-life balance is of increasing interest to researchers given the greater work demands placed on individuals and the larger number of women in the Irish workplace. Analysis will show whether study parent(s) are achieving an effective work-life balance, and whether this balance can be linked to any discernable outcomes for the Study Child. Any such linkage has potentially important implications for employment policies in relation to flexible working hours, for example. This question was previously used in Growing Up in Australia.
**K7 - K12**	**Religiosity** – These questions collected information on the denomination and religiosity of the main caregiver and the Study Child. Such questions provide important information in terms of being able to examine levels of religiosity in contemporary Ireland, one aspect of which is frequency of worship. It is also important in terms of understanding differences between children who are engendered with some form of religious upbringing and those who are not. Questions K7 and K8 were adapted from a range of surveys including the European Values Survey.
**K13**	**Household tasks** – Respondents were asked to rate on a three-point scale how fairly he/she felt household tasks were distributed between them and their partners. This question was also asked of the father/partner for comparison. As well as adding to the description of family relationships, it may also indicate the presence of gender roles within the household.
**K14**	**Child conduct disorder –** This set of questions asked about the Study Child's conduct over the last year. They were taken from the DSM IV classification of conduct disorder and include behaviours such as being physically cruel to other people or animals and deliberately damaging property. These items are indicative only and are not intended to diagnose any child with a psychiatric condition. This set of questions is relevant for the current cohort, since children who meet the full criteria for conduct disorder before puberty are more likely to have persistent conduct disorder, and are more likely to develop adult antisocial personality disorder than those with the adolescent onset type (American Psychiatric Association, 1994). Precursors of conduct disorder include difficult temperament, learning/reading difficulties, the child being bullied, and hyperactivity (as measured in the SDQ). This means that a longitudinal perspective on this issue can be taken in the current study.

7.1.11 SECTION L – SOCIODEMOGRAPHICS

This section recorded details on background characteristics of the household and/or Mother/Lone Father respondent, including information on household income.

**L1 - L6**	**Material Deprivation** – These questions recorded details on a number of aspects of material deprivation, which have been used in Ireland (and elsewhere) to provide indicators of non-monetary deprivation. The questions have also been used as input to a scaled variable of basic deprivation (see Chapter 6). When combined with indicators of relative income poverty the scale can be used to devise a measure of consistent poverty. These measures were derived from those used in the National Anti-Poverty Strategy (NAPS) (see, for example, Maitre, Nolan & Whelan, 2006.).
**L7 - L8**	**Nature of accommodation and status of tenure** – These questions recorded whether the household was located in a house, apartment, etc. and whether it was owner-occupied, rented, etc. Tenure status has been very widely used in ESRI surveys over several decades and adds variance explanation to measures of wellbeing independently of co-variates.
**L9 - L11**	**Bedroom Space –** Details were recorded on the number of bedrooms in the accommodation and whether or not the Study Child shared a bedroom with other

	children and with how many. This information can be used to derive an objective measure of space available in households given their size and composition. The international 'bedroom standard' (ODPM, 1998) can be used as a measure of crowding/bedroom density, etc.
**L12 - L22c**	**Principal Economic Status and related variables** – Depending on whether or not they were currently working (either as an employee, self-employed or farmer) outside the home, the respondent provided information on current or historic occupation and supervisory/managerial functions within the workplace. This information was recorded to allow a social class classification to be assigned to each household. This section also recorded details on number of hours worked outside the home (Q's L13 or L22b, depending on economic status). This information is clearly of direct relevance to issues of parental work-life balance, childcare, time spent with the Study Child, and the impact of these on the Study Child's outcomes (accounting for other co-variates).
**QL22d**	**Reason for not working in a full-time job** – This question was asked of those who did not work outside the home on a full-time basis (i.e. less than 25 hours per week). A choice of nine options was provided, including 'cannot find a job', and 'prefer to look after children oneself'.
**L23 - L27**	**Household income –** These questions addressed issues related to household income. L23 and L24 recorded the main sources of income received by the household. L25 - L27 recorded details on the level of household income. The concept is total household income from all sources and all household members, net of the statutory deductions of income tax and social insurance contributions (PRSI). This is a measure of the household's total disposable income. L25 offered the respondent the opportunity to record an exact figure per week/month/year. If this was not known or otherwise not forthcoming, L26 and L27 were then used to record the information using a series of rolling categories: the respondent was first asked to select which of the 10 categories his/her household falls into. This category was subsequently broken into sub-categories in an attempt to record the information on the most disaggregated basis possible. These income questions were used in the Living in Ireland survey, which is the Irish component of the European Household Panel Survey (ECHP). A major aim of this survey was to provide an up-to-date and comparable data source on personal incomes. There have been numerous publications based on the income data from this survey, particularly in the area of poverty and anti-poverty strategies (see for example Whelan, Layte, Maitre & Nolan, 2003).
**L28 - L32**	**Receipt of Social Welfare payments in the household** – All welfare schemes were listed (Q's L28 to L31b). We also recorded the household's estimate of its social welfare dependency (Q L32). This was included as a cross-check on the welfare dependency level which can be derived from the household income and receipt of welfare payments under various schemes. The details on Social Welfare receipts and dependency are interesting from a longitudinal as well as a cross-sectional perspective. Longitudinally they will allow an analysis of welfare receipt and transitions over time and their impact on child development.
**L33 - L36**	**Couple/Lone Parent income –** These questions recorded details on the net income accruing to the couple (or lone parent if relevant) where this was different from the total household figure.
**L37**	**Highest level of educational attainment** – This is a basic classificatory variable that is essential for analysis. Higher levels of maternal education on a child's development has been implicated in a number of domains: for example, accessing information in relation to child physical development (Thomas, Strauss & Henriques, 1991), and an enriched home learning environment in relation to child educational achievement (Christian, Morrison & Bryant, 1998).
**L38 - L42**	**Competence in English and other languages** – Information was collected on the languages spoken to the Study Child in the house. This section also recorded details on the respondent's functional and other literacy in English, and in their native

	language if not English or Irish.
**L43**	**Basic numeracy** – Respondents were asked whether or not they can usually tell if they have the correct change in shops from a five or ten euro note. The questions (or derivates) have been successfully used in other longitudinal child cohort studies such as the Millennium Cohort Study and give a broad indicator of basic numeracy.
**L44 - L53**	**Citizenship and length of time resident in Ireland** – Information was recorded on citizenship, country of birth, and residency in Ireland for both respondent and Study Child.
**L54**	**Ethnicity** – This information has substantive analytical benefit and may also be used as an input to the re-weighting of the data, having been taken directly from the most recent Irish Census of Population. This question was also asked in the Father/Partner and Non-Resident Parent questionnaires so that we have recorded ethnicity for both parents.
**L55 - L57**	**Receipt of regular care outside the home** – This information was used to provide the contact details necessary to administer the non-cohort caregiver questionnaire (where relevant).

7.1.12 SECTION M – NEIGHBOURHOOD AND COMMUNITY

In this section we recorded some background details on the characteristics of the neighbourhood or community of the Study Family. We also recorded the Study Child's family connections within the community.

**M1**	**Respondent's involvement in local voluntary organisations** – this item was derived from the NLSCY and is related to participation in the wider community as well as potential access to social networks.
**M2 - M3**	**Perception of neighbourhood as a place to live** – Questions M2 and M3 were designed to measure the respondent's perception of their local area, including subjective judgments of cleanliness, safety, public provision of play-spaces, etc. Question M2 was adapted from the Living in Ireland Survey (2000) and question M3 was derived from the Canadian National Longitudinal Study of Children and Youth (NLSCY)
**M4**	**Access to community and related services –** This question addressed the availability of a range of eight different types of services in the local community, including schools, clinics and recreational facilities for children.
**M5**	**Family living in the area** – This information relates to potential for personal support. Personal social networks, of both family and non-family members, can be an important support for parents. A personal social network can be a source of information (e.g. tips on child-rearing), practical assistance (e.g. child-minding) and emotional support. In a recent Irish study, 74% of parents identified their own family as a source of parenting influence and knowledge (Riordan, 2001).
**M6**	**Geographical situation of household** – This question has been used in numerous ESRI surveys over many years and is used in analysis according to area type. The respondent was asked to describe the area where the household was located. Options included open country, village, towns of various sizes, major cities, and Dublin county.

7.2 FATHER/PARTNER QUESTIONNAIRE

The Father/Partner questionnaire was administered to the resident spouse/partner of the main caregiver. This was usually the male parental figure in the household (generally the father of the Study Child). In situations in which, for example, the father of the Study Child clearly stated that he was the child's primary caregiver then he completed the longer, more detailed, primary caregiver questionnaire discussed in Section 7.1 above.

We outline very briefly below the main sections of the Father/Partner questionnaire. As this is almost exclusively a subset of the sections and questions from the Mother/Lone Father questionnaire, we do not discuss this questionnaire in detail, except for items that were not asked of the Mother/Lone Father. Cross-referencing is provided to fuller discussions of items elsewhere in this report. The Father/Partner questionnaire is in Appendix H. The Father/Lone Partner questionnaire contributes to meeting the study objective of providing a data bank on the whole child (#8).

7.2.1 SECTION A – INTRODUCTION

**A1**	**Relationship of respondent to the Study Child**

7.2.2 SECTION B – RESPONDENT'S HEALTH

**B1**	**Current health status of respondent** – as Section 7.1.5, E1
**B2 - B5**	**Chronic physical or mental health problems, illness or disability** – These included the nature, duration and constraints of current problem(s) (see Section 7.1.5, E2 - E8)
**B6 - B7**	**Current pregnancy status** – as Section 7.1.5, E12 - E13. Asked only if respondent was female.

7.2.3 SECTION C – RESPONDENT'S LIFESTYLE

**C1 - C4**	**Respondent's current and historic smoking patterns** – as Section 7.1.6, F1 - F4
**C5 - C6**	**Respondent's current alcohol consumption** – as Section 7.1.6, F5 - F6
**C7 - C8**	**Respondent's perception of weight** – as Section 7.1.6, F7 - F8
**C9 - C10**	**Respondent's self-reported height and weight –** as Section 7.1.6, F9 - F10

7.2.4 SECTION D – FAMILY CONTEXT

**D1 - D2**	**Parent-child relationship –** as Section 7.1.10, K1 - K2
**D3**	**Work-life balance** – as Section 7.1.10, K6
**D4**	**Household tasks** – as Section 7.1.10, K13
**D5**	**Perception of father role –** This was a ranking question in which the parent was asked to indicate the top three roles, in order, that he/she considered important to fulfil as a parent. A list, including 'showing my child love and affection' and 'taking care of my child financially' was provided, and there was also an option to specify and open-ended 'other' option. This question was intended to indicate how fathers/partners see

	their role and was also asked of non-resident fathers to facilitate comparison. This question was adapted from an item used by the Early Childhood Longitudinal Study.
**D6**	**Religiosity** – Father/Partner's religiosity.

7.2.5 SECTION E – SOCIODEMOGRAPHICS

**E1**	**Highest level of educational attainment** – as Section 7.1.11, L37
**E2 - E6**	**Competence in English and other languages** - as Section 7.1.11, L38 - L42
**E7**	**Basic numeracy -** as Section 7.1.11, L43
**E8 - E18c**	**Principal Economic Status and related variables** – as Section 7.1.11, L12 - L22c
**E18d**	**Reason for not working in a full-time job** – as Section 7.1.11, L22d
**E19 - E23**	**Citizenship and length of time resident in Ireland** – respondent's citizenship, country of birth, and residency in Ireland
**E24**	**Ethnicity–**- as Section 7.1.11, L54

7.3 SENSITIVE SUPPLEMENTARY SECTION

A common sensitive supplementary section was completed by *both the Mother/Lone Father and the Father/Partner*. As the questions were the same for both individuals, both questionnaires are covered in the following description.

The questions in the supplementary section were considered more sensitive than those in the main questionnaire and were included in a separate module for the respondent to self-complete on a PAPI basis. The questions covered issues about the marital relationship, marital conflict, experience of depression, feelings over the last week, use of drugs, and questions about a non-resident parent (if appropriate).

**S1 - S13**	**Relationship to Study Child –** This series of questions enquired about the respondent's relationship to the Study Child and whether he/she was the biological, adoptive or foster parent.
**S14 - S17, S26 - S27**	**Current and previous marital status –** These questions asked about current/previous marital status of parent(s) and, if appropriate, the number of previous partners who had had a close relationship with, or influence on, the Study Child. Research has repeatedly highlighted the link between family structure, changes in structure, and child outcomes. One such outcome found parental separation was linked to a significant increase in emotional/behavioural problems for the child even when demographic and other variables, such as marital quality, maternal depression, and socioeconomic circumstances were accounted for (Cheng, Dunn & Golding, 2006). Data from the current study should enable us to explore these factors, as well as links with others, such as the parent-child relationship, as a possible mediator of adjustment (Bernardini & Jenkins, 2002). Questions on current marital status have been used in a wide rage of ESRI surveys most notably in the ECHP – Living in Ireland survey (1994-2001).
**S18 - S25**	**Couple relationship** – These questions recorded details on length of time living together, frequency of arguments, and strength of relationship based on the DAS marital relationship scale (discussed in detail in section 10.4.3). Taking account of the information gleaned from both parents, analysis will highlight links between the couple relationship and outcomes for the Study Child. Marital conflict in particular can lead to an affective change in the quality of the parent-child relationship, which in turn has

	been shown to affect the child in terms of cognitive outcomes in young children, and social competence and work skills later in older children (Walsh, Clerkin & Nic Gabhainn, 2004). Research has also shown the spousal relationship to be the most important source of support for competent parenting (Belsky, 1984). Questions 18 - 22 were adapted from questions developed by researchers at Queen's University, Belfast.
**S29 - S30**	**Parental depression** – These questions enquired about past and current depression. Current depression (of both parents) was measured using the CES-D eight-item scale and related to feelings over the week preceding the survey. Both maternal and paternal depression have been linked to various child outcomes including children's socioemotional and cognitive development (Beardslee, et al., 1996). Although evidence for the link between parental mental health and child outcomes is unequivocal, many writers note that it often interacts with, or is associated with, other variables, such as a well-functioning family, that can either generate resilience (Dickstein, 2006), or increase risk, such as poverty (Eamon & Zuehl, 2001). The CES-D eight-item scale is discussed in more detail in section 10.4.2.
**S31**	**Parental drug use –** This question asked about the incidence and frequency of parental use of various drugs over the year preceding the survey. The list included prescription drugs as well as illicit drugs such as cannabis, amphetamines, heroin and cocaine. While research on the effects of parental drug use on children has typically highlighted such problem behaviours as antisocial behaviour, and conduct or oppositional disorders (e.g., Smith, 1993; Willens, et al., 1995), and negative impacts on the quality of parenting provided for the child (Dawe, et al., 2007), more recent research has begun to focus on the child's competencies and resiliency (Pilowsky, et al., 2004).
**S32 - S33**	**Parental contact with the Criminal Justice System** – These questions asked whether parents had been in trouble with the Gardai (the Irish police service) or ever been to prison. Findings from the Head Start programme in the US have found that children whose family members had contact with the criminal justice system were more likely to be described as having problem behaviour by parents and teachers, and were also likely to score lower on assessed vocabulary. Findings also show that substance abuse, domestic violence, parental mental illness, and poverty are more prevalent in households where parents have been arrested. However, it is important to remember that children of parents involved with the criminal justice system are not a homogenous group. While the overriding problem in some households may be extreme poverty, for others there may be a multitude of problems (Phillips & Gleeson, 2007), all of which need to be considered within the boundaries of the current study.
**S34 - S49**	**Non-resident parent** – If there was a non-resident parent then the respondent was asked a series of questions about his/her relationship with that person, when the relationship ended, the nature of the relationship when pregnancy occurred, custody and parenting arrangements, financial contributions (of the non-resident parent), and contact with the Study Child. These questions were also asked of the non-resident parent, and the logic behind them is outlined in section 9.1 (non-resident parent questionnaire). Asking the primary caregiver these questions enables comparisons in the information given by both parents, while also ensuring that the information is gleaned from at least one source, especially where contact details are not available for, or it is not possible to contact, a non-resident parent. Questions S35 - S36 and S42 - S44 were derived from the Growing up in Australia Study and S48 from the Millennium Cohort Study.

Chapter 8

INSTRUMENTS COMPLETED BY CHILDREN

CHAPTER 8: INSTRUMENTS COMPLETED BY CHILDREN

This chapter details the instruments used with the Study Children in the home. We start with the main questionnaire then move on to the core sensitive questionnaire and sensitive supplements dealing with individual parents/guardians/parental figures. The child questionnaires are a key part of achieving study objective #7, to obtain the views and opinions of children on their lives. Where no question sources are specified, these questions have been developed by ***Growing Up in Ireland***, typically in conjunction with the expert panels (section 3.4). We also describe the time-use diary which was completed by the parent/guardian with the help of the Study Child and this describes the child's activities for a specified 24-hour period while contributing to objective #1, to describe the lives of Irish children.

8.1 THE CHILD'S QUESTIONNAIRE

Interviewers provided the parent(s)/guardian(s) of the Study Child with a *blank*[10] copy of the Child's questionnaire for inspection before the Study Child completed it. The Child's questionnaire contains main, core and sensitive sections (as detailed in Appendix I). The main part of the questionnaire was administered to all children on a CAPI basis. More sensitive questions, such as those about relationships, were presented separately for self-completion on paper (with audio CD available to help less confident readers), as part of the Child's Sensitive/Supplementary Questionnaire. As with the adult respondents, when the children were finished filling out the sensitive supplements they were provided with an envelope in which to place their completed questionnaires.

In all cases, the children were reminded that there were no 'right' or 'wrong' answers and that they should take their time. The interviewer training encouraged interviewers to develop a rapport with the children by asking informal questions about their day and their favourite activities. The interviewer was instructed to sit at the same level as the children so as not to intimidate them, and to explain the materials being used and the type of questions that would be asked. A parent or guardian was present in the room at all times. However they were asked to complete their own sensitive questionnaires or continue with household tasks out of earshot. If the child became upset or distressed the interviewer was instructed to ask the parent/guardian to intervene and comfort the child. If, after a short break, the child was happy to continue the interview would recommence. Interviewers were instructed to offer the child a break during the interview and to inform the child that they could terminate the interview at any point.

All parts of the Child's Questionnaire are in Appendix I.

8.2 CHILD MAIN QUESTIONNAIRE

The main questionnaire was made up of four sections covering school, food, activities and likes/dislikes.

8.2.1 SECTION A: SCHOOL

This section focused on participants' perceptions of aspects of their current school life, such as academic performance and liking of various subjects.

**A1 - A4**	**Study Child's feelings about school and schoolwork** – These questions are relevant to current school experiences that can influence children's self-concepts, attitudes towards school and future educational outcomes. Children's beliefs in their

[10] It was made clear to all respondents in advance that none would have sight of a questionnaire or other instrument completed by any other respondent in the survey. This extended to parents/guardians having sight of children's questionnaires, test scores in the Drumcondra assessments, or teacher questionnaires.

	efficacy to regulate their own learning activities and master difficult subjects affect their academic motivation, their interest and their academic achievement (Bandura et al, 1996). These data can be linked to gender and performance on the Drumcondra attainment tests. The questions are adapted from items used in the National Longitudinal Study of Children and Youth.
**A5**	**Study Child's perception of his or her family's socioeconomic status** – Children are asked if they think that their families are better off (in terms of having a bigger house or car, for example) than the families of their classmates, the neighbours, and other families in Ireland. Responses may be analysed in terms of the possible influence of such perceptions on children's wellbeing and peer-relations, and how consistent they are with actual income and parental perceptions of economic status (as assessed in the main caregiver questionnaire). This subjective approach is based on research findings from adult studies that indicate how an individual's perception of his or her social standing may be more important to health outcomes than objective measures (Adler, Epel, Castellazzo, & Ickovics, 2000).

8.2.2 SECTION B: FOOD

**B6**	**Yesterday's Diet** – This section asked children to record which foods, from a list of ten, they ate on the day preceding the survey. Children who ate any of the listed foods were asked to indicate, for each, if they ate one serving or more than one serving. The list included fresh fruit, vegetables, animal products (dairy and meat) and processed foods (e.g. biscuits and cakes). This question is related to how children's diet can affect future health and weight. For example, Higgins, McArdle, McEvoy, & Tully (2005) have noted how many Irish children have inadequate intakes of calcium, iron, vitamins and folate, which may be detrimental to their health in the short-term, as well as increasing risk for a number of chronic diseases in the long-term. This item was adapted from Growing Up in Australia.

8.2.3 SECTION C: ACTIVITIES

Section C was concerned with investigating how children spend their time away from school and with whom they spent it.

**C7a-i**	**Activities engaged in with parents** – Children indicated which activities from a list of nine they had done with their parents in the preceding week. This list included, among others, sharing a meal, watching television and going to the park. Time spent together is relevant to the parent-child relationship, family dynamic and child wellbeing. In addition, parents are significant socialisation agents responsible for the leisure interests and values that their children develop (Barnett & Chick, 1986; Kleiber, 1999). This question was based on an item used in the National Longitudinal Survey of Children and Youth.
**C8 - C11**	**Computer use** – Children were asked about their experience with computers including ownership, usage and adult supervision of the internet. Children who come from homes that cannot afford a computer may be at a disadvantage compared to classmates who can practise their computer skills at home and use the internet as a resource for school projects (Malcolm, 1988, as cited by Santrock, 1998). These questions are also relevant to the potential risk that exposure to the internet can entail.
**C12 - C13**	**Preferred Pastimes** – The Study Child ranked, in order, first, second and third his/her most preferred pastimes from a list that included playing sports, hanging out with friends and listening to music. A further open-ended question asked him/her to name

	his/her favourite hobby or activity. The questions gathered descriptive data on the preferred pastimes of nine-year-old children in Ireland. Findings from a recent Irish study of children aged from 4 to 12 years indicated that sports and other outdoor activities are very popular, but when children are playing alone technology is a common source of entertainment (Downey, Hayes & O'Neill, 2007). The importance of pastimes and hobbies was highlighted by the participants in the Children's Advisory Forum (see Chapter 3, Section 3.3 for further information on the CAF).
**C14 - 16, C23**	**Sports and exercise** – The Study Child was asked about his/her participation in sports and exercise. Children who do not play sports were asked to specify the main reason why this was the case. These questions are related to how children's current exercise behaviour can affect their health and wellbeing. For example, Strauss, Rodzilsky, Burack & Colin (2001) have outlined how, in addition to immediate physiological benefits, exercise is associated with increased self-confidence and social connectedness.
**C17**	**Frequency of reading for pleasure** – This activity may be related to academic performance at school. For example, a recent study in England and Scotland found that only 33% of children read for pleasure, compared with the international average of 40%. In addition, there is a link between children's reading for pleasure and their achievement in reading tests (Baer, Baldi, Ayotte & Green, 2007). This question was previously used in the National Longitudinal Survey of Children and Youth.
**C18**	**Ownership of mobile phone –-** This question recorded descriptive data on whether the respondent owned a mobile phone. Research investigating cellular mobile phone ownership among a sample of primary school pupils in the UK indicated that nearly half of both girls and boys owned mobile phones (Charlton, Panting, & Hannan, 2002). Data on mobile phone ownership among children may be relevant to research on bullying (as a medium for bullying), and health-related issues.
**C19**	**Self-care –** This question collected information on the types of self-care activities which nine-year-old children are expected to do. Children chose from a list of self-care activities including showering, making breakfast and tidying their rooms. As children mature, they need adult supervision that is increasingly indirect, distal, and based on a parent-child relationship of open communication (Riley & Steinberg, 2004). This question was based on an item used by ALSPAC.
**C20**	**Helping with chores** – From a list of household chores (e.g. washing dishes, cleaning the car, vacuuming) children were asked to indicate which ones they did occasionally, often or never. This question is particularly relevant to children who may be engaged in a caring role within the home. In addition, self-care is a family adaptation rather than a characteristic of the individual child and should not be treated in isolation from the family (Riley & Steinberg, 2004).
**C21a-b**	**Presence and impact of illness, disability or medical condition** – The children were asked if they had any diagnosed long-term illness, disability or medical conditions such as asthma or diabetes. If they did, they were asked if their condition affected their attendance or participation in school. This question relates to the impact that a physical or psychological illness or disability can have on a child's quality of life, socioemotional development and education. For example, Sweeting & West (2001) have found that characteristics of appearance, disability or ability can increase the likelihood that a child will experience bullying.
**C22**	**Perception of weight** – The Study Child was asked how he or she would describe his/her physical appearance with regard to his/her weight. Response options ranged from *very skinny* to *very overweight*. As the child's actual height and weight was measured by an interviewer, this question provides an indication of the child's self-perception of his/her physical build (Collins, 1991), and links to questions on health and body image in the qualitative study. The Mother/Lone Father was also asked to assess the appropriateness of the child's current weight.

8.2.4 SECTION D: LIKES AND DISLIKES

This section included a number of open-ended questions that assess children's likes and dislikes. The last questions in this section asked about the presence of a pet in the child's family and activities that the child likes to do with his/her pet.

**D24**	**The occupation Study Child would like to fulfil in adulthood** – This was an open-ended question on what the child 'wants to be when they grow up'. The answers to these questions may be of interest to those looking at, for example, gender differences in motivation, self-concept, and views on appropriate activities for males and females. These constructs can have causal influences on cognitive outcomes such as school achievement and occupational choice (Wigfield, Battle, Keller & Eccles, 2001).
**D25**	**Person Study Child most admires** – Children were asked to choose one from a list of nine, such as TV star, mum or dad, pop star, or self-specified. Little is known about what role models Irish children admire and may wish to emulate. These data provide longitudinal and 'period effect' possibilities.
**D26**	**Three open-ended questions** – The Study Child was asked to complete three sentences in his/her own words: (a) a thing makes me most happy, (b) the thing I am most afraid of, and (c) why I like living in Ireland. The answers to these questions should provide interesting descriptive data with possibilities for linkage to other characteristics such as socioeconomic status and ethnic/cultural background.
**D27 - D29**	**Pet ownership** – The Study Child was asked what type of pet he/she had and what he/she liked about it. Children could indicate multiple options from a list including, 'I like to look after them' and 'They make me feel loved'. These data are mainly descriptive but with possible links to wellbeing and future attitudes to responsibility. For example, Van Houtte & Jarvis (1995) found support for their hypothesis that pre-adolescent pet owners would report higher autonomy, self-concept and self-esteem.

8.3 SENSITIVE QUESTIONNAIRES FOR NINE-YEAR-OLD CHILDREN

Although there is essentially one sensitive section to the Child's questionnaire, it is split into five parts to allow for alternative family structures. The first part of the sensitive section is the Core Sensitive Questionnaire. This was administered to all children. Questions elicited views on topics such as where the child lives and his/her experience of bullying. The other four parts of the questionnaire (on four individual, colour-coded sheets) recorded details of a child's view of the relationship he or she shared with his or her biological mother (coded M: Mum Questionnaire), biological father (coded D: Dad Questionnaire), mother's partner (coded MP: Mum's Partner Questionnaire), and father's partner (coded DP: Dad's Partner Questionnaire), as applicable.

The interviewer determined in advance of each child's interview which of the four sections the child should complete following discussion with the primary caregiver, that is the respondent who completed the Mother/Lone Father questionnaire. The primary caregiver was shown a prompt card (see Table 8.1) and the different family structures listed on it were clearly explained. Therefore, he/she controlled which supplementary sections his/her child completed. For example, a lone mother who completed the Mother/Lone Father questionnaire could request that her child not be asked questions in respect of his/her biological father.

Table 8.1: Table for determining family composition and child sensitive supplements required

Family Composition	Questionnaire
A. Mother and father (biological/adoptive)	M and D
B. Mother and her partner (contact with biological father in previous 12 months)	M, MP and D
C. Mother and her partner (no contact with biological father in previous 12 months)	M and MP
D. Mother with no partner (contact with biological father in previous 12 months)	M and D
E. Mother with no partner (no contact with biological father in previous 12 months)	M
F. Father and his partner (contact with biological mother)	D, DP and M
G. Father and his partner (no contact with biological mother in previous 12 months)	D and DP
H. Father with no partner (contact with biological mother in previous 12 months)	D and M
I. Father with no partner (no contact with biological mother in previous 12 months)	D

8.3.1 CORE SENSITIVE QUESTIONNAIRE

All children completed this questionnaire.

8.3.1.1 SECTION A: WHERE YOU LIVE

**A1 - A15**	**Characteristics of Study Child's local area** – This was an introductory section that comprised fifteen questions about the areas in which participants live, such as presence of local clubs and playgrounds, cleanliness of the streets, access to public transport, and feeling safe. Research in the United States suggests that neighbourhood can affect child outcomes through such aspects as quality of parks, playgrounds, and conditions of mutual trust and shared expectations among residents (Brooks-Gunn, Duncan, Klebanov, & Sealand, 1993).

8.3.1.2 SECTION B: SCHOOL

This section initially focused on participants' feelings towards school and teacher and moved on to their experience of bullying.

**B16 - B17**	**Looking forward to attending school and liking school teacher** – These were introductory questions to settle the child and are related to Section A1-4. These questions investigated whether children's views and experiences affect views of their own abilities to learn and their actual achievement and adjustment (Eccles, Wigfield, & Schiefele, 1998). These questions were based on items used in the National Longitudinal Survey of Children and Youth.
**B18 - B19**	**Picking on another person** – These questions asked the child about being a bully in the last year, and if relevant, the type of bullying he/she carried out. These and subsequent bullying questions were developed in conjunction with the Anti-bullying Centre at Trinity College Dublin. This information is related to how experiences of perpetrating bullying can impact upon a child's psychological wellbeing, as well as

	factors that may promote bullying behaviour. Olweus (summarised by Bee & Boyd, 2007) proposes four factors that contribute towards the development of a bully: experience of indifference and lack of warmth in the early years, parental failure to set clear limits on aggressive behaviour, parents' use of physical punishment, and a difficult, impulsive temperament in the child.
**B20 - B21a-b**	**Being picked on by another person** – These questions were about being a victim of bullying in the last year as well as how much they were upset by the experience. This information is related to how experiences of bullying can impact upon children's psychological wellbeing. Research indicates that the effects of victimisation include loneliness, school avoidance and reduced performance, low self-esteem, panic attacks, digestive disorders, and significant depression both at the time and in later life (Bee & Boyd, 2007; Anti-Bullying Centre, 2002).

8.3.1.3 SECTION C: FAMILY

**C22 - C23**	**Presence of siblings and relationship with them** – These were introductory questions to thinking about family. This question is related to family make-up and functioning (Dunn, 1996). This information supplements the information on household structure (nature of sibling relationships, relative ages, birth order, etc) collected in the first part of the Mother/Lone Father interview.
**C24**	**Whom Study Child talks to about a problem** – The Study Child selected from a list of people including parents, teachers and friends all those whom they would talk to about a problem. Faber and Mazlish (1999) advise that children need to have their feelings accepted, respected and acknowledged, and not just agreed with. This question will provide descriptive data on the people nine-year-old children choose to discuss their difficulties with. This is also explored in depth in the qualitative study. An Irish survey (HBSC, 2002) found that 78% of children aged 10-17 found it easy to talk to their mother when something was bothering them and, of those, the percentage was higher among girls, younger children and children from lower social groups.
**C25**	**Deciding family events** – This question was included to indicate how many nine-year-olds have a say in family events such as outings and leisure pursuits. The extent to which children are involved in family decisions in Ireland is not known. The UN Convention on the Rights of the Child (Unicef, 1989) sets out to<br><br>… assure that the child who is capable of forming his or her own views has the right to express those views freely in all matters affecting the child, the views of the child being given due weight in accordance with the age and maturity of the child.<br><br>Baumrind (1991) reports that social and academic outcomes of children are positive when parents set firm limits in the context of a warm, supportive relationship and encourage age-appropriate autonomy.

8.3.2 SUPPLEMENTARY SENSITIVE QUESTIONNAIRES

As described above, the same questions were asked in respect of mother, father, mother's partner and father's partner as applicable.

**1**	**[Mother's] encouragement of performance at school** – This question is related to how parental aspirations for, and involvement in, children's education can impact upon

	children's educational outcomes. Research in the UK found that parental involvement has significant effects on achievement into adolescence (Feinstein, 1999). This item was adapted from a similar question asked in the National Longitudinal Survey of Children and Youth.
**2**	**Getting along with [Mother]** – This was a general question on how the Study Child gets on with the individual parent/parental figure. Research in Ireland for the Health Behaviours in School Children survey suggests a link between parent-child relationship and self-ratings of good health and happiness among children aged 10-11 years (Walsh, Clerkin, & Nic Gabhainn, 2004). This question was previously used in the National Longitudinal Survey of Children and Youth.
**3a-j**	**Parenting Style Inventory II (Adapted) –** Following on from question 2, the PSI-II aims to assess the overall emotional climate in which parent-child interactions occur. Question 3 on the Supplementary Sensitive Questionnaire contained ten items from this scale. Parenting styles characterised by high warmth and high control have been widely associated with positive child outcomes (e.g. Steinberg, Elmen, & Mounts, 1989; Avenevoli, Sessa, & Steinberg, 1999). More information on the PSI II is included in Chapter 10, Section 10.4.5.
**4**	**[Mother's] reaction to 'bold' behaviour** – This question, with minor wording changes, was the same question as asked of the Mother/Lone Father in relation to the use of various disciplinary practices including smacking. Asking the Study Child for the same information allows the child's perspective on his/her experience to be noted in addition to that of the parent/guardian. It is also collected for each parental figure allowing comparisons to be drawn, for example between maternal and paternal discipline. See Chapter 7, Section 7.1.10, K3 for further discussion. The question was adapted from a similar item used in the Millennium Cohort Study.

8.4 SELF-COMPLETION TIME USE DIARY

At the end of the interview the interviewer left a copy of a self-completion time-use diary with the respondent who completed the Mother/Lone father questionnaire and asked him/her to fill it out with the Study Child on a specified date[11], for return to the Study Team by post in a prepaid envelope. The purpose of the time-use diary was to record what the Study Child did for each 15-minute slot during the reference day for the diary from 12.00 midnight until 12.00 midnight[12].

A worked example of the time-use diary was explained by the interviewer and left with the respondent**.** A specified date for filling out the diary was filled in on the front cover by the interviewer before leaving the household. The interviewer asked the respondent to complete the diary with the Study Child either in the course of the 'diary day' or on the following day. The 'diary days' were allocated to respondents in such a way as to provide a sample of days throughout the week. A copy of the time-use diary is given in Appendix L.

There were a total of 21 activities used in the time use diary

1. Sleeping
2. Resting/relaxing – taking time out, doing nothing
3. Personal care – getting washed, dressed in the morning – in the shower, bath, toilet, etc.
4. Eating/drinking – any type of food or drink taken during the day

---

[11] Day for completion is provided on the interviewers Work Assignment Sheet. This was transferred to the time-use diary by the interviewer.
[12] The structure, format and implementation of time-use diary was taken from a national study carried out by the ESRI in 2005. See McGinnity, Russell, Williams & Blackhall (2005). It is of a similar format to that used by Growing Up in Australia.

5. Travelling to and from school
6. Other travelling – travelling anywhere, possibly travelling to and from the shops on a shopping trip, etc. (Does not include travel to school.)
7. School – time spent in school
8. Homework – time spent doing homework
9. Physical play/sports/physical exercise – this specifically refers to physical games or sports.
10. Playing board games, cards – this specifically refers to some form of board game, cards, etc.
11. General play – this would include general playing at games – inside or outside – ‘playing house’, playing with dolls, imaginary games, cowboys and Indians etc.
12. Hobbies and other leisure activities – (crafts, painting, etc.)
13. Computer/internet/PlayStation/X-BOX, etc, – this is time spent using the computer or computer games at home for various activities.
14. Email/Bebo/Msn/Texting/On the phone – messaging, contacting friends or others
15. Watching TV, videos/DVDs, etc.
16. Reading books, comics, magazines – this covers reading for pleasure outside of school and homework.
17. Household chores – refers to any chores the Study Child has to do around the house.
18. Visiting a relative’s house for some purpose other than to play
19. On a family outing – this is a trip out as a family.
20. On a shopping trip – it could be for grocery shopping, clothes, etc.
21. Religious activity – attending religious services, prayer, etc.
22. Not sure

Chapter 9

OTHER INSTRUMENTS

CHAPTER 9: OTHER INSTRUMENTS

This section details the other instruments used to collect data. They contribute to objective #8, to provide a data bank on the whole child. In the first two sections we describe the three types of postal self-completion questionnaires that were used: the non-resident parent questionnaire and the two versions of the regular carer questionnaire. Where no question sources are specified, these questions have been developed by ***Growing Up in Ireland***, typically in conjunction with the expert panels (section 3.4). The latter part of the chapter deals with the physical measurements, interviewer observations and other information that was recorded by the interviewer.

9.1 NON-RESIDENT PARENT QUESTIONNAIRE

If applicable and if the Mother/Lone Father gave permission, the interviewer recorded the contact details of the biological non-resident parent for the purposes of sending out a self-completion questionnaire to that parent. There follows a detailed description of the questions contained in the non-resident parent questionnaire. An almost identical questionnaire was sent to non-resident fathers and mothers, but with questions relating to naming on the birth certificate and guardianship removed. The Non-Resident Parent Questionnaire (father's version) is included in Appendix J.

**Q1 - Q8**	**Contact visits with Study Child –** These questions collected information about the parent's personal visits with the child including length, timing and location of visits, satisfaction with amount of contact, reasons for dissatisfaction, and how visits were determined. These questions will help to describe the time non-resident parents spend with their children and how this affects current and future child wellbeing. The opportunity for overnight stays has been highlighted as affording greater opportunities for engagement in a parenting role (Parkinson & Smyth, 2003). Question 1 was previously used by the Early Childhood Longitudinal Study and Questions 2, 3 and 5 were previously used by Living in Australia (HILDA).
**Q9**	**Perception of parental role –** This was a ranking question in which the respondent was asked to indicate the top three roles, in order, that he/she considered important to fulfil as a parent. A list, including 'showing my child love and affection' and 'taking care of my child financially' is provided, and there is also an option to specify an open-ended 'other' response. This question is intended to indicate how non-resident parents see their role and was also asked of resident father/partners to facilitate comparison. This question was adapted from a question asked by the Early Childhood Longitudinal Study.
**Q10 - Q11**	**Contact other than personal visits –** These questions asked about the amount and type of contact between parent and Study Child other than personal visits. The information gathered may help to indicate the importance and impact of this type of contact in contemporary Ireland. It has been suggested that phone calls may be used as a substitute for personal visits, particularly for parents living some distance away (e.g. Skevik, 2006).
**Q12**	**Rating of quality of time spent with the Study Child** – Parents were asked to rate the perceived quality of time they spend with the Study Child on a scale of 1-5, where 1 = excellent and 5 = very poor. This information may be considered in relation to effects on child development/outcomes. Commentators in the international literature discuss the negative quality of 'Disneyland Dad'- type contact of short, recreational based visits (e.g. Kielty, 2006).
**Q13**	**Non-resident parent's performance of routine caring tasks** – This item asked how often the parent performed routine care tasks for the Study Child, such as preparing meals and helping with homework, which may be related to the parent's engagement

	in an authoritative parenting role as opposed to simple companionship. The exercise of authoritative parenting by non-resident (and resident) fathers has been strongly linked to positive child outcomes (Amato & Gilbreth, 1999), and in their meta-analysis was operationalised by the performance of tasks such as helping with homework. This question was adapted to tasks more relevant to nine-year-olds from a similar item asked by the Early Childhood Longitudinal Study.
**Q14 - Q18**	**Amount of financial and other support provided to the Study Child** –The answer to this question may be used in examining the resources available to the Study Child. This information can also be contrasted with similar information provided by the resident parent. Questions 14, 17 and 18 were based on questions used by the Early Childhood Longitudinal Study and Question 16 was adapted from Living in Australia.
**Q19**	**Status of relationship with Study Child's mother/father at pregnancy** – This question asked the parent to describe the status of his/her relationship at time of conceiving the Study Child. This status may affect subsequent contact between non-resident parent and Study Child. Many studies suggest that a father will be more likely to maintain contact if he has been married to, or at least cohabitating with, the mother (e.g. Argys, Peters, Cook, Garasky, Nepomnyaschy, Sorensen & Waller, 2003; Clarke, Cooksey & Verropoulou, 1998; Skevik, 2006), although some variation as to the relative effect of marriage versus cohabitation has been observed between cultures. This question was adapted from the Millennium Cohort Study.
**Q20**	**Age of Study Child when parents split up** – Information on the timing of the parental separation is important for considering potential timing effects of separation on child outcomes, for example whether earlier or later separation is less stressful, and this occurrence would be also a major event in the analysis of individual life pathways. This question was based on an item from Growing Up in Australia.
**Q21**	**Father's name on birth certificate (not asked of non-resident mothers as not applicable)** – This question was asked of fathers only if they were named on the Study Child's birth certificate, with a view to considering how this status might affect subsequent contact. An American study of 'fragile families' by Lundberg, McLanahan & Rose (2005) found that fathers were more likely to maintain contact with their children if they were named on the birth certificate. This question was adapted from the Millennium Cohort Study.
**Q22 - Q23**	**Application for guardianship status (not asked of non-resident mothers as not applicable)** – This question asked fathers who were not married to the Study Child's mother if they had applied for guardianship status, if this application was through the mother or the courts, and if the application was successful. It will provide useful information indicating the number of fathers who take up this option and whether the status affects their involvement with their children (see previous discussion on potential impact of being named on the birth certificate).
**Q24 - Q26**	**Current contact with the Study Child's mother/father** – These questions asked about current contact with the child's other biological parent including frequency of contact, tone of relationship and influence on decisions concerning the Study Child, with a view to examining how these aspects of the parental relationship affected the child. Amato & Rezac (1994) reported that contact with non-resident fathers is related to positive outcomes for the child when the parents have a co-operative relationship but not when they are in conflict. Questions 24 and 26 were based on questions used in the Early Childhood Longitudinal Study, and Question 25 came from the Millennium Cohort Study. [Questions 21-23 on Non-Resident Mother's Questionnaire]
**Q27 - Q28**	**Parent's date of birth and age at which he/she first became a parent** –This question was asked with a view to examining if a particular age-group of fathers/mothers are more or less likely to maintain contact. This information may also help to describe the profiles of non-resident parents in Ireland today. This question was also asked in the Early Childhood Longitudinal Study. [Questions 24-25 on Non-Resident Mother's Questionnaire]

Q29 - Q31	Socioeconomic status – These items provided a means of estimating the parent's socioeconomic status including employment and occupation. Socio-economic status is likely to affect the resources and/or time the parent has available to give to the Study Child. Parents of lower means may be less able to afford the cost of either maintaining accommodation suitable for keeping a child overnight or travelling some distance to visit their children. Similar questions have been asked in many surveys undertaken by the ESRI. [Questions 26-28 on Non-Resident Mother's Questionnaire]
**Q32 - Q35**	**Current family/relationship status** – These questions about current marital status and the presence of a new partner and other children are asked with a view to indicating how commitments to other families affects contact with and resources available to the Study Child. The findings on the impact of a 'new' family on contact with the 'old' family are conflicting, with some suggesting that contact remains steady (Skevik, 2006), and others that it decreases (e.g. Parkinson & Smyth, 2003). [Questions 29-32 on Non-Resident Mother's Questionnaire]
**Q36 - Q37**	**Parent's nationality and residence in Ireland –** This question provided important information on the ethnic origins of parent and child, and the length of parent's residence in Ireland. [Questions 33-34 on Non-Resident Mother's Questionnaire]
**Q38**	**Parent's state of health –** As for the health of the mother and resident father [Question 35 on Non-Resident Mother's Questionnaire]

9.2 CARER QUESTIONNAIRES

If another person provided care to the Study Child for eight or more hours a week on a regular basis, then the interviewer asked the Mother/Lone Father for permission to send out a questionnaire to the carer, and recorded the contact details. There were two different questionnaires: one for carers based in a home situation, and one for carers employed at a care-centre such as a homework club. The carer questionnaires are in Appendix K.

9.2.1 HOME-BASED CARER QUESTIONNAIRE

**Q1 - Q2**	**Relationship of carer to Study Child –** These questions asked the carer to describe his/her relationship with the Study Child (relative/non-relative/childminder) and if he/she lived with the Study Child. Findings in Ireland from the Quarterly National Household Survey (2005) found that nearly 10% of families with primary-school-going children used childcare provided by unpaid relatives. Questions 1 and 2 were previously used in Growing Up in Australia and the Early Childhood Longitudinal Study respectively.
**Q3 - Q6**	**Details of care provision –** These questions collected information on the location, hours, days and duration of care, to see how variations in these might affect child development. As well as providing descriptive information, these details are of interest in considering the effects of long periods of time in non-parental care. For example, Pettit, Laird, Bates & Dodge (1997) found a curvilinear relationship of time spent in non-parental care (where little or none and excessive amounts were both associated with negative child outcomes) for both neighbour-orientated and activity-oriented care. Questions 3 and 6 were similar to items used in the Early Childhood Longitudinal Study and Question 4 was used in Growing Up in Australia.
**Q7**	**Activities of Study Child while in care** – This item asked the carer how the frequently the Study Child engaged in activities such as watching TV, doing homework and reading while in the respondent's care. This information will supplement that provided by parents and in the time-use diary.

Q8	**Carer's perception of relationship with Study Child –** This information will supplement that provided by teachers and parents and will inform the picture of the overall care environment. This question came from Growing Up in Australia.
Q9 - Q10	**Other children in care situation** – These questions asked about other children being looked after by the home carer, including the number and ages of these children, with a view to seeing how time spent with other children, particularly older children, might affect the Study Child's socialisation. These questions were based on similar items used in Growing Up in Australia.
Q11	**Number of children's books available to Study Child** – This item complements information on books available in Study Child's home (if different), and was also used in Growing Up in Australia.
Q12	**Looking after Study Child when sick** – This question asked about caring for the Study Child when sick, looking at the potential for exposure to infections in childcare situations, and how many carers facilitate parents when children are sick. A similar question was asked by the Early Childhood Longitudinal Study.
Q13 - Q15	**Demographic characteristics of carer** – These questions provided basic information on the carer, including date of birth, gender and nationality.
Q16 - Q18	**Occupational/employment status of carer** – These questions asked about the carer's main occupation, if this was not childcare, and will be used mostly as descriptive information.
Q19 - Q21	**Education and training of carer –** These questions asked about the carer's education including childcare qualifications, other related training and highest educational level achieved. The information was sought with a view to considering how training affects quality of childcare as seen in child outcomes.
Q22 - Q23	**Carer's experience working in childcare and length of time spent in childcare each week** – These items reflected the carer's childcare experience and may affect the quality of care received by the Study Child.

9.2.2 CENTRE-BASED CARER QUESTIONNAIRE

Q1 - Q3	**Details of care provision –** as for Section 9.2.1, Qs 4-6
Q4	**Carer's perception of personality of Study Child compared to other children –** This information supplements that provided by teachers and parents. This question was previously used by Growing Up in Australia.
Q5, Q15 - Q16	**Activities of Study Child while in care, activities and books available** – as for Section 9.2.1, Qs 7 and 11, except for Q15, which asked for further details on facilities available to the child, such as organised team games
Q6	**Carer's perception of relationship with Study Child –** as for Section 9.2.1, Q8
Q7	**Is centre registered with HSE? –** This item provided descriptive information, with a view to indicating how many care centres for older children are registered with the Health Service Executive (national regulatory body in Ireland).
Q8 - Q12	**Other children in care situation –** These questions asked for details, including number and ages of these children, non-national and disabled children, with a view to examining how these variables might affect the Study Child's socialisation and quality of care. They overlap with Qs 9 and 10 on the Home Carer's questionnaire and with the additional questions on children with disabilities and other nationalities. These questions were based on items used in Growing Up in Australia.
Q13 - Q14	**Details of centre staff** – These items collected information on centre staff, including total numbers and those with childcare qualifications, with a view to considering how differences in training affect quality of care. Studies with younger children find that centre-based care provision is better when staff-child ratios are lower and when staff

	are better trained (e.g. NICHD Early Child Care Research Network, 2002). These questions were adapted from Growing Up in Australia.
**Q17**	**Looking after Study Child when sick** – as for Section 9.2.1, Q12. Research with younger children suggests that those in centre-based care suffer more illness (NICHD, 2001; 2003).
**Q18**	**Position of respondent in the care setting** (director or employee) – This classification allows the information supplied by the respondent to be put in context.
**Q19 - Q21**	**Demographic characteristics of carer** – as for Section 9.2.1, Qs 13-15
**Q22**	**Type of care provided by centre** – Respondent described the type of care provided in the centre, including supervision only, study group, etc. As well as contributing descriptive information, this item facilitates the possibility of looking at differing effects of different types of care, such as homework club versus supervision only.
**Q23 - Q26**	**Education and training of carer** – These questions sought details on the qualifications and training of the respondent carer. See earlier questions 13-14 above on implications for staff training.
**Q27 - Q28**	**Occupational/employment status of carer** – as for Home Carer
**Q29 - Q30**	**Carer's experience working in childcare and length of time spent in childcare each week** – as for Section 9.2.1, Qs 22-23
**Q31**	**Carer's job satisfaction** – Respondent was asked to rate how happy they were working in childcare, with a view to assessing the possible impact on quality of care and subsequent child outcomes.

9.3 OTHER INSTRUMENTS

9.3.1 FOLLOW-UP INFORMATION

At the time of the household interview, the Mother/Lone Father was asked if he/she would be willing to provide a PPS number and/or an alternative (possibly more stable) contact address for another person who would be likely to know the family's location in four years' time, should the Study Family have moved between surveys. These details were filled out on a separate follow-up information sheet. The Mother/Lone Father was also asked to indicate his/her willingness or otherwise to be contacted in the future about possible participation in the qualitative or nested studies.

9.3.2 HEIGHT AND WEIGHT OF MAIN RESPONDENTS

In each household, the interviewer took the weight and height measurements of the adult respondents and the Study Child(ren) in the household. Height and weight are necessary to derive a BMI score (Body Mass Index). A Leicester portable height measure was used to record height. The Leicester measure gives height in imperial and metric units, but the interviewer recorded height to the nearest millimetre. It has a range of 0 – 2.07 m. A SECA 761 flat mechanical scales was used for recording weight. They are a Class IIII medically approved scales. The scales give weight on the metric scale only and have a capacity of 150kg with 1kg graduations. Interviewers recorded weight to the nearest kilogram. Height and weight readings were recorded on the interviewer's Work Assignment Sheet for each household.

9.3.3 GPS CO-ORDINATES

The interviewer recorded the GPS co-ordinates of each household on the Work Assignment Sheet. GPS co-ordinates were recorded using a Garmin eTrex handheld GPS receiver. The receiver has 12 differential-ready parallel channels with a GPS accuracy of <15metres RMS. Latitude and longitude co-ordinates were recorded with the device by the interviewer and then converted by the Study Team to ITM (Irish Transverse Mercator/IRENET95) co-ordinates, in order to facilitate mapping using Grid In Quest software available from Ordnance Survey Ireland.

9.3.4 WORK ASSIGNMENT SHEET

A Work Assignment Sheet was issued to the interviewer for each household. It provided the interviewer with contact details for the family and was used to record response outcomes for each instrument, as well as height, weight, GPS and contact details for non-resident parents and regular carer (see Appendix N for a sample).

9.3.5 INTERVIEWER OBSERVATIONS

Interviewers recorded some basic observations about the interviews with each adult respondent in the household, such as how often the respondent asked for clarification and level of engagement with the survey. In other longitudinal cohort studies these details have been found to be highly correlated, inter alia, with the incidence of subsequent attrition, for example the Millennium Cohort Study in Britain. Interviewers were instructed to complete these observations as soon as was feasible after leaving the household. The complete list of observations is as follows

- Respondent sought clarification
- Respondent was engaged with survey
- Respondent was reluctant to answer questions
- Respondent appeared to answer questions to best of his/her knowledge
- Respondent appeared to understand the questions
- Anyone else present at interview.

Chapter 10

SCALES AND OTHER STANDARDISED MEASURES

CHAPTER 10: SCALES AND OTHER STANDARDISED MEASURES

In this chapter we discuss the scales and other standardised measures that have been used in ***Growing Up in Ireland***. Firstly, we discuss briefly why it is sometimes preferable to use scales rather than single questions, along with some of the concepts important in the development of scales, namely reliability and validity. We then describe the scales and standardised measures used in the schools before moving on to describe those used as part of the home interviews.

10.1 SCALES

Some survey questions are designed to address a bigger phenomenon than could be achieved by a single question. One such example in the current study is 'child behaviour', in which the Strengths and Difficulties Questionnaire (Goodman, 1997) is used to investigate different aspects or dimensions of child behaviour. These include emotional symptoms, conduct problems, hyperactivity/inattention, peer relationship problems, and prosocial behaviour. By asking just one or two questions, it would be difficult to capture the complexity of these constructs, and it is often deemed necessary that an assessment of these is most appropriately done through the use of a scale – simply, a group of questions that, put together, measures a particular concept or concept. Single-item measures are often not as reliable as multiple-item scales because the latter can average out measurement error in a construct when summed to obtain a total score; the measurement error that often occurs in single items is typically not assessed. Furthermore, a scale with multiple items will generally be better able to differentiate degrees of an attribute than will one single item. Therefore, choosing a scale for a survey instrument is an important decision that will shape the information collected. While the concepts of validity and reliability also apply to single items, they are described here as an introduction to the multi-item standardised measures used in ***Growing Up in Ireland***.

Scales in ***Growing Up in Ireland*** were chosen with regard to their appropriateness to the objectives of the study, and also with regard to previous findings of reliability and validity. Reliability and validity are essential for a good psychological measure, and it is to a brief consideration of these concepts that we turn first before the scales themselves are discussed in detail.

10.1.1 RELIABILITY

Reliability is concerned with the extent to which a scale should consistently reflect the construct it is measuring. In practice, this means that an individual should have similar scores at two different time points or that two people who are at a similar level of a construct should receive similar scores (Field, 2005). Scores should be relatively free of measurement error rather than true variance in the psychological construct being assessed. The reliability of a test is considered one of its most basic psychometric properties, and is necessary before assessment of the validity can even begin, although its existence does not guarantee validity.

10.1.1.1 INTERNAL CONSISTENCY

Internal consistency is based on the correlations between different items on the same test (or subscale), i.e. the *extent* to which the items in the tests or subscales assess the same characteristic, skill or quality. In internal consistency reliability estimation a single measurement instrument is administered to a group of people on one occasion to estimate reliability. This type of reliability can enable researchers to interpret data and predict the value of scores and the limits of the relationship among variables. The primary indexes of internal consistency are coefficient alpha (Cronbach, 1988), or, if the items are dichotomous, Kuder-Richardson Formula 20 (KR-20; Kuder & Richardson, 1937). A rule of thumb is that a correlation coefficient of 0.6-0.7 indicates acceptable reliability, and 0.8 or higher indicates good reliability. Extremely high reliabilities (say 0.95 or higher) are not necessarily desirable as these items may not just be consistent but actually redundant.

10.1.1.2 TEST-RETEST RELIABILITY

Test-retest reliability of an instrument is estimated by performing the same test with the same respondents at different points in time. The closer the results, the greater the test-retest reliability of the instrument. The correlation coefficient between two such sets of responses is often used as a quantitative measure of the test-retest reliability.

10.1.1.3 SPLIT HALF RELIABILITY

Split half reliability refers to a design in which a test is split in two and the scores for each half of the test is compared with the other half. If the results are consistent then it is more likely that the same thing is being measured.

10.1.2 VALIDITY

Validity refers to the degree to which a measuring instrument accurately reflects or assesses the specific concept that the researcher is attempting to measure. While reliability is concerned with the accuracy of the actual measuring instrument or procedure, validity is concerned with the study's success at measuring what the researchers set out to measure.

Researchers should be concerned with both *external* and *internal* validity. External validity refers to the extent to which the results of a study (regardless of whether it is descriptive or experimental) are generalisable or transferable. Internal validity is the extent to which account is taken of alternative explanations for any causal relationships explored, and the methodological rigour with which the study is carried out. Internal validity is only relevant to the specific study in question and the results of the study are therefore non-generalisable. Note that where validity coefficients are calculated, they will range between 0 (low) and 1 (high).

10.1.2.1 CONTENT VALIDITY

Content validity is based on the extent to which a measurement reflects the specific intended domain of content. For sociocultural studies, content validity forces the researchers to define the domains they are attempting to study.

10.1.2.2 CONSTRUCT VALIDITY

Construct validity seeks agreement between a theoretical concept and a specific measuring device or procedure. It can be broken down into two sub-categories: *Convergent validity* and *discriminate(or discriminant) validity*. Convergent validity is the actual general agreement between the instrument of interest and other instruments that purport to measure the same construct or concept, gathered independently of one another, where measures should be theoretically related. Discriminate validity is the lack of a relationship among measures that theoretically should not be related. For example, a new measure of anxiety should show similar results to another existing measure of anxiety but not to a measure of depression. In clinical settings, the term discriminate validity is sometimes used to describe the ability of an instrument to discriminate between groups, for example clinical and normal samples.

10.1.2.3 PREDICTIVE VALIDITY

Predictive validity refers to the level of agreement between the instrument of interest and some other more direct assessment of the construct, usually at some future point. It is the ability of the instrument to predict something it should, theoretically, be able to predict. For example, academic tests may be used to predict the ability of a potential student to complete a course in a given discipline.

In the remainder of the chapter we consider some of the characteristics of the scaled items used in ***Growing Up in Ireland***.

10.2 MEASURES USED IN THE SCHOOL

In this section we describe the scales used in the school in terms of description and rationale, administration and technical properties.

10.2.1 PIERS-HARRIS CHILDREN'S SELF-CONCEPT SCALE, 2ND EDITION (PIERS-HARRIS 2)

Description and Rationale

The Piers-Harris Children's Self-Concept Scale 2nd Edition (hereafter referred to as Piers Harris 2) is a revision of the Piers-Harris Children's Self-Concept Scale (Piers, 1963) and is a 60-item self-report instrument for the assessment of self-concept in children and adolescents between the ages of 7 and 18 (who have at least second grade reading ability). The authors define self-concept as a relatively stable set of attitudes reflecting both the description and evaluation of one's own behaviour and attitudes. The majority of children completed the Piers-Harris 2 in a group setting within the school (when they were completing the academic assessments). In some cases, however, the school was reluctant to have the Piers-Harris 2 administered in the school setting on two grounds: firstly, some principals were concerned about response load on the children, coming as it did after the academic assessments; secondly, some principals were concerned about the content and nature of some of the items, and how they might be misinterpreted out of context when subsequently discussed in the home with parents/guardians. Therefore some children completed the Piers-Harris 2 in the home, under similar conditions to the school administration in that they self-completed a paper booklet with no time limit for completion.

The items in the Piers-Harris 2 are statements that express how people feel about themselves, each with a yes/no answer option. The domain scales include: Behavioural Adjustment – a subscale of 14 items measuring admission or denial of problematic behaviours; Intellectual and School Status – a subscale of 16 items reflecting the Study Child's assessment of his/her abilities with respect to intellectual and academic tasks; general satisfaction with school and perceptions of future achievements; Physical Appearance and Attributes – a subscale of 11 items about perceptions of physical appearance and other attributes such as leadership and ability to express ideas; Freedom From Anxiety – a subscale of 14 items exploring a variety of feelings including fear, unhappiness, nervousness, shyness and feeling left out of things; Popularity – a subscale of 12 items exploring the Study Child's evaluation of his or her social functioning; and Happiness and Satisfaction – a subscale of 10 items reflecting feelings of happiness and satisfaction with life. The scales are scored so that a higher score indicates a more positive self-evaluation in the domain being measured. An Inconsistent Responding and a Response Bias index are also included to identify random response patterns and tendencies to respond in a certain manner irrespective of item content, such as a positive response bias.

The Piers-Harris 2 was chosen for use in the Growing Up in Ireland because it is relatively short and easy to administer, making it appropriate for use in the current research setting and thus providing an efficient quantitative assessment of children's reported self-concept. This will be important in enabling researchers to monitor children's self-concept over time as well as facilitating an exploration of the

relationship between self-concept and other factors. For example, Marsh and Craven (2006) claim that self-concept is an important mediating variable that causally impacts on a variety of desirable outcomes including academic achievement. The Piers-Harris 2 booklet is contained in Appendix Q.

Table 10.1: Summary of technical information for the Piers-Harris 2

**Title**<br>Piers-Harris Children's Self-Concept Scale, 2nd Edition
**Authors:**<br>Piers, Ellen, V., Harris, D.B., & Herzberg, D. S. (2002)
**Concept Measured:**<br>Children's Self-Concept
**Country of Origin:**<br>USA
**Respondents:**<br>Study children
**Administration in *Growing Up in Ireland*:**<br>The 60 items were self-completed on paper, mostly in the school at the same time as the reading and maths assessments, although some children completed it in the home
**Technical Info:**<br>***Source***: Piers, E. V., & Herzberg, D. S. (2007)<br>***Sample 1:*** 1,387 children aged 7-18<br>***Reliability:*** Figures for the reliability coefficients demonstrate good internal consistency with a Cronbach's alpha of .91 for the Total scale, .81 (Behavioural Adjustment), .81 (Intellectual and School Status), .75 (Physical Appearance and Attributes), .81 (Freedom From Anxiety), .74 (Popularity), and .77 (Happiness and Satisfaction).<br>***Construct validity:*** Most of the scales exhibit correlations with others in the moderate to high moderate range.<br><br>***Sample 2:*** 414 children aged 9-18 completed the Attitude Toward Guns and Violence Questionnaire (AGVQ) – a measure of attitudes concerning guns, physical aggression and interpersonal conflict.<br>***Convergent validity:*** Four of the six domain scale scores show significant negative correlations with the AGVQ Total score, holding most strongly for the Behavioural Adjustment subscale (-.46), which contains items that tap aggressive attitudes. The domain scale scores also show negative associations with one of the AGVQ subscales (Aggressive Response to Shame), which measures components of anger related to self-concept.<br><br>***Sample 3:*** 294 children aged 9-18 completed the Aggression Questionnaire (AQ) – measure of aggressive attitudes and angry beliefs.<br>***Convergent validity:*** Negative correlations are found between the AQ Total score and the Piers-Harris 2 Total score and three of its domain scales, holding most strongly for the Behavioural Adjustment subscale (-.32).

10.2.2 STRENGTHS AND DIFFICULTIES QUESTIONNAIRE (SDQ)

The SDQ was administered independently to both teachers and the Mother/Lone Father, but the detailed description is given here as it was administered to teachers as part of the school contact first.

Description and Rationale

The SDQ is a brief (25 item) behavioural screening questionnaire designed to assess emotional health and problem behaviours that can be completed by the parents or teachers of children aged 3-16 years. There is also a self-rated version for 11-16 year-olds which was not appropriate for our cohort at this

stage but may be considered for the second wave at age 13. The instrument produces scores for each of five subscales: *Emotional symptoms*, *Conduct problems*, *Hyperactivity/inattention*, *Peer relationship problems* and *Prosocial behaviour*. Each subscale comprises five items and a *Total Difficulties* score is obtained by summing scores across the four deficit-focused scales (i.e. all except the prosocial behaviour scale). Respondents are required to indicate their level of agreement with each item on a three-point scale of 'Certainly true', 'Somewhat true' or 'Not true'. Item scores vary from 0-2 depending on the type of endorsement, and the total difficulties score ranges from 0-40. Administration time is approximately five minutes.

The SDQ will provide an outcome measure of psychological adjustment across behavioural and psychosocial domains. In addition to having good psychometric properties it has the obvious advantage of being substantially shorter than comparable instruments (e.g. the Child Behaviour Checklist), and given its age profile, it can be used with our cohort at the second sweep. The SDQ has also been employed in previous large-scale longitudinal research programmes such as the Millennium Cohort Study and Growing Up in Australia, so its use in the present context will facilitate international comparisons.

Table 10.2: Summary of technical information for the SDQ

**Title:**<br>Strengths and Difficulties Questionnaire (Informant Version)
**Authors:**<br>Goodman, R. (1997) – See web site – www.sdqinfo.com
**Concept Measured:**<br>Emotional health and behavioural difficulties
**Country of Origin:**<br>United Kingdom
**Respondents:**<br>Mother/Lone Father of the Study Child<br>Teacher of the Study Child
**Administration in *Growing Up in Ireland*:**<br>Mother/Lone Father completed the SDQ as part of the Computer Assisted Personal Interview in the home. Teachers completed the SDQ on a self-completion basis as part of the Teacher-on-Pupil paper questionnaire.
**Technical Information:**<br>Validity<br>*Concurrent validity* – The SDQ has been shown to correlate highly with both the Rutter scales (Goodman, 1997) and the Child Behaviour Checklist (Goodman & Scott, 1999; Klasen et al., 2000; Koskelainen et al, 2001; Becker et al., 2004).<br><br>*Construct Validity* - The five-factor structure of the SDQ has been affirmed in a number of independent studies in Europe (Muris, Meesters & van den Berg, 2003; Smedje, Broman, Hetta, and von Knorring, 2004), in Australia (Hawes & Dadds, 2004), and the UK (Goodman, 2001). However, Dickey and Blumberg in their analysis of US NHIS data (2004) found that a three-factor solution provided the best fit to the data. Further evidence for the construct validity of the SDQ has been adduced by Hawes and Dadds (2004). In addition to showing significant cross-scale concordance, they reported that the strength and directions of these associations were conceptually meaningful. Thus, they found that the conduct problems and hyperactivity subscales were most heavily related to each other (r = 0.52), while the prosocial scale showed the expected inverse association with conduct problems (r = -0.46).<br><br>*Discriminant Validity* – As a screening tool for assessing emotional health and problem behaviour in children, the SDQ has been found to differentiate well between clinical and community based samples (Goodman, 1997; Goodman & Scott, 1999; Klasen et al., 2000), and to be sensitive to changes in behaviour following intervention (Mathai, Anderson & Bourne, 2003). Goodman et al.

(2000) showed that a total difficulties score at or above the 90th percentile predicted a 15-fold increase in the likelihood of any independently diagnosed psychiatric disorder.

Reliability
Internal consistency reliability – Goodman (2001) evaluated the internal scale reliability of the SDQ in a sample of 10,438 British children aged 5-15 years and reported coefficient alphas ranging from moderate (Peer problems – 0.59) to strong (Total difficulties – 0.82) for the parental informant version of the instrument. The mean alpha across all scales and all informants (parent, teacher and self-report) was good at 0.73.

Test-retest reliability – Hawes & Dadds (2004) examined the stability of SDQ scores over a 12-month period and found that the correlations between scores at time 1 and time 2 were remarkably stable. Test-retest reliabilities for the various scales were as follows: hyperactivity, r = 0.77; conduct problems, r = 0.65; emotional symptoms r = 0.71; peer problems, r = 0.61; prosocial, r = 0.64; total difficulties, r = 0.77.

10.3 DRUMCONDRA MATHS AND READING TESTS

Rationale

The Drumcondra Maths and Reading Tests are developed for Irish school children and are linked to the national curriculum. The versions used for ***Growing Up in Ireland*** were revised for use from 2007. They would not have been used, or seen, by the schools prior to their use in ***Growing Up in Ireland***. Only one part of each test was used in order to reduce the burden on schools participating in the Study (following the advice of the test developers) and should provide a sufficient indicator of ability for research purposes. The tests are grade-specific and are strongly linked to the syllabus for each year. Nine-year-old children are distributed across three year groups in national school system (2nd, 3rd and 4th Classes). Accordingly, Levels 2, 3 and 4 of the Drumcondra Maths and Reading tests were administered in the schools. The majority of children were in 3rd class and so completed Level 3 tests in Maths and Reading. Level 4 followed the same procedures as for Level 3, but there were some differences for Level 2 in both tests. Prior to analysis, scores are adjusted according to class level and child's age at administration so that they are comparable across the different levels. As the majority of children completed Level 3, this level of the test is described in detail, with supplementary information on Levels 2 and 4 where these are different. One per cent of all children did not complete the Drumcondra tests due to special needs or having insufficient English (according to their teacher) to complete the test. The wishes of parents/guardians, principals or teachers who requested that a child should not sit the test were, of course, respected in all cases.

The cover sheet only for the Drumcondra Level 3 Reading and Maths tests are provided in Appendices O and P respectively.

10.3.1 DRUMCONDRA PRIMARY MATHS TEST – REVISED – DPMT-R LEVEL 3

Description

The DPMT-R Level 3 assesses the skills and content strands laid out in the national curriculum for Third Class. Part A of Form A assesses the following (number of items in brackets, each item measured one content strand and one skill):

Content Strands	Skills
Number (21)	Applying & Problem Solving (5)
Algebra (1)	Reasoning (10)
Data (3)	Implementing (8)
	Understanding & Recalling (2)

Part A consists of 25 questions in a booklet. Each Study Child was awarded one mark for each correct answer giving a raw score range of 0 – 25. Questions were framed in a mixture of short-answer and multiple-choice formats. For example:

Short-answer:

It takes Paula half an hour to paint 10m of fence. How many metres does she paint in 2½ hours?

[ ] (correct answer written into box)

Multiple-choice:

Mark the number that goes in the box. 50 x 20 = [ ]

A. 100
B. 1000
C. 10000
D. 50000 (correct answer indicated by circling relevant letter)

Children were given 35 minutes to complete all 25 questions. The administrator did not read out the questions. An Irish language version of the maths test was available to schools that requested it.

Table 10.4 Summary of technical information for DPMT-R Level 3

**Title:**<br>Drumcondra Primary Mathematics Test – Revised Level 3 (Form A, Part A)
**Authors:**<br>Educational Research Centre, St Patrick's College (2006)
**Concept Measured:**<br>Achievement in mathematics
**Country of Origin:**<br>Ireland
**Respondents:**<br>Study Children
**Administration in *Growing Up in Ireland*:**<br>Children completed the tests in a group setting within the school.
**Technical Information:**<br>*Sample size:* 1968 Third Class pupils in 70 schools for Form A and Form B<br>989 pupils completed Form A.<br>*Reliability:* Internal consistency of KR_{20} = 0.929<br>*Validity:* Construct validity was checked via significant correlation between strands and with the total score.
**Notes:**<br>Technical information above relates to the complete test for Form A: Parts A, B and C. ***Growing Up in Ireland*** used only Part A

10.3.2 DIFFERENCES IN REVISED LEVEL 2 MATHS TEST (DPMT-R) COMPARED TO LEVEL 3

Description
The DPMT-R Level 2 assesses the skills and content strands laid out in the national curriculum for Second Class. Part A of Form A assesses the following (number of items in brackets, each item measured one content strand and one skill):

Content Strands	Skills
Number (23)	Applying & Problem Solving (8)
Algebra (2)	Integrating & Connecting (4)
Measures (5)	Reasoning (12)
	Implementing (5)
	Understanding & Recalling (1)

Part A consists of 30 questions in a booklet, giving a raw score range of 0 – 30.

The test administrator (the interviewer) read out each question, pausing to allow children to write in their answers on the booklet (all questions also appeared in print in the booklet). There was no proscribed time limit within which children answered the questions, but a guideline time of 30 minutes was suggested to interviewers. This is in line with the test protocols set down by the Educational Research Centre, Drumcondra, Dublin who developed the tests.

Technical information
Sample size: 2133 Second Class pupils in 72 schools
Reliability: Internal consistency of KR_{20} = 0.932 (Spring)
Validity: Construct validity was checked via significant correlation among strands and with the total score.
NB: Technical information above relates to the complete test: Part A and Part B.

10.3.3 DIFFERENCES IN REVISED LEVEL 4 MATHS TEST (DPMT-R) COMPARED TO LEVEL 3

Description
The DPMT-R Level 4 assesses the skills and content strands laid out in the national curriculum for Fourth Class. Part A of Form A assesses the following (number of items in brackets, each item measured one content strand and one skill):

Content Strands	Skills
Number (19)	Applying & Problem Solving (4)
Algebra (3)	Reasoning (8)
Data (3)	Implementing (5)
	Understanding & Recalling (4)
	Integrating & Connecting (4)

Technical information
Sample size: 1920 Fourth Class pupils in 74 schools for Form A and Form B
961 pupils completed Form A.
Reliability: Internal consistency of KR20 = 0.948
Validity: Construct validity was checked via significant correlation among strands and with the total score
NB: Technical information above relates to the complete test for Form A: Parts A, B and C. Growing Up in Ireland used only Part A.

10.4.1 DRUMCONDRA PRIMARY READING TEST – REVISED – DPMT-R LEVEL 3

Description

The DPRT-R Level 3 assesses the reading skills expected for Third Class pupils. The total test comprises Vocabulary and Comprehension. For the purposes of ***Growing Up in Ireland***, however, only the Vocabulary part of the test was administered.

Form A of the Vocabulary Test consists of a booklet of 40 questions. Each Study Child was awarded one mark for each correct answer giving a raw score range of 0 – 40. Questions were in the form of a short sentence with a word underlined. Children were asked to select, from a choice of four, the words closest in meaning for the underlined words. For example:

(Which word is closest in meaning to the underlined word?)

They had an anxious wait.

A. a lengthy
B. an uneasy
C. an unusual
D. a relaxed

Having completed the sample questions with the children, the test administrator (the interviewer) allowed them 20 minutes to complete all the questions. The questions were not read aloud. Children filled in their answers on a separate, computer-readable answer sheet. This involved filling in a box corresponding to the letter beside the correct answer for the appropriate question number. The reading test was always in English, including in Gaelscoileanna (Irish speaking schools)[13].

Table 10.7: Summary of technical information DPRT-R Level 3

**Title:**<br>Drumcondra Primary Reading Test – Revised Level 3 (Form A, Vocabulary)
**Authors:**<br>Educational Research Centre, St Patrick's College (2007)
**Concept Measured:**<br>Achievement in reading (vocabulary)
**Country of Origin:**<br>Ireland
**Respondents:**<br>Study Children
**Administration in *Growing Up in Ireland*:**<br>Children completed the tests in a group setting within the school.
**Technical Information:**<br>*Sample size:* 2275 Third Class pupils in 84 schools (Spring)<br>*Reliability:* Internal consistency of KR_{20} = 0.93, (n=1141) for Spring administration of Vocabulary Test<br>*Validity:* Construct validity was checked via significant correlation among strands and with the total score. Correlation between Vocabulary and Total Score was 0.96. Factor analysis of the complete test identified five factors. Two of these factors appeared to reflect vocabulary, one dealing with easier items and the other more difficult items. A third factor comprised only comprehension items. The remaining two unidentified factors were a mixture of vocabulary and comprehension item

[13] The test is an English reading test which, by definition, cannot be translated into Irish.

loadings.

10.4.2 DIFFERENCES IN REVISED LEVEL 2 READING TEST (DPMT-R) COMPARED TO LEVEL 3

Description
The DPRT-R Level 2 assesses the reading skills expected for Second Class pupils.

Form A of the Vocabulary test consists of 36 questions in a booklet, giving a raw score range of 0 – 36. Children marked their answers directly into the booklet (not a separate sheet).

Technical information
Sample size: 2184 Second Class pupils in 82 schools (Spring)
Reliability: Internal consistency of KR20 = 0.92, (n=1088) for Spring administration of Vocabulary
Validity: Construct validity was checked via significant correlation among strands and with the total score. Correlation between Vocabulary and Total Score was 0.96. A factor analysis of the complete test identified five factors, one each apparently reflecting vocabulary and comprehension as distinct factors. However, the remaining three factors are unidentified and have loadings from a mixture of vocabulary and comprehension items.

10.4.3 DIFFERENCES IN REVISED LEVEL 4 READING TEST (DPMT-R) COMPARED TO LEVEL 3

Description
The DPRT-R Level 4 assesses the reading skills expected for Fourth Class pupils.

Technical Information:
Sample size: 2164 Fourth Class pupils in 84 schools (Spring)
Reliability: Internal consistency of KR_{20} = 0.92, (n=1083) for Spring administration of Vocabulary
Validity: Is not separately reported for Level 4, but factor analysis is said to be similar to that reported for Level 3 (see Table 10.7)

10.4 MEASURES USED IN THE HOME

10.4.1 BASIC DEPRIVATION SCALE

Description and Rationale
A substantial amount of research into poverty and deprivation, as well as their influence on outcomes across a very wide range of substantive research areas, has been undertaken in Ireland in recent years (for an overview see, for example, Maitre et al., 2006). Fundamental to much of this work has been the development and implementation of a Basic Deprivation scale. This measure has been developed by the Economic and Social Research Institute (ESRI) and has been used to assess the incidence, correlates and drivers of poverty and deprivation both in Ireland and, increasingly, internationally. The Basic Deprivation Scale has been extremely important in framing Ireland's National Anti-Poverty Strategy, as well as in monitoring progress towards achieving national targets.

The scale has been developed through work going back to 1987 (see Callan et al., 1993, Layte et al., 2001, Nolan et al., 2002 and Maitre et al., 2006). It has been revised most recently using data collected by the Central Statistics Office in 2003 as part of the EU-harmonised European Union-Survey of Income and Living Conditions (EU-SILC).

The Basic Deprivation scale is made up of 11 items relating to poverty in areas such as food, clothing, furniture, debt and minimal participation in social life. The index can be used on its own as a measure of non-monetary deprivation. It has also been very widely combined with thresholds of relative income

poverty to provide a measure of 'consistent' poverty status and changes therein over time. Using it in this way helps form a comprehensive picture of the household's command of resources – financial and otherwise.

The Basic Deprivation scale is one of four identified in analysis of the CSO's EU-SILC data. The other three subscales relate to Secondary Deprivation, Housing Deprivation, and Neighbourhood/Environmental deprivation. The dimensionsality of deprivation were investigated using exploratory factor analysis on an initial set of 39 items from the EU-SILC survey. Item loadings on the basic deprivation dimension ranged from 0.55 for going without heating to 0.71 for being able to afford new clothes and eating a roast joint or equivalent (Whelan, Maitre & Nolan, 2007).

Given the focus of the ***Growing Up in Ireland*** project and space constraints in the relevant instruments, we included only the items associated with Basic Deprivation. Experience in administering the items included in the Basic Deprivation scale has shown that the set of items in question are relatively non-threatening for the respondent and are relatively short and easily measured, making them appropriate for use in the current research setting.

Table 10.9: Summary of technical information for the Basic Deprivation Scale

**Title:**<br>Basic Deprivation Scale
**Authors:**<br>Economic and Social Research Institute, Dublin (for development and history see, for example, Maitre et al., 2006)
**Concept Measured:**<br>Basic deprivation and component of consistent poverty
**Country of Origin:**<br>Ireland
**Respondents:**<br>National samples of households and adults therein
**Administration in *Growing Up in Ireland*:**<br>The 11 items were recorded on the Mother/Lone Father CAPI instrument.
**Technical Information:**<br>Developmental work was carried out on a national survey of private households in a 1987 survey on Lifestyle and Usage of State Services that included c. 4,000 households and related adults. Similarly, annual European Community Household Panel Surveys between 1994 and 2001 used varying sample sizes ranging from c. 4,000 to 2,500 households. Most recently, a national survey of 3,112 private households and related adults was conducted in the EU-SILC survey. This was carried out on behalf of Eurostat by the Irish Central Statistics Office. The 11-item Basic Scale included in ***Growing Up*** is based on this data source.<br><br>***Reliability:*** Very good internal consistency with an alpha Cronbach of 0.84<br><br>***Validity:*** Construct validity strong. The scale exhibits high correlations with others in this area including the ECHP 8-item Basic Deprivation index.

10.4.2 CENTRE FOR EPIDEMIOLOGICAL STUDIES DEPRESSION SCALE (8-ITEM) (CESD-8)

Description and Rationale

The CES-D is a widely used self-report measure that was developed specifically as a screening instrument for depression in the general population as opposed to being a diagnostic tool that measures the presence of clinical depression. It was originally designed as a dimensional assessment of

depression in adults and has also been used to screen for depression in children and adolescents. The CES-D has been shown to discriminate children with depressive disorders from those without psychopathology (e.g. Prescott, McArdle, Hishinuma et al., 1998) and to discriminate depressive disorders from other forms of psychopathology (e.g., Roberts, Andrews, Lewinsohn & Hops, 1990), as well as correlating highly with other measures of depression, thereby supporting its validity.

The ***Growing Up in Ireland*** used the short (8 item) version of the CES-D, which correlates highly with the full 20-item version (r = 0.93). Sample items include, ‘I felt that I could not shake off the blues even with help from my family and friends’, and ‘I thought my life had been a failure’, which were answered on a 4-point Likert-scale ranging from 0 (<1 day) to 3 (5–7 days), with reference to the previous 7-day period. A composite score is calculated by summing item responses (range: 0–24). Respondents are categorised according to the recommended criterion for depression, with composite scores of ≥7 being classified as depressed and scores <7 defined as not depressed. It should be noted however that while a score above or equal to 7 suggests a clinically significant level of psychological distress, it does not necessarily mean that the participant has a clinical diagnosis of depression. In a general population, about 20% would be expected to score in this range. It is incorporated into the ***Growing Up in Ireland*** on a self-complete basis because of its sensitivity and to minimise report bias.

Although several studies have reported only a modest relationship between the CES-D and a diagnosis of depression from a structured clinical interview, it is still likely that there will be important psychological differences between those scoring above and below the cut-off points on the scale.

The 8-item CES-D has the advantage of being a short measure (administered in 2-3 minutes) that has been used in a large number of studies. Short depressive symptom indices such as this are generally regarded as acceptable in those instances where a brief assessment is needed for broad screening or research purposes, although there is also a conversion formula for projecting the full 20-item CES-D from the 8-item version in order to compare results. Access to information on experience of depression is particularly important in light of research showing that not only is depression a prevalent condition but that depression in a parent can also impact on child outcomes (e.g. Beardslee, Keller, Seifer et al., 1996).

Table 10.10: Summary of technical information for the CES-D

**Title:**<br>Center for Epidemiological Studies Depression Scale (8 items)
**Authors:**<br>Melchior, L.A., Huba, G.J., Brown, B. & Reback, C.J. (1993)<br>Derived from the Center for Epidemiological Studies Depression Scale (20 items) - NIMH
**Concept Measured:**<br>Depression
**Country of Origin:**<br>USA
**Respondents:**<br>Administered to Mother/Lone Father and Father/Partner on paper as sensitive part of main interview.
**Administration in *Growing Up in Ireland*:**<br>Self-completed on paper during the sensitive part of main interview.
**Technical Information:**<br>***Source:*** Melchior, Huba, Brown & Reback (1993)<br>***Sample 1:*** heterogeneous community sample of 411 women<br>***Reliability:*** scale shows high internal consistency .86<br>***Validity:*** The scale correlates highly (.93) with the original 20-item version CES-D scale.<br><br>***Sample 2:*** 83 women in a residential drug abuse program<br>***Concurrent validity:*** The scale correlates with the BPI depression scale (.54).

Source: DiClemente et al. (2005)
Sample: 460 black female adolescents
Reliability: Test-retest reliability of .83 and .87 respectively was found for the 6- and 12-month follow-up assessments.

Source: Huba, Melchior, Panter (1998-2001)
Sample: 683 clients with HIV/AIDS
Reliability: Internal consistency reliability was 0.88.

10.4.3 THE SEVEN-ITEM SHORT FORM OF THE DYADIC ADJUSTMENT SCALE (DAS-7)

Description and Rationale

The original version of the DAS had 32 items and was developed by Spanier (1976). It provides an assessment of dyadic satisfaction based on participants' self-report and is used as a means of categorising marriages as either distressed or adjusted. It has also been shown to discriminate between couples in the community and those seeking marital therapy services. Findings from several studies provide strong evidence that the shorter DAS has maintained the content coverage of the original DAS while maintaining strong levels of reliability and validity.

The ***Growing Up in Ireland*** used the 7-item DAS (Sharpley & Rogers, 1984) which comprises three subscales and seven questions: three items assessing *dyadic consensus*, where participants rate the degree to which they agree with their partner on several issues including 'Philosophy of life' and 'Amount of time spent together'; three items assessing *dyadic cohesion* where participants indicate how often specific dyadic activities occur, such as 'Have a stimulating exchange of ideas' and 'Calmly discuss something together'; and one item assessing *global marital satisfaction* where participants rated their general satisfaction with their 'real life' relationship. Six of the items are rated on a six-point Likert-type scale (with endpoints *always agree* and *always disagree* or *all the time* and *never*), while the seventh item is rated on a seven-point scale ranging from *extremely unhappy* to *perfect*. A general satisfaction score is calculated as a sum of all seven items' scores.

Marital satisfaction is an important factor in family functioning and the manner in which parents interact is crucial for child outcomes. For example, marital satisfaction has been highlighted as not only important in impacting the child's wellbeing, but also on that of the parents, as it is seen as a component of adult life satisfaction (Bradbury, Fincham, & Beach, 2000). While the researchers are aware that reliance solely on the DAS (7-item) to determine marital distress might result in some classification errors, the brevity of the measure and its reliability and validity make it an ideal tool for the research purposes of a project such as the ***Growing Up in Ireland***.

Table 10.11: Summary of technical information for the DAS-7

**Title:**<br>Seven-item Short Form of the Dyadic Adjustment Scale (DAS-7)
**Authors:**<br>Sharpley, C.F. & Rogers, H.J. (1984)<br>Derived from the original Dyadic Adjustment Scale, Spanier (1976)
**Concept Measured:**<br>Marital Satisfaction
**Country of Origin:**<br>USA/Australia
**Respondents:**<br>Mother/Lone Father and Father/Partner

Administration in *Growing Up in Ireland*:
Self-completed on paper during sensitive part of main interview

Technical Information:
Source: Sharpley & Rogers (1984)
Sample: 545 married, separated and divorced individuals
Reliability: Scale shows acceptable internal consistency of .76 for an abbreviated screening test.
Discriminant validity: Scale differentiated between married, separated and divorced couples.

Source: Hunsley, Pinsent, Lefebvre, James-Tanner & Vito (1995)
Sample: 196 cohabiting or married individuals
Reliability: The scale yielded a coefficient alpha of .82.
Concurrent validity: Moderate to high correlations of .46 and .72 were found with the Emotional Self-disclosure Scale and the Kansas Marital Satisfaction Scale.

Source: Hunsley, Best, Levebvre & Vito (2001)
Sample 1: 392 individuals – 148 from a clinical sample, 244 from a community sample
Reliability: An internal consistency measure of .79 was yielded for both the clinical and community samples.
Discriminant validity: Criterion validity was evidenced as the scale proved effective in distinguishing couples in the community sample from those seeking marital therapy services. The measure was also successful in classifying participant marriages as distressed or adjusted.

Sample 2: 162 cohabiting or married individuals
Reliability: The internal consistency reliability was .78 for this sample.
Concurrent validity: Correlations of .69 and .43 were found with the Kansas Marital Satisfaction Scale and the Emotional Self-disclosure Scale.

10.4.4 EMOTIONALITY, ACTIVITY AND SOCIABILITY TEMPERAMENT QUESTIONNAIRE (EAS)

Description and Rationale

The EAS is a 20-item instrument designed to measure heritable aspects of temperament that are related to developmental differences in personality and behaviour. The instrument produces scores for each of four scales: *Emotionality*, *Activity Level*, *Sociability* and *Shyness*. *Emotionality* relates to negative quality of emotional style and intensity of reactions. *Activity level* refers to preferred levels of activation and speed of action. *Sociability* reflects the tendency to prefer the company of others to being alone and is associated with positive emotionality, and *Shyness* reflects the tendency to be inhibited and awkward in new social situations. Each scale comprises five items and respondents are required to indicate their level of agreement to each item on a five-point scale ranging from *Not Characteristic* to *Very Characteristic*. It is recommended for use with children aged one to nine years.

Temperament has been shown to have significant long-term effects on development and is an important predictor of later social and psychological wellbeing. Indeed, the predictive value of temperament dimensions in assessing vulnerability to behavioural and psychosocial difficulties has become an active area of research in child psychology. For example, emotionality has been identified as a risk factor for onset of major depression in adults (Kelvin, Goodyear & Altham, 1996), and individual differences in reactivity and behaviour are often associated with developmental outcomes such as psychological adjustment and resilience to stress (Gasman, Purper-Ouakil, Michel et al., 2002). Although temperament researchers continue to dispute the exact number and composition of temperament dimensions, the EAS provides a measure of the constructs (Emotionality, Activity, and Sociability) that are considered to be among the most stable and heritable temperament traits (Buss & Plomin, 1984; Rutter, 1987; Prior, 1992). It also benefits from having good psychometric properties and a relatively short administration time (approximately. 5 mins).

Table 10.12: Summary of technical information for EAS

**Title:**<br>The EAS Temperament Survey for Children: Parental Ratings
**Authors:**<br>Buss, A.H. & Plomin R. (1984)
**Concept Measured:**<br>Temperament
**Country of Origin:**<br>United Kingdom
**Respondents:**<br>Mother/Lone Father
**Administration in *Growing Up in Ireland*:**<br>Respondents completed the EAS as part of the Computer Assisted Personal Interview in the home.
**Technical Information:**<br>Validity<br>*Construct Validity* - Mathiesen & Tambs (1999) examined the four-factor structure of the EAS with a population-based sample of Norwegian children who were assessed at three different times between 18 and 50 months of age. A four-factor structure fitted the data best and the factorial structure did not appear to vary substantially with the age of the children. Examination of the factor loadings revealed that 16 of the 20 items (All five of the Activity items, four of the Emotionality items, four of the Sociability items, and three of the Shyness items) had their highest loadings on the expected factor. Boer & Westenberg (1994) in their analysis of Dutch data concluded that the emotionality, activity and shyness items were factorially robust as all 15 items loaded only on their posited factor (i.e. none of the off-diagonal factor loadings exceeded 0.30). However, they were more equivocal in their support for the sociability factor which had substantive loadings on the shyness and/or activity constructs. Similar conclusions were reached by Gasman et al. (2002) who found it difficult to decide between a three or a four-factor solution using confirmatory factor analytic techniques. The average scale inter-correlation across the three temperament dimensions of Emotionality, Activity and Shyness ranges from 0.10 (Rowe & Plomin, 1977) to 0.16 (Boer & Westenberg, 1994; Mathiesen & Tambs, 1999), which can be considered good evidence for the relative independence of the EAS temperament traits.<br><br>Reliability<br>*Internal consistency reliability* meets the standard criterion [Emotionality (0.70); Activity (0.70); Shyness (0.69); Sociability (0.77)] with mean Cronbach's alpha of 0.71 for the parental ratings version of the instrument (Gasman et al, 2002).<br><br>*Test-retest reliability* is good as evidenced by stability coefficients averaging 0.81, 0.79 and 0.68 across three time periods with children aged 18-50 months (Mathiesen & Tambs, 1999).

10.4.5 PARENTING STYLE INVENTORY – II (PSI-II)

Description and Rationale

The Parenting Style Inventory was originally designed to assess the construct of parenting style independently of parenting practice. Parenting style refers to the overall emotional climate in which particular parent-child interactions occur. Limitations of previous scales have been that they confuse parenting style with parenting practices that are directed towards particular goals, for example social or academic (Steinberg et al., 1992; Dornbusch et al., 1987).

The adapted PSI-II was used as it was short and easy for the children to read. Study children completed the *Responsiveness* and *Demandingness* subscales from the Parenting Style Inventory – II. The third subscale, *Psychological Autonomy-Granting*, was not used as it was thought to be less appropriate for 9-

year-olds than for the adolescents for which it was originally developed. As with the other questions on this supplement, the Study Child completed the scales in respect of the adults who completed the Mother/Lone Father and Father/Partner interviews in the household. They also completed one in respect of a non-resident parent if appropriate. The Mother/Lone Father may not, however, have approved the Study Child's completion of all possible relevant questionnaires. Hence each Study Child described the parenting style of some combination of adults acting in a parental role: mother, father, mother's partner, father's partner – subject to the permission of the Mother/Lone Father.

Each subscale consisted of five questions asking about the parental figure's parenting style with the Study Child, phrased 'Does your dad . . .?', to which the Study Child answered on a three-point scale of *Always*, *Sometimes* or *Never*. The questions on the *Responsiveness* subscale reflect positive, warm interactions such as discussing problems, being praised and doing things together. The questions on the *Demandingness* subscale relate to the setting of, and sticking to, family rules, and to discipline.

The Parenting Style Inventory – II subscales of *Responsiveness* and *Demandingness* were selected as these were most closely related to the concepts of warmth and control in parenting, which are the dimensions commonly used to categorise parenting styles as authoritarian, authoritative, neglectful or permissive. Parenting style is widely acknowledged as being an important input to child development and later wellbeing (see literature review in GUI Research Paper 2), particularly in relation to the positive impact of an authoritative parenting style (high warmth combined with high control). This particular child-report inventory had certain advantages: it was much shorter than most of the inventories aimed at adult respondents, which are commonly 40 items or more in length; the scale was entirely self-report requiring no direct observations of parenting behaviour; and it is in keeping with the Study's focus on affording children the opportunity to give their views and report on their own lives.

Table 10.14: Summary technical information for the CPR-S

**Title:**<br>Child-Parent Relationship Scale (CPR-S)
**Authors:**<br>Pianta, R.C. (1992)
**Concept Measured:**<br>Relationship between parent and child; subscales of conflicts, positive aspects and dependence
**Country of Origin:**<br>USA
**Respondents:**<br>Mother/Lone Father and Father/Partner
**Administration in *Growing Up in Ireland*:**<br>Respondents completed the scale as part of the Computer-Assisted Personal Interview in the home.
**Technical Information:**<br>***Sample size:*** 714 children aged 4.5 - 5.5 years old<br>***Reliability:*** Alpha levels = .83 (Conflicts), .72 (Positive aspects) and .50 (Dependence)

10.4.7 THE STRENGTHS AND DIFFICULTIES QUESTIONNAIRE (SDQ)

The SDQ was administered on a CAPI basis to the Mother/Lone Father as part of the home interview. As the questionnaire was also administered to the Study Child's teacher, it is described in detail in the section on Measures used in the School (10.2.2).

10.5 RELATIONSHIP OF SCALES AND STANDARDISED MEASURES TO CHILD OUTCOMES

Five of the scales and standardised measures used in ***Growing Up in Ireland*** measure an outcome of the Study Child (see Table 10.15). The other scales measure concepts that are thought to affect child outcomes, now or in the future, and are more accurately described as input variables. For example, the CES-D, DAS and PSI-II would be expected to affect the child's socioemotional/behavioural wellbeing outcomes. The Basic Deprivation Scale could be considered as an outcome measure in itself, but could also potentially influence all three child outcomes: physical health and development, socio-emotional/behavioural wellbeing, and educational achievement and intellectual capacity.

Table 10.15: Relationship of standardised measures to child outcomes and use in Wave 2

Measure	Section	Respondent(s)	Primary related child outcome	Procedure at Wave 2 (Age 13)a
Piers-Harris Children's Self-Concept Scale, 2nd Edition	10.2.1	Study Child	Socioemotional/behavioural wellbeing	Repeated
Strengths and Difficulties Questionnaire (SDQ)	10.2.2	Teacher and Mother/Lone Father	Socioemotional/behavioural wellbeing	Repeated
Drumcondra Maths and Reading Tests	10.3	Study Child	Educational achievement and cognitive capacity	Possibly followed by the Verbal Reasoning and Numerical Ability tests from the Differential Aptitudes Test. [a]
Basic Deprivation Scale	10.4.1	Mother/Lone Father	*Input variable/Deprivation Outcome*	Repeated
Centre for Epidemiological Studies Depression Scale (CES-D, 8-item)	10.4.2	Mother/Lone Father and Father/Partner	*Input variable*	Repeated
Seven Item Short Form of the Dyadic Adjustment Scale (DAS)	10.4.3	Mother/Lone Father and Father/Partner	*Input variable*	Repeated
Emotionality, Activity and Sociability Temperament Questionnaire (EAS)	10.4.4	Mother/Lone Father	Socio-emotional/behavioural wellbeing	While temperament will be assessed at age 13, this particular scale will not be used as it is not suitable for older children. Possibly use the SATI.[b]
Parenting Style Inventory	10.4.5	Study Child	*Input variable*	Repeated in original format

II (PSI –II)				
Pianta Child-Parent Relationship Scale	10.4.6	Mother/Lone Father and Father/Partner	Socioemotional/behavioural wellbeing	Although this measure is normally used with younger children, the Study Team hope to repeat the scale when the children are 13 years.[c]

a) The Verbal Reasoning and Numerical Ability tests from the Differential Aptitudes Test (Adapted Irish Version) can be used in combination as an index of scholastic ability according to the Educational Research Centre who publish both the Drumcondra and Differential Aptitudes Test.

b) The Australian Temperament Project used the EAS at age 9-10 and the School Aged Temperament Inventory (SATI) between ages 11-16, so this is an option Growing Up in Ireland will also consider.

c) The version used in phase 1 of Growing Up in Ireland was adapted from the original which targeted adolescents. Hence it could be repeated, perhaps with the wording originally used with adolescents and also with the third scale of 'autonomy' which was not used with the nine-year-olds.

The table also indicates whether or not the standard measures will be repeated at the second wave at age 13 years. While the Study Team values continuity in measures, a final decision on whether or not to repeat a given measure will depend on its usefulness in the nine-year-old data collection as well as the applicability of the measure for older children. Where alternatives are suggested, these alternatives are only speculative at this early stage.

Chapter 11

TRIANGULATION AND DISTRIBUTION OF VARIABLES

CHAPTER 11: TRIANGULATION AND DISTRIBUTION OF VARIABLES

In this chapter we consider two important aspects of the data discussed in the foregoing chapters. First we consider triangulation strategies before moving on to a general overview of the distribution of outcome and explanatory variables.

11.1 TRIANGULATION

Triangulation is undertaken principally with a view to data confirmation and completeness. At least four different types of triangulation can be identified in applied research

- Methodological triangulation – different research methodologies, quantitative / qualitative etc.
- Theoretical triangulation – approaching the same research question from different theoretical positions
- Investigator triangulation – different investigators or studies approaching a similar question
- Data triangulation – recording information on the same basic unit of analysis or subject from different perspectives and informants.

This last aspect of triangulation was principally used in ***Growing Up in Ireland.***

As noted in the previous chapters, data on various aspects of the Study Child were recorded by different respondents, e.g primary caregivers, secondary caregivers, teachers, the children themselves, and so on. Whilst the same questions or question wordings did not appear in every instrument, the opportunity was taken to record similar measures and concepts from different informants. There are three main reasons why data triangulation was incorporated into the study design. In some cases it was important to check that reports were consistent when assessing their accuracy, f or example, academic ability. At other times we were particularly interested in the inconsistencies, for example those evident between self-reported weight and actual weight. In the first scenario a user of the data may want to check that a very low score on the Drumcondra test is not an aberration by referencing it against the parent and teacher reports of the child's academic ability. In the second example a researcher might be interested in the implications for health and lifestyle if a child or adult thinks he/she is more or less heavy than their actual weight. A third reason for data triangulation is related to within survey response rates. Triangulation is a useful tool in multiple respondent surveys when the response rate among the 'best' informant may be lower than desirable (e.g. among teachers). Supplementary triangulated information from a high-response informant (e.g. the parent) serves two functions. First, it provides at least some information on the Study Child. Secondly, it can be used in an adjustment model to allow the analyst to account for missing information (possibly even facilitating imputation).

Triangulation obviously comes at a cost. It clearly increases respondent burden and adds to the time taken to administer the instruments. If it is not implemented with due care, planning and discretion, it may lead to a negative reaction from survey participants. If respondents construe the recording of similar information from different study participants as a test of the veracity of the information already provided this could have serious negative implications for cohort maintenance and attrition rates. Accordingly, though it is highly desirable, triangulation must be applied judiciously and reasonably within a study. This was a major constraining factor in deciding on where and when to use triangulations throughout the development and design of the study.

In Table 11.1 below we present summary details of the main areas in which triangulation was applied. This outlines the nature of the triangulated information, the sources (informants) and the questions involved at each stage of the process.

Variable	Instruments	Question No.'s
Frequency of homework	Teacher-on-Self	15
	Mother/Lone Father	J10
	Child Main	4
Time spent on homework	Teacher-on-Self	16
	Mother/Lone Father	J11
Strengths and Difficulties Questionnaire	Teacher-on-Pupil	9
	Mother/Lone Father	H2
Academic Ability	Teacher-on-Pupil<br>Mother/Lone Father<br>Child Main<br><br>Drumcondra Reading Test<br>Drumcondra Maths Test<br>Piers-Harris 2	10<br>J13, J14<br>2<br><br>Intellectual and School Status subscale items
Absenteeism (frequency)	Teacher-on-Pupil<br>Mother/Lone Father<br>Principal (school-level)	5<br>J8<br>22, 23
Absenteeism (reason)	Teacher-on-Pupil<br>Mother/Lone Father	6<br>J9
Attendance at parent-teacher meetings	Teacher-on-Pupil<br>Mother/Lone Father<br>Teacher-on-Self (class-level)<br>Principal (school-level)	11<br>J7<br>20a<br>34, 35
Limitations on child's school activities	Teacher-on-Pupil<br>Child Main	12<br>21b
Child as victim of bullying (occurrence)	Mother/Lone Father<br>Child Core Sensitive<br>Principal (school-level)	J18<br>20<br>41
Child as victim of bullying (form)	Mother/Lone Father<br>Child Core Sensitive	J19<br>21a
Child as bully	Teacher-on-pupil<br>Mother/Lone Father<br>Child Core Sensitive<br>Principal (school-level)	9l (SDQ item)<br>K14a, H2l (SDQ item)<br>18<br>41

Table 11.2: Health and family related variables

Variable	Instruments	Question No.'s
Child Disability/Medical Condition	Mother/Lone Father<br>Child Main	B11<br>21a
Child's diet in last 24 hours	Mother/Lone Father<br>Child Main	D1<br>6
Perception of appropriateness of child's weight	Mother/Lone Father<br>Child Main<br>Child's actual BMI	D9<br>22
Mother/Lone Father self-reported weight	Mother/Lone Father<br>Actual weight	F10

Mother/Lone Father self-reported height	Mother/Lone Father<br>Actual height	F9
Father/Partner self-reported weight	Father/Partner<br>Actual weight	C10
Father/Partner self-reported height	Father/Partner<br>Actual height	C9
Parental discipline practices	Mother/Lone Father<br>Child Sensitive Supplement	K3<br>4
Family Activities	Mother/Lone Father<br>Child Main	K4<br>7
Spouse/partner occupation	Mother/Lone Father<br>Father/Partner	L22e<br>E10
Relationship of father/partner to child	Mother/Lone Father<br>(household grid)<br>Father/Partner	A5<br><br>A1

Table 11.3: Non-resident Parents

**Variable**	**Instruments**	**Question No.'s**
Financial support from non-resident parent	Mother/Lone Father Sensitive Supplement<br>Non-resident Parent Questionnaire	S44<br><br>14
Custody Arrangements	Mother/Lone Father Sensitive Supplement<br>Non-resident Parent Questionnaire	S38<br><br>8
Frequency non-resident parent visits	Mother/Lone Father Sensitive Supplement<br>Non-resident Parent Questionnaire	S43<br><br>2.3
Relationship at pregnancy	Mother/Lone Father Sensitive Supplement<br>Non-resident Parent Questionnaire	S37<br><br>19
Timing of split with non-resident parent	Mother/Lone Father Sensitive Supplement<br>Non-resident Parent Questionnaire	S36<br><br>20
Talk to non-resident parent about child	Mother/Lone Father Sensitive Supplement<br>Non-resident Parent Questionnaire	S47<br><br>24
Current emotional relationship with non-resident parent	Mother/Lone Father Sensitive Supplement<br>Non-resident Parent Questionnaire	S48<br><br>25

Table 11.4: Non-parental Care

Variable	Instruments	Question No.'s
Main type of care	Mother/Lone Father<br>Home-based Carer, *or*<br>Centre-based Carer	J2<br>1.3<br>22
Hours spent in main care	Mother/Lone Father<br>Home-based Carer, *or*<br>Centre-based Carer	J3<br>5<br>2
Days per week spent in care	Mother/Lone Father<br>Home-based Carer, *or*<br>Centre-based Carer	J4<br>6<br>3

11.2 DISTRIBUTION OF OUTCOMES AND EXPLANATORY VARIABLES

Child outcomes are obviously central to the study. In developing the project and its design we defined an outcome largely in terms very similar to those of, for example, Sanson et al. (2005) who note that '... an outcome is an attribute of the child at a particular point in time' (p.5). Child outcomes will generally be influenced by a range of inputs which might include parenting, health, social and educational services, as well as natural developmental processes – to name but a few. Furthermore, children's own behaviour and attributes can serve as influences on later outcomes. Although often closely related, outcomes are not the same as indicators of wellbeing. For example, poverty is not a child outcome – though it clearly impacts on the child's wellbeing and may well impact on the child's development in areas such as education, socialisation, physical health, emotional and behavioural development, and so on. In contrast, health status, parent-child relationships and the level of educational attainment are all examples of outcomes.

Outcomes may be considered from different perspectives, such as at the level of the individual child, but also in terms of family outcomes and outcomes for society in general. Given the child-centred nature of ***Growing Up in Ireland*** our focus is clearly on the longitudinal development of child outcomes over time – though outcomes for the family are also of relevance. Much in line with LSAC (2005), the Canadian National Longitudinal Study of Children and Youth (NLSCY) and the National Children's Strategy (2000) we consider outcomes in the following three domains[14]

- Physical health and development
- Social/emotional/behavioural well-being
- Educational achievement and intellectual capacity.

Within these areas we can differentiate between two types of outcomes: developmental outcomes and performance outcomes (or assessments). Developmental outcomes are cumulative (such as intellectual capacity) and involve gains and losses over time. Performance outcomes, on the other hand, are the specific levels attained during assessments within the study. Being longitudinal, of course, we focus on the developmental trajectory of the child, plotted by linking his/her outcomes over time in the three domains outlined above. In many respects all of the information recorded in the first wave of the project may be construed as an input to the child's developmental trajectory, since first wave details, characteristics and outcomes will be inputs to child outcomes in subsequent rounds of the project.

---

[14] For a full discussion of outcomes adopted for the project see GUI Literature Review Series Paper No. 1 Background and Conceptual Framework.

It is worth noting that outcomes can also function as inputs at a single point in time – not just longitudinally. For example, ill health may be considered an outcome, but it may also be an input with regard to the child's later emotional or economic wellbeing. It depends on the particular analytical context.

Not all of the variables contained in the study instruments are outcome measures - some are explanatory variables that may be used in analytical models either cross-sectionally or longitudinally. To describe the proportion of variables that relate primarily to child outcomes, in contrast to those variables that are primarily explanatory or descriptive, a simple classification exercise was undertaken. All instruments completed by respondents were included in the exercise with the exception of non-resident and carer questionnaires as these were completed for a minority of children only. Only non-filtered questions were included, in other words only those questions which were asked of all respondents completing a given instrument. For example, on the Mother/Lone Father Questionnaire question C16. 'Does the Study Child usually require ongoing support to be able to move around?' was included, but the questions routed on a 'yes' answer were not. Hence question C17, 'What supports does the Study Child require?' was excluded. For the purposes of this exercise it was assumed that the biological mother was completing the Mother/Lone Father Questionnaire to include questions on pregnancy, etc in the analysis. One each of the child sensitive supplements, adult sensitive supplements and Drumcondra Reading Vocabulary and Maths Tests (3rd class) were included for the purposes of calculating the percentage of items falling into a particular group. In generating the distribution of outcome and explanatory variables, each item was given a single classification. It should also be borne in mind that all items could also be used, potentially, to produce descriptive statistics.

As can be seen from the shaded section of Table 11.5 there is a total of 603 non-filtered items/questions across all relevant instruments (Sections A to I of the table). A total of 364 (60%) of these referred primarily to child outcomes. Fifteen percent of items referred to intellectual/educational outcomes, 36% to emotional/behavioural outcomes and 8%to health outcomes.

All remaining items were classified as either explanatory or descriptor variables. When the descriptor/explanatory variables of Teacher-on-Self, Principal and Adult sensitive instruments are added to the analysis (in Sections J to L of Table 11.5) one can see that the total number of non-filtered items across all instruments rises to 804 – 55 % of which were classified as explanatory variables.

Table 11.5: Classification of items as outcome, descriptor or explanatory variables (i) in all instruments and (ii) only for instruments containing child-outcome-related items (shaded section).

Questionnaire/ Instrument	Health/ Development	Intellectual/ Educational	Emotional/ Behavioural	**Total Outcomes**	**Total Other Variables**
A. Mother/Lone Father Main	36	7	74	**117**	**152**
% within instrument A	13%	3%	28%	**43%**	**57%**
B. Father/Partner Main	0	0	43	**43**	**19**
% within instrument B	0%	0%	69%	**69%**	**31%**
C. Child Main	15	5	12	**32**	**28**
% within instrument C	25%	8%	20%	**53%**	**47%**
D. Child Core Sensitive	0	0	5	**5**	**14**
% within instrument D	0%	0%	26%	**26%**	**74%**
E. Child Sensitive Supplemental (1)	0	0	1	**1**	**21**
% within instrument E	0%	0%	5%	**5%**	**95%**
F. Teacher-on-Pupil	0	16	25	**41**	**5**
% within instrument F	0%	35%	54%	**89%**	**11%**

G. Piers-Harris	0	0	60	**60**	**0**
% within instrument G	0%	0	100%	**100%**	**0%**
H. Drumcondra Reading (3rd)	0	40	0	**40**	**0**
% within instrument H	0%	100%	0%	**100%**	**0%**
I. Drumcondra Maths (3rd)	0	25	0	**25**	**0**
% within instrument I	0%	100%	0%	**100%**	**0%**
No. items based on A - I only	*51*	*93*	*220*	***364***	***239***
% items based on A - I	*8%*	*15%*	*36%*	***60%***	***40%***
J. Teacher-on-self	0	0	0	**0**	**72**
% within instrument J	0%	0%	0%	**0%**	**100%**
K. Principal	0	0	0	**0**	**100**
% within instrument K	0%		0%	**0%**	**100%**
L. Sensitive supplemental (1)	0	0	0	**0**	**25**
% within instrument L	0%	0%	0%	**0%**	**100%**
No. items based on A - L	*51*	*93*	*220*	***364***	***436***
% based on A - L	*6%*	*12%*	*28%*	***46%***	***55%***

11.3 THE LONGITUDINAL APPROACH

Growing Up in Ireland is, by definition, longitudinal in nature. This is the optimal vehicle for recording information on change in the population (Magnusson, 2000; Magnusson & Bergman, 2000). The longitudinal approach greatly enhances the analytical potential of the project. It has two main advantages over cross-sectional studies. First, it allows one to examine change at the micro-level of the study child and his/her family and to consider the impact of changes which are specific to the individual child and family on developmental and other outcomes. The measurement and analysis of dynamic relationships over time can be achieved only by using good quality longitudinal data. For example, longitudinal data and analysis allow a better understanding of causal processes (as cause precedes effect) and means that analysis can be made of effects that are immediate, sequential, lagged or latent. Secondly, the longitudinal design allows one to control the analysis for unobserved characteristics of the child and his/her family and environments which do not change over time. Examining the effect of different levels of influence across different domains, contexts and time is a particularly important aspect of ***Growing Up in Ireland.*** The move towards a more complex, interactive view of development over the life course has been facilitated by 'enormous advances in quantitative statistical approaches, arguably especially in the longitudinal methods required to appraise the changing relations in the developmental system between the individual and the context' (Lerner, 2006, p.5). Although many of the advantages of the longitudinal approach will mature only after the third and subsequent waves of data collection, ***Growing Up in Ireland*** represents a major advance on what has hitherto been available for research into children and childhood in Ireland.

A longitudinal focus on the child cohort will allow us to analyse and interpret changes taking place at this particularly important developmental phase in the lives of the study children as they make the transition to early adolescence, with all the physical, emotional, psychological and institutional changes[15] involved

[15] The institutional changes in question refer principally to the education system as the children make their transition to second level.

in that particular period of their lives. After two waves it will be possible to draw some conclusions about the developmental trajectories between childhood and early adolescence.

Within the complex of factors which impact on child outcomes some can increase the risk of poor outcomes whereas others are protective. Indeed, the same factor can be both protective and risk-inducing, depending on the outcome examined and the temporal period in question. As an example, the model hypothesises that processes contributing to a dysfunctional outcome will have more impact in a disadvantaged context, whereas processes contributing to an outcome of competence will have more impact in an advantaged or stable environment. In simple terms, a negative variable would be more likely to worsen a situation that is already quite bad and a positive variable would be more likely to improve a situation that is already quite good. Risk inducing and protective factors can thus be cumulative in nature with each factor increasing or decreasing the probability of a specific outcome in an additive or exponential fashion (c.f. Layte & Whelan 2002). So, for example, being the child of a single mother might be considered a risk factor on its own, but the chances of a poor outcome for that child are increased in the presence of other risk factors such as poverty or substance misuse. Similarly, a person may arrive at a poor life outcome (or indeed a positive life outcome) through a number of different routes – hence the advantage of a longitudinal approach in tracing and unpacking individual pathways.

A protective factor refers to something that has ‘an effect that operates only (or mainly) as a resistance against a risk factor’ (Rutter, 2006, p.20). For example, participation in an after-school homework club might help protect a child who is left to fend for himself/herself for hours after school against delinquency or school failure. For children who return to a responsible adult and a supportive home environment after school, participation in a homework club may make no difference to his/her risk of delinquency.

A principal aim of ***Growing Up in Ireland*** is to identify risk and protective factors and, in so doing, to assist in the development of effective policies which help to maximise the welfare of all children and their families. The design outlined in the previous chapters will allow us to more than meet this overarching objective of the study.

Chapter 12

SUMMARY

CHAPTER 12: SUMMARY

The purpose of this report was to describe in detail the design, instruments and procedures used to implement the child cohort of Growing Up in Ireland. The focus throughout was on operational issues as well as the content, structure and format of the instrumentation and related documentation.

Growing Up in Ireland has a key role in the implementation of the National Children's Strategy (2000). The project has nine key objectives relating to the development of a comprehensive data bank on the whole child and all the variations encompassed by that concept. In the present report we have outlined in detail the design and implementation of the project aimed at ensuring that the data captured in respect of the whole child has been fully achieved. The work has been carried out within the broad bioecological model underlying the project which will allow analysis of child outcomes and outcome trajectories within the Bronfenbrenner framework (Chapter One).

We described the broad outline of the sample design. This was based on a two-stage schema with the initial selection of a random sample of national schools as primary sampling units. Children who fell within the age range were then selected in each school (PSU). The data were reweighted prior to analysis using an iterative procedure to adjust sample totals to column marginals derived from external sources: administrative data on schools from the Department of Education and Science and data provided by the Central Statistics Office (Chapter Two).

The background to the development and design of procedures was discussed in full and this outlined the important inputs, especially from various advisory committees. We discussed the work of the Scientific and Policy Advisory Committee (SPAC), the Panel of Expert Advisors, the Delphi process used in questionnaire development, the Children's Advisory Forum, and the various stakeholder groups and the key groups in the overall governance structure of the project – the Project Team and Steering Groups (Chapter Three).

The Study Team was very conscious of its responsibilities in conducting a scientifically rigorous, ethically sound study to the highest international standards. The overall study substantially benefits from a multi-layered and interlocking governance structure – the overarching element of which is a high level Inter-Departmental Steering Group and Working Group (the latter referred to as the Project Team). A particularly important aspect of the monitoring structure is the Research Ethics Committee. The importance of rigorous ethical protocols in research is assuming an ever-increasing priority, all the more so in a study of children and families. Procedures and protocols to ensure that the study has been carried out to the highest ethical standards have been put in place. The fact that the project is being carried out under the Statistics Act (1993) has been extremely important for the conduct of the study. This is the legislation which underpins the work of the Central Statistics Office (CSO). While the Statistics Act facilitates access to certain data sources, its most important implication is that it provides a particularly strong legal basis for the protection of all information collected from all informants. Under the Act, the information collected must be treated as strictly confidential and used only for statistical purposes. The protection of the data against unlawful disclosure greatly strengthened the Study Team's guarantee of confidentiality (Chapter Four).

The project has successfully recorded information from 8,500 children and their families. As noted above, the first point of contact with the participants was through the national school system which was used for sample generation and to secure informed consent from children and their families. When consent had been secured, questionnaires were completed in the schools by the teachers in respect of each participating child, along with an academic assessment test in English and Maths (the Drumcondra tests). In addition, the teachers and school principals completed questionnaires in respect of themselves and their schools (Chapter Five and Six).

When data collection was completed in the school the focus of the study moved to the home. Intensive questionnaires were completed by the child and his/her primary and secondary caregivers (where

relevant). The instruments used contained a number of standardised measures with information being recorded on a broad range of variables, which can both affect and describe the life of a nine-year-old child in contemporary Ireland. These areas include health, parenting, family context, pastimes and activities, education, intellectual capacity, temperament, income, and community. Throughout all the questionnaires used in the study there was an emphasis on obtaining children's views and opinions on their lives, including some qualitative information on issues such as role models and aspirations. In addition, an attempt was made to record details from non-resident parents and non-cohort caregivers (Chapters Seven to Ten).

The focus throughout the study was child outcomes and factors which impacted upon them. We saw that 60% of all items in the main household-based questionnaires were directly oriented towards outcomes. The remaining items were descriptive and explanatory variables, which will be used to analyse and understand the processes and drivers of child development – both cross-sectionally and longitudinally (Chapter Eleven).

Growing Up in Ireland is wholly funded by the Irish government with a primary aim of addressing policy issues and providing a very direct input to policy formation. This extremely complex, intensive, long-term project will bridge many of the gaps in data available on Irish children and childhood. It will enable the assessment, over time, of whether or not key national goals of child development and policy are being achieved – be they measured in terms of individual outcomes of the child and his/her family or in terms of access to services aimed at children and families. The project will enable us to identify children who are most at risk of less than optimal development and poor outcomes and, by identifying the early antecedents of poor outcomes, will substantially assist in developing preventive strategies and measures where they are most needed. Overall, of course, Growing Up in Ireland will, for the first time, allow us to develop a picture of the lives of all children in Ireland in their full diversity. In addition to a set of descriptive and analytical reports the project will very substantially contribute to the infrastructure of research into children's lives. All of the data included in the survey (as described in the above report) will be lodged in the Irish Social Science Data Archive for use by the research and policy communities.

REFERENCES

Adler, N. E., Epel, E. S., Castellazzo, G., & Ickovics, J. R. (2000), 'Relationship of subjective and objective social status with psychological and physiological functioning: Preliminary data in healthy White women', *Health Psychology*, 19, 586–592.

Alva, S., Elmore, A., Nord, C. W., & Zill, N. (2004), *High School Participation in Extracurricular Activities: Implications for Positive Outcomes Later in Life?* Paper presented at the annual meeting of the American Sociological Association [On-line]. Available: http://www.allacademic.com/meta/p110208_index.html

Alwash, R. & McCarthy, M. (1988), 'Accidents in the home among children under 5: Ethnic differences or social disadvantage', *British Medical Journal*, 296, 145 –1453.

Amato, P. R. & Gilbreth, J. G. (1999), 'Nonresident fathers and children's well-being: A meta-analysis', *Journal of Marriage and the Family*, 61, 55 –573.

Amato, P.R. & S. Rezac (1994), 'Contact with nonresident parents, interparental conflict, and children's behavior', *Journal of Family Issues*, 15, 191–207

Anderson, C.A. & Bushman, B.J. (2001), 'Effects of violent video games on aggressive behavior, aggressive cognition, aggressive affect, physiological arousal, and prosocial behavior: A meta-analytic review of the scientific literature', *Psychological Science*, 12, 5, 353–359.

Anderson, J. W., Johnstone, B. M., & Remley, D. T. (1999), 'Breastfeeding and cognitive development: a meta-analysis', *American Journal of Clinical Nutrition*, 70, 525–535.

Anderson, R. C., Fielding, L. G., & Wilson, P. T. 1988, 'Growth in reading and how children spend their time outside of school', *Reading Research Quarterly* 23, 285–304.

Amato, P. R. & Gilbreth, J. G. (1999), 'Nonresident fathers and children's well-being: A meta-analysis', *Journal of Marriage and the Family*, 61, 557–573.

American Psychiatric Association (1994), *Diagnostic and Statistical Manual of Mental Disorders (DSM-IV).* Fourth ed..(Washington DC, American Psychiatric Association).

Anti-Bullying Centre (2002), *School issues in bullying* (Dublin, Trinity College) [On-line]. Available: http://www.abc.tcd.ie/

Argys, L., Peters, E., Cook, S., Garasky, S., Nepomnyaschy, L., Sorensen, E., & Waller, M. (2003), 'Nonresidential parenting: Measuring contact between children and their nonresident fathers', in *Bethesda, MD: Paper presented at Measurement Issues in Family Demography Workshop.*

Arenz, S., Ruckerl, R., Koletzko, B., & Von Kries, R. (2004), 'Breastfeeding and childhood obesity: a systematic review',, *International Journal of Obesity and Related Metabolic Disorders*, 28, 1247–1256.

Avenevoli, S., Sessa, F. M., & Steinberg, L. (1999), 'Family structure, parenting practices and adolescent adjustment: An ecological examination', in E. M. Hetherington (ed.), *Coping with Divorce, Single Parenting, and remarriage: A Risk and Resiliency Perspective*, 65–90 (New Jersey, Lawrence Erlbaum Associates).

Baer, J., Baldi, S., Ayotte, K., & Green, P. (2007), *The Reading Literacy of U.S. Fourth-Grade Students in an International Context: Results From the 2001 and 2006 Progress in International Reading Literacy Study (PIRLS),* 2008–017 (National Center for Education Statistics, Institute of Education Sciences, U.S. Department of Education. Washington, DC).

Bandura, A., Barbarabellie, C., Caprara, G.V., & Pastorelli, C.,(1996), 'Multifaceted Impact of Self-Efficacy Beliefs on Academic Functioning', *Child Development*, 67, 1206–1222.

Barnett, L. A., & Chick, G. E. (1986), 'Chips off the ol' block: Parents' leisure and their children's play', *Journal of Leisure Research*, 18, 266–283.

Barry, S., Kearney, A., Lawlor, E., McNamee, E., & Barry, J. (2006), *The Coombe Women's Hospital study of alcohol, smoking and illicit drug use, 1988-2005.* Unpublished.

Baumrind, D. (1991), 'Effective parenting during the early adolescent transition', in P. A. Cowen and E. M. Hetherington (eds.), *Advances in family research*, 2, 111–163 (Hillsdale, NJ,Erlbaum).

Beardslee, W. R., Keller, M. B., Seifer, R., Podorefsky, D., Staley, J., Lavori, P. W., & Shera, D. (1996), 'Prediction of adolescent affective disorder: Effects of prior parental affective disorders and child psychopathology', *Journal of the American Academy of Child and Adolescent Psychiatry*, 35, 279–288

Beardslee, W. R. & Podorefsky, D. (1988), 'Resilient adolescents whose parents have serious affective and other psychiatric disorders: Importance of self-understanding and relationships', *American Journal of Psychiatry*, vol. 145, no. 1: 63–69

Becker, A., Woerner, W., Hasselhorn, M., Banaschewski, T., & Rothenberger, A. (2004), 'Validation of the parent and teacher SDQ in a clinical sample', *European Child and Adolescent Psychiatry* 13 (Supplement 2): 11–16.

Bee, H. & Boyd, D. (2007), *The Developing Child,* 11th edn. (Boston, Pearson Education).

Belsky, J. (1984), 'The determinants of parenting: a process mode', *Child Development*, 55: 83–96.

Benard, B. (1991), *Fostering Resiliency in Kids: Protective Factors in the Family, School, and Community* (Portland, Oregon, Western Center for Drug-Free Schools and Communities).

Bernardini, S. C. & Jenkins, J. M. (2002), *An Overview of Risks and Protectors for Children of Separation and Divorce*, Rep. No. 2E (Ottawa, Department of Justice Canada; Family, Children and Youth Section).

Bess, F, Dodd-Murphy, J., & Parker, R. (1998), 'Children with minimal sensorineural hearing loss: Prevalence, educational performance, and functional status', *Ear Hear,*19: 339–354.

BioMed Central (May 12, 2007), 'Curb the Pocket Money, Curb the Problem Drinking', *Science Daily*. Retrieved 14/12/2007 from http://www.sciencedaily.com/releases/2007/05/070510194102.htm

Blaxter, M. (1989), 'A comparison of measures of inequality in morbidity', in A. J. Fox (ed.), *Health Inequalities in European Countries*, 199–230 (Aldershot, Gower).

Boer, F. & Westenberg, P. M. (1994), 'The factor structure of the Buss & Plomin EAS Temperament Survey (parental ratings) in a Dutch sample of elementary school children', *Journal of Personality Assessment*, 62(3): 537–551.

Boyce, W. T., Jensen, E. W., James, S. A., & Peacock, J. L. (1983), 'The Family Routines Inventory: Theoretical origins', *Social Science & Medicine*, 17: 193–200.

Bradbury, T. N., Fincham, F. D., & Beach, S. R. H. (2000), 'Research on the nature and determinants of marital satisfaction: A decade in review', *Journal of Marriage and the Family*, 62: 964–980.

Breslau, N., Paneth, N. S., & Lucia, V. C. (2004), ‘ The Lingering Academic Deficits of Low Birth Weight Children’, *Pediatrics*, 114: 1035–1040.

Bronfenbrenner, U. (1979), *The ecology of human development: Experiment by nature and design* (Cambridge, Harvard University Press).

Bronfenbrenner, U. & Morris, P. (2006), ‘The bioecological model of human development’, in R. M.V. Lerner, W. Damon, & R. M. S. Lerner (eds.), *Handbook of Child Psychology, Vol. 1: Theoretical Models of Human Development,,* 6th edn., 793–828 (Hoboken, NJ, Wiley).

Brooks-Gunn, J., Duncan, G. J., Klebanov, P. K., & Sealand, N. (1993), ‘Do Neighbourhoods Influence Child and Adolescent Development?’, *American Journal of Sociology*, 353–395.

Buss, A. H. & Plomin, R. (1984, *Temperament: Early Developing Personality Traits* (Hillsdale, NJ.,Lawrence Erlbaum Associates).

Callan, T., Nolan, B. & Whelan, C. T. (1993), ‘Resources, deprivation and the measurement of poverty’, *Journal of Social Policy*, 22: 2

Carlson, M. & Corcoran, M. (2001), ‘Family structure and children's behavioral and cognitive outcomes’, *Journal of Marriage and the Family*, 63: 779–792.

Central Statistics Office (2006), *Quarterly National Household Survey: Childcare, Quarter 1 2005* (Dublin, Central Statistics Office).

Charlton, T., Panting, C., & Hannan, A. (2002), ‘Mobile telephone ownership and usage among 10- and 11-year-olds’, *Emotional and Behavioural Difficulties*, 7, 3: 152–163.

Cheng, H., Dunn, J., O'Connor, T., & Golding, J. (2006), ‘Factors Moderating Children's Adjustment to Parental Separation: Findings From a Community Study in England’, *Journal of Abnormal Child Psychology,* 34: 230-241.

Christian, K., Morrison, F.J. & Bryant, F. B. (1998), ‘Predicting kindergarten academic skills: Interactions among child care, maternal education and family literacy environments’, *Early Childhood Research Quarterly*, 13, 3: 501–521.

Clarke, E. C., Cooksey, E. C., & Verropoulou, G. (1998), ‘Fathers and absent fathers: Sociodemographic similarities in Britain and the United States’, *Demography*, 35: 217–228.

Collins, C. (2001)., *Design of the National Children’s Strategy Longitudinal Study of Children*. Unpublished report submitted to the Health Research Board by the consortium represented by S. Greene, A. Hyland, C. Kelleher, S. Mennell, B. Whelan & J. Wilde.

Collins, M. E. (1991), ‘Body figure perceptions and preferences among preadolescent children’, *International Journal of Eating Disorders*, 10: 199–208.

Cotton, K. (2000), *The Schooling Practices that Matter Most* (Northwest Regional Educational Laboratory).

Cronbach, L. J. (1988), ‘Internal consistency of tests: Analyses old and new’, *Psychometrika*, 53: 63–70

Cullinan, B.E. (2000), *Independent Reading and School Achievement* (School Library Media Research, 3. http://news.ala.org/ala/aasl/aaslpubsandjournals/slmrb/slmrcontents/volume32000/independent.cfm

Dalziel, D. & Henthorne, K. (2005), *Parents'/Carers' Attitudes towards School Attendance. Report for Department for Education and Skills* (Research Report No. 618).

Darling, N. & Toyokawa, T. (1997), *Construction and Validation of the Parenting Style Inventory II (PSI-II).* Available online at: http://www.oberlin.edu/faculty/ndarling/lab/psiii.pdf

Dawe, S., Frye, S., Best, D., Moss, D., Atkinson, J., Evans, C., & Lynch, M. H. P. (2007), *ANCD Research Paper: Drug use in the family: impacts and implications for children* (Australian National Council on Drugs).

Department of Health and Children (1999), *Children First: National Guidelines for the Protection and Welfare of Children* (Dublin, The Stationery Office).

Department of Health and Children (2000), *The National Children's Strategy: Our Children – Their Lives* (Dublin, The Stationery Office).

Department of Health and Children (2006), *State of the Nation's Children – Ireland 2006* (Dublin, The Stationery Office).

Dickey, W. C. & Blumberg, S. J. (2004), 'Revisiting the factor structure of the Strengths and Difficulties Questionnaire: United States, 2001', *Journal of the American Academy of Child and Adolescent Psychiatry* 43: 1159–1167.

DiClemente, R. J., Wingood, G. M., Lang, D. L., Crosby, R. A., Salazar, L. F., Harrington, K. & Hertzberg, V. S. (2005), 'Adverse Health Consequences that Co-Occur with Depression: A Longitudinal Study of Black Adolescent Females', *Pediatrics*, 116: 78–81.

Dickstein, S. (2006), *Family Mealtime Functioning, Maternal Depression, and Early Childhood Outcomes*. http://www.apa.org [On-line]. Available: http://www.apa.org/pi/cyf/fam3.html

Dornbusch, S., Ritter, P., Leiderman, P., Roberts, D., & Fraleigh, M. (1987), 'The relation of parenting style to adolescent school performance', *Child Development*, 58: 1244–1257.

Downey, S., Hayes, N., & O'Neill, B. (2007), *Play and Technology for children aged 4-12* (Dublin, Ireland, Centre for Social and Educational Research, Dublin Institute of Technology).

Downes, P., Maunsell, C., & Ivers, J. (2006), *A Holistic Approach to Early School Leaving and School Retention in Blanchardstown: Current Issues and Future Steps for Services and Schools* (Dublin, Blanchardstown Area Partnership).

Dunn, J. (1996), 'Arguing with siblings, friends, and mothers: Developments in relationships and understanding', in D. Slobin, J. Gerhardt, A. Kyratzis, & J. Guo (eds.), *Social interaction, social context, and language: Essays in honor of Susan Ervin-Tripp* (Mahwah, NJ, Lawrence Erlbaum Associates).

Eamon, M. K., & Zuehl, R. M. (2001), 'Maternal Depression and Physical Punishment as Mediators of the Effect of Poverty on Socioemotional Problems of Children in Single-Mother Families', *American Journal of Orthopsychiatry*, 71(2): 218–226.

Eccles, J. S., Wigfield, A., & Schiefele, U. (1998), 'Motivation to succeed', in W. Damon (ed.), *Handbook of Child Psychology: Social, emotional and personality development,* 5th ed.n), 1017–1096 (New York, Wiley).

Educational Research Centre (2006a), *Drumcondra Primary Mathematics Test – Revised. Level 2 Administration Manual and Technical Manual* (Dublin, Educational Research Centre, St Patrick's College).

Educational Research Centre (2006b), *Drumcondra Primary Mathematics Test – Revised. Levels 3-6 Administration Manual and Technical Manual.* (Dublin: Educational Research Centre, St Patrick's College).

Educational Research Centre (2007a), *Drumcondra Primary Reading Test – Revised. Level 2 Administration Manual and Technical Manual* (Dublin, Educational Research Centre, St Patrick's College).

Educational Research Centre (2007b), *Drumcondra Primary Reading Test – Revised. Levels 3-6 Administration Manual and Technical Manual* (Dublin: Educational Research Centre, St Patrick's College).

Edward, S. & Malcolm, H. (2002), *The Causes and Effects of Truancy* (Edinburgh, Report for SCRE).

Eiser, C. (1997), 'Effects of chronic illness on children and their families', *Advances in Psychiatric Treatment*, 3: 204–210.

Erel, O. & Burman, B. (1995), 'Interrelatedness of marital relations and parent-child relations: A meta-analytic review', *Psychological Bulletin*, 118: 108–132.

Etelson, D., Brand, D. A., Patrick, P. A., & Shirali, A. (2003), 'Childhood obesity: Do parents recognize this health risk?', *Obesity Research*, 11(11): 1362–1368.

Eurostat (2003), *Eurostat yearbook 2003: the statistical guide to Europe: data 1991–2001* (Luxembourg, Office for Official Publications of the European Communities)..

Faber, A. & Mazlish, E., (1999), *How to Talk so Kids Will Listen and Listen so Kids will Talk* (NY, Harper Collins).

Fahey, T., Delaney, L., & Gannon, B. (2005), *School Children and Sport in Ireland* (Dublin, The Economic and Social Research Institute).

Feinstein, L. S. J. (1999), *Attainment in Secondary School* (Oxford Economic Papers, 1999 - Oxford Univ Press, 51).

Fekkes, M., Piipers, F. I. M., & Verloove-Vanhorick, S. P. (2006), 'Effects of Antibullying School Program on Bullying and Health Complaints', *Archives of Pediatric Adolescent Medicine*, 160,:, 638–644.

Field, A. (2005), *Discovering Statistics Using SPSS*, 2nd Edn. (London,, Sage).

Flay, B. R., Hu, F. B., Siddiqui, O., Day, E., Hedeker, D., Petraitis, J., Richardson, J., & Sussman, S. (1994), 'Differential Influence of Parental Smoking and Friends' Smoking on Adolescent Initiation and Escalation and Smoking', *Journal of Health and Social Behaviour*, 35: 248–265.

Friel, S., Hope, A., Kelleher, C., Comer, S., & Sadlier, D. (2002), 'Impact evaluation of an oral health intervention amongst primary school children in Ireland', *Health Promotion International*, 17: 119–126.

Fullarton, S. (2002), *Student Engagement with School: Individual and School-Level Influences* (Longitudinal Surveys of Australian Youth) [On-line]. Available: http://www.acer.edu.au/documents/LSAY_lsay27.pdf

Gomulka, J., (1992), 'Grossing Up Revisited' in R. Hancock and H. Sutherland (eds.) *Microsimulation Models for Public Policy Analysis: New Frontiers, STICERD* (Occasional Paper 17, LSE).

Gomulka, J., (1994). 'Grossing Up A Note on Calculating Household Weights from Family, Composition Totals', (University of Cambridge, Department of Economics, Microsimulation Research Note MU/RN/4, March 1994).

Gannon, B. & Nolan, B. (2005), *Disability and Social Inclusion in Ireland* (Dublin, Equality Authority and National Disability Authority).

Gasman, I., Purper-Ouakil, D., Michel, G., Mouren-Simeoni, M.C., Bouvard, M., Perez-Diaz, F. & Jouvent, R. (2002), 'Cross-cultural assessment of childhood temperament: A confirmatory factor analysis of the French Emotionality, Activity and Sociability (EAS) questionnaire', *European Child and Adolescent Psychiatry*, 101–107.

Gershoff, E. T. (2002), 'Corporal punishment by parents and associated child behaviours and experiences: A meta-analytic and theoretical review', *Psychological Bulletin*, 128: 539–579.

Glascoe, F.P. (2006). *Parents' Evaluation of Developmental Status: An Evidence-based Approach to Developmental/Behavioral Screening and Surveillance* (Nashville, Tennessee, Ellsworth & Vandermeer Press).

Goodman, R (1997), 'The Strengths and Difficulties Questionnaire: A Research Note', *Journal of Child Psychology and Psychiatry*, 38: 581–586

Goodman, R. (2001), 'Psychometric properties of the Strengths and Difficulties Questionnaire', *Journal of the American Academy of Child Adolescent Psychiatry*, 1337–1345.

Goodman, R, & Scott, S. (1999). 'Comparing the Strengths and Difficulties Questionnaire and the Child Behavior Checklist: Is small beautiful?', *Journal of Abnormal Child Psychology*, 27: 17–24.

Goodman, R., Renfrew, D., & Mullick, M. (2000), 'Predicting type of psychiatric disorder from Strengths and Difficulties Questionnaire (SDQ) scores in child mental health clinics in London and Dhaka, *European Child and Adolescent Psychiatry*, 9: 129–134.

Gortmaker, S. L., Must, A., Sobol, A. M., Peterson, K., Colditz, G. A., & Dietz, W. H. (1996), 'Television viewing as a cause of increasing obesity among children in the United States, 1986-1990', *Archives of Pediatric and Adolescent Medicine*, 150:, 356–362.

Greenwald, R., Hedges, L. V., & Laine, R. D (1996), 'The Effect of School Resources on Student Achievement', *Review of Educational Research* 66[3]: 361–396..

Greer, F. R. & Krebs, N. F. (2006),'Optimizing bone health and calcium intakes of infants, children, and adolescents', *Pediatrics,* 117[2]:, 578–585

Griffin, A. C., Younger, K. M., & Flynn, M. A. (2004), Assessment of obesity and fears of fatness among inner city Dublin school children in a one-year follow-up study. Public Health Nutrition 729-735.

Gruenewald, P. J. & Johnson, F. W. (2006). 'The Stability and Reliability of Self-Reported Drinking Measures', *Journal of Studies on Alcohol*, 67: 738–745.

Grusec, J. E. & Goodnow, J. J. (1994), 'Impact of Parental Discipline Methods on the Child's Internalization of Values: A Reconceptualization of Current Points of View', *Developmental Psychology,* 30: 1, 4–19.

Haas, S. A. (2007), 'The Long-Term Effects of Poor Childhood Health: An Assessment and Application of Retrospective Reports', *Demography,* 44[1]: 113–135.

Hack, M., Klein, N. K., & Taylor, H. G. (1995), 'Long-term developmental outcomes of low birth infants', *Future Child, Spring, 5*: 176–196.

Hannan, D. F., O' Riain, S., & Whelan, C. T. (1997), 'Youth Unemployment and Psychological Distress in the Republic of Ireland', *Journal of Adolescence*, 20: 307–320.

Hannan, D. F. Smyth, E., McCullagh, J., O'Leary, R., & McMahon, D. (1996), 'Coeducation and Gender Equality: Exam Performance, Stress and Personal Development',, General Research Series 169 9Oak Tree Press in association with the ESRI).

Hanushek, E. A. (1997,. 'Assessing the Effects of School Resources on Student Performance: An Update',. *Educational Evaluation and Policy Analysis*, 19: 141–164.

Hanushek, E. A. (2003), 'The Failure of Input-based Schooling Policies', *The Economic Journal*, 113:,, F64–F98.

Hawes, D. J. & Dadds, M. R. (2004), 'Australian data and psychometric properties of the Strengths and Difficulties Questionnaire',_*Australian and New Zealand Journal of Psychiatry*, 38: 644–651.

Higgins, S., McArdle, J., McEvoy, R., & Tully, A. (2005), *Munch & Crunch, Healthy Lunch Project: Evaluation Report* (Waterford, South Eastern Area, Health Promotion Department, Health Service Executive).

Hoover-Dempsey, K. V., Battiato, A. C., Walker J. M. T., Reed, M. P., DeJong J. M., Jones, K. P. 'Parental Involvement in Homework', *Educational Psychologist*, 36, 3: 195–209.

Huba, G. J., Melchior, L. A., and Panter, A. T. (1998–2001), 'The Measurement Group Knowledge Base on HIV/AIDS Care'. On the World Wide Web: http://www.TheMeasurementGroup.com

Hunsley, J., Best, M., Levebvre, M. and Vito, D. (2001), 'The Seven-Item Short Form of the Dyadic Adjustment Scale: Further Evidence for Construct Validity', *The American Journal of Family Therapy*, 29: 325–335.

Hunsley, J., Pinsent, C., Lefebvre, M., James-Tanner, S., & Vito, D. (1995), 'Construct validity of the short forms of the Dyadic Adjustment Scale', *Family Relations*, 44: 231–237.

Johnson, D. W., Shah, M. & Shields, M. A. (2007). 'Handedness, Time Use and Early Childhood Development. Discussion Paper No. 2752'. http://ftp.iza.org/dp2752.pdf

Kalkwarf, H. J., Khoury, J. C., Lanphear, B. P. (2003). 'Milk intake during childhood and adolescence, adult bone density, and osteoporotic fractures in US women', *Am J Clin Nutr*, 77:257–65.

Kandel, E. & Mednick, S. A. (1991), 'Perinatal complications predict violent offending', *Criminology*, 29: 519-529.

Kearney, C. A. (2003). Bridging the gap among professionals who address youths with school absenteeism: Overview and suggestions for consensus. Professional Psychology: Research and Practise, 34, 57-65.

Kelly, Y. J., Nazroo, J. Y., McMunn, A., Boreham, R., & Marmot, M. (2001), 'Birthweight and behavioural problems in children: a modifiable effect?', *International Journal of Epidemiology,* 88–94.

Kelvin, R. G., Goodyer, I. M., & Altham, P. M. E. (1996), 'Temperament and psychopathology amongst siblings of probands with depressive and anxiety disorders', *Journal of Child Psychology and Psychiatry,* 37: 543–550.

Kenkel, D., Lillard, D. R., & Mathios, A. (2003), 'Smoke or fog? The usefulness of retrospectively reported information about smoking', *Addiction* 98[9]: 1307–1313.

Kerr, D. C. R., Lopez, N. L., Olson, S. L., & Sameroff, A. J. (2004), 'Parental discipline and externalising behaviour problems in early childhood: The roles of moral regulation and child gender', *Journal of Abnormal Child Psychology*, 32, 4: 369–383, 32: 369–383.

Kielty, S. (2006), 'Similarities and differences in the experiences of non-resident monthers and non-resident fathers', *International Journal of Law, Policy and the Family*, 20: 74–94.

Kish., L. (1965), *Survey Sampling* (New York: John Wiley and Sons).

Klasen, H., Woerner, W., Wolke, D., Meyer, R., Overmeyer, S., Kaschnitz, W., Rothenberger, A. & Goodman, R. (2000), 'Comparing the German versions of the Strengths and Difficulties Questionnaire (SDQ-Deu) and the Child Behavior Checklist', *European Child and Adolescent Psychiatry*, 9: 271–276.

Kleiber, D. A. (1999), *Leisure Experience and Human Development* (New York, Basic Books).

Koskelainen M, Sourander A, Kaljonen A (2001) The Strengths and Difficulties Questionnaire among Finnish school-aged children and adolescents. European Child and Adolescent Psychiatry, 9, 277-284.

Kosonen, H., Rimpelä, A., Rauma, A. L., Väisänen, P., Pere, L., Virtanen, S. M., Rimpelä, M. (2005), 'Consumption of special diet among Finnish adolescents in 1979-2001: repeated national cross-sectional surveys', *SOZ PRAVENTIVMED*, 50:142–150.

Kuder,G. F., & Richardson, M. W. (1937). 'The theory of estimation of test reliability', *Psychometrika*, 2: 151–160.

Krueger, A. B. (2002), 'Economic considerations and class size', *Economic Journal* 113: F34–F63

Lamdin, D. J. (1996), 'Evidence of student attendance as an independent variable in education production functions', *Journal of Educational Research*, 89: 155–162.

Layte, R. (2004), 'Equity in the use of Health Care in Ireland', in B.Nolan (ed.), *The Provision and Use of Health Services, Health Inequalities and Health and Social Gain,* 164–176 (Dublin: The Economic and Social Research Institute).

Layte, R. & Nolan, B. (2004), 'Equity in the Utilisation of Health Care in Ireland', *The Economic and Social Review*, 35: 111–134.

Layte, R., Nolan, B., Whelan, C. T. (2001), 'Reassessing income and deprivation approaches to the measurement of poverty in Ireland', *Economic and Social Review*, 32: 3.

Leon, D. A., Lithell, H. O., Vagero, D., Koupilova I.., Mohsen, R., Berglund, L., Lithell, U. B., & McKeigue, P. M. (1998), 'Reduced foetal growth rate and increased risk of death from ischaemic heart disease: cohort study of 15,000 Swedish men and women born 1915–1929', *British Medical Journal,* 317: 241–245.

Li, J. S., Peat, J. K., Xuan, W., & Berry, G. (1999), 'Meta-analysis on the association between environmental tobacco smoke (ETS) exposure', *Pediatric Pulmonology,* 27: 5–13.

Li, R., Scanlon, K. S., & Serdula, M. K. (2005), 'The validity and reliability of maternal recall of breastfeeding practice'*, Nutrition Reviews*, 63: 103–110.

Livingstone, M. B. E. (2001), 'Childhood Obesity in Europe: a growing concern', *Public Health Nutrition,* 4: 109–116.

Lundberg, S., McLanahan, S., & Rose, E. (2005), 'Child gender and father involvement in fragile families',(Working Paper, Department of Economics, Cent. Res. Fam., Univ. Washington).

Ma, X. (1999), ' Dropping out of advanced mathematics: The effects of parental involvement', *Teachers College Record* 101[1]: 60–81.

Maes, L., Maser, M. & Honkala, S. (2004), 'Oral health' , In Young People's Health in Context; Health Behaviour in School-aged Children (HBSC) Study: International Report from the 2001/2002 Survey, C. Currie, C. Roberts, A. Morgam, R. Smith, W. Setterbulte, O. Samdal and V. B. Rasmusen (eds.), (Denmark, World Health Organisation).

Maitre, B., Nolan, B, & Whelan, C.T. (2006), 'Reconfiguring the measurement of deprivation and consistent poverty in Ireland', *Policy Research Series*, 58.

Marsh, H.W., & Craven, R.G. (2006), 'Reciprocal effects of self-concept and performance from a multidimensional perspective: Beyond seductive pleasure and unidimensional perspectives', *Perspectives on Psychological Science*, 1: 133–163.

Marzano, R. J. (2002), 'A New Era of School Reform: Going Where the Research Takes Us', *Mid-continent Research for Education and Learning (McREL)* [On-line]. Available: http://www.mcrel.org/PDF/SchoolImprovementReform/5002RR_NewEraSchoolReform.pdf

Mathai, J., Anderson, P., & Bourne, A. (2002), 'The Strengths and Difficulties Questionnaire (SDQ) as a screening measure prior to admission to a child and adolescent mental health service (CAMHS)', *Australian e-Journal for the Advancement of Mental Health*, 1(3): 1446–7984.

Mathiesen, K. S. and Tambs, K. (1999). 'The EAS Temperament Questionnaire – Factor Structure, Age Trends, Reliability, and Stability in a Norwegian Sample', *The Journal of Child Psychology and Psychiatry and Allied Disciplines,* 40: 431–439.

McCormick, M. C. & Brooks-Gunn, J. (1999), 'Concurrent Child Health Status and Maternal Recall of Events in Infancy', *Pediatrics*, 104: 1176–1181.

McCoy, S., Darmody, M, Smyth, E., Dunne, A. (2007), *Attendance and Students' Schools Experiences* (Dublin, ESRI and the National Educational Welfare Board).

McGinnity, F., Russell, H., Williams, J. & Blackwell, S. (2005), *Time Use in Ireland 2005: Survey Report* (Dublin, ESRI Books and Monographs, 183).

McKeown, K., Pratschke, J., & Haase, T. (2003), *Family Wellbeing: What Makes a Difference? Insights and Initiatives for a Changing Society* (County Clare, Ireland, Report to the Céifin Centre).

Meece, J. L., Anderman, E. M., & Anderman, L. H. (2006), 'Classroom Goal Structure, Student Motivation and Academic Achievement', *Annual Review of Psychology*, 57: 487–503.

Melchior, Huba, Brown and Reback (1993), 'A Short Depression Index for Women', *Educational and Psychological Measurement*, 53: 1117–1125

Mortimer, P., Sammons, P., Stoll, L., Lewis, D. and Ecob, R.(1988), *School Matters: The Junior Years* (London: Open Books).

Mulkerrins, D. (2007), 'The Transformational Potential of the Home School Community Liaison Scheme', in P. Downes and A. L. Gilligan (eds.), *Beyond Educational Disadvantage* (Dublin, IPA).

Muris, P., Meesters, C., & van den Berg, F. (2003), 'The Strengths and Difficulties Questionnaire (SDQ): Further evidence for its reliability and validity in a community sample of Dutch children and adolescents', *European Child and Adolescent Psychiatry*, 12: 1–8.

Murphy, S. P., & Allen, L. H. (2003), 'Nutritional Importance of Animal Source Foods', in *J. Nutr*. 133: 3932S–3935S

Nathan, L., Stackhouse, J., Goulandris, N., & Snowling, M. (2004,. 'The development of early literacy skills among children with speech difficulties: A test of the "critical age hypothesis"', *Journal of Speech, Language, and Hearing Research,* 47: 377–391.

National Children's Strategy: Our Children, Their Lives (2000), (Dublin, The Stationery Office).

National Commission on Education (1996), *Success Against the Odds: Effective Schools in Disadvantaged Areas* (London: Routledge).

National Institute of Child Health and Human Development Early Child Care Research Network (2001), 'Child care and communicable illnesses: Results from NICHD Study of Early Child Care', *Archives of Pediatric and Adolescent Medicine*, 155: 481–488.

National Institute of Child Health and Human Development Early Child Care Research Network (2002), 'Child care structure-process-outcomes: Direct and indirect effects of child care quality on young children's development', *Psychological Science*, 13: 199–206.

National Institute of Child Health and Human Development Early Child Care Research Network (2003), 'Child care and communicable illnesses in children aged 37 to 54 months', *Archives of Pediatric and Adolescent Medicine*, 157: 196–200.

National Task Force on Obesity (2005), *Obesity the Policy Challenges - The Report of the National Task Force on Obesity* (Dublin, Department of Health and Children)

NCTE (2003), *Children's study – investigating online behaviour, Executive Summary, SAFT – Safety Awareness Fact and Tool* (Dublin, National Centre for Technology in Education).

NCSE (2006), *Guidelines on the Individual Education Plan Process* (Trim, Co. Meath, National Council of Special Education).

Newacheck, P. W. & Halfon, N. (1998), 'Prevalence and Impact of Disabling Chronic Conditions in Childhood', *Americal Journal of Public Health*, 88: 610–616.

NEWB (2005), *Pre-Budget Submission 2006*, (Dublin, National Education and Welfare Board).

Nicklas, T. A., Bao, W., Webber, L. S., & Berenson, G. S. (1993), 'Breakfast consumption affects adequacy of total daily intake in children', *Journal of the American Dietetic Association*, 93(8): 886–891.

Nolan, B., Gannon, B., Layte, R. Watson, D., Whelan, C.T. & Williams, J. (2002), 'Monitoring poverty trends in Ireland: Results from the 2000 Living in Ireland survey', *Policy Research Series*, (Nolan ESRI Policy Research Series, 45, Dublin)

Nunn, J. (2006), 'The burden of oral ill health for children', *Archives of Disease in Childhood* 91: 251–253.

O'Briain, E. (2006), *Analysis of School Attendance Data at Primary and Post-Primary Levels for 2004/2005*

O'Connor, T. G., Hetherington, E. M., & Clingempeel, W. G. (1997), 'Family systems and adolescent development: Implications for bidirectionality in development', *Journal of Social and Personal Relationships*, 14: 491–504.

O'Connor, T. G. & Jenkins, J. M. (2000), *Marital transitions and children's adjustment: Understanding why families differ from one another and why children in the same family show different patterns of adjustment* (Ottawa, Canada, Human Resources Development Canada).

ODPM - Office of the Deputy Prime Minister (1998), *English House Condition Survey, 1996* (London, The Statistics Office).

Olweus, D. (1995), 'Bullying or peer abuse at school: Facts and intervention', *Current Directions in Psychological Science*, 4: 200.

O'Moore, A. M., Kirkham, C., & Smith, M. (1997), 'Bullying behaviour in Irish schools: A nationwide study', *Irish Journal of Psychology*, 18: 141–169

O'Neill, P. (1992), *Telling It Like it Is* (Dublin, Combat Poverty Agency).

Parada, R.H., Marsh, H.W., Craven, R.G. and Papworth, B.A. (2005), *Bullying in Schools: What Can We Glean from Self-concept Theory?* (Australia, SELF Research Centre, University of Western Sydney).

Parkinson, P. & Smyth, B. (2003), *When the difference is night and day: Some empirical insights into patterns of parent-child contact after separation* (Melbourne, Paper presented at 8th Australian Institute of Family Studies Conference).

Patrick, D. L., Cheadle, A., Thompson, D., Diehr, P., Koepsell, T., & Kinne, S. (1994), 'The Validity of Self-Reported Smoking: A Review and Meta-Analysis', *Americal Journal of Public Health* 84[7]: 1086–1093.

Pettit, G. S., Laird, R. D., Bates, J. E., & Dodge, K. A. (1997), 'Patterns of after-school care in middle childhood: Risk factors and developmental outcomes', *Merrill-Palmer Quarterly*, 43: 515–538.

Pianta, R. C. (1992), *Child-parent relationship scale* (Unpublished measure, University of Virginia).

Phillips, S. D. & Gleeson, J. P. (2007), *What we Know Now that we Didn't Know Then about the Criminal Justice System's Involvement in Families with whom Child Welfare Agencies have Contact: Findings from a Landmark National Study* (Chicago, Center for Social Policy and Research, Jane Addams College of Social Work, University of Illinois at Chicago).

Piers,E.V. (1963), *Factor Analysis for the Piers-Harris Children's Self-Concept Scale* (un published).

Piers, E. V., & Herzberg, D. S. (2007), *Piers-Harris Children's Self-Concept Scale-Second Edition Manual* (Los Angeles, Cal., Western Psychological Services),.

Pilowsky, D. J. & Zybert, P. A. &. V. D. (2004), 'Resilient children of injection drug users', *Journal of American Academy of Child and Adolescent Psychiatry*, 43: 1372–1379.

Prescott, C. A., McArdle, J. J., Hishinuma, E. S., Johnson, J. C., Miyamoto, R. H., Andrade, N. N., Edman, J. L., Makini, G. K., Nahulu, L. B., Yuen, N. Y. C., & Carlton, B. S. (1998), 'Prediction of major depression and dysthymia from CES-D scores among ethnic minority adolescents', *Journal of American Academy of Child Adolescent Psychiatry,* 37(5): 495–503.

Prior, M. (1992), 'Childhood temperament', *Journal of Child Psychology and Psychiatry*, 33: 249–279.

Raine, A., Brennan, P., & Mednick, S. A. (1994), ‘Birth complications combined with early maternal rejection at age one year predispose to violent crime at age 18 years’, *Archives of General Psychiatry*, 51: 984–988.

Rampersaud, G. C., Pereira, M. A., Girard, B. L., Adams, J., & Metzl, J. D. (2005), ‘Breakfast habits, nutritional status, body weight, and academic performance in children and adolescents’, *Journal of the American Dietetic Association,* 105: 743–760.

Redmond, S.M. & Rice M.L. (1998), ‘The socio-emotional behaviours of children with Speech and Language Impairment: Social adaption or social deviance?’, *Journal of Speech, Language and Hearing Research,* 41: 688–700

Riley, D. & Steinberg, J., (2004). Four Popular Stereotypes About Children in Self-Care: Implications for Family Life Educators. Family Relations, 53, 95-101.

Rimal, R. N. (2003), ‘Intergenerational Transmission of Health: The Role of Intrapersonal, Interpersonal, and Communicative Factors’, *Health Education and Behaviour*, 30: 10–28.

Riordan, S. (2001), *Supporting Parenting: A Study of Parents' Support Needs* (Dublin, Department of Social, Community and Family Affairs).

Roberts, R. E., Andrews, J. A., Lewinsohn, P. M., & Hops, H. (1990),. *Assessment of depression in adolescents using the Center of Epidemiologic Studies Depression Scale*, *Journal of Consulting and Clinical Psychology,* 2: 122–128.

Roberts, I. & Power, C. (1996), ‘Does the decline in mortality vary by social class? A comparison of class specific mortality in 1981 and 1991’, *British Medical Journal*, 313: 784–786.

Robinson, T. N. (1999), ‘Reducing Children's Television Viewing to Prevent Obesity: A Randomized Controlled Trial’, *The Journal of the American Medical Association*, 282: 1561–1567.

Roby, D. E. (2004), ‘Research on school attendance and student achievement: A study of Ohio schools’, *Education Research Quarterly*, 28: 3–4.

Rowe, D.C. & Plomin, R. (1977),’ Temperament in early childhood’, *Journal of Personality Assessment*, 41: 150–156.

Rutter, M. (1987), ‘Temperament, personality and personality disorder’, *British Journal of Psychiatry*, 150: 443–558.

Rutter, M. & Maughan, B. (2002), ‘School Effectiveness Findings 1979–2002’, *Journal of School Psychology*, 40: 451–475.

Sallis, J. F., Taylor, A. H., Dowda, M., Freedson, P. S. & Pate, R. R. (2001), *Correlates of vigorous physical activity for children in grades in 1 through 12: Comparing parent reported and objectively measured physical activity* (Unpublished work).

Samson, A. et al. (2005), *Summarising children’s wellbeing: The LSAC Outcome Index. LSAC Technical Paper No 2* (Melbourne, Australian Inst. of Family Studies).

Santrock, J. W. (1998), Child Development (International Edition), 8th edn. (Boston, McGraw Hill).

Schneider, M. (2002*), Do School Facilities Affect Academic Outcomes? National Clearinghouse for Educational Facilities* [On-line]. Available: http://www.edfacilities.org/pubs/outcomes.pdf

Scragg, R., Laugesen, M. & Robinson, E. (2002), ‘Cigarette smoking, pocket money and socioeconomic status: Results from a national survey of 4th form students in 2000’, *The New Zealand Medical Journal*, 115: 1158. Retrieved online 14/12/2007 from http://www.nzma.org.nz/journal/115-1158/108.

Shaarani, S., Walsh, T., Khawaja, N., Collins, C., Byrne, P., Geary, M. & Harrison, R. F.

Alcohol and cigarettes in the pregnant Irish population (forthcoming).

Sharpley, C. F., & Cross, D. G. (1982), ‘A psychometric evaluation of the Spanier Dyadic Adjustment Scale’, *Journal of Marriage and the Family*, 44: 739–741.

Sharpley, C. F. and Rogers, H. J. (1984),’Preliminary Validation of the Abbreviated Spanier Dyadic Adjustment Scale: Some Psychometric Data Regarding a Screening test of marital Adjustment’, *Educational and Psychological Measurement*, 44: 1045–1049.

Skevik, A. (2006), ‘ “Absent fathers” or “reorganized families”? Variations in father-child contact after parental break-up in Norway’, *The Sociological Review*, 54: 114–132.

Smedje, H., Broman, E., Hetta, J., & von Knorring, A. L. (1999), ‘Psychometric properties of a Swedish version of the Strengths and Difficulties Questionnaire’, *European Child and Adolescent Psychiatry* 8: 63–70.

Smith, H. G. (1993), ‘Intervention strategies for children vulnerable for school failure due to exposure to drugs and alcohol’*, International Journal of Addiction*, 28: 1435–1470.

Smith, P. & Ananiadou, K. (2003), ‘The Nature of School Bullying and the Effectiveness of School-Based Interventions’, *Journal of Applied Psychoanalytic Studies,* 5: 189–209.

Smolak, L., Levine, M. P., & Schermer, F. (1999), ‘Parental input and weight concerns among elementary school children’, *International Journal of Eating Disorders*, 25: 263–271.

Smyth, E. (1999), Do schools differ? Academic and personal development among pupils in the second-level sector. (General Research Series, 173 Insert place of publication and publisher).

Smyth, E. (2000), ‘Assessing school effectiveness: Evidence from the Irish context’, *Irish Educational Studies*, 19

Smyth, E. (2004), *Do Schools Differ: Academic and Personal Development among Pupils in the Second-Level Sector* (Dublin, Oak Tree Press).

Smyth, E., Byrne, D., & Hannan, C., (2004), *The Transition Year Programme: An Assessment* (Dublin, The Liffey Press in association with the ESRI).

Smyth, E., Dunne, A., Darmody, M., and McCoy, S. (2007), *Gearing Up for the Exam, the Experiences of Junior Certificate Students* (Dublin, The Liffey Press in association with the ESRI).

Smyth, E., McCoy, S., & Darmody, M. (2004), *Moving Up: The Experiences of First-year Students in Post-Primary Education* (Dublin, Liffey Press, ESRI).

Spanier, G. B. (1976), ‘Measuring dyadic adjustment: New scales for assessing quality of marriage and similar dyads’, *Journal of Marriage and the Family*, 38 (1): 15–28.

Starfield, B. & Budetti, P. P. (1985), ‘Child health status and risk factors’, *Health Services Research*, 19: 81–-886.

Steinberg, L., Elmen, J. D., & Mounts, N. S. (1989), 'Authoritative parenting, psychosocial maturity and academic success among adolescents', *Child Development*, 60: 1424–1436.

Steinberg, L., Lamborn, S. D. ,Dornbusch, S. M., & Darling, N. (1992), 'Impact of Parenting Practices on Adolescent Achievement: Authoritative Parenting, School Involvement, and Encouragement to Succeed', *Child Development* , 63: 1266–1281.

Strauss, R. S., Rodzilsky, D., Burack, G., & Colin, M. (2001), 'Psychosocial Correlates of Physical Activity in Healthy Children', *Archives of Pediatric and Adolescent Medicine*, 155: 897–902

Studts, J. L., Ghate, S. R., Gill, J. L., Studts, C. R., Barnes, C. N., LaJoie, A. S., Andrykowski, M. A., & LaRocca, R. V. (2006,. 'Validity of Self-reported Smoking Status among Participants in a Lung Cancer Screening Trial', *Cancer Epidemiology Biomarkers & Prevention* 15: 1825–1828.

Sweeting, H. & West, P. (2001), 'Being different: correlates of the experience of teasing and bullying at age 11', *Research Papers in Education*, 16: 225–246.

Thomas, D., Strauss, J. & Henriques, M-H. (1991), 'How does mother's education affect child height?', *The Journal of Human Resources*, 27, 2: 183–-211

Tienboon, P., Rutishauser, I. H., & Wahlqvist, M. L. (1994), 'Maternal recall of infant feeding practices after an interval of 14 to 15 years', *Australian Journal of Nutrition and Dietetics*, 51: 25–27.

Truby, D. (2001), 'Attendance makes the difference', *Instructor*, 110.

Van Houtte, B. A., & Jarvis, P. A. (1995), 'The role of pets in preadolescent psychosocial development', *Journal of Applied Developmental Psychology*, 16, 3: 463–479.

Van Odijk, J., Kull, I., Borres, P., Brandtzaeg, P., Edberg, U., Hanson, L. A., Host, A., Kuitunen, M., Olsen, S. F., Skerfving, S., Sundell, J., & Wille, S. (2003), 'Breastfeeding and allergic disease: a multidisciplinary review of the literature (1966-2001) on the mode of early feeding in infancy and its impact on later atopic manifestations', *Allergy,* 58: 833–843.

Videon, T., & Manning, C. (2003), 'Influences on adolescent eating patterns: The importance of family meals', *Journal of Adolescent Health*, 32: 365–373.

Walsh, K., Clerkin, P., & Nic Gabhainn, S. (2004), 'Relationship with mothers among Irish schoolchildren', in *Health Behaviour Survey of Children 2002* [On-line]. Available: Retrieved from http://www.nuigalway.ie/hbsc/documents/factsheet_12.pdf

Walton, K. A., Murray, L. J., Gallagher, A. M., Cran, G. W., Savage, M. J., & Boreham, C. (2000), 'Parental recall of birthweight: A good proxy for recorded birthweight?', *European Journal of Epidemiology*, 16[9]: 793–796.

Watson, D. & Williams, J. (2000). *Perceptions of the Quality of Health Care in the Public and Private Sector: Report to the Centre for Insurance Studies, Graduate Business School, UCD* (Dublin, ES RI).

Whelan, C. T., Layte, R., Maître, B., & Nolan, B. (2003), 'Persistent Income Poverty and Deprivation in the European Union: An Analysis of the First Three Waves of the European Community Household Panel', *Journal of Social Policy*, 32: 1–32.

Whelan, C.T., Maître, B. & Nolan, B. (2007), *Multiple deprivation and multiple disadvantage in Ireland: An analysis of EU-SILC. Policy Research Series, 6*. (Dublin,ESRI).

Wigfield, A., Battle, A., Keller, L.B., Eccles, J.S. (2002), ‘Sex Differences in Motivation, Self-Concept, Career Aspiration and Career Choice: Implications for Cognitive Development’,, in *Biology, Society and Behaviour: the Development of Sex Differences in Cognition,* 93–126 (Westport, CT, Ablex).

Willens, T. E., Biederman, J., Kiely, K., Bredin, E., Lic, S. W., & Spencer, T. J. (1995),, ‘Pilot study of behavioural and emotional disturbances in the high-risk children of parents with opioid dependence’, *Journal of American Academy of Child Adolescent Psychiatry*, 34: 779–785.

Williams, W. R., Latif, A. H. A., Hannington, L., & Watkins, D. R. (2005), ‘Hyperopia and educational attainment in a primary school cohort’, *Archives of Disease in Childhood*, 90: 150–153.

Yawn, B. P., Suman, V. J., & Jacobsen, S. J. (1998), ‘Maternal Recall of Distant Pregnancy Events’, *Journal of Clinical Epidemiology*, 51[5]: 399–405.

Zeiger, R. S. (2003), ‘Food allergen avoidance in the prevention of food allergy in infants and children’, Pediatrics, 111: 1662–1771.

If you would like further information about *Growing Up in Ireland,* please visit

www.growingup.ie

e-mail growingup@esri.ie

or freephone 1800 200 434
